# Age-related decline in nuclear envelope LINC complex drives neuronal aging via axon initial segment dysfunction

**DOI:** 10.1038/s44319-026-00786-5

**Published:** 2026-05-22

**Authors:** Koichi Hasegawa, Noriyuki Hama, Mina Amemiya, Chao Zeng, Yasuyuki Ito, Sadafumi Suzuki, Keiichiro Nakamura, Junpei Kondo, Chiharu Takeda, Yuji Kurihara, Kazuho Ikeda, Yuki Fujita, Yasushi Okada, Atsushi Toyoda, Michiaki Hamada, Ken-ichiro Kuwako

**Affiliations:** 1 Department of Neural and Muscular Physiology, School of Medicine, Shimane University, Izumo, Japan; 2 Faculty of Science and Engineering, Waseda University, Tokyo, Japan; 3 Department of Developmental Biology, School of Medicine, Shimane University, Izumo, Japan; 4 Department of Cell Biology, Graduate School of Medicine, The University of Tokyo, Tokyo, Japan; 5 Laboratory for Cell Polarity Regulation, RIKEN Center for Biosystems Dynamics Research (BDR), Osaka, Japan; 6 Department of Physics, Graduate School of Science, The University of Tokyo, Tokyo, Japan; 7 Universal Biology Institute (UBI), The University of Tokyo, Tokyo, Japan; 8 International Research Center for Neurointelligence (WPI-IRCN), Institutes for Advanced Study, The University of Tokyo, Tokyo, Japan; 9 Comparative Genomics Laboratory, Department of Genomics and Evolutionary Biology, National Institute of Genetics, Mishima, Japan; 10 AIST-Waseda University Computational Bio Big-Data Open Innovation Laboratory (CBBD-OIL), National Institute of Advanced Industrial Science and Technology, Tokyo, Japan; 11 Present Address: Department of Clinical Laboratory Science, Sanyo Women’s College, Hiroshima, Japan

**Keywords:** Chromatin, Transcription & Genomics, Molecular Biology of Disease, Neuroscience

## Abstract

Brain aging is an intricate process that inevitably leads to functional deterioration. However, its molecular drivers remain unclear. Here, we show that the age-related decline in LINC complex expression on the neuronal nuclear envelope impairs axon initial segment (AIS)-mediated excitability and triggers brain aging. With aging, the expression of LINC complex components, including Sun1, decreases in various brain regions, accompanied by a reduction in AIS length. Preserving Sun1 expression rescues nuclear structural abnormalities in aged neurons, shifting chromatin dynamics and global gene expression toward those of young neurons. Particularly, it restores the expression of AIS-related molecules, including voltage-gated sodium or potassium channels essential for action potential generation. Inhibiting the LINC complex in young mice impairs AIS integrity, leading to reduced neuronal excitability and brain dysfunction. Furthermore, Sun1 administration to aged neurons prevents age-related AIS shortening, excitability impairment, and brain function changes. Thus, we uncover the mechanism of normal brain aging involving AIS dysfunction, identifying the LINC complex component Sun1 as essential for preserving brain function.

## Synopsis

Age-related decline of the LINC complex component Sun1 disrupts neuronal nuclei and gene expression, causing axon initial segment (AIS) dysfunction, reduced excitability, and brain aging. Maintaining Sun1 prevents these abnormalities, highlighting its role in preserving brain function.

- Aging reduces neuronal LINC complex expression, leading to nuclear structural abnormalities and AIS shortening across multiple brain regions.
- Restoration of Sun1 in aged neurons preserves nuclear structure and chromatin dynamics, globally modulating gene expression, including AIS-related molecules essential for neuronal excitability.
- Dysfunction of the LINC complex in young neurons compromises nuclear structure, AIS-dependent excitability, and brain function.
- Sustained Sun1 expression in aged neurons preserves AIS integrity and prevents age-related declines in excitability and brain function.

## Abstract (web summary)

Age-related decline of the LINC complex component Sun1 disrupts neuronal nuclei and gene expression, causing axon initial segment (AIS) dysfunction, reduced excitability, and brain aging. Maintaining Sun1 prevents these abnormalities, highlighting its role in preserving brain function.

## Graphical abstract

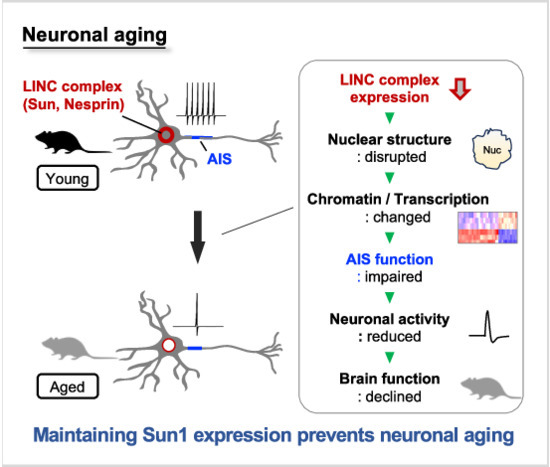

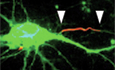

## Introduction

Aging drives the decline of brain functions through gradual neuronal impairment. It is influenced by multiple factors, such as mitochondrial dysfunction, oxidative stress, and metabolic dysregulation (Bishop et al, 2010; Jin and Cai, 2023). Although many age-related neurodegenerative disorders are characterized by extensive cell death, normal brain aging in humans and rodents is not primarily associated with neuronal loss (Burke and Barnes, 2006; Rapp and Gallagher, 1996; West et al, 1994). Conversely, during normal neuronal aging, intrinsic properties, such as dendritic arbor complexity and spine density (Markham and Juraska, 2002), epigenetic modifications and associated gene expression (Jin et al, 2025; Zhang et al, 2022), and neuronal excitability (Hickmott and Dinse, 2013; Randall et al, 2012), undergo significant changes, thereby leading to functional decline (Burke and Barnes, 2006; Rizzo et al, 2014). Since single-neuron activity underpins brain-wide network function, age-related changes in neuronal excitability are considered potential key drivers of cognitive decline (Rizzo et al, 2014). However, the molecular basis underlying changes in neuronal excitability with aging remains largely unknown.

Neuronal excitability relies on the axon initial segment (AIS), where action potentials are initially generated (Freal and Hoogenraad, 2025; Huang and Rasband, 2018; Leterrier, 2018). The AIS has a high density of specific voltage-gated sodium (Nav) and potassium (Kv) channels anchored by scaffold proteins, such as Ankyrin-G and PSD93 (Freal and Hoogenraad, 2025; Huang and Rasband, 2018; Leterrier, 2018). Nav1.1, Nav1.2, and Nav1.6 are expressed in the AIS and are essential for generating and modulating action potentials (Freal and Hoogenraad, 2025; Huang and Rasband, 2018; Leterrier, 2018). Particularly, Nav1.6 is a subtype crucial for determining the action potential threshold and is expressed across all neuron types. In contrast, Nav1.1 and Nav1.2 are expressed in inhibitory and excitatory neurons, respectively (Yamada and Kuba, 2016). The AIS exhibits activity-dependent structural plasticity in its length and position, changing the types and number of Nav or Kv channels it expresses, thereby diversifying its electrical properties (Freal and Hoogenraad, 2025; Huang and Rasband, 2018; Leterrier, 2018). Different types of neurons display distinct patterns in the structure, molecular composition, and plasticity of the AIS (Fried et al, 2009; Kuba et al, 2006; Lorincz and Nusser, 2008). Despite substantial evidence highlighting the AIS as a principal regulator of brain network homeostasis (Freal and Hoogenraad, 2025), its involvement in normal brain aging remains uncharacterized.

The linker of nucleoskeleton and cytoskeleton (LINC) complex consists of two families of nuclear envelope (NE) proteins: Sad1/UNC84 (SUN) domain proteins at the inner nuclear membrane, including Sun1 and Sun2, and Klarsicht/Abnormal nuclear anchorage-1/SYNE homology (KASH) domain proteins at the outer nuclear membrane, including nuclear envelope spectrin repeat protein-1 (Nesprin-1) and Nesprin-2 (Kuwako and Suzuki, 2024; Tapley and Starr, 2013). Nesprin interacts with cytoskeleton proteins, such as actin filaments and microtubules, while Sun binds to the nuclear lamina in the nucleus (Razafsky and Hodzic, 2009). The C-terminal KASH domain of Nesprin and the SUN domain of Sun interact within the NE lumen to form the LINC complex, which physically connects the cytoplasm and nucleoplasm (Jahed et al, 2021). The LINC complex transmits mechanical forces to the nucleus by interacting with the cytoskeleton, thereby regulating nuclear structure and movement across various cell types (Kuwako and Suzuki, 2024). The LINC complex is essential for brain development in mammals, particularly for cell migration and nuclear positioning (Goncalves et al, 2020; Horn et al, 2013; Zhang et al, 2009; Zhou et al, 2024). However, its homeostatic role in the mature nervous system remains poorly understood.

In this study, we demonstrate that the age-related decline in neuronal LINC complex expression underlies normal brain aging by impairing AIS-mediated intrinsic excitability. Progressive loss of LINC complex proteins with aging—particularly Sun1—induces nuclear structural abnormalities that alter global gene expression, affecting key AIS-associated molecules such as Nav and Kv channels. This results in impaired AIS integrity and neuronal excitability, ultimately leading to age-related changes in brain function. Our findings reveal a novel mechanism linking nuclear envelope changes to AIS dysfunction and neural aging, highlighting that targeting Sun1 in the LINC complex is a promising therapeutic strategy to preserve brain function during aging.

## Results

### Neuronal expression of the LINC complex declines with aging

**Figure 1:**
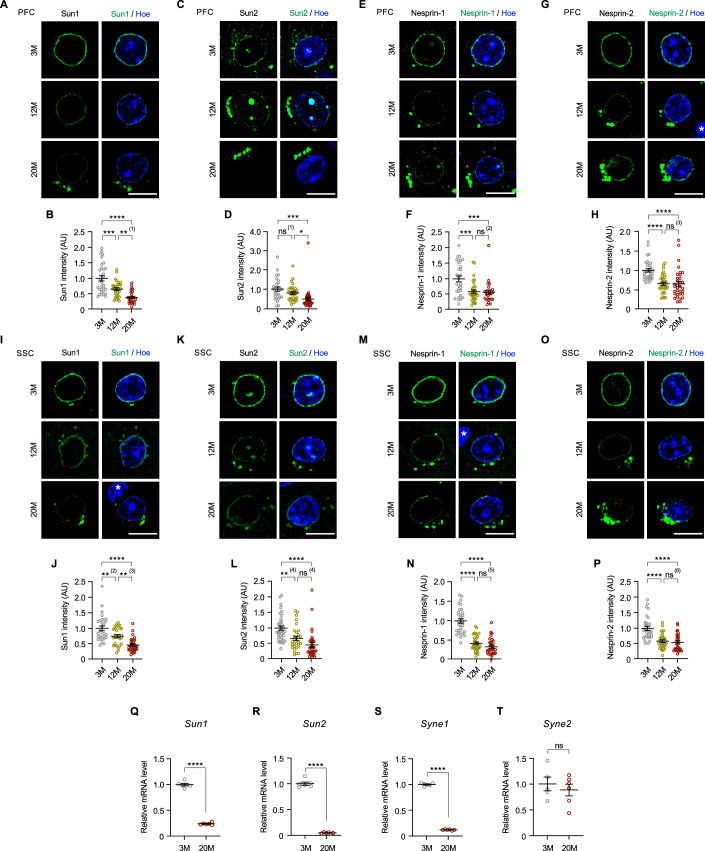
Age-related decline in the expression of LINC complex components in neurons. (**A**–**P**) Expression of LINC complex molecules. Brain sections from 3-, 12-, and 20-month-old mice were immunostained with antibodies against Sun1 (**A**, **I**), Sun2 (**C**, **K**), Nesprin-1 (**E**, **M**), or Nesprin-2 (**G**, **O**). Representative images are shown for the nuclei of pyramidal neurons in layer V of the prefrontal cortex (PFC) and layer II/III of the somatosensory cortex (SSC). Asterisks indicate presumptive glial cells. Signal intensity of Sun1 (**B**, **J**), Sun2 (**D**, **L**), Nesprin-1 (**F**, **N**), and Nesprin-2 (**H**, **P**) on the NE were quantified. The data represent the mean ± SEM. *n* = 32 (3 M), *n* = 31 (12 M), and *n* = 27 (20 M) for Sun1 in the PFC (**B**); *n* = 28 (3 M), *n* = 33 (12 M), and *n* = 35 (20 M) for Sun2 in the PFC (**D**); *n* = 30 (3 M), *n* = 33 (12 M), and *n* = 25 (20 M) for Nesprin-1 in the PFC (**F**); *n* = 33 (3 M, 12 M, and 20 M) for Nesprin-2 in the PFC (**H**); *n* = 30 (3 M), *n* = 29 (12 M), and *n* = 31 (20 M) for Sun1 in the SSC (**J**); *n* = 37 (3 M), *n* = 30 (12 M), and *n* = 33 (20 M) for Sun2 in the SSC (**L**); *n* = 32 (3 M), *n* = 32 (12 M), and *n* = 30 (20 M) for Nesprin-1 in the SSC (**N**); *n* = 31 (3 M), *n* = 30 (12 M), and *n* = 29 cells (20 M) for Nesprin-2 in the SSC (**P**) from three brains. *P = 0.0329; **P = 0.0096 (1), 0.0081 (2), 0.0022 (3), 0.0050 (4); ***P < 0.001; ****P < 0.0001; ns, not significant, *P* = 0.2827 (1), 0.9905 (2), 0.9986 (3), 0.1247 (4), 0.3740 (5), 0.8496 (6) (ordinary one-way ANOVA Tukey’s multiple comparison test). (**Q**–**T**) mRNA expression levels of LINC complex genes (*Sun1*, *Sun2*, *Syne1* encoding Nesprin-1, and *Syne2* encoding Nesprin-2) determined via qPCR analysis. The data represent the mean ± SEM. *n* = 6 (3 M and 20 M) for *Sun1* (**Q**); *n* = 6 (3 M and 20 M) for *Sun2* (**R**); *n* = 6 (3 M and 20 M) for *Syne1* (**S**); *n* = 5 (3 M) and *n* = 6 cerebral cortices (20 M) for *Syne2* (**T**). ****P < 0.0001; ns, not significant, *P* = 0.5238 (unpaired two-tailed Welch’s *t* test). Scale bars: 10 μm. Source data are available online for this figure.

To explore the importance of the LINC complex in neuronal homeostasis, we first investigated the age-related expression changes of its components. In 3-month-old mice, key molecules of the LINC complex—Sun1, Sun2, Nesprin-1, and Nesprin-2—were highly expressed on the NE of neurons across multiple brain regions, including the prefrontal, somatosensory, and motor cortices, as well as hippocampal CA3 (Fig. 1A–P; Appendix Fig. S1). However, their expression significantly decreased by 12 months and was dramatically reduced by 20 months across all these regions (Fig. 1A–P; Appendix Fig. S1B–Q), likely causing dysfunction of the LINC complex in aged neurons. The LINC complex molecules were not only downregulated in the NE but also misaccumulated exclusively in the Golgi apparatus in aged neurons (Appendix Fig. S2A). A similar accumulation was observed for Lamin B1 (Appendix Fig. S2B).

We further examined the age-related changes in the expression of LINC complex molecules per neural lineage. Glutamatergic and GABAergic neurons exhibited a significant reduction in the expression of Sun1, Sun2, Nesprin-1, and Nesprin-2 on the NE at 20 months, compared to that in 3-month-old mice (Appendix Fig. S3A–H). In contrast, astrocytes and oligodendrocytes expressed only Sun2, which was reduced in both glial cell types by 20 months (Appendix Fig. S3I–P). In addition, in neural stem cells, the expression of Sun2 increased at 20 months, whereas that of the other three LINC complex molecules remained stable (Appendix Fig. S3Q–T). These results suggest that aging induces cell-type-specific changes in the expression of LINC complex components in neuronal lineage cells, characterized by a coordinated reduction of all major components in neurons. We also investigated age-related changes in the mRNA levels of the LINC complex molecules in selectively isolated neurons of the cerebral cortex of young and aged mice. A marked decrease in the mRNA expression of Sun1, Sun2, and Nesprin-1 was observed at 20 months, without a significant reduction in Nesprin-2 expression (Fig. 1Q–T). This result suggests that aging leads to the transcriptional suppression of Sun1, Sun2, and Nesprin-1 in neurons.

### Age-related dysfunction of the LINC complex disrupts nuclear integrity of aged neurons

**Figure 2:**
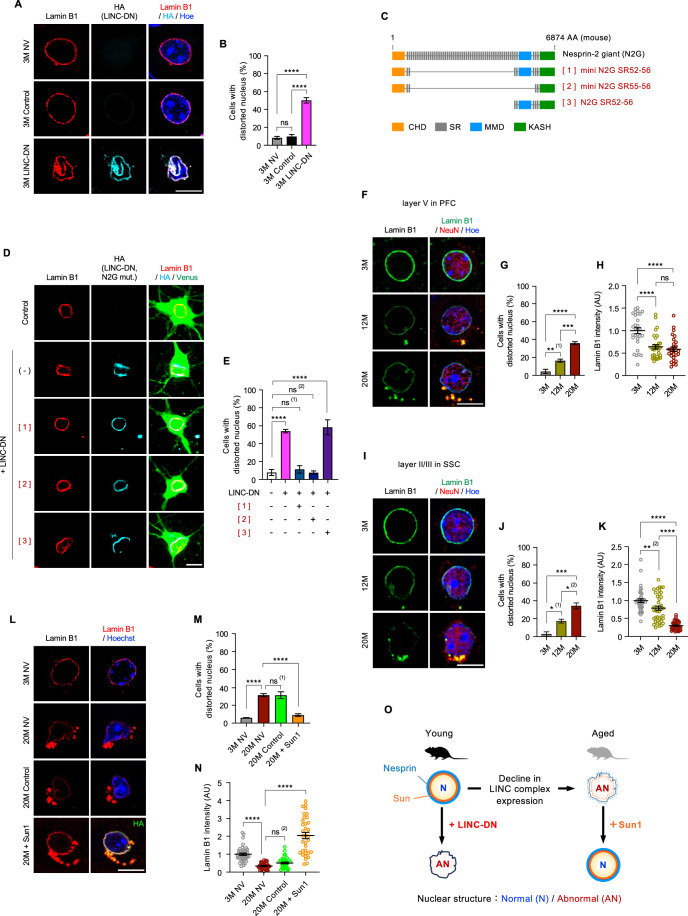
Age-related decline in the LINC complex compromises nuclear integrity in neurons. (**A**, **B**) Representative images of the nuclei of layer V pyramidal neurons in the prefrontal cortex of 3-month-old mice in no virus (3 M NV), AAV-Venus alone (3 M Control), and AAV-Venus + AAV-LINC-DN (3 M LINC-DN) groups (**A**). Cells with distorted nuclei, visualized by Lamin B1 staining, were quantified (**B**). The data represent the mean ± SEM. *n* = 4 brains, 14–20 cells per brain. ****P < 0.0001; ns, not significant, *P* = 0.8997 (ordinary one-way ANOVA Tukey’s multiple comparison test). (**C**) Schematic diagram of Nesprin-2 giant mutants analyzed in (**D**, **E**). CHD, calponin homology domain; SR, spectrin repeats; MMD, microtubule motor interaction domain; KASH, KASH domain. (**D**, **E**) Mini N2G SR52-56, mini N2G SR55-56, or N2G SR52-56 were expressed in cortical neurons with or without LINC-DN and analyzed at 21 days in vitro (DIV) for nuclear structure. Cells with distorted nuclei were quantified (**E**). The data represent the mean ± SEM. *n* = 3 independent experiments, 12–21 cells per experiment. ****P < 0.0001; ns, not significant, *P* = 0.7617 (1), >0.9999 (2) (ordinary one-way ANOVA Dunnett’s multiple comparison test). (**F**–**K**) Representative images are shown for the nucleus of layer V neurons in the prefrontal cortex (PFC) and layer II/III neurons in the somatosensory cortex (SSC) from 3-, 12-, and 20-month-old mice (**F**, **I**). Cells with distorted nuclei (**G**, **J**) and signal intensity of Lamin B1 on the NE (**H**, **K**) were quantified. The data represent the mean ± SEM. *n* = 4 brains, 11–17 cells per brain (**G**); *n* = 30 (3 M), *n* = 31 (12 M), and *n* = 28 cells (20 M) in the PFC from three brains (**H**); *n* = 3 brains, 13–15 cells per brain (**J**); *n* = 41 (3 M), *n* = 41 (12 M), and *n* = 44 cells (20 M) in the SSC from three brains (**K**). *P = 0.0224 (1), 0.0110 (2); **P = 0.0048 (1), 0.0036 (2); ***P < 0.001; ****P < 0.0001; ns, not significant, *P* = 0.7798 (ordinary one-way ANOVA Tukey’s multiple comparison test). (**L**–**N**) Brain sections from 3- or 20-month-old mice in no virus (3 M NV or 20 M NV), AAV-Venus (20 M Control), and AAV-Sun1 (20 M + Sun1) groups were co-immunostained with antibodies against Lamin B1 and HA (for Sun1). Representative images are shown for the nuclei of layer V pyramidal neurons in the prefrontal cortex (**L**). Cells with distorted nuclei (**M**) and signal intensity of Lamin B1 on the NE (**N**) were quantified. The data represent the mean ± SEM. *n* = 4 brains, 14–18 cells per brain (**M**); *n* = 41 (3 M NV), *n* = 38 (20 M NV), *n* = 39 (20 M Control), and *n* = 34 cells (20 M + Sun1) from four brains (**N**). ****P < 0.0001; ns, not significant, *P* > 0.9999 (1), *P* = 0.4053 (2) (ordinary one-way ANOVA Dunnett’s multiple comparison test). (**O**) Schematic diagram of the relationship between nuclear structure and the LINC complex in neurons. Scale bars: 10 μm. Source data are available online for this figure.

Next, we examined the effect of age-related decline in the expression of the LINC complex on nuclear structure in neurons. While the role of the LINC complex in regulating nuclear dynamics in migrating neurons is well understood, its contribution to maintaining the nuclear structural integrity in mature neurons remains unclear. Therefore, we first examined the nuclear structure after LINC complex inhibition in mature neurons. The dominant-negative (DN) tool targeting the LINC complex (hereafter referred to as LINC-DN), comprising only the KASH domain of Nesprin-1 that binds Sun, displaces endogenous Nesprins from the NE and strongly inhibits their activity (Razafsky and Hodzic, 2014; Stewart-Hutchinson et al, 2008) (Appendix Fig. S4A–E). To achieve widespread neuronal expression of LINC-DN, we used transvenous administration of a blood-brain barrier-penetrating adeno-associated virus (AAV) vector carrying the PHP.eB capsid (Chan et al, 2017) and a pan-neuronal synapsin1 promoter (Appendix Fig. S5). AAV-mediated neuronal delivery of LINC-DN in 1.5-month-old mice induced severe nuclear structural abnormalities in cortical neurons by 3 months of age, without causing cell death (Fig. 2A,B; Appendix Fig. S4F,G). This suggests that the LINC complex is crucial for maintaining the structural integrity of neuronal nuclei. We then determined the pivotal role of Nesprin-cytoskeletal interactions in regulating neuronal nuclear structure, using rescue experiments with Nesprin mutants lacking cytoskeletal-binding domains. A Nesprin-2 giant (N2G) mutant without most of the spectrin repeat (SR) domain (referred to as mini N2G SR52-56), which mimics the function of full-length Nesprin-2 (Goncalves et al, 2020), rescued nuclear structural abnormalities caused by LINC-DN in cultured cortical neurons (Fig. 2C–E). In addition, the mini N2G SR55-56 mutant, which lacks the microtubule interaction domain of mini N2G SR52-56, also achieved a complete rescue effect, whereas the N2G SR52-56 mutant, lacking the calponin homology domain essential for interaction with actin filaments, showed no rescue effect (Fig. 2C–E). Consistent with the critical role of the interaction between Nesprin and the perinuclear actin network (Maninova et al, 2017; Rothballer and Kutay, 2013), including the actin cap and TAN line, in transmitting mechanical forces to the nucleus, these results strongly suggest that the association of Nesprin with actin filaments is critical for the LINC complex-mediated maintenance of nuclear structural integrity in neurons.

**Figure EV1:**
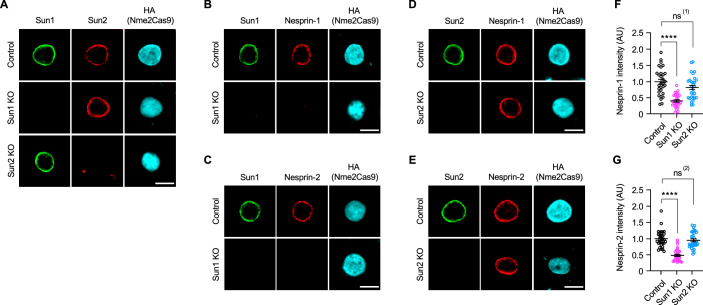
Effects of Sun deletion on Nesprin expression on the NE. (**A**–**E**) Control, Sun1 knockout (KO), and Sun2 KO cortical neurons at 21 DIV. (**F**, **G**) Signal intensity of Nesprin-1 (**F**) and Nesprin-2 (**G**) on the NE was quantified. The data represent the mean ± SEM. *n* = 30 cells from three independent experiments (Control, Sun1 KO, and Sun2 KO) (**F**, **G**). ****P < 0.0001; ns, not significant, *P* = 0.0822 (1), 0.6530 (2) (ordinary one-way ANOVA Dunnett’s multiple comparison test). Scale bars: 10 μm. Source data are available online for this figure.

**Figure EV2:**
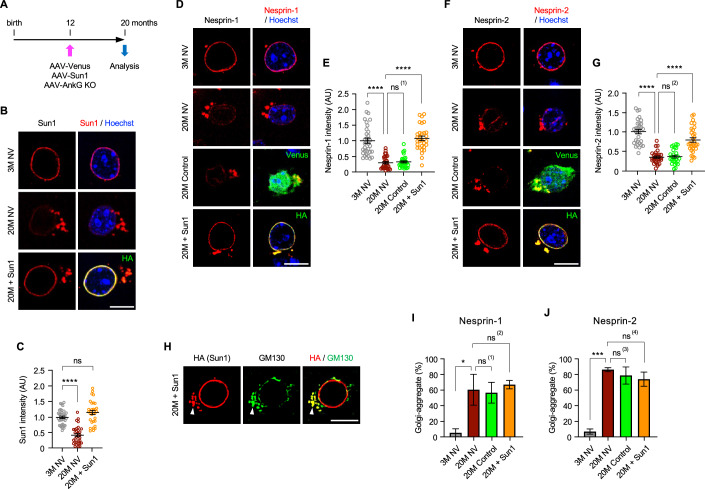
Analysis of LINC complex expression in AAV-administered mice. (**A**) Schematic of the AAV experiment to investigate the effects of Sun1 introduction and AIS disruption in aged neurons. 12-month-old mice were infected with AAV-Venus (20 M Control), AAV-Sun1 (20 M + Sun1), AAV-AnkG KO (20 M + AnkG KO), or AAV-Sun1 plus AAV-AnkG KO (20 M + Sun1 + AnkG KO), and analyzed at 20 months of age. (**B**, **C**) Analysis of Sun1 expression. Representative images are shown for layer V pyramidal neurons in the prefrontal cortex from 3- and 20-month-old mice in no virus (3 M NV or 20 M NV) and AAV-Sun1 (20 M + Sun1) groups (**B**). Signal intensity of Sun1 on the NE was quantified (**C**). The data represent the mean ± SEM. *n* = 37 (3 M NV), *n* = 32 (20 M NV), and *n* = 31 cells (20 M + Sun1) from three brains. ****P < 0.0001; ns, not significant, *P* = 0.0708 (ordinary one-way ANOVA Dunnett’s multiple comparison test). (**D**–**G**) Analysis of Nesprin expression in young and aged neurons. Brain sections from 3- or 20-month-old mice in no virus (3 M NV or 20 M NV), AAV-Venus (20 M Control), and AAV-Sun1 (20 M + Sun1) groups were co-immunostained with antibodies against Nesprin-1 (**D**) or Nesprin-2 (**F**), along with HA (for Sun1). Representative images are shown for layer V pyramidal neurons in the prefrontal cortex. Signal intensity of Nesprin-1 (**E**) and Nesprin-2 (**G**) on the NE were quantified. The data represent the mean ± SEM. *n* = 33 (3 M NV), *n* = 29 (20 M NV), *n* = 30 (20 M Control), and *n* = 30 (20 M + Sun1) for Nesprin-1 (**E**); *n* = 30 (3 M NV), *n* = 29 (20 M NV), *n* = 27 (20 M Control), and *n* = 31 cells (20 M + Sun1) for Nesprin-2 (**G**) from three brains. ****P < 0.0001; ns, not significant, *P* = 0.9797 (1), 0.9920 (2) (ordinary one-way ANOVA Dunnett’s multiple comparison test). (**H**) Localization of exogenous Sun1 in layer V pyramidal neurons in the prefrontal cortex of 20 M+Sun1 mice. Note that HA-tagged Sun1 localizes at the NE and GM130-labeled Golgi apparatus (arrowheads) in neurons. (**I**, **J**) Quantification of the percentage of cells containing five or more Golgi-localized aggregates (> 0.5 μm) of Nesprin-1 (**I**) and Nesprin-2 (**J**) in the experiments shown in (**D**, **F**). The data represent the mean ± SEM. *n* = 3 brains, 6–13 cells per brain (**I**, **J**). *P = 0.0347; ***P < 0.0005; ns, not significant, *P* = 0.9919 (1), 0.9658 (2), 0.8204 (3), 0.5478 (4) (ordinary one-way ANOVA Dunnett’s multiple comparison test). Scale bars: 10 μm. Source data are available online for this figure.

We next examined nuclear structure in aged neurons. In aged brains, the number of neurons with distorted nuclei was markedly increased in the prefrontal and somatosensory cortices compared with that of young brains, as previously observed in the visual cortex (Frey et al, 2023) (Fig. 2F,G,I,J). Furthermore, a decrease in Lamin B1 expression, a well-established marker of cellular senescence (Freund et al, 2012; Meqbel et al, 2022), was observed in these brain regions of aged mice (Fig. 2H,K). We then aimed to clarify whether the age-related decline in LINC complex expression on the NE drives nuclear structural abnormalities in aged neurons. Since the deletion of Sun1, but not Sun2, resulted in the exclusion of Nesprin-1 and Nesprin-2 from the NE in cultured cortical neurons (Fig. EV1; Appendix Table S1A,B), we hypothesized that the reduction in Sun1 expression may trigger the downregulation of Nesprins in aged neurons. Using the AAV system previously described, we expressed Sun1 or Venus (as a control) in the neurons of 12-month-old mice (Appendix Fig. S5). In 20-month-old mice that were administered AAV-Sun1 (20 M+Sun1 mice, Fig. EV2A), Sun1 expression levels on the NE of pyramidal neurons in the prefrontal cortex increased to levels similar to those in 3-month-old wild-type mice (Fig. EV2B,C). Supporting our hypothesis, the cortical neurons in 20 M+Sun1 mice showed a marked recovery of Nesprin-1 and Nesprin-2 expression on the NE, whereas no such changes were observed in aged mice administered AAV-Venus, suggesting that the LINC complex function may be specifically and significantly restored in the neurons of 20 M+Sun1 mice (Fig. EV2A,D–G). We found that the age-induced nuclear structural abnormalities were largely suppressed in 20 M+Sun1 mice, accompanied by a significant recovery of Lamin B1 expression levels (Figs. 2L–N and EV2A). These results strongly suggest that the LINC complex is essential for maintaining the normal nuclear structure in mature neurons, and its downregulation with aging is detrimental to nuclear integrity (Fig. 2O).

### LINC complex dysfunction with aging alters AIS-related gene expression in neurons

**Figure 3:**
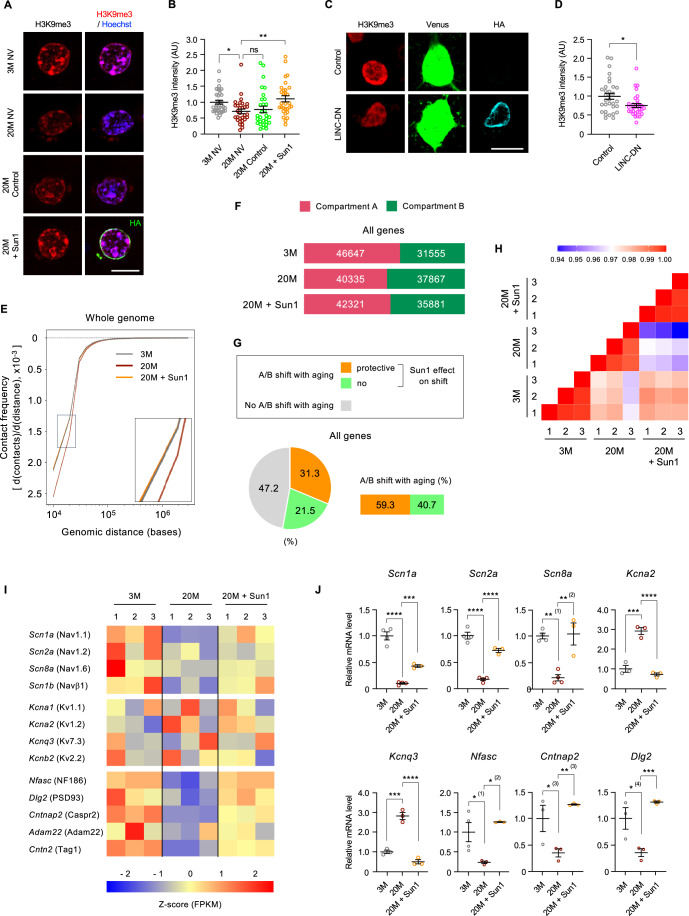
Age-related decline of the LINC complex in neurons alters the expression of genes associated with the AIS. (**A**, **B**) Neuronal H3K9me3 signals in vivo. Brain sections from 3- or 20-month-old mice in no virus (3 M NV or 20 M NV), AAV-Venus (20 M Control), and AAV-Sun1 (20 M + Sun1) groups were co-immunostained with antibodies against H3K9me3 and HA (for Sun1). Representative images are shown for the nuclei of layer V pyramidal neurons in the prefrontal cortex (**A**). H3K9me3 intensity was quantified (**B**). The data represent the mean ± SEM. *n* = 33 (3 M NV), *n* = 30 (20 M NV), *n* = 30 (20 M Control), and *n* = 30 cells (20 M + Sun1) from four brains. *P = 0.0425; **P = 0.0040; ns, not significant, *P* = 0.9217 (ordinary one-way ANOVA Dunnett’s multiple comparison test). (**C**, **D**) H3K9me3 signals in control and LINC-DN-expressing cortical neurons at 21 DIV (**C**). H3K9me3 intensity was quantified (**D**). The data represent the mean ± SEM. *n* = 32 (Control) and *n* = 31 cells (LINC-DN) from three independent experiments. *P = 0.0114 (unpaired two-tailed Welch’s *t* test). (**E**) Distance-dependent contact frequency profiles across the whole genome in AAV-eGFP-Cre alone (3 M or 20 M) and AAV-eGFP-Cre + AAV-Sun1 (20 M + Sun1) groups. The boxed region is magnified in the inset. d(contacts)/d(distance) represents the derivative of the contact number according to distance. (**F**) A/B compartment analysis across all genes. The number of genes is indicated in the graphs. (**G**) Percentage of age-related A/B compartment shift across all genes. Note that 52.8% of the genes undergo A/B shifts with aging (indicated in orange and green in the left pie chart), whereas 59.3% of these shifts are protected by Sun1 (indicated in orange in the right bar graph). (**H**) Correlation heatmap of gene expression across the three indicated groups. Each group consists of three mice. The color scale represents the Pearson correlation across all genes. (**I**) Heatmap of selected genes encoding AIS-related molecules, including Nav and Kv channels. DEGs were defined as those with |log2(fold_change)| >1, *P* < 0.001. Fragments per kilobase of exon per million mapped reads (FPKM) values were subjected to gene-wise z-score normalization before generating the heatmap. Each group consisted of three mice. The terms in the parentheses represent the proteins. (**J**) mRNA expression levels of AIS-related genes determined via qPCR analysis. The data represent the mean ± SEM. *n* = 4 (3 M and 20 M) and *n* = 3 (20 M + Sun1) for *Scn1a, Scn2a*, and *Scn8a*; *n* = 3 (3 M, 20 M, and 20 M + Sun1) for *Kcna2*, *Kcnq3*, *Cntnap2*, and *Dlg2*; *n* = 4 (3 M) and *n* = 3 cerebral cortices (20 M and 20 M + Sun1) for *Nfasc*. *P = 0.0290 (1), 0.0102 (2), 0.0393 (3), 0.0190 (4); **P = 0.0012 (1), 0.0014 (2), 0.0092 (3); ***P < 0.005; ****P < 0.0001 (ordinary one-way ANOVA Dunnett’s multiple comparison test). Scale bars: 10 μm. Source data are available online for this figure.

Nuclear structural abnormalities severely impact chromatin dynamics, leading to global gene expression changes (Kalukula et al, 2022). The reduction of the histone modification H3K9me3 is a hallmark of age-related chromatin changes, observed in aged neurons of wild-type mice, as well as in fibroblasts derived from patients with Hutchinson-Gilford progeria syndrome (HGPS), a premature aging disorder (Chen et al, 2012; Zhang et al, 2022). In the cortical neurons of 20 M+Sun1 mice, the age-related reduction in H3K9me3 levels was significantly restored to those in young mice (Figs. 3A,B and EV2A). Conversely, H3K9me3 levels were reduced in LINC-DN-expressing cultured cortical neurons (Fig. 3C,D). These findings suggest that nuclear structural abnormalities caused by LINC complex dysfunction induce alterations in chromatin dynamics in neurons.

**Figure EV3:**
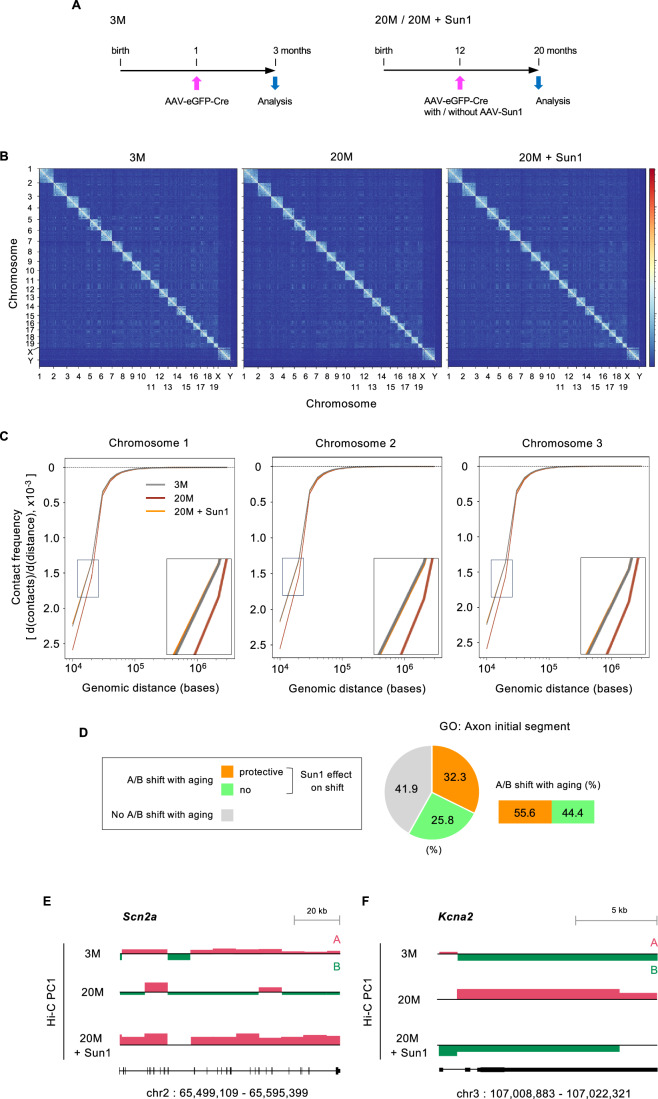
Analysis of genome-wide chromatin structure in young and aged neurons. (**A**) Schematics of the AAV experiment for genome analyses. 1- or 12-month-old mice were infected with AAV-eGFP-Cre to label the neuronal nuclei, and analyzed at 3 or 20 months of age, respectively (3 M or 20 M). In a separate group, 12-month-old mice were co-infected with AAV-Sun1 and AAV-eGFP-Cre, and analyzed at 20 months of age (20 M + Sun1). (**B**) Contact map across the whole genome in AAV-eGFP-Cre alone (3 M or 20 M) and AAV-eGFP-Cre + AAV-Sun1 (20 M + Sun1) groups. (**C**) Distance-dependent contact frequency profiles on chromosomes 1–3. The boxed regions are magnified in the inset. d(contacts)/d(distance) represents the derivative of the contact number according to distance. (**D**) Percentage of age-related A/B compartment shift for the GO term “Axon initial segment.” Note that 58.1% of the genes undergo A/B shifts with aging (indicated in orange and green in the left pie chart), whereas 55.6% of these shifts are inhibited by Sun1 (indicated in orange in the right bar graph). (**E**, **F**) A/B compartment analysis of the *Scn2a* (**E**) and *Kcna2* (**F**) loci. Source data are available online for this figure.

Then, we analyzed genome-wide high-order chromatin structures and gene expression in selectively isolated cortical neurons of young and aged mice using Hi-C and RNA-seq analyses, respectively (Fig. EV3A). In aged neurons, the chromatin interaction frequency was increased on each chromosome and across the whole genome compared to that of young neurons, and this increase was suppressed in 20 M+Sun1 mice neurons (Figs. 3E and EV3B,C). Furthermore, nuclear A/B compartment analysis revealed that, across all genes, aged neurons exhibited a decrease in the number of genes classified into compartment A, which are genomic regions with active histone modifications, and exhibited an increase in the number of genes classified into compartment B, which are genomic regions with repressive histone modifications, compared to those of young neurons (Fig. 3F). However, in the neurons of 20 M+Sun1 mice, the distribution of genes in compartments A and B showed a partial shift toward the pattern observed in young neurons (Fig. 3F). In addition, 52.8% of all genes underwent an A/B compartment shift with aging, whereas 59.3% of these genes were protected from this shift and retained the compartment pattern of young neurons following Sun1 introduction (Fig. 3G). These results suggest that sustaining the Sun1 levels in aged neurons reduces age-related changes in chromatin structure.

**Figure EV4:**
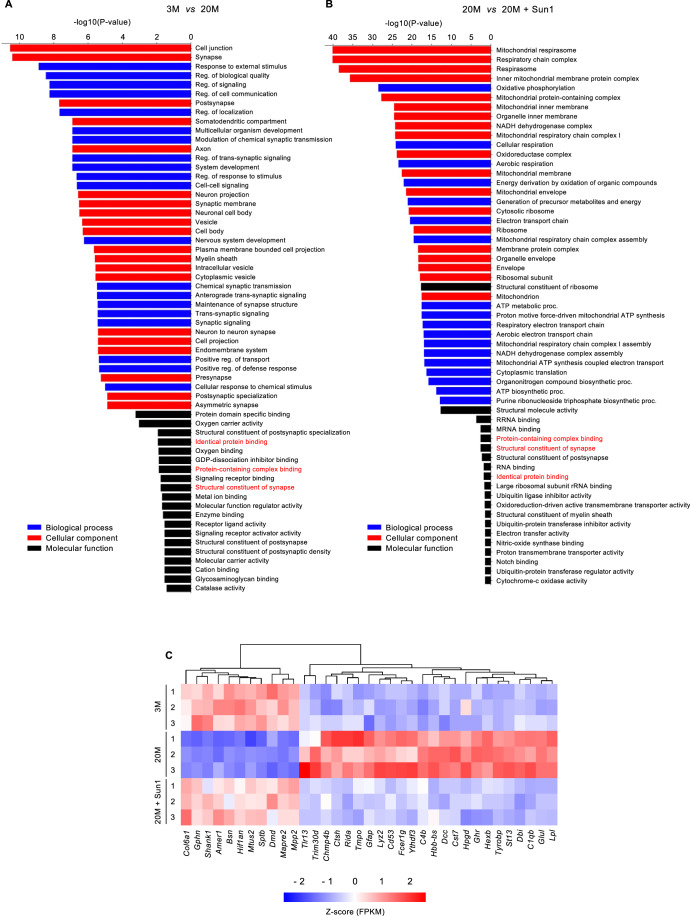
Analysis of gene expression in young and aged neurons. (**A**, **B**) GO analysis for AAV-eGFP-Cre alone (3 M or 20 M) and AAV-eGFP-Cre + AAV-Sun1 (20 M + Sun1) groups. The three GO terms highlighted in red showed significant expression changes in both 3 M *vs* 20 M (**A**) and 20 M *vs* 20 M + Sun1 (**B**). *P* values were derived from hypergeometric tests and adjusted using the Benjamini–Hochberg method. (**C**) Heatmap of clustered DEGs associated with GO terms highlighted in red in (**A**, **B**). The line above the heatmap indicates the classification of expression similarity of the 3 M and 20 M + Sun1 groups relative to the 20 M group. Each group consists of three mice. DEGs were defined as those with |log2(fold_change)| >1, *P* < 0.001. FPKM values were subjected to gene-wise z-score normalization before generating the heatmap. Source data are available online for this figure.

Analysis of expression correlations across all genes revealed that neurons of 20 M+Sun1 mice exhibited changes in their expression profiles that differed from those of control-aged neurons, showing an increased correlation with young neurons (Fig. 3H). Gene ontology (GO) analysis identified gene categories with expression changes during aging (comparison of young and aged neurons) and anti-aging (comparison of control-aged neurons and Sun1-introduced aged neurons) (Fig. EV4A,B). Among these categories, a specific subset of genes showed a pronounced shift toward a young neuron expression pattern due to Sun1 introduction (Fig. EV4C).

We further explored gene expression changes potentially contributing to age-related decline of neuronal function and identified significant changes in genes critical for AIS function in aged neurons (Fig. 3I). Aged neurons showed a dramatic decrease in the expression of AIS-localized Nav channels compared to that of young neurons, including Nav1.1, Nav1.2, and Nav1.6, which are essential for action potential generation (Yamada and Kuba, 2016), while those of Kv channels, including Kv1.2 and Kv7.3, which suppress action potential generation by counteracting Nav channels (Yamada and Kuba, 2016), were significantly increased (Fig. 3I,J), suggesting that these changes may underlie the reduced neuronal excitability in aged neurons. Notably, the age-related changes in the expression of these genes were substantially suppressed in the neurons of 20 M+Sun1 mice, resulting in expression levels that shifted closer to those of young neurons (Fig. 3I,J). In addition, the expression of AIS membrane-associated molecules, such as NF186, Caspr2, and PSD93, was reduced in control-aged neurons but restored in 20 M+Sun1 mice neurons (Fig. 3I,J). Chromatin structure analysis revealed that 58.1% of the genes associated with the GO term “Axon initial segment” underwent an A/B compartment shift with aging, and that 55.6% of these genes were protected from this shift following Sun1 introduction (Fig. EV3D). Specifically, the *Scn2a* and *Kcna2* genes, which encode Nav1.2 and Kv1.2, respectively, exhibited A/B compartment shifts with aging, which were suppressed by Sun1 introduction (Fig. EV3E,F). These results suggest that age-related decline in the expression of LINC complex components, including Sun1, modifies the expression of molecules essential for AIS function, such as Nav and Kv channels, by disrupting nuclear structural integrity in neurons.

### Aging induces changes in the molecular expression and structure of the AIS

**Figure 4:**
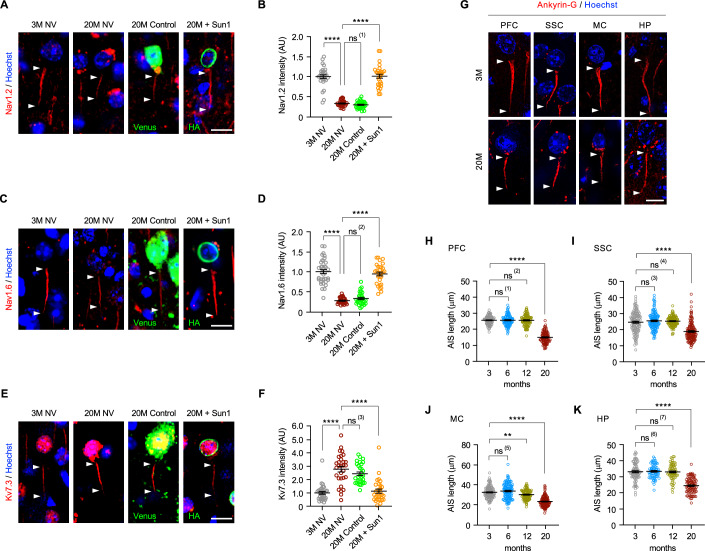
Aging induces changes in the expression of AIS-localized ion channels and AIS structure. (**A**–**F**) Expression analysis of Nav and Kv channels at the AIS. Brain sections from 3- or 20-month-old mice in no virus (3 M NV or 20 M NV), AAV-Venus (20 M Control), and AAV-Sun1 (20 M + Sun1) groups were co-immunostained with antibodies against Nav1.2, Nav1.6, Kv7.3, Ankyrin-G, and HA (for Sun1). Representative images are shown for layer V pyramidal neurons in the prefrontal cortex (**A**, **C**, **E**). The Nav or Kv channels on the AIS is indicated by the two arrowheads, and the total intensity of AIS-localized Nav1.2 (**B**), Nav1.6 (**D**), and Kv7.3 (**F**) in the AIS were quantified. AU, arbitrary unit. The data represent the mean ± SEM. *n* = 30 (3 M NV, 20 M NV, and 20 M + Sun1) and *n* = 34 (20 M Control) (**B**); *n* = 36 (3 M NV) and *n* = 30 (20 M NV, 20 M Control, and 20 M + Sun1) (**D**); *n* = 33 (3 M NV) and *n* = 30 cells (20 M NV, 20 M Control, and 20 M + Sun1) (**F**) from three brains. ****P < 0.0001; ns, not significant, *P* = 0.8170 (1), 0.6694 (2), 0.2749 (3) (ordinary one-way ANOVA Dunnett’s multiple comparison test). (**G**–**K**) Analysis of AIS structure. Representative images are shown for the AIS in pyramidal neurons in layer V of the prefrontal cortex (PFC), layer II/III of the somatosensory cortex (SSC), layer V of the motor cortex (MC), and CA3 neurons in the hippocampus (HP) from 3- and 20-month-old mice (**G**). The Ankyrin-G-localized axonal structure, indicated by the two arrowheads, defines the AIS. The AIS length in the PFC (**H**), SSC (**I**), MC (**J**), and HP (**K**) from 3-, 6-, 12-, and 20-month-old mice was quantified. The data represent the mean ± SEM. *n* = 100 (3 M, 6 M, 12 M, and 20 M) in the PFC (**H**); *n* = 120 (3 M), *n* = 119 (6 M), *n* = 120 (12 M), and *n* = 128 (20 M) in the SSC (**I**); *n* = 115 (3 M), *n* = 117 (6 M), *n* = 111 (12 M), and *n* = 131 (20 M) in the MC (**J**); *n* = 84 (3 M), *n* = 70 (6 M), *n* = 68 (12 M), and *n* = 94 cells (20 M) in the HP (**K**) from three brains. **P = 0.0096; ****P < 0.0001; ns, not significant, *P* > 0.9999 (1), *P* = 0.9991 (2), 0.4700 (3), 0.6621 (4), 0.2264 (5), 0.9737 (6), 0.9982 (7) (ordinary one-way ANOVA Dunnett’s multiple comparison test). Scale bars: 10 μm. Source data are available online for this figure.

We next investigated age-related changes in the protein expression of Nav and Kv channels at the AIS of pyramidal neurons in the prefrontal cortex, as well as the effect of Sun1 introduction on these changes in aged neurons. Consistent with the mRNA expression analyses (Fig. 3I,J), the protein levels of Nav1.2 and Nav1.6, the key Nav channels for action potential generation at the AIS of excitatory neurons, were significantly reduced at the AIS of control-aged neurons, but were markedly increased in aged neurons following Sun1 introduction (Figs. 4A–D and EV2A). In contrast, Kv7.3 expression at the AIS increased with aging but was suppressed by Sun1 introduction (Fig. 4E,F). These results suggest that an increase in Sun1 levels may significantly affect AIS-mediated regulation of neuronal excitability in aged neurons. We also examined the AIS morphology in aged neurons. In the prefrontal cortex, the length of the AIS in pyramidal neurons remained stable between 3 and 12 months of age; however, it was significantly shortened by 20 months (Fig. 4G,H). A similar reduction in AIS length was observed in the neurons of the somatosensory and motor cortices, as well as hippocampal CA3, which are regions where aging induced a marked decrease in the expression of LINC complex molecules (Fig. 4G,I–K). These results indicate that age-related structural changes in the AIS are extensive across brain regions, coinciding with the reduced expression of the LINC complex.

### LINC complex is essential for the structural organization of the AIS

**Figure 5:**
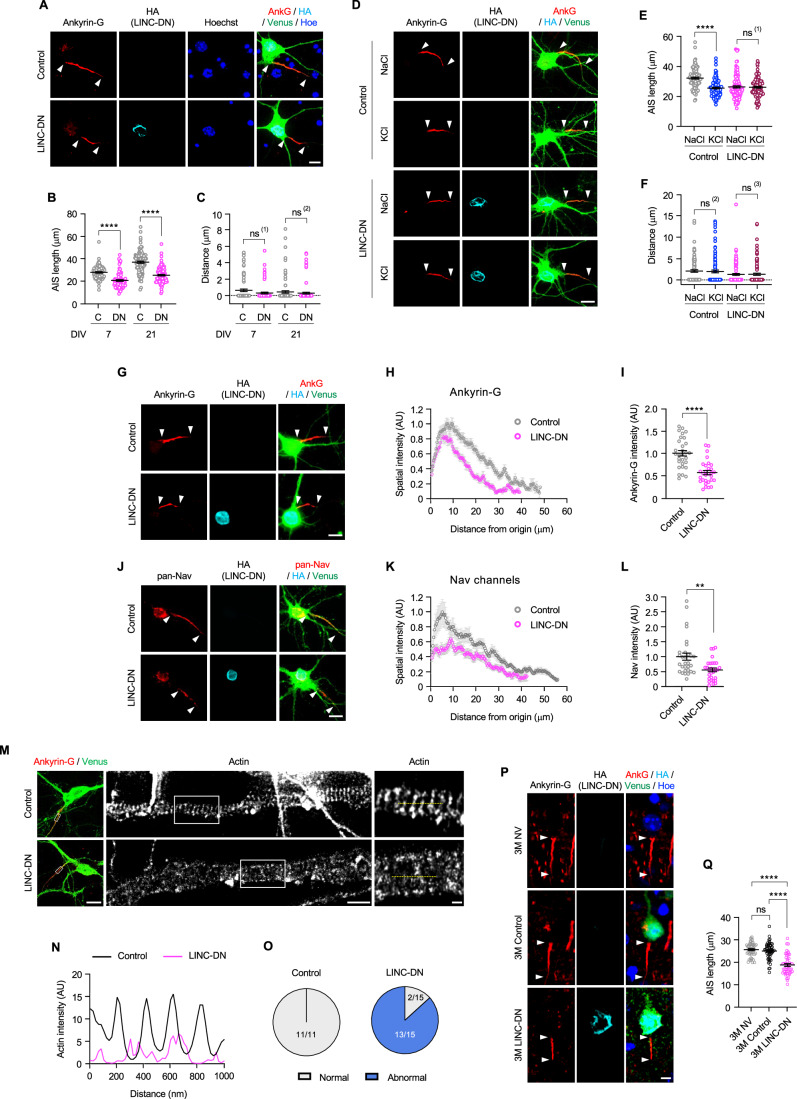
LINC complex is essential for maintaining AIS integrity. (**A**–**C**) Control and LINC-DN-expressing cortical neurons at 7 or 21 (**A**) DIV. The AIS is indicated by the two arrowheads, and its length (**B**) and position (**C**), measured as the distance from the soma, were quantified. C, Control; DN, LINC-DN. The data represent the mean ± SEM. *n* = 82 (C, 7 DIV), *n* = 83 (DN, 7 DIV), *n* = 101 (C, 21 DIV), and *n* = 101 cells (DN, 21 DIV) from three independent experiments (**B**, **C**). ****P < 0.0001 (unpaired two-tailed Welch’s *t* test for (**B**)]. ns, not significant, *P* = 0.1044 (1), 0.3688 (2) [unpaired two-tailed Mann–Whitney test for (**C**)). (**D**–**F**) Structural plasticity of the AIS under chronic depolarization. Control and LINC-DN-expressing cortical neurons at 12 DIV were treated with 10 mM NaCl or 10 mM KCl for 48 h (**D**). The AIS is indicated by the two arrowheads, and its length (**E**) and position (**F**) were quantified. The data represent the mean ± SEM. *n* = 101 (Control, NaCl), *n* = 100 (Control, KCl), *n* = 121 (LINC-DN, NaCl), and *n* = 104 cells (LINC-DN, KCl) from three independent experiments (**E**, **F**). ****P < 0.0001; ns, not significant, *P* = 0.8393 (1), 0.3197 (2), 0.6380 (3) (unpaired two-tailed Welch’s *t* test for (**E**)). ns, not significant (unpaired two-tailed Mann–Whitney test for (**F**)). (**G**–**L**) Spatial profiles of Ankyrin-G (**G**–**I**) and Nav channels (**J**–**L**) in control and LINC-DN-expressing cortical neurons at 21 DIV. The AIS is indicated by the two arrowheads. The spatial intensity (**H**, **K**) and total intensity (**I**, **L**) of Ankyrin-G and Nav channels in the AIS were quantified. AU, arbitrary unit. The data represent the mean ± SEM. *n* = 30 (Control and LINC-DN) (**H**, **I**); *n* = 30 cells (Control and LINC-DN) (**K**, **L**) from three independent experiments. **P = 0.0018; ****P < 0.0001 (unpaired two-tailed Welch’s *t* test). (**M**–**O**) Analysis of actin ring structure. STED images of actin ring structures in control and LINC-DN-expressing cortical neurons at 14 DIV (**M**). The AIS was identified by Ankyrin-G staining. Boxed regions in the left and middle panels are shown at higher magnifications in the middle and right panels, respectively. Spatial intensity profiles of actin along the yellow dotted line in the right panels in (**M**) are shown in (**N**). The periodicity of actin rings in the experiments shown in (**M**) was quantified (**O**). The number in the graph represents the number of analyzed neurons. (**P**, **Q**) Representative images of layer V pyramidal neurons in the prefrontal cortex of 3-month-old mice in no virus (3 M NV), AAV-Venus alone (3 M Control), and AAV-Venus + AAV-LINC-DN (3 M LINC-DN) groups (**P**). The AIS is indicated by the two arrowheads, and its length was quantified (**Q**). The data represent the mean ± SEM. *n* = 51 (3 M NV), *n* = 54 (3 M Control), and *n* = 48 cells (3 M LINC-DN) from three brains. ****P < 0.0001; ns, not significant, *P* = 0.6643 (ordinary one-way ANOVA Tukey’s multiple comparison test). Scale bars: 10 μm (**A**, **D**, **G**, **J**, the left panels in (**M**), and **P**); 1 μm (the middle panels in (**M**)); 200 nm (the right panels in (**M**)). Source data are available online for this figure.

**Figure EV5:**
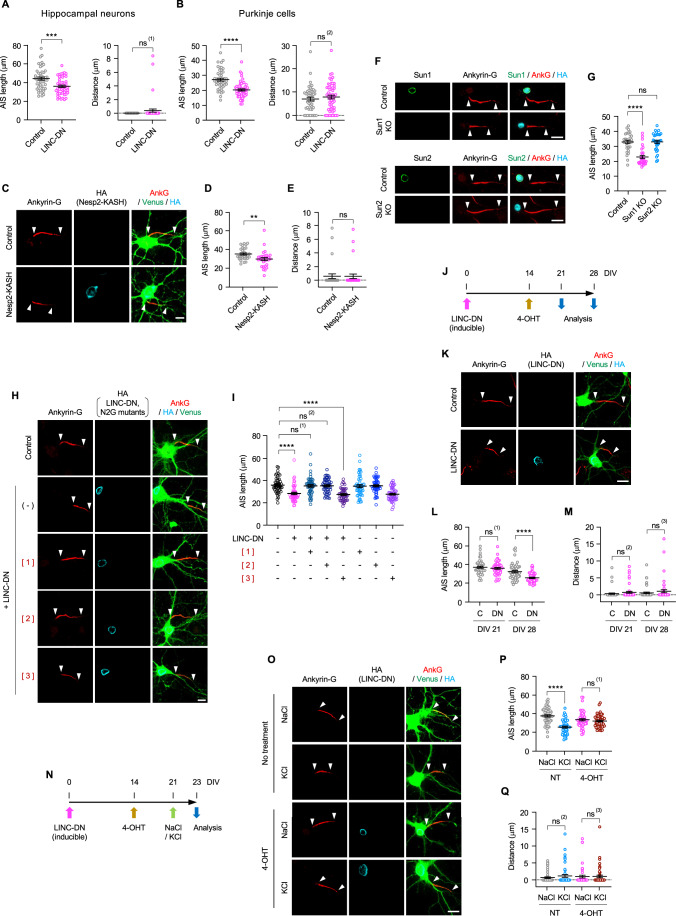
Effects of LINC complex inhibition on the AIS in vitro. (**A**, **B**) Quantification of AIS length and position, measured as the distance from the soma, in control and LINC-DN-expressing hippocampal neurons at 21 DIV (**A**) and Purkinje cells at 7 DIV (**B**). The data represent the mean ± SEM. *n* = 50 (Control and LINC-DN) (**A**); *n* = 47 (Control) and *n* = 55 cells (LINC-DN) (**B**) from three independent experiments. ***P < 0.0005; ****P < 0.0001 (unpaired two-tailed Welch’s *t* test for the left graphs in (**A**, **B**)). ns, not significant, *P* = 0.2424 (1), 0.4637 (2) (unpaired two-tailed Mann–Whitney test for the right graphs in (**A**, **B**)). (**C**–**E**) Effects of Nesp2-KASH expression on the AIS. Representative images of control and Nesp2-KASH-expressing cortical neurons at 21 DIV are shown (**C**). The AIS is indicated by the two arrowheads, and its length (**D**) and position (**E**) were quantified. The data represent the mean ± SEM. *n* = 30 cells from three independent experiments (Control and Nesp2-KASH) (**D**, **E**). **P = 0.0089 (unpaired two-tailed Welch’s *t* test for (**D**)). ns, not significant, *P* > 0.9999 (unpaired two-tailed Mann–Whitney test for (**E**)). (**F**, **G**) Effects of Sun deletions on the AIS. Representative images of control, Sun1 knockout (KO), and Sun2 KO cortical neurons at 21 DIV are shown (**F**). Nme2Cas9 (HA-tagged) was detected by immunostaining with an anti-HA antibody. The AIS is indicated by the two arrowheads, and its length was quantified (**G**). The data represent the mean ± SEM. *n* = 30 cells from three independent experiments (Control, Sun1 KO, and Sun2 KO). ****P < 0.0001; ns, not significant, *P* = 0.9995 (ordinary one-way ANOVA Dunnett’s multiple comparison test). (**H**, **I**) Analysis of cytoskeletal interactions essential for LINC complex-mediated regulation of the AIS. Mini N2G SR52-56, mini N2G SR55-56, or N2G SR52-56 (see schematic shown in Fig. 2C) were expressed in cortical neurons with or without LINC-DN and analyzed at 21 DIV for AIS structure (**H**). The AIS is indicated by the two arrowheads, and its length was quantified (**I**). The data represent the mean ± SEM. *n* = 50 cells from three independent experiments (all groups). ****P < 0.0001; ns, not significant, *P* = 0.9831 (1), 0.9973 (2) (ordinary one-way ANOVA Dunnett’s multiple comparison test). (**J**–**M**) Effects of LINC complex inhibition on AIS structure in mature neurons. Cortical neurons were induced to express LINC-DN by the addition of 4-OHT at 14 DIV and cultured for an additional 7 or 14 days (**J**). Representative images of neurons at 28 DIV are shown (**K**). The AIS is indicated by the two arrowheads, and its length (**L**) and position (**M**) were quantified. C, Control; DN, LINC-DN. The data represent the mean ± SEM. *n* = 51 (C, 21 DIV), *n* = 50 (DN, 21 DIV), *n* = 50 (C, 28 DIV), and *n* = 50 cells (DN, 28 DIV) (**L**, **M**) from three independent experiments. ****P < 0.0001; ns, not significant, *P* = 0.4454 (1), 0.1778 (2), 0.3621 (3) (unpaired two-tailed Welch’s *t* test for (**L**)). ns, not significant (unpaired two-tailed Mann–Whitney test for (**M**)). (**N**–**Q**) Effects of LINC complex inhibition on the structural plasticity of the AIS in mature neurons. Cortical neurons were induced to express LINC-DN by the addition of 4-OHT at 14 DIV and cultured for an additional 7 days, followed by treatment with 10 mM NaCl or 10 mM KCl for 48 h (**N**, **O**). The AIS is indicated by the two arrowheads, and its length (**P**) and position (**Q**) were quantified. NT no treatment. The data represent the mean ± SEM. *n* = 51 (NT, NaCl), *n* = 52 (NT, KCl), *n* = 50 (4-OHT, NaCl), and *n* = 50 cells (4-OHT, KCl) (**P**, **Q**) from three independent experiments. ****P < 0.0001; ns, not significant, *P* = 0.3741 (1), 0.8195 (2), 0.4115 (3) (unpaired two-tailed Welch’s *t* test for (**P**)). ns, not significant (unpaired two-tailed Mann–Whitney test for (**Q**)). Scale bars: 10 μm. Source data are available online for this figure.

Next, we investigated the impact of LINC complex dysfunction on the structural organization of the AIS. Although the expression of LINC-DN during neuronal maturation did not impair axon or dendrite formation in cortical neurons (Appendix Fig. S6A–E), it significantly shortened the AIS length without altering its position in cultured developing neurons, including cortical, hippocampal, and Purkinje cells (Figs. 5A–C and EV5A,B). The expression of the Nesprin-2-derived KASH domain or the deletion of Sun1, but not Sun2, also induced AIS shortening (Fig. EV5C–G). Consistent with the essential role of the interaction between the LINC complex and actin filaments in maintaining nuclear structure (Fig. 2C–E), the LINC-DN-induced AIS shortening was fully rescued by the co-expression of mini N2G SR52-56 or mini N2G SR55-56 lacking the microtubule interaction domain, whereas N2G SR52-56, unable to bind actin filaments, failed to elicit a rescue effect (Fig. EV5H,I). Moreover, LINC-DN completely disrupted the structural plasticity of the AIS in developing cortical neurons in response to the chronic depolarization induced by potassium chloride (Fig. 5D–F). We further examined whether the LINC complex is essential for maintaining the structural organization of the AIS. Using a tamoxifen-inducible Cre/loxP system, LINC-DN was expressed in cortical neurons following AIS maturation, resulting in the loss of structural plasticity and subsequent AIS shortening (Fig. EV5J–Q). Furthermore, the expression levels of AIS-associated molecules (Freal and Hoogenraad, 2025; Leterrier, 2018), including Ankyrin-G, βIV-spectrin, Nav channels, Trim46, and phosphorylated myosin light chain (pMLC), were significantly reduced at the AIS in LINC-DN-expressing cortical neurons (Fig. 5G–L; Appendix Fig. S7). The expression of LINC-DN in cortical neurons severely disrupted the 190-nm periodic actin ring structure at the AIS, which provides mechanical support to the axon and influences its electrophysiological properties (Fig. 5M–O) (Costa and Sousa, 2021; Xu et al, 2013).

**Figure EV6:**
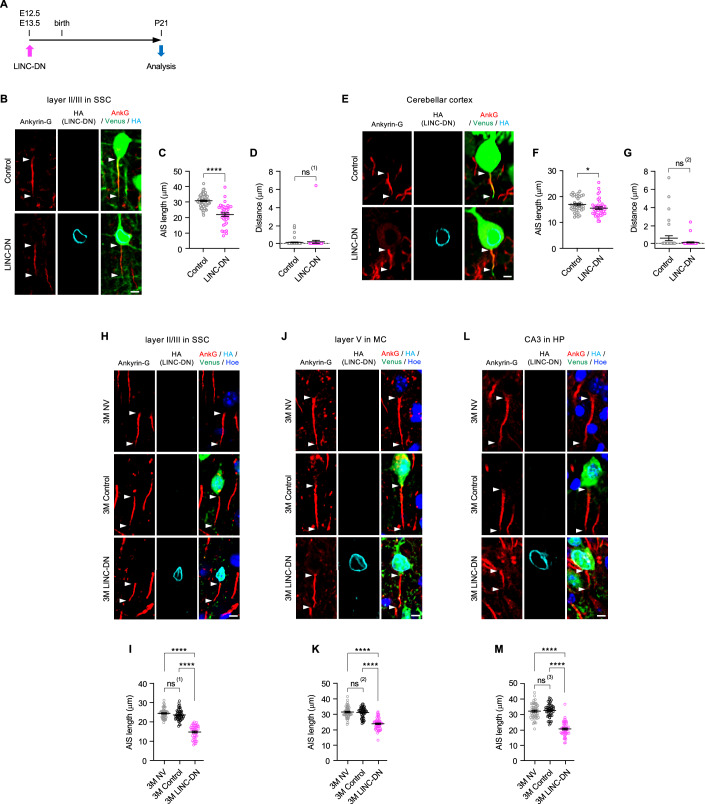
Effects of LINC complex inhibition on the AIS in vivo. (**A**–**G**) Effects of embryonic LINC-DN expression on the AIS. LINC-DN was expressed in neurons using in utero electroporation (**A**). Representative images are shown for layer II/III pyramidal neurons in the somatosensory cortex (**B**) and Purkinje cells in the cerebellum (**E**). The AIS is indicated by the two arrowheads. AIS length (**C**, **F**) and position (**D**, **G**), measured as the distance from the soma, were quantified. The data represent the mean ± SEM. *n* = 72 (Control) and *n* = 39 cells (LINC-DN) (**C**, **D**) from four brains; *n* = 37 (Control) and *n* = 42 cells (LINC-DN) (**F**, **G**) from six brains. *P = 0.0472; ****P < 0.0001 (unpaired two-tailed Welch’s *t* test for (**C**, **F**)). ns, not significant, *P* = 0.4226 (1), 0.1258 (2) (unpaired two-tailed Mann–Whitney test for (**D**, **G**)). (**H**–**M**) Effects of postnatal LINC-DN expression on the AIS. LINC-DN was expressed in neurons of 1.5-month-old mice via AAV delivery. Representative images are shown for layer II/III pyramidal neurons in the somatosensory cortex (SSC) (**H**), layer V pyramidal neurons in the motor cortex (MC) (**J**), and CA3 neurons in the hippocampus (HP) (**L**) from 3-month-old mice in no virus (3 M NV), AAV-Venus alone (3 M Control), and AAV-Venus + AAV-LINC-DN (3 M LINC-DN) groups. The AIS is indicated by the two arrowheads, and its length was quantified (**I**, **K**, **M**). The data represent the mean ± SEM. *n* = 55 (3 M NV), *n* = 53 (3 M Control), and *n* = 47 (3 M LINC-DN) (**I**); *n* = 51 (3 M NV), *n* = 50 (3 M Control), and *n* = 51 (3 M LINC-DN) (**K**); *n* = 51 (3 M NV), *n* = 50 (3 M Control), and *n* = 51 cells (3 M LINC-DN) (**M**) from three brains. ****P < 0.0001; ns, not significant, *P* = 0.3183 (1), 0.8891 (2), 0.8679 (3) (ordinary one-way ANOVA Tukey’s multiple comparison test). Scale bars: 10 μm. Source data are available online for this figure.

Then, we investigated the effects of LINC complex inhibition on the AIS in vivo. Expressing LINC-DN during embryonic development via in utero electroporation resulted in a reduction in AIS length in postnatal somatosensory cortical neurons and Purkinje cells (Fig. EV6A–G). In addition, AAV-mediated neuronal delivery of LINC-DN in 1.5-month-old mice induced AIS shortening across various neuronal types, including pyramidal neurons in the prefrontal, somatosensory, and motor cortices, as well as hippocampal CA3 neurons, at 3 months (Figs. 5P,Q and EV6H–M; Appendix Fig. S4F).

As structural changes in the AIS are essential for physiological adaptation to fluctuations in neural activity levels (Freal and Hoogenraad, 2025; Leterrier, 2018; Yamada and Kuba, 2016), the LINC complex may be essential in AIS function in developing and mature neurons.

### LINC complex inhibition leads to reduced neuronal excitability and altered brain function

Next, we investigated how LINC complex inhibition affects AIS function, which is key for regulating neuronal excitability (Freal and Hoogenraad, 2025; Huang and Rasband, 2018; Leterrier, 2018) and cell polarity (Eichel and Shen, 2022; Freal and Hoogenraad, 2025). Transferrin receptors (TfRs) are selectively directed to dendrites because the AIS restricts their entry into the axon (Burack et al, 2000). This selective transport of TfRs was preserved in LINC-DN-expressing neurons, suggesting that the LINC complex is not required for the “gatekeeper” function of the AIS (Appendix Fig. S6F,G).

**Figure 6:**
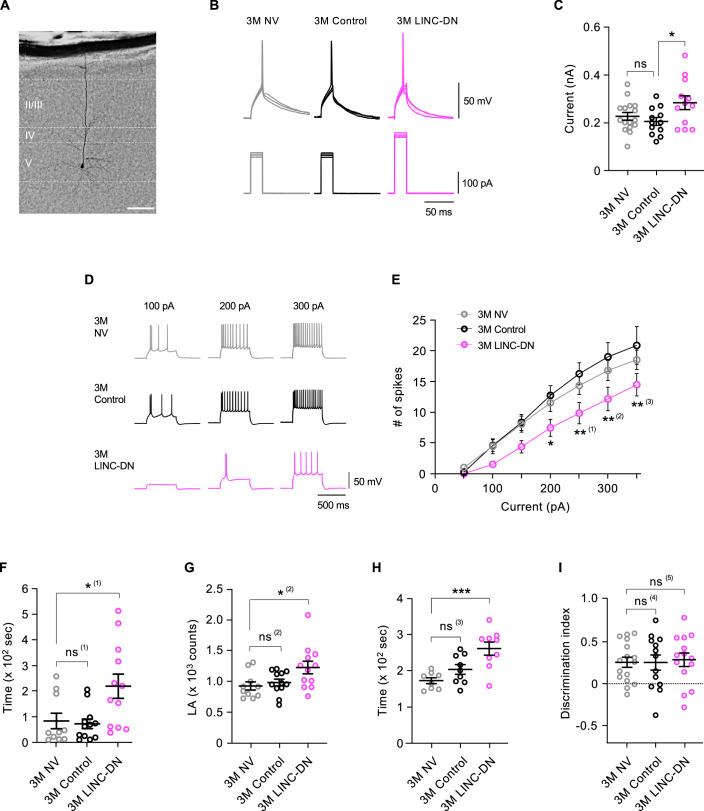
Dysfunction of the LINC complex alters neuronal excitability and brain function in young mice. (**A**–**E**) Neural activity recordings in pyramidal neurons. A representative image of a biocytin-filled layer V pyramidal neuron in the prefrontal cortex of a 3-month-old mouse is shown in (**A**). The analysis was performed on 3-month-old mice in no virus (3 M NV), AAV-Venus alone (3 M Control), and AAV-Venus + AAV-LINC-DN (3 M LINC-DN) groups. Representative recordings in layer V pyramidal neurons of the prefrontal cortex are shown, depicting responses to a 20-ms current injection (**B**) and spiking activity during 500-ms current injections at 100, 200, and 300 pA (**D**). Current threshold (**C**) and firing frequency (**E**) were quantified. The data represent the mean ± SEM. For current threshold (**C**): *n* = 16 (3 M NV), *n* = 12 (3 M Control), and *n* = 12 cells (3 M LINC-DN) from six or seven brains. *P = 0.0266; ns, not significant, *P* = 0.6688 (ordinary one-way ANOVA Dunnett’s multiple comparison test). For firing frequency (**E**): *n* = 15 (3 M NV), *n* = 15 (3 M Control), and *n* = 13 cells (3 M LINC-DN) from six or seven brains. *P = 0.0345; **P = 0.0080 (1), 0.0044 (2), 0.0082 (3) (two-way ANOVA Dunnett’s multiple comparison test *vs* 3 M Control). (**F**–**I**) Behavioral analyses. Anxiety-like behavior (**F**, **H**), locomotor activity (LA) (**G**), and memory function (**I**) of 3-month-old mice from the same groups as shown in (**B**–**E**), assessed using the open-field test (**F**, **G**), elevated plus maze test (**H**), and novel object recognition test (**I**). The data represent the mean ± SEM. *n* = 10 (3 M NV), *n* = 12 (3 M Control), and *n* = 12 (3 M LINC-DN) (**F**, **G**); *n* = 8 (3 M NV), *n* = 9 (3 M Control), and *n* = 9 (3 M LINC-DN) (**H**); *n* = 16 (3 M NV), *n* = 13 (3 M Control), and *n* = 14 mice (3 M LINC-DN) (**I**). *P = 0.0201 (1), 0.0237 (2); ***P < 0.001; ns, not significant, *P* = 0.9635 (1), 0.8364 (2), 0.2350 (3), 0.9996 (4), 0.9442 (5) (ordinary one-way ANOVA Dunnett’s multiple comparison test). Scale bar: 100 μm. Source data are available online for this figure.

Since a reduced AIS length is thought to be correlated with decreased neuronal excitability (Jamann et al, 2018; Kole and Brette, 2018), we examined the effect of LINC complex inhibition on the generation of action potentials. Whole-cell patch-clamp recordings from layer V pyramidal neurons in acute prefrontal cortex slices revealed that LINC-DN-expressing neurons exhibited an increased action potential threshold (Fig. 6A–C; Appendix Fig. S4F), without affecting the input resistance (*P* = 0.12, one-way ANOVA). Furthermore, LINC-DN-expressing neurons displayed fewer spikes induced by prolonged current injection (Fig. 6D,E). These results strongly suggest that inhibiting the LINC complex reduces neuronal excitability in cortical neurons, which correlates with AIS shortening.

We further examined how LINC complex impairment in neurons affects brain function in mice using behavioral assays. In the open-field test, LINC-DN-expressing mice exhibited altered anxiety-like behaviors associated with open spaces and increased spontaneous locomotor activity compared to the control groups (Fig. 6F,G; Appendix Fig. S4F). Anxiety-like phenotype was confirmed using the elevated plus maze test (Fig. 6H), which assesses acrophobia-related anxiety. In contrast, the novel object recognition test revealed that cognitive memory function remained intact in these mice (Fig. 6I). These results suggest that the LINC complex is essential for maintaining specific brain functions, likely through AIS-dependent regulation of neural activity. Notably, anxiety-related emotions were significantly affected by reduced neural activity regardless of their underlying causes, whereas cognitive memory was unaffected, highlighting the heterogeneity in the mechanisms generating distinct brain functions.

### Sustaining Sun1 expression during aging prevents age-related changes in AIS, neuronal excitability, and brain function

**Figure 7:**
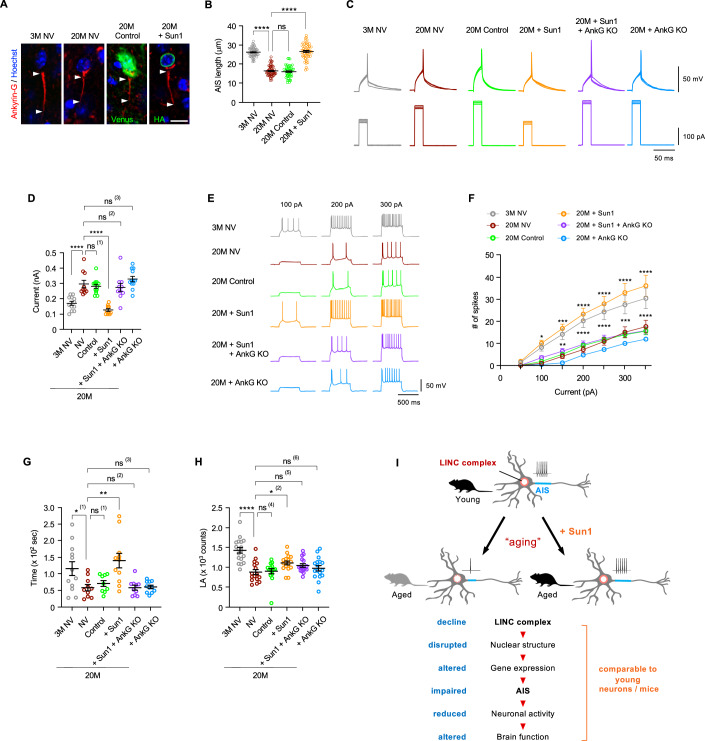
Sustained expression of Sun1 prevents age-related changes in AIS structure, neuronal excitability, and brain function. (**A**, **B**) AIS structure in young and aged neurons. Brain sections from 3- or 20-month-old mice in no virus (3 M NV or 20 M NV), AAV-Venus (20 M Control), and AAV-Sun1 (20 M + Sun1) groups were co-immunostained with antibodies against Ankyrin-G and HA (for Sun1). Representative images are shown for layer V pyramidal neurons in the prefrontal cortex (**A**). The AIS is indicated by the two arrowheads, and its length was quantified (**B**). The data represent the mean ± SEM. *n* = 60 (3 M NV), *n* = 60 (20 M NV), *n* = 53 (20 M Control), and *n* = 55 cells (20 M + Sun1) from three brains. ****P < 0.0001; ns, not significant, *P* = 0.8500 (ordinary one-way ANOVA Dunnett’s multiple comparison test). (**C**–**F**) Representative recordings in layer V pyramidal neurons of the prefrontal cortex: responses to a 20-ms current injection (**C**) and spiking activity during 500-ms current injections at 100, 200, and 300 pA (**E**). The analysis was performed on 3- or 20-month-old mice in no virus (3 M NV or 20 M NV), AAV-Venus (20 M Control), AAV-Sun1 (20 M + Sun1), AAV-Sun1 + AAV-AnkG KO (20 M + Sun1 + AnkG KO), and AAV-AnkG KO alone (20 M + AnkG KO) groups. Current threshold (**D**) and firing frequency (**F**) were quantified. The data represent the mean ± SEM. For current threshold (**D**): *n* = 12 (3 M NV), *n* = 10 (20 M NV), *n* = 14 (20 M Control), *n* = 10 (20 M + Sun1), *n* = 10 (20 M + Sun1 + AnkG KO), and *n* = 12 cells (20 M + AnkG KO) from 4 to 11 brains. ****P < 0.0001; ns, not significant, *P* = 0.9457 (1), 0.8451 (2), 0.5786 (3) (ordinary one-way ANOVA Dunnett’s multiple comparison test). For firing frequency (**F**): *n* = 13 (3 M NV), *n* = 10 (20 M NV), *n* = 14 (20 M Control), *n* = 11 (20 M + Sun1), *n* = 10 (20 M + Sun1 + AnkG KO), and *n* = 13 cells (20 M + AnkG KO) from 4 to 11 brains. *P = 0.0179; **P = 0.0042; ***P < 0.001; ****P < 0.0001 (two-way ANOVA Dunnett’s multiple comparison test *vs* 20 M NV). (**G**, **H**) Anxiety-like behavior (**G**) and locomotor activity (LA) (**H**) of 3- or 20-month-old mice from the same groups as shown in (**C**–**F**), assessed using the open-field test. The data represent the mean ± SEM. *n* = 12 (3 M NV), *n* = 10 (20 M NV), *n* = 9 (20 M Control), *n* = 11 (20 M + Sun1), *n* = 10 (20 M + Sun1 + AnkG KO), and *n* = 11 (20 M + AnkG KO) (**G**); *n* = 18 (3 M NV), *n* = 16 (20 M NV), *n* = 15 (20 M Control), *n* = 17 (20 M + Sun1), *n* = 16 (20 M + Sun1 + AnkG KO), and *n* = 17 mice (20 M + AnkG KO) (**H**). *P = 0.0289 (1), 0.0378 (2); **P = 0.0012; ****P < 0.0001; ns, not significant, *P* = 0.9677 (1), *P* > 0.9999 (2), *P* = 0.9999 (3), 0.9988 (4), 0.2151 (5), 0.6939 (6) (ordinary one-way ANOVA Dunnett’s multiple comparison test). (**I**) A schematic of the LINC complex-mediated mechanism underlying normal neuronal aging, as revealed in this study. Scale bar: 10 μm. Source data are available online for this figure.

**Figure EV7:**
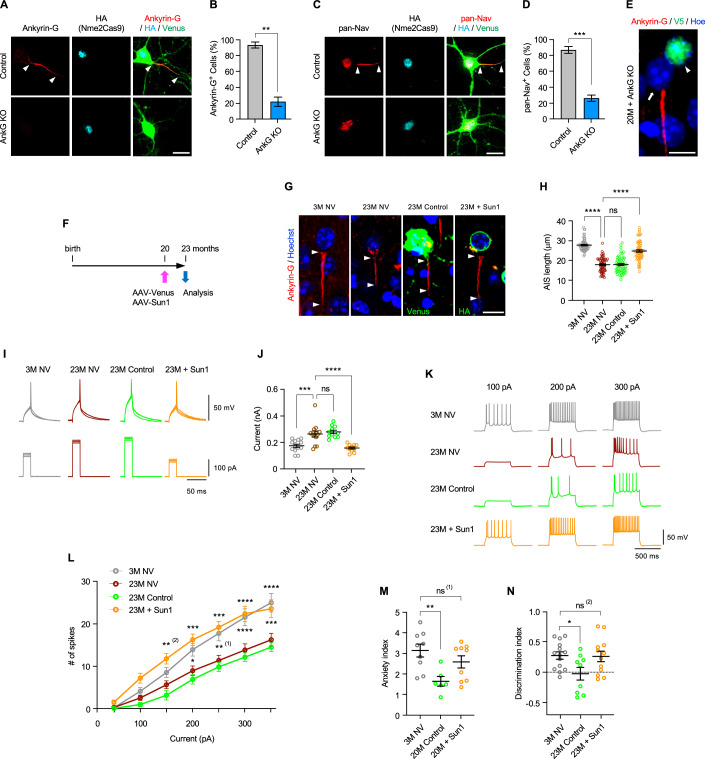
Analysis of AIS length, neuronal excitability, and brain function in aged mice. (**A**–**D**) Analysis of Ankyrin-G deletion in neurons in vitro. Control and Ankyrin-G knockout (AnkG KO) cortical neurons at 21 DIV (**A**, **C**). The AIS is indicated by the two arrowheads. Ankyrin-G-positive cells (**B**) and cells exhibiting AIS-localized pan-Nav signal (**D**) were quantified. The data represent the mean ± SEM. *n* = 3 independent experiments, eight–fifteen cells per experiment (**B**, **D**). **P = 0.0011; ***P < 0.001 (unpaired two-tailed Welch’s *t* test). (**E**) Analysis of Ankyrin-G deletion in neurons in vivo. Representative image of layer V pyramidal neurons in the prefrontal cortex of 20-month-old mice administered AAV-AnkG KO (20 M + AnkG KO). The arrowhead and arrow indicate Nme2Cas9-positive and -negative neurons, respectively. Note that the Ankyrin-G signal is specifically lost in Nme2Cas9-positive neurons. The efficiency of Ankyrin-G deletion is 81.6% ± 0.2 (*n* = 3) and 82.0% ± 1.1 (*n* = 3) in the prefrontal and somatosensory cortices, respectively. (**F**) Schematic of the AAV experiment to investigate the effects of Sun1 introduction in aged neurons. 20-month-old mice were infected with AAV-Venus (23 M Control) or AAV-Sun1 (23 M + Sun1), and analyzed at 23 months of age. (**G**, **H**) Analysis of AIS structure in 3- or 23-month-old mice in no virus (3 M NV or 23 M NV), AAV-Venus (23 M Control), and AAV-Sun1 (23 M + Sun1) groups. Brain sections from the indicated groups were co-immunostained with antibodies against Ankyrin-G and HA (for Sun1). Representative images are shown for layer V pyramidal neurons in the prefrontal cortex (**G**). The AIS is indicated by the two arrowheads, and its length was quantified (**H**). The data represent the mean ± SEM. *n* = 60 (3 M NV), *n* = 60 (23 M NV), *n* = 58 (23 M Control), and n = 60 cells (23 M + Sun1) from three brains. ****P < 0.0001; ns, not significant, *P* = 0.9996 (ordinary one-way ANOVA Dunnett’s multiple comparison test). (**I**–**L**) Representative recordings in layer V pyramidal neurons in the prefrontal cortex of 3- or 23-month-old mice from the same groups as shown in (**G**, **H**): responses to a 20-ms current injection (**I**) and spiking activity during 500-ms current injections at 100, 200, and 300 pA (**K**). Current threshold (**J**) and firing frequency (**L**) were quantified. The data represent the mean ± SEM. For current threshold (**J**): *n* = 13 (3 M NV), *n* = 13 (23 M NV), *n* = 11 (23 M Control), and n = 11 cells (23 M + Sun1) from 6 to 11 brains. ***P < 0.001; ****P < 0.0001; ns, not significant, *P* = 0.8224 (ordinary one-way ANOVA Dunnett’s multiple comparison test). For firing frequency (**L**): *n* = 17 (3 M NV), *n* = 13 (23 M NV), *n* = 11 (23 M Control), and *n* = 11 cells (23 M + Sun1) from 6 to 11 brains. *P = 0.0167; **P = 0.0012 (1), 0.0063 (2); ***P < 0.001; ****P < 0.0001 (two-way ANOVA Dunnett’s multiple comparison test *vs* 23 M NV). (**M**, **N**) Behavioral analyses. The elevated plus maze test was performed on 3- or 20-month-old mice in no virus (3 M NV), AAV-Venus (20 M Control), and AAV-Sun1 (20 M + Sun1) groups (**M**). Novel object recognition test was performed on 3- or 23-month-old mice in no virus (3 M NV), AAV-Venus (23 M Control), and AAV-Sun1 (23 M + Sun1) groups (**N**). The data represent the mean ± SEM. *n* = 9 (3 M NV), *n* = 6 (20 M Control), and *n* = 9 (20 M + Sun1) (**M**); *n* = 14 (3 M NV), *n* = 9 (23 M Control), and *n* = 12 mice (23 M + Sun1) (**N**). *P = 0.0308; **P = 0.0056; ns, not significant, *P* = 0.2995 (1), 0.9890 (2) (ordinary one-way ANOVA Dunnett’s multiple comparison test). Scale bars: 10 μm. Source data are available online for this figure.

Finally, we investigated the effect of increasing Sun1 levels on the AIS structure, neuronal excitability, and brain function in aged neurons. In the pyramidal neurons of the prefrontal cortex of 20 M+Sun1 mice, AIS length was fully restored to the levels of 3-month-old mice neurons (Figs. 7A,B and EV2A). Remarkably, the action potential threshold and spike firing frequency in these aged neurons were restored to the levels of 3-month-old mice neurons (Fig. 7C–F). This effect of Sun1 introduction on neuronal excitability was abolished by disrupting the AIS by deleting Ankyrin-G (Figs. 7C–F and EV7A–E; Appendix Table S1C) (Barry et al, 2014; Freal and Hoogenraad, 2025). Moreover, cortical neurons of mice that were administered AAV-Sun1 at 20 months of age also showed the restoration of AIS length and neuronal excitability at 23 months (Fig. EV7F–L). These findings strongly suggest that age-related changes in AIS structure and neuronal excitability are preventable and reversible. Given that exogenous Sun1 and the upregulated endogenous Nesprin-1 and Nesprin-2 were localized at the Golgi apparatus as well as the NE in the cortical neurons of 20 M+Sun1 mice (Fig. EV2H–J), resembling the misaccumulation of endogenous LINC complex molecules in control-aged mice (Appendix Fig. S2A), the AIS shortening and reduced neuronal excitability in aged neurons were attributed to the significant reduction of the LINC complex on the NE, rather than its misaccumulation in the Golgi apparatus.

We further examined whether the characteristic age-related behavioral changes in mice (Li et al, 2020), such as those associated with anxiety, spontaneous motor activity, and memory, could be mitigated by inducing the expression of Sun1 in aged neurons. The age-induced increase in anxiety-like behaviors and decrease in locomotor activity were significantly suppressed in 20 M+Sun1 mice, and this effect was reversed by AIS disruption (Figs. 7G,H, EV2A, and EV7M), suggesting that these changes depend on AIS regulation. Moreover, the decline in memory function with aging was substantially attenuated by Sun1 introduction (Fig. EV7F,N). These findings suggest that the age-related decline in LINC complex expression in neurons contributes to changes in emotion, locomotor activity, and memory in mice. In young and aged mice, memory function exhibits differential responses to LINC complex integrity and the neural activity it governs (Figs. 6I and EV7N), suggesting the involvement of additional factors whose effects vary with age in the regulation of this process.

Taken together, these results suggest that the LINC complex is crucial for regulating neuronal excitability through the AIS, and that a reduction in the expression levels of its key elements may contribute to AIS dysfunction, diminished neural activity, and changes in brain function during aging.

## Discussion

This study demonstrates that AIS dysfunction, driven by the age-related decline in LINC complex expression, is a key contributor to normal neuronal aging (Fig. 7I). The age-associated reduction of the LINC complex components, particularly Sun1, causes nuclear structural abnormalities, leading to chromatin remodeling and global gene expression changes in aged neurons—especially affecting molecules essential for AIS function, such as Nav and Kv channels. This altered expression of AIS molecules likely reduces neuronal excitability and contributes to age-related changes in brain function. Remarkably, introducing only Sun1 significantly prevented AIS dysfunction and the resulting neuronal aging, offering novel insights into brain aging, a process traditionally attributed to the accumulation of various pro-aging factors (Bishop et al, 2010; Jin and Cai, 2023).

The LINC complex regulates the nuclear structure, and abnormalities in this structure may contribute to cellular aging in non-neuronal cells (Meqbel et al, 2022). However, its neuronal role remained elusive. The involvement of Sun1 in cellular aging has been demonstrated in fibroblasts derived from patients with HGPS and mouse models of HGPS (Chang et al, 2019; Chen et al, 2012), although the mechanisms that drive aging may differ from those in neurons. In fibroblasts from HGPS mouse models, abnormally accumulated Sun1 in the Golgi apparatus accelerates cellular aging, without notable changes in mRNA expression of Sun1 (Chen et al, 2012). In contrast, Sun1 mRNA levels and its protein expression on the NE significantly decrease in aged neurons (Fig. 1A,B,I,J,Q; Appendix Fig. S1B,C,J,K). Similar to HGPS cells, Sun1 also accumulates in the Golgi apparatus of aged neurons (Appendix Fig. S2A). However, despite its localization in the Golgi apparatus and NE, exogenously introduced Sun1 restores nuclear integrity, AIS structure, and neuronal excitability in aged neurons to levels comparable to those of young neurons (Figs. 2L,M, 7A–F, and EV2H). These findings suggest that Sun1 accumulation in the Golgi apparatus during aging is not a primary cause of neuronal dysfunction. In addition, Sun1 expression is increased in aged muscle cells and fibroblasts (Chang et al, 2019; Mattioli et al, 2011), indicating that the age-related regulation of Sun1 expression and its role in cellular aging may differ across cell types. Although the mechanism by which Golgi-localized Sun1 promotes cellular aging in HGPS cells remains poorly understood, it is intriguing that Sun1 abnormalities induce nuclear structural defects and chromatin alterations in aged neurons and HGPS cells (Chen et al, 2012). This suggests that regardless of the underlying mechanisms, Sun1 dysfunction ultimately converges on the disruption of nuclear integrity, leading to cellular aging. It is noteworthy that the pronounced restoration of Lamin B1, which directly associates with the genome, following Sun1 introduction in aged neurons, suggests that the resulting change in Lamin B1 level may impact chromatin dynamics. Future studies should aim to uncover the mechanisms that contribute to the age-related decline in Sun1 expression in neurons, which may provide insights into crucial processes that drive neuronal aging.

All Nav channels that are essential for action potential generation at the AIS (Yamada and Kuba, 2016)—including Nav1.1, Nav1.2, and Nav1.6—showed a significant decrease in expression in cortical neurons with aging, whereas the expression of Kv channels, such as Kv1.2 and Kv7.3, increased (Fig. 3J). The introduction of Sun1 in aged neurons significantly restored the changes in the expression of these ion channels (Fig. 3J). These findings strongly suggest that age-related changes in the expression of Nav and Kv channels at the AIS are the most compelling mechanism underlying the decline in neuronal excitability and accompanying AIS shortening in aged neurons. Similar to its effects on Nav1 expression, aging leads to a decrease in the expression of NF186, an AIS-localized cell adhesion molecule, which can be restored by Sun1 introduction (Fig. 3J). The reduced expression of Nav1 and NF186 impedes the assembly of Ankyrin-G (Alpizar et al, 2019; Xu and Shrager, 2005), the master organizer of the AIS (Freal and Hoogenraad, 2025). Therefore, the age-related downregulation of specific AIS molecules may sequentially cause abnormalities in the proper localization of other AIS-related molecules (Fig. 5G–L; Appendix Fig. S7) and specialized structures, such as the actin ring (Fig. 5M–O).

The global shift in chromatin structure and gene expression toward a young neuron pattern in 20 M+Sun1 mice neurons suggests that various cellular mechanisms induce changes in brain function in these mice. For example, mitochondrial abnormalities are considered key contributors to brain aging (Bishop et al, 2010; Jin and Cai, 2023). Consistent with this, many GO terms that exhibit expression changes upon Sun1 introduction in aged neurons are strongly associated with mitochondrial regulation (Fig. EV4B). Nevertheless, brain function analyses, such as the anxiety-like behavior test, revealed that the effect of Sun1 introduction in aged neurons was abolished after the disruption of AIS function (Fig. 7G,H). This finding underscores the essential role of AIS-mediated neural activity regulation in the age-related changes of brain function, at least with regard to specific aspects. In neuronal aging, wherein the expression of LINC complex molecules drastically decreases, direct changes in gene expression related to nuclear structural defects and the subsequent reduction in neural activity may secondarily affect gene expression (Yap and Greenberg, 2018). Together, these factors could diminish the resistance to various pro-aging processes, underscoring the importance of preserving LINC complex activity to prevent the initiation of aging and thereby maintain brain function.

## Methods

**Table:** Reagents and tools table

Reagent/resource	Reference or source	Identifier or catalog number
**Experimental models**		
C57BL/6J mouse	Japan SLC	Cat. #: C57BL/6JmsSlc
ICR mouse	Japan SLC	Cat. #: Slc:ICR
NIH3T3	JCRB Cell Bank	Cat. #: JCRB0615
HEK293T	GenHunter	Cat. #: Q401
**Recombinant DNA**		
pENN.AAV.hSyn.HI.eGFP-Cre.WPRE.SV40	Addgene	Addgene plasmid: #105540
pUCmini-iCAP-PHP.eB	Chan et al, 2017	Addgene plasmid: #103005
pCAG-ERT2CreERT2	Matsuda and Cepko, 2007	Addgene plasmid: #13777
Nme2Cas9_AAV	Edraki et al, 2019	Addgene plasmid: #119924
pHelper	Agilent Technologies	N/A
pCAGIG-mini N2G SR52-56-HA	Goncalves et al, 2020	N/A
pCAGIG-mini N2G SR55-56-HA	Goncalves et al, 2020	N/A
pCAGIG-N2G SR52-56-HA	Goncalves et al, 2020	N/A
pCAG-neo-3xHA-Syne1-KASH	This paper	N/A
pCAG-neo-3xHA-Syne2-KASH	This paper	N/A
pCAG-neo	FUJIFILM Wako	Cat. #: 163-25601
pCAG-Venus	Hasegawa et al, 2022	N/A
pCALNL-3xHA-Syne1-KASH	This paper	N/A
pENN.AAV.hSyn.HI.Venus.WPRE.SV40	This paper	N/A
pENN.AAV.hSyn.HI.3xHA-Syne1-KASH.WPRE.SV40	This paper	N/A
pENN.AAV.hSyn.HI.Sun1-3xHA.WPRE.SV40	This paper	N/A
pCAG-neo-TfR-mCherry	This paper	N/A
Nme2Cas9-AAV-hSyn-gRNA(-)-3xHA	This paper	N/A
Nme2Cas9-AAV-hSyn-Sun1 KO-3xHA	This paper	N/A
Nme2Cas9-AAV-hSyn-Sun2 KO-3xHA	This paper	N/A
Nme2Cas9-AAV-hSyn-AnkG KO-3xHA	This paper	N/A
Nme2Cas9-AAV-hSyn-AnkG KO-V5	This paper	N/A
**Antibodies**		
Rabbit anti-Sun1	Chi et al, 2009	N/A
Guinea pig anti-Sun1	Gob et al, 2010	N/A
Guinea pig anti-Sun2	Link et al, 2014	N/A
Rabbit anti-Sun2	Zhang et al, 2007	N/A
Mouse anti-Sun2	Millipore	Cat. #: MABT880 RRID: N/A
Rabbit anti-Nesprin-1	Razafsky et al, 2013	N/A
Mouse anti-Nesprin-1	DSHB	Cat. #: MANNES1E RRID: AB_2753303
Rabbit anti-Nesprin-2	ImmunoQuest	Cat. #: IQ565 RRID: N/A
Rabbit anti-Lamin B1	HistoSure	Cat. #: HS-404 003 RRID: AB_3677699
Rabbit anti-Histone H3 (tri methyl K9)	Abcam	Cat. #: ab8898 RRID: AB_306848
Mouse anti-Ankyrin-G	NeuroMab	Cat. #: 75-146 RRID: AB_10673030
Rabbit anti-Nav1.2	Alomone Labs	Cat. #: ASC-002 RRID: AB_2040005
Rabbit anti-Nav1.6	Alomone Labs	Cat. #: ASC-009 RRID: AB_2040202
Rabbit anti-Kv7.3	Alomone Labs	Cat. #: APC-051 RRID: AB_2040103
Mouse anti-pan Nav	Rasband et al, 1999	N/A
Rabbit anti-βIV-spectrin	Yang et al, 2004	N/A
Rabbit anti-Trim46	Synaptic Systems	Cat. #: 377 003 RRID: AB_2631232
Rabbit anti-phospho-myosin light chain 2	Cell Signaling Technology	Cat. #: 3674 RRID: AB_2147464
Mouse anti-CaMKIIα	Santa Cruz Biotechnology	Cat. #: sc-13141 RRID: AB_626789
Mouse anti-GAD65	Millipore	Cat. #: MAB351 RRID: AB_11214081
Mouse anti-S100β	Sigma-Aldrich	Cat. #: S2532 RRID: AB_477499
Mouse anti-APC	Sigma-Aldrich	Cat. #: OP80 RRID: AB_2057371
Mouse anti-Sox2	Santa Cruz Biotechnology	Cat. #: sc-365823 RRID: AB_10842165
Mouse anti-NeuN	Chemicon	Cat. #: MAB377 RRID: AB_2298772
Guinea pig anti-MAP2	Synaptic Systems	Cat. #: 188 004 RRID: AB_2138181
Mouse anti-GM130	BD Biosciences	Cat. #: 610822 RRID: AB_398141
Chick anti-GFP	Aves Labs	Cat. #: GFP-1010 RRID: AB_2307313
Rabbit anti-RFP	MBL	Cat. #: PM005 RRID: AB_591279
Rat anti-HA	Roche	Cat. #: 11867423001 RRID: AB_390918
Rabbit anti-V5	Abcam	Cat. #: ab9116 RRID: AB_307024
Alexa Fluor 594-goat anti-rabbit IgG	Jackson ImmunoResearch	Cat. #: 111-586-144 RRID: AB_2338070
Alexa Fluor 594-donkey anti-rabbit IgG	Jackson ImmunoResearch	Cat. #: 711-585-152 RRID: AB_2340621
Alexa Fluor 594-goat anti-mouse IgG	Jackson ImmunoResearch	Cat. #: 115-586-146 RRID: AB_2338899
Alexa Fluor 594-donkey anti-mouse IgG	Jackson ImmunoResearch	Cat. #: 715-585-151 RRID: AB_2340855
Alexa Fluor 488-goat anti-rabbit IgG	Jackson ImmunoResearch	Cat. #: 111-546-144 RRID: AB_2338057
Alexa Fluor 488-goat anti-mouse IgG	Jackson ImmunoResearch	Cat. #: 115-546-146 RRID: AB_2338868
Alexa Fluor 488-goat anti-rat IgG	Jackson ImmunoResearch	Cat. #: 112-546-143 RRID: AB_2338370
Alexa Fluor 488-donkey anti-guinea pig IgG	Jackson ImmunoResearch	Cat. #: 706-546-148 RRID: AB_2340473
Alexa Fluor 488-donkey anti-chick IgY	Jackson ImmunoResearch	Cat. #: 703-545-155 RRID: AB_2340375
Alexa Fluor 647-donkey anti-mouse IgG	Jackson ImmunoResearch	Cat. #: 715-605-151 RRID: AB_2340863
Alexa Fluor 647-donkey anti-rat IgG	Jackson ImmunoResearch	Cat. #: 712-605-153 RRID: AB_2340694
**Oligonucleotides and other sequence-based reagents**		
Guide RNA sequence for *Ank3*: TGCGATCCCGGGACCGTTTGCGGT	This paper	N/A
Guide RNA sequence for *Sun1*: TTTGGTCTGCTCACGAATCA	This paper	N/A
Guide RNA sequence for *Sun2*: TCTCAGGATGATAACGATGG	This paper	N/A
Primers of AAV titer check for WPRE sequence: 5′-CTGTTGGGCACTGACAATTC-3′ and 5′-GAAGGGACGTAGCAGAAGGA-3′	This paper	N/A
Primers of AAV titer check for Nme2Cas9 sequence: 5′-GTGTTTGAGAGGGCCGAGGTGC -3′ and 5′-AGCACGCCCTCTCTCTTCAGCA-3′	This paper	N/A
Primers for qPCR	Appendix Table S1	N/A
**Chemicals, enzymes, and other reagents**		
Papain	Worthington Biochemical Corporation	Cat. #: LS003127
EDTA	FUJIFILM Wako	Cat. #: 345-01865
Opti-MEM	Gibco	Cat. #: 31985070
Neurobasal	Gibco	Cat. #: 21103049
B-27	Gibco	Cat. #: 17504044
Glutamate	NACALAI TESQUE	Cat. #: 16948-04
Penicillin-Streptomycin Solution (x100)	FUJIFILM Wako	Cat. #: 168-23191
Poly-D-lysine	Sigma-Aldrich	Cat. #: P0899
4-hydroxytamoxifen	Sigma-Aldrich	Cat. #: H6278
DMEM/F12	Gibco	Cat. #: 11320033
HEPES	Sigma-Aldrich	Cat. #: H4034
D-(+)-Glucose	NACALAI TESQUE	Cat. #: 16806-25
Insulin	Sigma-Aldrich	Cat. #: I0516
Progesterone	Sigma-Aldrich	Cat. #: P6149
Putrescine dihydrochloride	Sigma-Aldrich	Cat. #: P7505
Sodium selenite	Sigma-Aldrich	Cat. #: S9133
Transferrin	Sigma-Aldrich	Cat. #: T8158
3-iodo-L-tyrosine	Sigma-Aldrich	Cat. #: I8250
Glutamax	Gibco	Cat. #: 35050061
Bovine serum albumin	NACALAI TESQUE	Cat. #: 01860-94
Cytosine β-D-arabinofuranoside	Sigma-Aldrich	Cat. #: C1768
BLOCKING REAGENT POWDER for TNB blocking buffer	Akoya Biosciences	Cat. #: FP1012
Hoechst 33342	Dojindo	Cat. #: 346-07951
D-MEM (High Glucose) with L-Glutamine, Phenol Red and Sodium Pyruvate	FUJIFILM Wako	Cat. #: 043-30085
PEI MAX	Polysciences	Cat. #: 24765-100
Isoflurane	Pfizer	N/A
Fast Green	Sigma-Aldrich	Cat. #: F7252
STAR RED, phalloidin	Abbeior	Cat. #: STRED-0100
ProLong Diamond Antifade Mountant	Invitrogen	Cat. #: P36970
Biocytin	Sigma-Aldrich	Cat. #: B4261
Percoll	Cytiva	Cat. #: 17-0891-01
cOmplete Mini, EDTA-free	Roche	Cat. #: 11836170001
Nonidet(R) P40 Substitute	NACALAI TESQUE	Cat. #: 18558-54
SPRIselect beads	Beckman Coulter	Cat. #: B23317
AMPure XP beads	Beckman Coulter	Cat. #: A63880
**Software**		
Cas-OFFinder	Bae et al, 2014	RRID: SCR_023390
Prism 9.5.1	GraphPad Software	RRID: SCR_005375
BZ-H4M image analysis software	Keyence	N/A
PenTablet software	XP-PEN Technology	N/A
ImageJ 1.53 software	NIH	RRID: SCR_003070
a custom MATLAB/Octave script for AIS analysis	Berger et al, 2018	N/A
a custom MATLAB/Octave script for nuclear analysis	This paper	N/A
iMSPECTOR LiGHTBOX	Abberior	N/A
Huygens software	Scientific Volume Imaging	RRID: SCR_014237
Neurolucida 360	MBF Bioscience	RRID: SCR_016788
Neurolucida Explorer	MBF Bioscience	RRID: SCR_017348
Chart v7	ADInstruments	N/A
SCANET MV40	MELQUEST	N/A
ANY-maze	Stoelting	RRID: SCR_014289
GENECODE vM36	Mudge et al, 2025	RRID: SCR_014966
SILVA database	Glöckner, 2019	RRID: SCR_006423
Cutadapt v4.4	Martin, 2011	RRID: SCR_011841
Bowtie2 v2.5.4	Langmead et al, 2019	RRID: SCR_016368
STAR v2.7.11b	Dobin et al, 2013	RRID: SCR_004463
SAMtools v1.21	Li et al, 2009	RRID: SCR_002105
RSEM v1.3.1	Li and Dewey, 2011	RRID: SCR_000262
DESeq2 v1.46.0	Love et al, 2014	RRID: SCR_015687
ShinyGO v0.82	Ge et al, 2020	RRID: SCR_019213
SRplot	Tang et al, 2023	RRID: SCR_025904
HiCUP v0.9.2	Wingett et al, 2015	RRID: SCR_005569
Pairix v0.3.7	Lee et al, 2022	N/A
Cooler v0.10.3	Abdennur and Mirny, 2020	RRID: SCR_024194
HiCExplorer v3.7.6	Ramirez et al, 2018	RRID: SCR_022111
hicNormalize module	Ramirez et al, 2018	N/A
hicCorrectMatrix	Ramirez et al, 2018	N/A
Knight-Ruiz (KR) matrix balancing algorithm	Ramirez et al, 2018	N/A
hicPlotMatrix	Ramirez et al, 2018	N/A
hicPlotDistVsCounts	Ramirez et al, 2018	N/A
Numpy	Harris et al, 2020	RRID: SCR_008633
hicPCA	Ramirez et al, 2018	N/A
UCSC Genome Browser	Perez et al, 2025	RRID: SCR_005780
**Other**		
VECTASTAIN Elite ABC-HRP Kit	Vector Laboratories	Cat. #: PK-6100
Thunderbird SYBR qPCR Mix	TOYOBO	Cat. #: QPS-201
Arima-HiC Kit	Arima Genomics	Cat. #: A510008
SMART-Seq mRNA Kit	Takara Bio	Cat. #: 634772
Nextera XT DNA Library Preparation Kit	Illumina	Cat. #: FC-131-1024
AAV-Venus	This paper	N/A
AAV-LINC-DN	This paper	N/A
AAV-Sun1-3xHA	This paper	N/A
AAV-eGFP-Cre	This paper	N/A
AAV-AnkG KO	This paper	N/A

### Animals

All animal care and experimental procedures were conducted in accordance with institutional guidelines and approved by the Experimental Animal Care Committee of Shimane University School of Medicine (approval numbers: IZ3-113, IZ4-2, IZ6-4, and IZ7-1). C57BL/6J and ICR mice (Japan SLC) were maintained under a 12:12 light–dark cycle, with food and water available ad libitum, and bred in the animal facility of Shimane University School of Medicine. The day on which a vaginal plug was confirmed was designated as embryonic day 0 (E0), and the day of birth was designated as postnatal day 0 (P0). Male mice were used for the in vivo analyses.

### Plasmids

The following plasmids were purchased: pENN.AAV.hSyn.HI.eGFP-Cre.WPRE.SV40 (Addgene plasmid #105540), pUCmini-iCAP-PHP.eB (Chan et al, 2017) (Addgene plasmid #103005), pCAG-ERT2CreERT2 (Matsuda and Cepko, 2007) (Addgene plasmid #13777), pCALNL-GFP (Matsuda and Cepko, 2007) (Addgene plasmid #13770), Nme2Cas9_AAV(Edraki et al, 2019) (Addgene plasmid #119924), and pHelper (Agilent Technologies). The following plasmids were kindly gifted by Dr. R.B. Vallee: pCAGIG-mini N2G SR52-56-HA (Goncalves et al, 2020), pCAGIG-mini N2G SR55-56-HA(Goncalves et al, 2020), and pCAGIG-N2G SR52-56-HA (Goncalves et al, 2020). The open reading frames (ORFs) of mouse *Syne1* and *Syne2*, which encode the C-terminal 69 amino acids of Nesprin-1 (LINC-DN) and 66 amino acids of Nesprin-2, respectively, were amplified by polymerase chain reaction (PCR) from a cDNA library derived from the P12 mouse brain for *Syne1* and from pGFP-mini-Nesprin2G (Luxton et al, 2010) (a gift from Dr. G.G. Gundersen) for *Syne2*, and subsequently cloned into the pCAG-neo vector (FUJIFILM Wako). The hemagglutinin (HA) tag was inserted at the 5′-end of the *Syne1* and *Syne2* ORFs. To construct the pCALNL-3xHA-Syne1-KASH, GFP was excised from pCALNL-GFP by digestion at the EcoRI and NotI sites, and the PCR-amplified 3xHA-Syne1-KASH was cloned into these sites. To construct the AAV vectors expressing Venus, 3xHA-Syne1-KASH, or Sun1-3xHA, eGFP-Cre was excised from pENN.AAV.hSyn.HI.eGFP-Cre.WPRE.SV40 by digestion at the NcoI and HindIII sites, and the PCR-amplified Venus, 3xHA-Syne1-KASH, or Sun1-3xHA (3xHA sequence was tagged at the 3′-end of the *Sun1* ORF) was cloned into these sites. To construct pCAG-neo-TfR-mCherry, Arp3 was excised from pCAG-neo-Arp3-mCherry (Hasegawa et al, 2022) by digestion at the KpnI and EcoRI sites, and the PCR-amplified ORF of mouse TfR was cloned into these sites. For the Nme2Cas9-based AAV vectors expressing target guide RNAs specifically in neurons, the U1a promoter was excised from Nme2Cas9_AAV by digestion at the SalI and NcoI sites, and the PCR-amplified human synapsin1 (hSyn) promoter from pENN.AAV.hSyn.HI.eGFP-Cre.WPRE.SV40 was cloned into these sites to generate the Nme2Cas9-hSyn plasmid. The guide RNA sequences (TGCGATCCCGGGACCGTTTGCGGT for *Ank3* encoding Ankyrin-G, TTTGGTCTGCTCACGAATCA for *Sun1* encoding Sun1, and TCTCAGGATGATAACGATGG for *Sun2* encoding Sun2) were cloned into Nme2Cas9-hSyn using the KOD -Plus- Mutagenesis Kit (TOYOBO). The HA tag on Nme2Cas9 in the Ankyrin-G knockout (KO) vector was substituted with a V5 tag using the KOD -Plus- Mutagenesis Kit to generate the Nme2Cas9-AAV-hSyn-AnkG KO-V5 plasmid. To assess potential off-target effects, the top ten predicted off-target sites, identified using the Cas-OFFinder (Bae et al, 2014) (CRISPR RGEN Tools, http://www.rgenome.net/cas-offinder), were sequenced in transfected NIH3T3 cells for Sun1 and Sun2 deletions or in AAV-infected mouse brains for Ankyrin-G deletion; no off-target activity was detected (Appendix Table S1).

### Cell culture

#### Primary culture of cortical and hippocampal neurons

Cerebral cortices and hippocampi were dissected from E18 ICR mouse embryos in ice-cold Ca²⁺/Mg²⁺-free Hanks’ Balanced Salt Solution (HBSS) and dissociated with 0.478 mg/mL papain (Worthington Biochemical Corporation) and 121 μM EDTA (pH 8.0) (FUJIFILM Wako). The dissociated cells were resuspended in Opti-MEM (Gibco) for electroporation. Electroporated cells were immediately resuspended in NBM/B-27 medium (Neurobasal (Gibco) containing 2% B-27 supplement (Gibco), 581 μM glutamine (NACALAI TESQUE), and 1× penicillin (100 units/mL)/streptomycin (100 μg/mL) (FUJIFILM Wako)), plated onto 14-mm round coverslips pre-coated with 1 mg/mL poly-D-lysine (PDL; Sigma-Aldrich) at a density of 5.0 × 10^5^ cells per well in a 24-well plate, and incubated at 37 °C in 5% CO_2_. Twenty-four hours after plating, the culture medium was replaced with fresh NBM/B-27, and the medium was partially refreshed every 4 days thereafter. For chronic depolarization, electroporated primary cortical neurons were treated with 10 mM NaCl or 10 mM KCl for 48 h. To induce LINC-DN expression in mature neurons, primary cortical neurons expressing pCALNL-3xHA-Syne1-KASH and pCAG-ERT2CreERT2 were treated with 20 nM 4-hydroxytamoxifen (4-OHT; Sigma-Aldrich) at 14 DIV.

#### Purkinje cell culture

Cerebella were dissected from E18 ICR mouse embryos in ice-cold Ca^2+^/Mg^2+^-free HBSS and dissociated with 0.375 mg/mL papain. The dissociated cells were resuspended in Opti-MEM for electroporation. Electroporated cells were immediately resuspended in DFB-27 medium (Dulbecco’s Modified Eagle Medium (DMEM)/F12 (Gibco) containing 1% B-27 supplement, 0.05 M HEPES (Sigma-Aldrich), 0.6% D-(+)-glucose (NACALAI TESQUE), 1.125 mg/mL NaHCO_3_ (FUJIFILM Wako), 20 μg/mL insulin (Sigma-Aldrich), 40 nM progesterone (Sigma-Aldrich), 10 μg/mL putrescine dihydrochloride (Sigma-Aldrich), 30 nM sodium selenite (Sigma-Aldrich), 100 μg/mL transferrin (Sigma-Aldrich), 10 nM 3-iodo-L-tyrosine (Sigma-Aldrich), 2× Glutamax (Gibco), 0.1 mg/mL bovine serum albumin (BSA, NACALAI TESQUE), 1× penicillin (100 units/mL)/streptomycin (100 μg/mL), and 3 μM cytosine β-D-arabinofuranoside (Sigma-Aldrich)) with 10% fetal bovine serum (FBS). Cells were plated onto 14-mm round coverslips pre-coated with 1 mg/mL PDL at a density of 1.0 × 10^6^ cells per well in a 24-well plate and incubated at 37 °C in 5% CO_2_. Three hours after plating, the culture medium was replaced with fresh DFB-27 medium, and cells were cultured for 7 days.

### Electroporation for primary neurons

A total of 0.5–1.0 × 10^6^ cells suspended in 100 μL Opti-MEM containing 10.0 μg plasmid DNA were electroporated using a NEPA21 electroporator (Nepagene). The plasmids that were used for electroporation are further described. Experiments for LINC-DN expression: 2.0 μg of pCAG-Venus and 8.0 μg of pCAG-neo (for control); 2.0 μg of pCAG-Venus and 8.0 μg of pCAG-neo-3xHA-Syne1-KASH (for LINC-DN). Experiments for inducible LINC-DN expression: 2.0 μg of pCAG-Venus, 2.0 μg pCAG-ERT2CreERT2, and 6.0 μg of pCAG-neo (for control); 2.0 μg of pCAG-Venus, 2.0 μg pCAG-ERT2CreERT2, and 6.0 μg pCALNL-3xHA-Syne1-KASH (for inducible LINC-DN). Experiments for Nesp2-KASH expression: 2.0 μg of pCAG-Venus and 8.0 μg of pCAG-neo (for control); 2.0 μg of pCAG-Venus and 8.0 μg of pCAG-neo-3xHA-Syne2-KASH (for Nesp2-KASH). Experiments for N2G mutants expression: 2.0 μg of pCAG-Venus and 8.0 μg of pCAG-neo (for control); 2.0 μg of pCAG-Venus, 4.0 μg of pCAG-neo, and 4.0 μg of pCAG-neo-3xHA-Syne1-KASH (for LINC-DN alone); 2.0 μg of pCAG-Venus, 4.0 μg of pCAG-neo or pCAG-neo-3xHA-Syne1-KASH, 4.0 μg of pCAGIG-mini N2G SR52-56-HA, pCAGIG-mini N2G SR55-56-HA, or pCAGIG-N2G SR52-56-HA (for N2G mutants with or without LINC-DN). Experiments for Sun1 and Sun2 KO: 10.0 μg of Nme2Cas9-AAV-hSyn-gRNA(-)-3xHA (for control); 10.0 μg of Nme2Cas9-AAV-hSyn-Sun1 KO-3xHA or Nme2Cas9-AAV-hSyn-Sun2 KO-3xHA (for Sun1/2 KO). Experiments for Ankyrin-G KO: 2.0 μg of pCAG-Venus and 8.0 μg of Nme2Cas9-AAV-hSyn-gRNA(-)-3xHA (for control); 2.0  μg of pCAG-Venus and 8.0 μg of Nme2Cas9-AAV-hSyn-AnkG KO-3xHA (for Ankyrin-G KO). Experiments for TfR expression: 2.0 μg of pCAG-Venus and 8.0 μg of pCAG-neo (for control); 2.0 μg of pCAG-Venus and 8.0 μg of pCAG-neo-TfR-mCherry (for TfR). Electroporation was performed using the following settings. For cortical and hippocampal neurons: poring pulse; 275 V, 0.3 ms pulse length, 50 ms interval, 2 pulses, 40% decay rate, + polarity and transfer pulse; 20 V, 50 ms pulse length, 50 ms interval, 5 pulses, 40% decay rate, ± polarity. For Purkinje cells: poring pulse; 150 V, 0.8 ms pulse length, 50 ms interval, 2 pulses, 10% decay rate, + polarity and transfer pulse; 20 V, 50 ms pulse length, 50 ms interval, 5 pulses, 40% decay rate, ± polarity.

### Immunocytochemistry (ICC)

Cultured cells were fixed for 15 min at 4 °C with 2% paraformaldehyde (PFA) in phosphate-buffered saline (PBS, pH 7.4) for immunostaining with the antibodies against pan-Nav and pMLC, or with 4% PFA for the other antibodies. The fixed cells were permeabilized with 0.3% Triton X-100 in PBS for 15 min at room temperature (RT), blocked with TNB blocking buffer (Akoya Biosciences) for 1 h at RT, incubated with primary antibodies overnight at 4 °C, and then with secondary antibodies 1.5 h at RT. Nuclear DNA was counterstained with 10 μg/mL Hoechst 33342 (Dojindo). Immunofluorescence images were acquired using a fluorescence microscope (BZ-X810, Keyence).

### Immunohistochemistry (IHC)

Immunostaining of brain sections was performed as previously described (Hasegawa et al, 2022), with some modifications. Briefly, mice were transcardially perfused with 2% (for immunostaining with the antibodies against Nav1.2, Nav1.6, and Kv7.3) or 4% (for the other antibodies) PFA in PBS at a volume twice their body weight, and dissected brains were postfixed in 2% or 4% PFA in PBS overnight (for immunostaining with the antibody against CaMKIIα) or for 2 h (for the other antibodies) at 4 °C. Sagittal sections (50–60-μm thick) were prepared using vibratomes (VT1000S and VT1200S, Leica). Floating sections were permeabilized with 0.4% (for immunostaining with the antibodies against Nav1.2, Nav1.6, and Kv7.3) or 0.3% (for the other antibodies) Triton X-100 in PBS for 15 min at RT, blocked with 2% normal goat serum (NGS) in PBS (for immunostaining with the antibody against CaMKIIα), 3% NGS in PBS containing 0.4% Triton X-100 (for immunostaining with the antibodies against Nav1.2, Nav1.6, and Kv7.3) or TNB blocking buffer (for the other antibodies) for 1 h at RT, and incubated with primary antibodies in the blocking buffer overnight at 4 °C, followed by incubation with secondary antibodies for 1.5 h at RT. For immunostaining with the guinea pig Sun2 antibody, floating sections were immersed in 10 mM citrate buffer (pH 6.0) and subjected to antigen retrieval at 95 °C for 30 min prior to permeabilization. Nuclear DNA was counterstained with 10 μg/mL Hoechst 33342. Immunofluorescence images were obtained using a BZ-X810 fluorescence microscope.

### Antibodies

The primary antibodies used for ICC and IHC are as follows: anti-Sun1 (Chi et al, 2009) (rabbit, a gift from Dr. Y.H. Chi, 1:10,000), anti-Sun1 (Gob et al, 2010) (guinea pig, a gift from Dr. M. Alsheimer, 1:2000), anti-Sun2 (Link et al, 2014) (guinea pig, a gift from Dr. M. Alsheimer, 1:600), anti-Sun2 (Zhang et al, 2007) (rabbit, a gift from Dr. M. Han, 1:600), anti-Sun2 (mouse, MABT880, Millipore, 1:600), anti-Nesprin-1 (Razafsky et al, 2013) (rabbit, a gift from Dr. D.M. Hodzic, Nes1HAA12, 1:2000), anti-Nesprin-1 (mouse, MANNES1E, DSHB, 1:50), anti-Nesprin-2 (rabbit, IQ565, ImmunoQuest, 1:2000), anti-Lamin B1 (rabbit, HS-404 003, HistoSure, 1:2000), anti-Histone H3 (tri methyl K9) (rabbit, ab8898, Abcam, 1:2000), anti-Ankyrin-G (mouse, 75-146, NeuroMab, 1:500 for ICC, 1:2000 for IHC), anti-Nav1.2 (rabbit, ASC-002, Alomone Labs, 1:1000), anti-Nav1.6 (rabbit, ASC-009, Alomone Labs, 1:1000), anti-Kv7.3 (rabbit, APC-051, Alomone Labs, 1:200), anti-pan-Nav (Rasband et al, 1999) (mouse, a gift from Dr. M.N. Rasband, 1:600), anti-βIV-spectrin (Yang et al, 2004) (rabbit, a gift from Dr. M.N. Rasband, 1:2000), anti-Trim46 (rabbit, 377 003, Synaptic Systems, 1:4000), anti-phospho-myosin light chain 2 (rabbit, 3674, Cell Signaling Technology, 1:100), anti-CaMKIIα (mouse, sc-13141, Santa Cruz Biotechnology, 1:100), anti-GAD65 (mouse, MAB351, Millipore, 1:1000), anti-S100β (mouse, S2532, Sigma-Aldrich, 1:1000), anti-APC (mouse, OP80, Sigma-Aldrich, 1:500), anti-Sox2 (mouse, sc-365823, Santa Cruz Biotechnology, 1:1000), anti-NeuN (mouse, MAB377, Chemicon, 1:1000), anti-MAP2 (guinea pig, 188 004, Synaptic Systems, 1:1000), anti-GM130 (mouse, 610822, BD Biosciences, 1:400), anti-GFP (chick, GFP-1010, Aves Labs, 1:1000), anti-RFP (rabbit, PM005, MBL, 1:1000), anti-HA (rat, 11867423001, Roche, 1:2000), and anti-V5 (rabbit, ab9116, Abcam, 1:2000). The following secondary antibodies from Jackson ImmunoResearch were used at a dilution of 1:1000: Alexa Fluor 594-goat anti-rabbit IgG (111-586-144), -donkey anti-rabbit IgG (711-585-152), -goat anti-mouse IgG (115-586-146), and -donkey anti-mouse IgG (715-585-151); Alexa Fluor 488-goat anti-rabbit IgG (111-546-144), -goat anti-mouse IgG (115-546-146), -goat anti-rat IgG (112-546-143), -donkey anti-guinea pig IgG (706-546-148), and -donkey anti-chicken IgY (703-545-155); Alexa Fluor 647-donkey anti-mouse IgG (715-605-151) and -donkey anti-rat IgG (712-605-153).

### AAV vector production

Recombinant AAV-PHP.eB vectors were produced in HEK293T cells (GenHunter) as previously described (Konno and Hirai, 2020). Briefly, HEK293T cells were cultured in DMEM (FUJIFILM Wako) supplemented with 10% FBS in 10 cm dishes. Upon reaching confluency, cells were transfected with three plasmids using the polyethylenimine method with PEI MAX (Polysciences): 6.0 μg of the expression plasmids (pENN.AAV.hSyn.HI.Venus.WPRE.SV40, pENN.AAV.hSyn.HI.3xHA-Syne1-KASH.WPRE.SV40, pENN.AAV.hSyn.HI.Sun1-3xHA.WPRE.SV40, pENN.AAV.hSyn.HI.eGFP-Cre.WPRE.SV40, or Nme2Cas9-AAV-hSyn-AnkG KO-V5), 12.0 μg of the packaging plasmid (pUCmini-iCAP-PHP.eB), and 10.0 μg of the helper plasmid (pHelper). After 24 h of incubation, the culture medium was replaced with serum-free DMEM, and AAV particles were harvested from the medium 6 days post-transfection and concentrated using Vivaspin 20 (membrane 100 K MWCO, PES, SARTORIUS). The genomic titer of the viral vectors was determined via quantitative PCR (qPCR) using THUNDERBIRD SYBR qPCR Mix (TOYOBO) with the primers specific to the WPRE sequence for AAV-Venus, AAV-3xHA-Syne1-KASH, AAV-Sun1-3xHA, and AAV-eGFP-Cre (5′-CTGTTGGGCACTGACAATTC-3′ and 5′-GAAGGGACGTAGCAGAAGGA-3′), and specific to the Nme2Cas9 sequence for AAV-AnkG KO (5′-GTGTTTGAGAGGGCCGAGGTGC-3′ and 5′-AGCACGCCCTCTCTCTTCAGCA-3′). qPCR was performed under the following conditions: an initial denaturation at 95 °C for 60 s, followed by 40 cycles of denaturation at 95 °C for 15 s, and annealing/extension at 60 °C for 30 s.

### AAV vector administration

Mice were anesthetized with 5% isoflurane (Pfizer), and 1.0–6.0 × 10^12^ viral genomes (vg) of AAV vectors suspended in 100 μL PBS were intravenously injected into the retro-orbital sinus of mice using a 0.5-mL syringe with a 30-gauge needle (Nipro). The AAV vectors and injection timelines are described further. For experiments injected at 1.5 months of age and analyzed at 3 months (Appendix Fig. S4F): 1.0 × 10^12^ vg of AAV-Venus (for 3 M Control); 1.0 × 10^12^ vg of AAV-Venus + 1.0 × 10^12^ vg of AAV-LINC-DN (for 3 M LINC-DN). For experiments injected at 12 months of age and analyzed at 20 months (Fig. EV2A): 1.0 × 10^12^ vg of AAV-Venus (for 20 M Control); 1.0 × 10^12^ vg of AAV-Sun1-3xHA (for 20 M + Sun1); 1.0  × 10^12^ vg of AAV-Sun1-3xHA + 5.0 × 10^12^ vg of AAV-AnkG KO (for 20 M + Sun1 + AnkG KO); 5.0 × 10^12^ vg of AAV-AnkG KO (for 20 M + AnkG KO). For experiments injected at 20 months of age and analyzed at 23 months (Fig. EV7F): 1.0 × 10^12^ vg of AAV-Venus (for 23 M Control); 1.0 × 10^12^ vg of AAV-Sun1-3xHA (for 23 M + Sun1). For experiments for Hi-C and RNA-seq analyses (Fig. EV3A): 1.0 × 10^12^ vg of AAV-eGFP-Cre (for 3 M, injected at 1 month of age and analyzed at 3 months; for 20 M, injected at 12 months of age and analyzed at 20 months); 1.0 × 10^12^ vg of AAV-eGFP-Cre + 1.0 ×  10^12^ vg of AAV-Sun1-3xHA (for 20 M + Sun1, injected at 12 months of age and analyzed at 20 months). The efficiency of each AAV infection was assessed 16–18 days post-injection by quantifying the percentage of cells expressing target proteins among NeuN-positive layer V pyramidal neurons in the prefrontal cortex. Ankyrin-G KO efficiency was evaluated 6 weeks post-injection by quantifying Ankyrin-G immunoreactivity in layer V pyramidal neurons in the prefrontal and somatosensory cortices that were double-positive for MAP2 (a neuronal marker) and V5, the latter indicating Nme2Cas9 expression.

### In utero electroporation

In utero electroporation was performed as previously described (Hasegawa et al, 2022; Kuwako et al, 2014; Kuwako and Okano, 2018), with some modifications. Briefly, pregnant ICR mice at E12.5 (for Purkinje cells) or E13.5 (for cortical neurons) were anesthetized with 1.5% isoflurane, and the uterus was externalized from the abdominal cavity. A total of 1.0–2.0 μL of plasmid DNA (1.0 μg of pCAG-Venus and 5.0 μg of pCAG-neo for control; 1.0 μg of pCAG-Venus and 5.0 μg of pCAG-neo-3xHA-Syne1-KASH for LINC-DN) containing 0.01% Fast Green (Sigma-Aldrich) in PBS was injected into the fourth (for Purkinje cells) or lateral (for cortical neurons) ventricle with a glass capillary. Electroporation was performed by holding the embryo with a forceps-type electrode (CUY650P3, Nepagene) and delivering electrical pulses through the uterine wall using a NEPA21 electroporator under the following parameters: poring pulse—40 V, 30 ms duration, 50 ms interval, 2 pulses, 10% decay rate, + polarity; transfer pulse—10 V, 50 ms duration, 50 ms interval, 3 pulses, 40% decay rate, + polarity. After electroporation, the uterus was repositioned into the abdominal cavity, and the abdominal wall and skin were sutured to ensure continued normal embryonic development.

### Analysis of AIS

Images of the AIS visualized by Ankyrin-G staining were acquired using a BZ-X810 fluorescence microscope equipped with ×20 (for brain section neurons) or ×40 (for primary cultured neurons) objective lens. AIS length and its distance from the soma were analyzed using the BZ-H4M image analysis software (Keyence). AIS length was defined as the distance from the onset of the Ankyrin-G signal at the proximal axon near the soma to the point where the signal completely disappeared in the distal axon. Cells with Ankyrin-G-immunoreactive signals that were fragmented near the endpoint, branched along the axon, or had an unclear endpoint were excluded from the analysis. For spatial molecular profiling, immunoreactive signals of the AIS-localized proteins were manually traced with a line that accurately followed the AIS structure using PenTablet software (XP-PEN Technology), and signal intensities per 0.38-μm-length segments along the traced line were measured using ImageJ 1.53 software. Background intensity from regions lacking specific staining was subtracted from the values obtained in signal-positive regions. The resulting intensity profiles were exported to Excel (Microsoft), analyzed using a custom MATLAB/Octave script (Berger et al, 2018) (provided by Dr. JL Salzer) (Fig. 5H,K; Appendix Fig. S7B,E,H). The total intensity of the AIS-localized proteins was calculated by integrating the signal intensity of each segment along the traced line (Figs. 4B,D,F and 5I,L and Appendix Fig. S7C,F,I). In addition to the exclusion criteria applied for AIS length analysis, cells with overlapping immunoreactive signals within the AIS were excluded from the analysis.

### Stimulated emission depletion (STED) super-resolution imaging

Primary cortical neurons were electroporated to express either Venus alone or Venus in combination with LINC-DN and plated onto 15-mm diameter circular coverslips (NO.1SHT: thickness of 0.17 ± 0.005 mm, Matsunami Glass), pre-coated with 1 mg/mL PDL. After 2 weeks in culture, cells were fixed with 4% PFA in PBS for 20 min at RT, followed by quenching with 50 mM NH_4_Cl in PBS for 15 min at RT to reduce background fluorescence caused by PFA fixation. The fixed cells were permeabilized with 0.1% Triton X-100 in PBS for 5 min, and then incubated in blocking buffer containing 3% BSA in PBS for 1 h at RT. The cells were incubated with primary antibodies in the blocking buffer overnight at 4 °C, followed by a 3-h incubation at RT with secondary antibodies and STAR RED-conjugated phalloidin (STRED-0100, Abberior, 1:200) in the blocking buffer. After antibody incubation, the cells were washed three times in PBS for 10 min at RT and re-incubated with STAR RED-conjugated phalloidin for 30 min at RT. Finally, the cells were washed three times in PBS for 1 min at RT and mounted immediately using ProLong Diamond Antifade Mountant (Invitrogen).

STED imaging was performed using an Infinity instrument (Abberior), integrated with an inverted microscope (IX83, Olympus) and a ×60 objective lens (UPLXAPO60XO 1.42 N.A., Olympus). STED images of actin labeled with STAR RED-conjugated phalloidin were acquired with a pixel size of 20 nm, a dwell time of 10 µs, a pinhole of 1.0 AU, a pulsed 640-nm excitation laser line, and a pulsed 775-nm depletion laser line with 10% z-donut configuration. The images of Ankyrin-G (labeled with Alexa 594) and Venus (enhanced with Alexa 488) were obtained in confocal mode. The raw images were acquired using iMSPECTOR LiGHTBOX software (Abberior) and processed with a classical maximum likelihood estimation deconvolution algorithm (Huygens, Scientific Volume Imaging) to eliminate haze from the STED immunofluorescence signals. The periodicity of actin rings was evaluated by measuring the fluorescence intensity of actin along a 1-μm-length segment of an Ankyrin-G-labeled axon using ImageJ 1.53 software. Neurons with clearly disturbed periodicity of actin rings, determined by visual inspection, were classified as abnormal.

### Nuclear analysis

To analyze the expression of Sun1, Sun2, Nesprin-1, Nesprin-2, and Lamin B1 on the NE, and H3K9me3 within the nucleus, fluorescence images were acquired using a BZ-X810 fluorescence microscope equipped with a ×100 objective lens and ×2 digital magnification. Quantification of signal intensity on the NE was performed by manually tracing a line that accurately followed the contour of the NE using PenTablet software, and the total signal intensity along this line was measured using ImageJ 1.53 software. Cells exhibiting immunoreactive signals on the NE that could not be clearly distinguished from those of Golgi-localized aggregates were excluded from the analysis. For quantification of nuclear H3K9me3 signal, the NE was manually traced using PenTablet software to define the nuclear region, and total signal intensity within this area was measured using ImageJ 1.53 software. Background intensity from regions lacking specific staining was subtracted from the values obtained in signal-positive regions. The resulting data were exported to Excel and analyzed using a custom MATLAB/Octave script for nuclear analysis. For the analysis of nuclear structure, nuclei in layer V pyramidal neurons of the prefrontal cortex, layer II/III pyramidal neurons of the somatosensory cortex, and primary cultured cortical neurons were visualized by Lamin B1 staining, manually inspected, and those with two or more distortions were classified as structurally abnormal.

### Neurite analysis

To analyze neurite morphology, cortical neurons at 5 DIV were visualized via Venus expression. For dendritic structural analysis, fluorescence images of neurons were selected to ensure complete visibility of the entire dendritic arbor and then imported into Neurolucida 360 (MBF Bioscience). The soma was automatically detected, and dendrites were manually traced using PenTablet software. Reconstructed neurons were analyzed for the number of dendritic branches (nodes), total dendritic length, and the number of intersections between dendrites and concentric circles spaced at 10-μm intervals using the Sholl analysis method (Sholl, 1953) in Neurolucida Explorer (MBF Bioscience). Axon length was measured using BZ-H4M image analysis software. Axon and dendrites were classified based on Ankyrin-G immunoreactivity: neurites positive for Ankyrin-G were defined as axons, and all others were considered dendrites.

### Cell polarity analysis

mCherry-fused TfR-expressing primary cortical neurons were fixed at 21 DIV, and the ratio of TfR distribution between the axon and dendrites was evaluated. Using ImageJ 1.53 software, the total fluorescence intensity of mCherry along a 10-μm long line with a 0.17 μm width extending from the soma was measured for the axon and dendrites. The axon was identified by immunostaining for Ankyrin-G. For the dendrites, signals were measured from three per cell, and the average value was calculated.

### Cellular aggregates and cell death analyses

To analyze Golgi apparatus-localized aggregates of Sun1, Sun2, Nesprin-1, Nesprin-2, Lamin B1, or Ankyrin-G, the percentage of layer V pyramidal neurons in the prefrontal cortex containing five or more aggregates, each with a diameter of 0.5 μm or greater, was assessed in 3- and 20-month-old mice. Cell death in layer V pyramidal neurons of the prefrontal cortex was quantified based on pyknotic nuclear morphology, as observed through counterstaining with Hoechst 33342.

### Electrophysiology

Mice were anesthetized with 3% isoflurane and then decapitated. The brain was quickly removed and placed in ice-cold cutting solution consisting of 189 mM sucrose, 10 mM D-(+)-glucose, 1.25 mM NaH_2_PO_4_, 24 mM NaHCO_3_, 3 mM KCl, 5 mM MgSO_4_, and 0.1 mM CaCl_2_. Coronal slices (350- or 600-μm thick) containing the prefrontal cortex were prepared using a microslicer (Linear Slicer PRO7, Dosaka EM). The slices were then incubated in oxygenated (5% CO_2_–95% O_2_) artificial cerebrospinal fluid (aCSF) containing 124 mM NaCl, 1.25 mM NaHPO_4_, 24 mM NaHCO_3_, 3 mM KCl, 1 mM MgSO_4_, 2 mM CaCl_2_, and 10 mM D-(+)-glucose for 1 h at RT before proceeding with electrophysiological recordings. After incubation, the slice was transferred to the recording chamber, which was continuously perfused with oxygenated aCSF (31 °C, 2 mL/min). The membrane potential was measured using blind whole-cell patch-clamp recordings with Axoclamp 2B (Axon Instruments). Patch pipettes were pulled from borosilicate glass (1B120F-3, World Precision Instruments) using a micropipette puller (P-87, Sutter Instruments) and were filled with an internal solution containing 130 mM K-gluconate, 20 mM NaCl, 4 mM Mg-ATP, 0.3 mM Na_2_-GTP, 0.2 mM EGTA, and 10 mM HEPES, pH 7.3. The pipette resistance was ~10 MΩ. The liquid junction potential (approximately -16 mV) was not corrected. The signals were low-pass filtered at 3 kHz and digitized at 20 kHz using an AD converter (PowerLab 8/35, ADInstruments) and software (Chart v7, ADInstruments). Neurons with a series resistance greater than 30 MΩ and a resting membrane potential less than −50 mV were excluded from the analysis. The membrane potential of neurons was held at −70 mV during excitability measurements, with a constant holding current applied. To determine the current threshold, 20-ms pulse currents were injected in 10-pA increments starting from the holding current, defining the threshold as the smallest current step that triggered an action potential. To assess the correlation between injected current and firing frequency, 500-ms pulse currents were applied in 50-pA increments, ranging from 50 to 500 pA. Negative step currents of 100, 200, and 300 pA were applied, and the change in membrane potential was measured. Input resistance was calculated as the slope of the regression line for these data. Following the recordings, pyramidal neurons were selected based on previously reported electrical properties of those in the prefrontal cortex(Dembrow et al, 2010; Kawaguchi, 1993), including action potentials with a full width at half maximum greater than 1 ms, tonic and regular firing patterns during prolonged current injection, and input resistances ranging from 100 to 400 MΩ. Furthermore, in specific experiments, 1% biocytin (Sigma-Aldrich) was included in the internal solution to label the recording neurons, enabling morphological confirmation of their identity as pyramidal neurons. Briefly, after the recording, biocytin was injected by applying a current pulse ( + 200 pA, 250-ms duration, and 2 Hz for over 10 min), followed by fixation of the slice in 4% PFA in PBS overnight at 4 °C. Endogenous peroxidase activity was blocked using 1% H_2_O_2_ in PBS for 30 min at RT. The slice was then permeabilized with 0.3% Triton X-100 in PBS for 15 min at RT, and incubated overnight at 4 °C with the avidin-biotin-peroxidase complex solution (VECTASTAIN Elite ABC-HRP Kit, Vector Laboratories). The labeled neurons were visualized by treating them with 0.05% 3,3-diaminobenzidine and 0.003% H_2_O_2_.

### Behavioral analysis

The open-field test was used to assess anxiety and locomotor activity (Benice et al, 2006; Li et al, 2020), while the elevated plus maze test specifically evaluated anxiety (Li et al, 2020). The novel object recognition test was performed to assess recognition memory (Li et al, 2020; Zanos et al, 2015). Mice were habituated to the experimenters for 3 days through gentle handling for 5 min each day prior to the tests. During this habituation period, the health of the mice was closely monitored, and those exhibiting abnormalities, such as tumors or cloudy eyes, were excluded from the subsequent tests. All behavioral tests were conducted from 9:00 to 13:00. The experimental apparatus was cleaned with 70% ethanol between trials.

#### Open-field test

A mouse was initially placed at the corner of the experimental apparatus (L44 × W44 × H45 cm: SCANET MV40, MELQUEST) and allowed to move freely for 30 min while its activity was recorded. The center of the apparatus floor was illuminated at 100 lux. The locomotor activity and the time spent in the center (10 × 10 cm) of the apparatus were automatically measured using the SCANET MV40 system.

#### Elevated plus maze test

The elevated plus maze consisted of a cross-shaped apparatus with two open arms and two closed arms surrounded by 15-cm walls. The height of the apparatus was 50 cm from the floor, and the center of the maze was illuminated at 100 lux. A mouse was initially placed in the center of the maze, and its behavior was recorded for the following 10 min. The time spent in the open arms was measured manually. Since total locomotor activity in mice declined with aging (Fig. 7H), the time spent in the open arm was strongly influenced by age. Therefore, to eliminate this effect, the anxiety index was calculated in the tests involving both young and aged mice. The anxiety index was determined by dividing the time spent in the open arm by the number of times the mouse turned its head toward the open arm, reflecting attention to that area.

#### Novel object recognition test

The test was conducted over 5 consecutive days. On day 1, a mouse was placed in the empty apparatus (L35 × W35 × H30 cm) with a white floor, illuminated at 100 lux, for 5 min to habituate. From days 2 to 4, two identical objects (plastic square bottles) were placed 5 cm apart from the corner of the apparatus, and the mouse was allowed to explore freely for 5 min (training phase). On day 5, one of the identical objects was replaced with a novel object (metal circular bottle), and the mouse was given 5 min to explore. The locomotor activity and exploration time were analyzed using ANY-maze (Stoelting). Exploration was defined as the mouse orienting its head toward an object within 1.0 cm. The discrimination index (DI) was calculated as follows:

$${{\rm{DI}}}=\frac{{Exploring}\,{time}\,{for}\,{novel}\,{object}-{Exploring}\,{time}\,{for}\,{identical}\,{object}}{{Exploring}\,{time}\,{for}\,{novel}\,{object}+{Exploring}\,{time}\,{for}\,{identical}\,{object}}$$

Mice that stuck to either the left or right identical object or did not explore any objects during the training phases were excluded from the experiment.

### Isolation of neuronal nuclei

Mice infected with AAV-hSyn-eGFP-Cre were anesthetized with 3% isoflurane and decapitated. The cerebrum was quickly removed and placed in an ice-cold 54% Percoll (Cytiva) solution containing a protease inhibitor cocktail (cOmplete Mini, EDTA-free, Roche) and then homogenized with five strokes at 1000 rpm using an overhead stirrer (WHEATON). Nonidet(R) P40 Substitute (final concentration 0.1%, NACALAI TESQUE) was added to the homogenized cerebrum, suspended with a 1% BSA-coated tip, and incubated for 15 min on ice. After incubation, the cortical cell lysates were mixed with a Tris-based buffer (50 mM Tris-HCl, pH 7.4, 25 mM KCl, 5 mM MgCl_2_, and 250 mM sucrose) at a 1:1 ratio, suspended with 10 strokes using a 1% BSA-coated tip, and then placed in a 1.5-mL microfuge tube (Beckman Coulter) coated with 1% BSA. The lysates were ultracentrifuged at 30,000×*g* for 10 min at 4 °C in a Percoll density gradient prepared with 31% and 35% solutions. After centrifugation, the cell nuclear fraction was collected as approximately one-quarter of the total volume by puncturing the bottom of the tube with a needle. The neuronal nuclei were isolated from the GFP-positive population using Cell Sorter SH800 (SONY), collected in 15-mL tubes coated with DMEM containing 10% FBS and then gently centrifuged at 2600×*g* for 30 min at 4 °C. The pelleted neuronal nuclei were resuspended in ice-cold PBS, stained with 300 nM DAPI solution, and counted.

### Hi-C analysis

The Hi-C libraries were prepared using an Arima-HiC Kit (Arima Genomics) according to the manufacturer’s instructions for Mammalian Cell Lines (A160134 v01) and Library Preparation using a KAPA Hyper Prep Kit (A160139 v00). Briefly, neuronal nuclei were crosslinked with 37% formaldehyde solution, and 1 × 10^6^ nuclei were digested with two restriction enzymes (^GATC and G^ANTC). Biotinylated nucleotides were incorporated at the digested DNA ends, followed by ligation of spatially proximal DNA fragments. After reversing the crosslinks, the ligated DNA was sheared to approximately 400 bp using the Focused-ultrasonicator M220 (Covaris). Double-sided size selection was performed with SPRIselect beads (Beckman Coulter) at ratios of 1.0× and 0.5×. The size-selected DNA was enriched using streptavidin magnetic beads and subsequently subjected to end repair, dA-tailing, and adapter ligation. The library was then amplified with 10 cycles of PCR and purified twice using SPRI beads at ratios of 0.9× and 1.0×. The quality and concentration of the Hi-C libraries were assessed using the Qubit 4 Fluorometer (Thermo Fisher Scientific), the 2100 Bioanalyzer system (Agilent Technologies), and the 7900HT Fast Real-Time PCR System (Thermo Fisher Scientific). The final libraries were sequenced using the Illumina NovaSeq 6000 system with a read length of 150 bp.

### RNA-seq analysis

cDNA was directly amplified from 1000 nuclei using a SMART-Seq mRNA kit (Takara Bio) according to the manufacturer’s instructions. The cDNA was purified twice using 0.7× AMPure XP beads (Beckman Coulter). The quality and concentration of cDNA were assessed using the Qubit 3 Fluorometer (Thermo Fisher Scientific) and the 2100 Bioanalyzer system (Agilent Technologies). A sequencing library was prepared from 1 ng of purified cDNA using a Nextera XT DNA Library Preparation Kit (Illumina) according to the manufacturer’s instructions. Sequencing was performed on the Illumina NovaSeq 6000 with a paired-end read length of 2× 100 bp.

### Bioinformatic analysis

The mouse reference genome (GRCm39) excluding mitochondrial DNA was used for the following analyses. Gene annotations were retrieved from GENCODE (vM36) (Mudge et al, 2025), and ribosomal RNA sequences were obtained from the SILVA database (Glöckner, 2019). For RNA-seq data, adapter sequences were trimmed from raw reads using Cutadapt (v4.4) (Martin, 2011) with parameters *--trim-n -m 20:20 -a CTGTCTCTTATA -A CTGTCTCTTATA*. Ribosomal RNA reads were removed via Bowtie2 (v2.5.4) (Langmead et al, 2019) in end-to-end alignment mode. Cleaned reads were aligned to the reference genome using STAR (v2.7.11b) (Dobin et al, 2013) with parameters *--outFilterMultimapNmax 100 --quantMode TranscriptomeSAM --outFilterIntronMotifs RemoveNoncanonicalUnannotated*. Uniquely mapped reads were extracted using SAMtools (v1.21) (Li et al, 2009) (*-d NH:1*). Gene expression quantification was performed with RSEM (v1.3.1) (Li and Dewey, 2011) using default parameters. Differential expression analysis was conducted using DESeq2 (v1.46.0) (Love et al, 2014), and differentially expressed genes (DEGs) were defined as those with |log2(fold_change)| >1 and *P* value < 0.001. GO analysis of DEGs was performed in ShinyGO (v0.82) (Ge et al, 2020), in which *P* values were derived from hypergeometric tests and adjusted using Benjamini–Hochberg method. For visualization of gene expression heatmaps (Fig. 3J and EV4C), FPKM values were calculated by RSEM and subsequently subjected to z-score normalization on a per-gene basis. Specifically, for each gene, the mean FPKM across all nine samples (three biological replicates per condition) was subtracted, and the result was divided by the standard deviation across those samples, yielding a z-score for each sample. Heatmaps were generated using the SRplot (Tang et al, 2023) with the gene-wise z-score values. Hi-C reads were aligned to the reference genome using HiCUP (v0.9.2) (Wingett et al, 2015) with default parameters, producing BAM files. Pairix (v0.3.7) (Lee et al, 2022) was utilized to convert BAM files into pairs format with bam2pairs, and then Cooler (v0.10.3) (Abdennur and Mirny, 2020) converted the pairs format to COOL format for subsequent processing with HiCExplorer toolkit (v3.7.6) (Ramirez et al, 2018). Normalization of contact matrices was conducted with the hicNormalize module using default parameters. Matrix correction was applied via hicCorrectMatrix with the Knight-Ruiz (KR) matrix balancing algorithm (*--correctionMethod KR*). Chromatin interaction heatmaps generated by hicPlotMatrix module of HiCExplorer. Distance-dependent contact frequency profiles were computed using the hicPlotDistVsCounts module of HiCExplorer at 10-kb resolution, retaining only intra-chromosomal contacts at genomic distances ≥ 10 kb, and log-transformed prior to plotting to allow comparison across samples. To quantify the rate of change in contact frequency with increasing genomic distance, the first derivative of contact frequency with respect to genomic distance (d(Contacts)/d(Distance)) was calculated using a custom Python script. Briefly, contact frequency profiles were first smoothed using the Savitzky–Golay filter (window size = 51 bins, polynomial order = 3) as implemented in NumPy (Harris et al, 2020) to suppress high-frequency noise while preserving the overall decay structure, and the first derivative was subsequently computed via finite differences using numpy.gradient. Derivative profiles were plotted as a function of genomic distance on a log-scaled axis to visualize differences in chromatin compaction dynamics across conditions. For A/B compartment analysis, contact matrices were generated at 100-kb resolution. Principal component analysis (PCA) was performed on the observed/expected contact matrices using the hicPCA module (--format bedgraph -we 1), and the first principal component (PC1) values were used to define compartment identity. The sign of PC1 was assigned such that genomic regions with higher gene density and active histone marks corresponded to compartment A (PC1 > 0), and regions with repressive marks at the nuclear periphery corresponded to compartment B (PC1 < 0). PC1 values for specific genomic regions were integrated into the UCSC Genome Browser (Perez et al, 2025) for comparative visualization.

### qPCR analysis

Purified cDNA (5 ng) derived from isolated neuronal nuclei was used as a template in the PCR reaction with THUNDERBIRD SYBR qPCR Mix. The amplification condition using a real-time PCR system (Thermal Cycler Dice Real Time System IV, Takara Bio) was as follows: an initial denaturation at 95 °C for 30 s, followed by 40 cycles of denaturation at 95 °C for 5 s, and annealing/extension at 60 °C for 30 s. Dissociation curves were analyzed to verify the presence of a single product for each PCR reaction. *Gapdh* cDNA was used as a reference gene to calculate gene expression by the ΔΔCt method. The primers for qPCR are listed in Appendix Table S2.

### Statistics

Sample sizes for each experiment were determined based on prior studies without statistical consideration. All statistical comparisons without Bioinformatic analysis were made using GraphPad Prism 9 (GraphPad Software). No blinding was used in this study.

## Supplementary information

Appendix

Peer Review File

Source data Fig. 1

Source data Fig. 2

Source data Fig. 3

Source data Fig. 4

Source data Fig. 5

Source data Fig. 6

Source data Fig. 7

Figure EV1 Source Data

Figure EV2 Source Data

Figure EV3 Source Data

Figure EV4 Source Data

Figure EV5 Source Data

Figure EV6 Source Data

Figure EV7 Source Data

Appendix Figure S1 Source Data

Appendix Figure S2 Source Data

Appendix Figure S3 Source Data

Appendix Figure S4 Source Data

Appendix Figure S5 Source Data

Appendix Figure S6 Source Data

Appendix Figure S7 Source Data

Expanded View Figures

## Data Availability

The Hi-C and RNA-seq data sets are deposited in the DNA Data Bank of Japan under BioProject accession number PRJDB18989. These data are available at: https://ddbj.nig.ac.jp/search/entry/bioproject/PRJDB18989. The dataset for distance-dependent contact frequency, contact map, and compartment A/B analyses is available at 10.6084/m9.figshare.28827575. Files with the “.cool” extension contain the data on distance-dependent contact frequency and contact maps and relate to Figs. 3E and EV3B,C. Files with the “.bw” extension contain the data on compartment A/B analyses of Scn2a and Kcna2 genes and relate to Fig. EV3E,F. The source data of this paper are collected in the following database record: biostudies:S-SCDT-10_1038-S44319-026-00786-5.
