# Author Correction: Age-related decline in nuclear envelope LINC complex drives neuronal aging via axon initial segment dysfunction

**DOI:** 10.1038/s44319-026-00869-3

**Published:** 2026-07-29

**Authors:** Koichi Hasegawa, Noriyuki Hama, Mina Amemiya, Chao Zeng, Yasuyuki Ito, Sadafumi Suzuki, Keiichiro Nakamura, Junpei Kondo, Chiharu Takeda, Yuji Kurihara, Kazuho Ikeda, Yuki Fujita, Yasushi Okada, Atsushi Toyoda, Michiaki Hamada, Ken-ichiro Kuwako

**Affiliations:** 1 Department of Neural and Muscular Physiology, School of Medicine, Shimane University, Izumo, Japan; 2 Faculty of Science and Engineering, Waseda University, Tokyo, Japan; 3 Department of Developmental Biology, School of Medicine, Shimane University, Izumo, Japan; 4 Department of Cell Biology, Graduate School of Medicine, The University of Tokyo, Tokyo, Japan; 5 Laboratory for Cell Polarity Regulation, RIKEN Center for Biosystems Dynamics Research (BDR), Osaka, Japan; 6 Department of Physics, Graduate School of Science, The University of Tokyo, Tokyo, Japan; 7 Universal Biology Institute (UBI), The University of Tokyo, Tokyo, Japan; 8 International Research Center for Neurointelligence (WPI-IRCN), Institutes for Advanced Study, The University of Tokyo, Tokyo, Japan; 9 Comparative Genomics Laboratory, Department of Genomics and Evolutionary Biology, National Institute of Genetics, Mishima, Japan; 10 AIST-Waseda University Computational Bio Big-Data Open Innovation Laboratory (CBBD-OIL), National Institute of Advanced Industrial Science and Technology, Tokyo, Japan; 11 Present Address: Department of Clinical Laboratory Science, Sanyo Women’s College, Hiroshima, Japan

## Abstract

**Figure:**
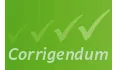

**Correction to:** *The EMBO Journal*. https://doi.org/10.1038/s44319-026-00786-5 | Published online 22 May 2026

The authors contacted the journal after discovering several textual errors in the published version. The journal has reviewed the matter and makes the following corrections.The errors do not affect the results, interpretations, or conclusions of the paper.

**The Figure 5 legend is corrected**.

**The Figure EV5 legend is corrected**.

**The Figure EV6 title is corrected**.

**Figure 5 Legend**.

**The Figure 5 Legend is corrected from:** (**A**–**C**) Control and LINC-DN-expressing cortical neurons at 7 or 21 (**A**) DIV. The AIS is indicated by the two arrowheads, and its length (**B**) and position (**C**), measured as the distance from the soma, were quantified. ease return to C, Control; DN, LINC-DN. The revised description may cause confusion for readers.

**To:** (**A**–**C**) Control and LINC-DN-expressing cortical neurons at 7 or 21 (**A**) DIV. The AIS is indicated by the two arrowheads, and its length (**B**) and position (**C**), measured as the distance from the soma, were quantified. C, Control; DN, LINC-DN.

**Figure EV5 Legend**.

**The Figure EV5 Legend is corrected from:** (**A**, **B**) Quantification of AIS length and position, measured as the distance from the soma, in control and LINC-DN-expressing hippocampal neurons at 21 DIV (**A**) and Purkinje cells at 7 DIV (**B**). The data represent the mean ± SEM. *n* = 50 (C, Control; DN, LINC-DN) (**A**).

**To:** (**A**, **B**) Quantification of AIS length and position, measured as the distance from the soma, in control and LINC-DN-expressing hippocampal neurons at 21 DIV (**A**) and Purkinje cells at 7 DIV (**B**). The data represent the mean ± SEM. *n* = 50 (Control and LINC-DN) (**A**).

**Figure EV6 Title**.

**The Figure EV6 title is corrected from:** Figure EV6: Effects of LINC complex inhibition on the AIS and neuronal viability in vivo.

**To:** Figure EV6: Effects of LINC complex inhibition on the AIS in vivo.

**Author statement:**

**Figure 5 legend**

During the editorial proofing process, text was inadvertently inserted into the Figure 5 legend and is now deleted.

**Figure EV5 legend**

The following text in the published Figure EV5 legend is incorrect:

**Published:** *“n = 50 (C, Control; DN, LINC-DN) (A)”*

**Corrected:** *“n = 50 (Control and LINC-DN) (A)”*

**Figure EV6 title**

During revision, the neuronal viability data were moved from Figure EV6 to Appendix Figure S4G. The title of Figure EV6 was not updated accordingly. The corrected title reads:

**Published:** *Figure EV6: Effects of LINC complex inhibition on the AIS and neuronal viability* in vivo.

**Corrected:** *Figure EV6: Effects of LINC complex inhibition on the AIS* in vivo.

None of these changes affect the results, interpretations, or conclusions of the paper.

