# Supplementary material for: Age-related decline in nuclear envelope LINC complex drives neuronal aging via axon initial segment dysfunction
Source: EMBO Rep. 2026 May 22;27(13):3788–825. doi: 10.1038/s44319-026-00786-5 (PMC13354796; doi:10.1038/s44319-026-00786-5)
Supplement: Supplementary file 1 — Appendix [file 44319_2026_786_MOESM1_ESM.pdf]

Appendix

Age-related decline in nuclear envelope LINC complex drives neuronal aging via axon initial segment dysfunction

Table of contents

Item	Page
Appendix Figure S1	2, 3
Appendix Figure S2	4
Appendix Figure S3	5, 6
Appendix Figure S4	7, 8
Appendix Figure S5	9
Appendix Figure S6	10
Appendix Figure S7	11
Appendix Table S1	12, 13
Appendix Table S2	14

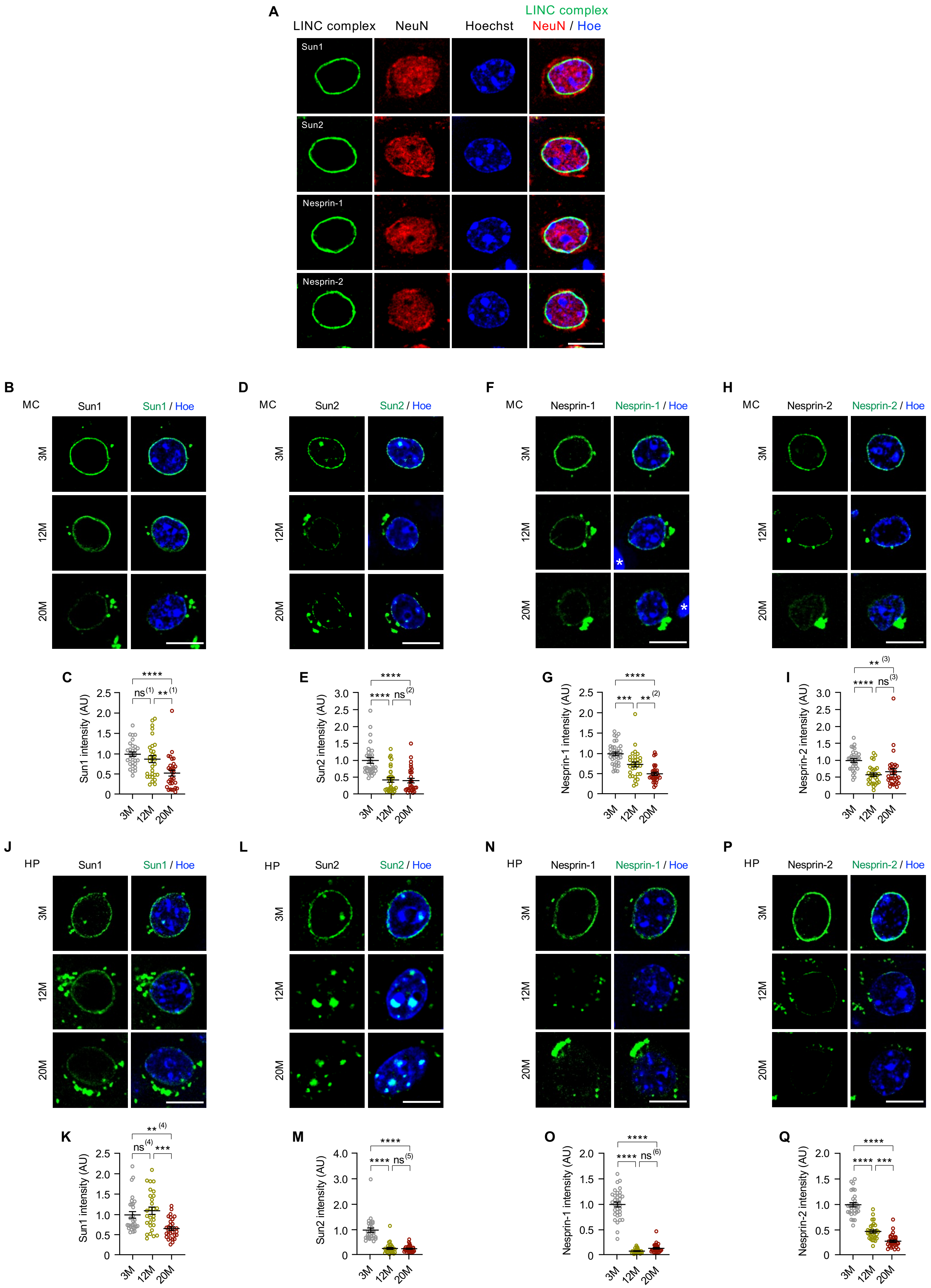

A
LINC complex
NeuN
Hoechst
LINC complex
NeuN / Hoe
Sun1
Sun2
Nesprin-1
Nesprin-2
B
MC
Sun1
Sun1 / Hoe
3M
12M
20M
C
Sun1 intensity (AU)
D
MC
Sun2
Sun2 / Hoe
E
Sun2 intensity (AU)
F
MC
Nesprin-1
Nesprin-1 / Hoe
G
Nesprin-1 intensity (AU)
H
MC
Nesprin-2
Nesprin-2 / Hoe
I
Nesprin-2 intensity (AU)
J
HP
Sun1
Sun1 / Hoe
K
Sun1 intensity (AU)
L
HP
Sun2
Sun2 / Hoe
M
Sun2 intensity (AU)
N
HP
Nesprin-1
Nesprin-1 / Hoe
O
Nesprin-1 intensity (AU)
P
HP
Nesprin-2
Nesprin-2 / Hoe
Q
Nesprin-2 intensity (AU)

Appendix Figure S1

Analysis of LINC complex expression in young and aged neurons.

(**A**) Neuronal expression of LINC complex molecules in young mice. Brain sections from 3-month-old mice were co-immunostained with antibodies against Sun1, Sun2, Nesprin-1, or Nesprin-2, along with the neuronal marker NeuN. (**B**–**Q**) Age-related changes of LINC complex expression. Brain sections from 3-, 12-, and 20-month-old mice were immunostained with antibodies against Sun1 (**B**, **J**), Sun2 (**D**, **L**), Nesprin-1 (**F**, **N**), or Nesprin-2 (**H**, **P**). Representative images are shown for the nucleus of layer V pyramidal neurons in the motor cortex (MC) and CA3 neurons in the hippocampus (HP). Asterisks indicate presumptive glial cells. Signal intensities of Sun1 (**C**, **K**), Sun2 (**E**, **M**), Nesprin-1 (**G**, **O**), and Nesprin-2 (**I**, **Q**) were quantified. The data represent the mean ± SEM. n=30 (3M), n=30 (12M), and n=31 (20M) for Sun1 in the MC (**C**); n=30 (3M, 12M, and 20M) for Sun2 in the MC (**E**); n=36 (3M), n=31 (12M), and n=31 (20M) for Nesprin-1 in the MC (**G**); n=31 (3M), n=30 (12M), and n=31 (20M) for Nesprin-2 in the MC (**I**); n=29 (3M), n=28 (12M), and n=30 (20M) for Sun1 in the HP (**K**); n=30 (3M), n=30 (12M), and n=33 (20M) for Sun2 in the HP (**M**); n=33 (3M), n=31 (12M), and n=31 (20M) for Nesprin-1 in the HP (**O**); n=30 (3M), n=30 (12M), and n=31 cells (20M) for Nesprin-2 in the HP (**Q**) from three brains. $^{**}P$=0.0035 (1), 0.0038 (2), 0.0022 (3), 0.0044 (4); $^{***}P$<0.001; $^{****}P$<0.0001; ns, not significant, *P*=0.4707 (1), 0.9781 (2), 0.6172 (3), 0.5978 (4), 0.9857 (5), 0.4465 (6) (ordinary one-way ANOVA Tukey's multiple comparison test). Scale bars: 10 µm.

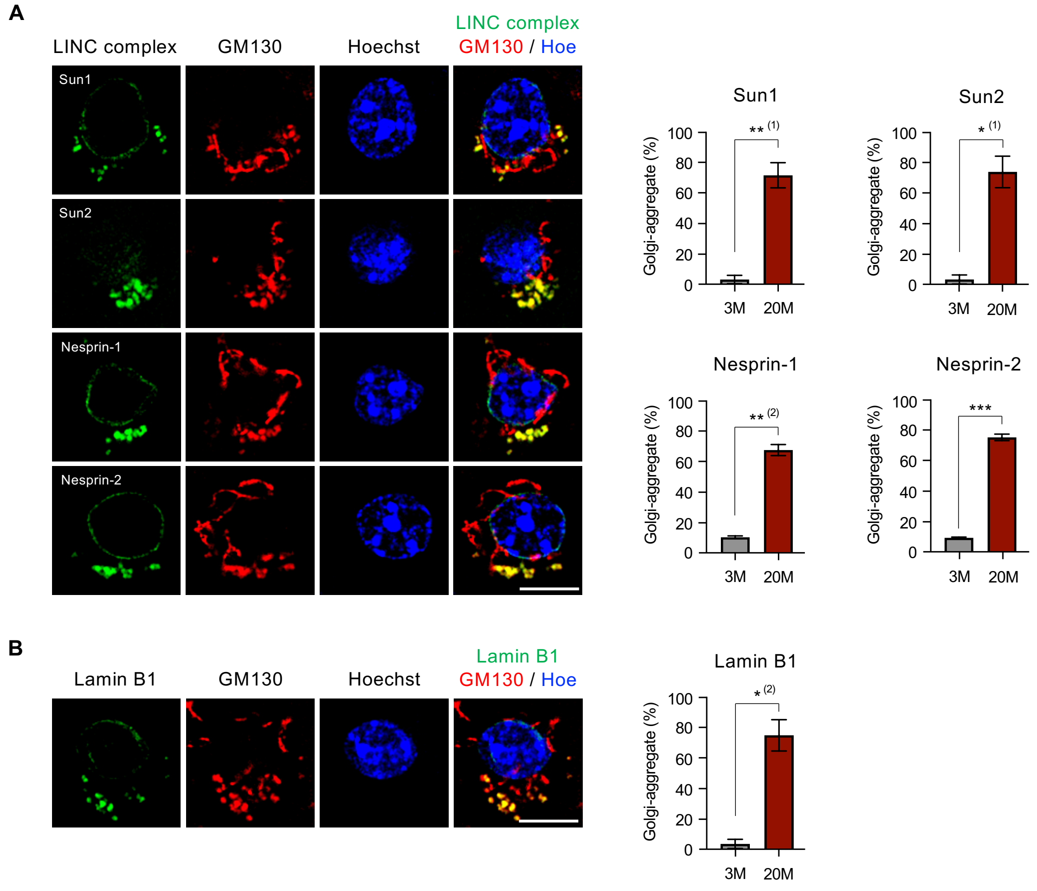

Appendix Figure S2

Analysis of the localization of LINC complex molecules in aged neurons.

(**A**, **B**) Brain sections from 20-month-old mice were co-immunostained with antibodies against LINC complex molecules (**A**) or Lamin B1 (**B**), along with GM130 (a marker for the Golgi apparatus). Representative images are shown for layer V pyramidal neurons in the prefrontal cortex. Note that LINC complex molecules and Lamin B1 are misaccumulated in the Golgi apparatus in aged neurons. The graphs show the percentage of cells containing five or more Golgi-localized aggregates (>0.5 μm) of LINC complex molecules (**A**) and Lamin B1 (**B**). The data represent the mean ± SEM. n=3 brains, eight–fourteen cells per brain (**A**, **B**). *P=0.0150 (1), 0.0142 (2); **P=0.0086 (1), 0.0025 (2); ***P<0.001 (unpaired two-tailed Welch's t-test). Scale bars: 10 μm.

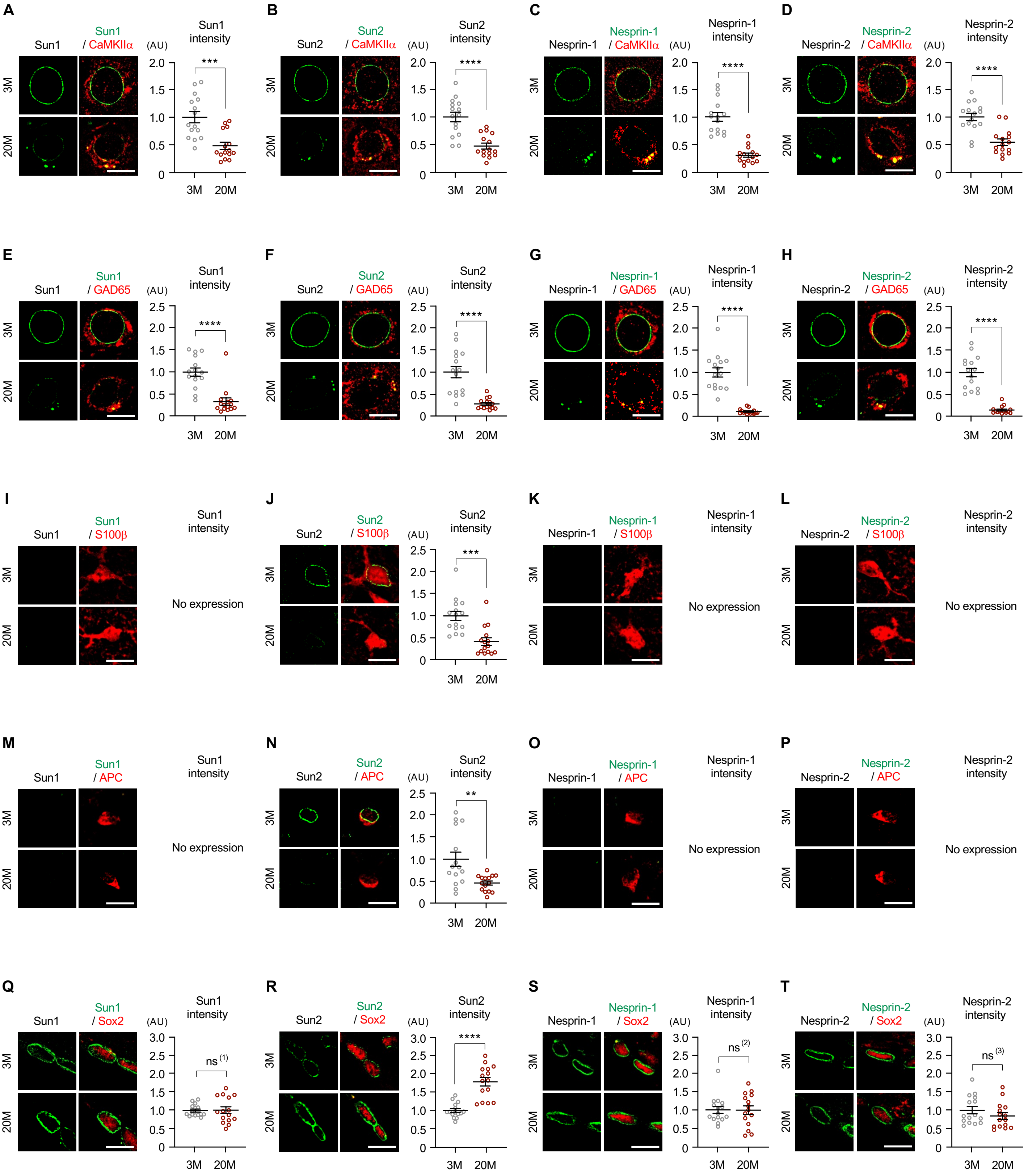
A
B
C
D
E
F
G
H
I
J
K
L
M
N
O
P
Q
R
S
T
Sun1
Sun2
Nesprin-1
Nesprin-2
/ CaMKIIα
/ GAD65
/ S100β
/ APC
/ Sox2
Sun1 intensity
Sun2 intensity
Nesprin-1 intensity
Nesprin-2 intensity
(AU)
3M
20M
No expression
ns (1)
ns (2)
ns (3)

Appendix Figure S3

Analysis of LINC complex expression in neural lineage cells.

(**A**–**T**) Brain sections from 3- and 20-month-old mice were co-immunostained with antibodies against Sun1, Sun2, Nesprin-1, or Nesprin-2, along with markers for specific cell types: CaMKIIα (glutamatergic neurons) (**A**–**D**), GAD65 (GABAergic neurons) (**E**–**H**), S100β (astrocytes) (**I**–**L**), APC (oligodendrocytes) (**M**–**P**), and Sox2 (neural stem cells) (**Q**–**T**). Representative images are shown for neurons and glial cells in layer V of the prefrontal cortex and neural stem cells in the dorsolateral subventricular zone. The graphs show signal intensity of LINC complex molecules on the NE. The data represent the mean ± SEM. n=15 (3M, 20M) for Sun1, Sun2, Nesprin-1, and Nesprin-2 (**A**–**H** and **Q**–**T**); n=15 cells (3M, 20M) for Sun2 (**J**, **N**) from three brains. ***P*=0.0052; ****P*<0.001; *****P*<0.0001; ns, not significant, *P*=0.9128 (1), 0.9725 (2), 0.2534 (3) (unpaired two-tailed Welch's *t*-test). Scale bars: 10 μm.

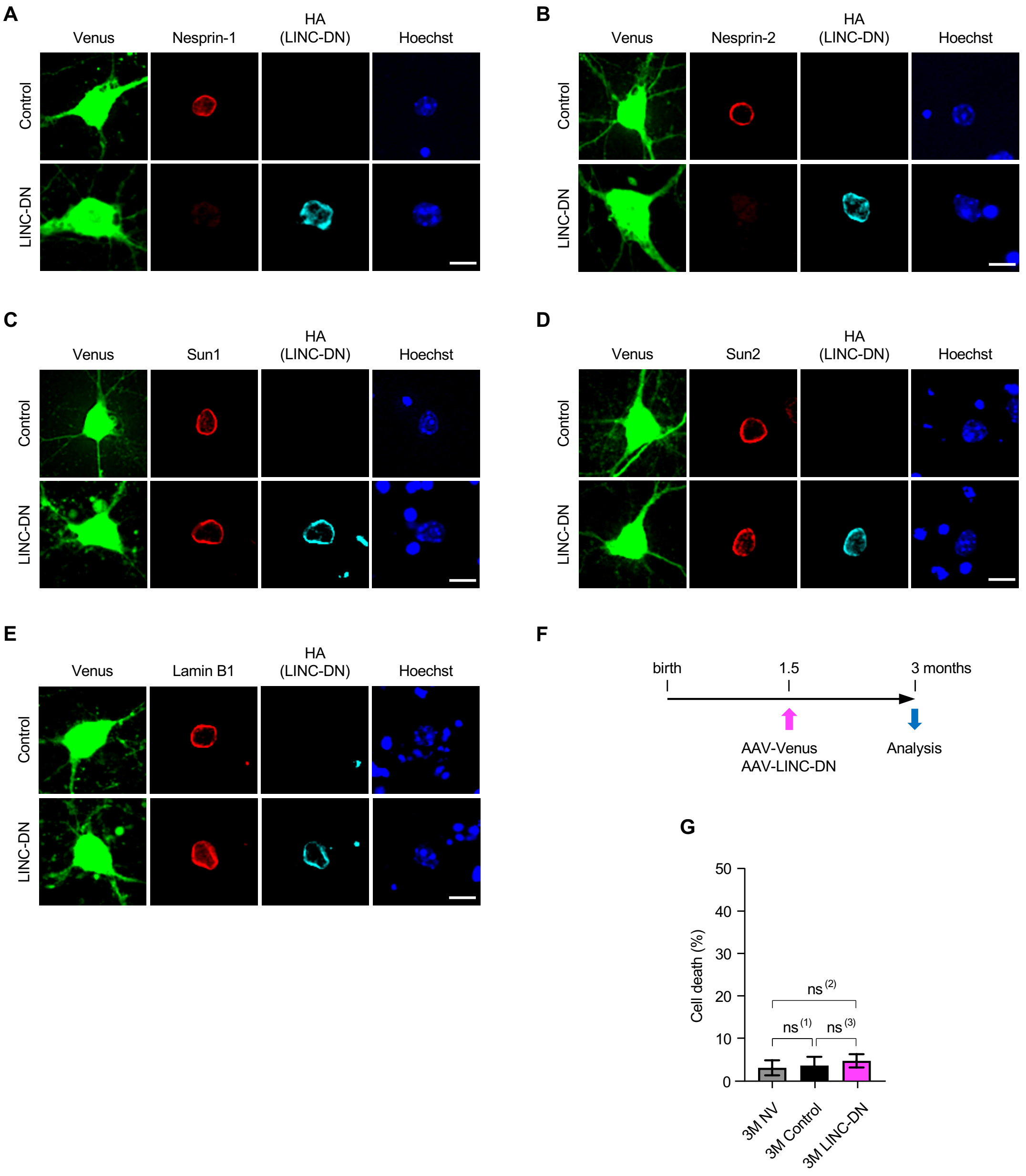
A
Venus
Nesprin-1
HA (LINC-DN)
Hoechst
Control
LINC-DN
B
Venus
Nesprin-2
HA (LINC-DN)
Hoechst
Control
LINC-DN
C
Venus
Sun1
HA (LINC-DN)
Hoechst
Control
LINC-DN
D
Venus
Sun2
HA (LINC-DN)
Hoechst
Control
LINC-DN
E
Venus
Lamin B1
HA (LINC-DN)
Hoechst
Control
LINC-DN
F
birth
1.5
3 months
AAV-Venus
AAV-LINC-DN
Analysis
G
Cell death (%)
0
10
20
30
40
50
ns (2)
ns (1)
ns (3)
3M NV
3M Control
3M LINC-DN

Appendix Figure S4

LINC complex inhibition by LINC-DN.

(**A**–**E**) Disruption of LINC complex assembly via LINC-DN. Control and LINC-DN-expressing cortical neurons at 21 DIV were co-immunostained with antibodies against Nesprin-1 (**A**), Nesprin-2 (**B**), Sun1 (**C**), Sun2 (**D**), or Lamin B1 (**E**), along with HA (for LINC-DN). Note that LINC-DN displaces endogenous Nesprin proteins but not Sun or Lamin B1 proteins from the NE. (**F**) Schematic of the AAV experiment to investigate the effects of neuronal expression of LINC-DN. 1.5-month-old mice were infected with AAV-Venus alone (3M Control) or AAV-Venus plus AAV-LINC-DN (3M LINC-DN), and analyzed at 3 months of age. (**G**) Analysis of cell death in layer V pyramidal neurons of the prefrontal cortex from 3-month-old mice in no virus (3M NV), AAV-Venus alone (3M Control), and AAV-Venus + AAV-LINC-DN (3M LINC-DN) groups. The data represent the mean ± SEM. n=4 brains, 13–21 cells per brain. ns, not significant, *P*=0.9770 (1), 0.8002 (2), 0.9006 (3) (ordinary one-way ANOVA Tukey's multiple comparison test). Scale bars: 10 μm.

A

Virus	Infection efficiency (% ± SEM)
AAV-Venus	80.6 ± 1.6
AAV-LINC-DN	96.9 ± 0.7
AAV-Sun1	94.5 ± 1.3
AAV-AnkG KO	87.1 ± 1.3

B

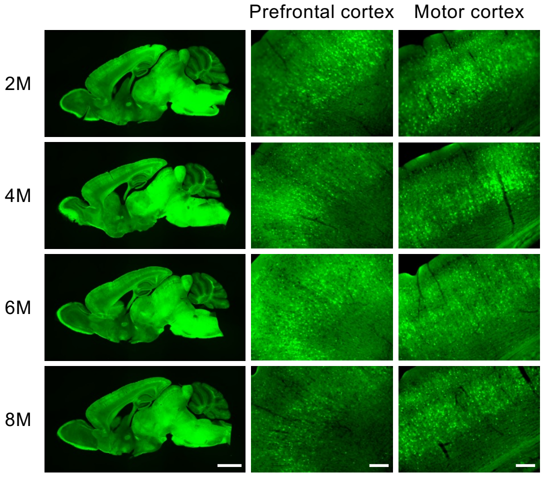

Appendix Figure S5

Characterization of AAV-mediated transgene expression.

(**A**) AAV infection efficiency in layer V pyramidal neurons in the prefrontal cortex (n=3–4 mice). (**B**) Brain sections at 2, 4, 6, and 8 months (M) after AAV-Venus administration. Representative images are shown for the whole brain (left panels) and prefrontal (middle panels) and motor (right panels) cortices. Note that Venus expression remains stable throughout the brain even 8 months after AAV administration. Scale bars: 2 mm (left panel in (**B**)); 200 µm (middle and right panels in (**B**)).

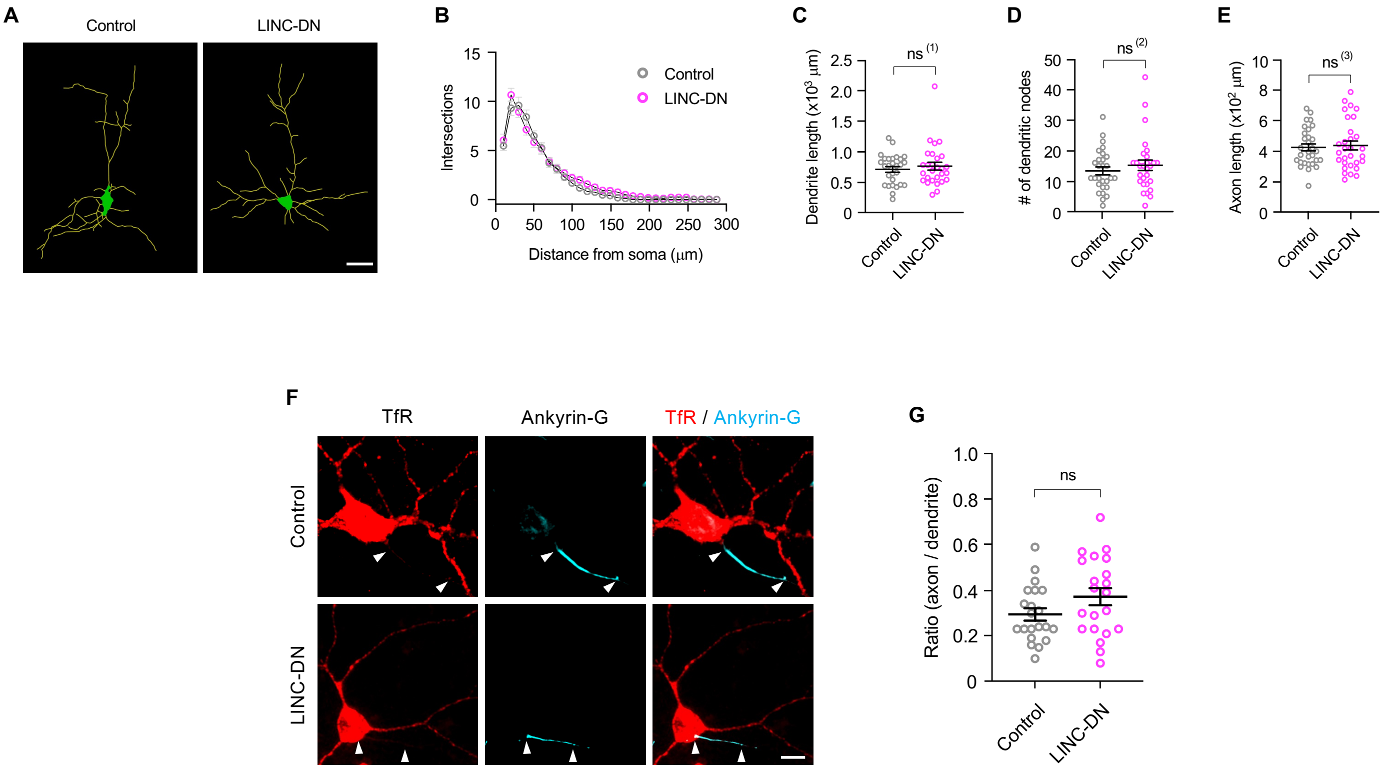

Appendix Figure S6

Effects of LINC complex inhibition on neurite morphology and cell polarity.

(**A**–**E**) Effects of LINC-DN expression on neurite morphology. Representative Neurolucida reconstructions of control and LINC-DN-expressing cortical neurons at 5 DIV are shown (**A**). The soma and dendrites are visualized in green and yellow, respectively. Neurons were analyzed for dendritic arborization (**B**), dendrite length (**C**), number of dendritic nodes (**D**), and axonal length (**E**). The data represent the mean ± SEM. n=28 (Control and LINC-DN) (**B**–**D**); n=30 cells (Control and LINC-DN) (**E**) from three independent experiments. ns, not significant, *P*=0.5154 (1), 0.3999 (2), 0.7349 (3) (unpaired two-tailed Welch's *t*-test). (**F**, **G**) Analysis of the intracellular distribution of TfR. Cortical neurons expressing mCherry-fused TfR, with or without LINC-DN, at 14 DIV are shown in (**F**). The AIS is indicated by the two arrowheads. The axon-to-dendrite ratio of TfR signal intensity was quantified (**G**). The data represent the mean ± SEM. n=21 cells from three independent experiments (Control and LINC-DN). ns, not significant, *P*=0.1026. (unpaired two-tailed Welch's *t*-test). Scale bars: 20 µm (**A**) and 10 µm (**F**).

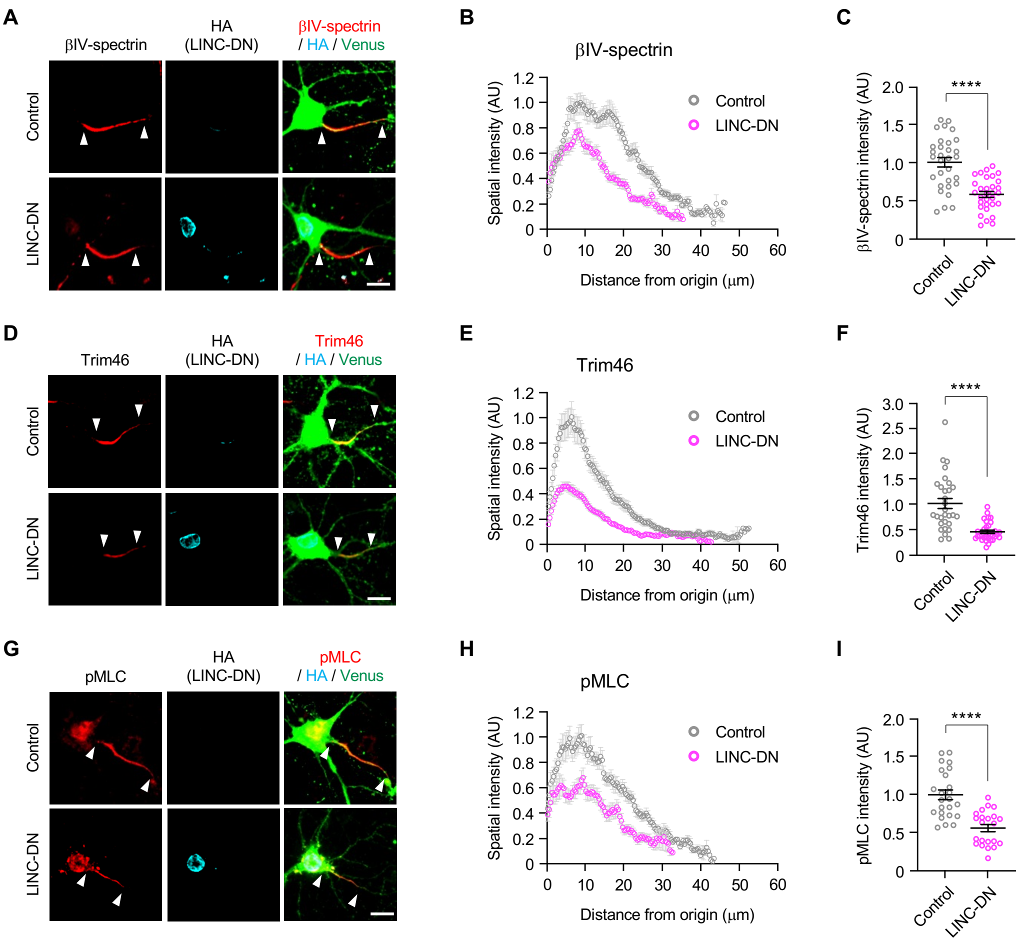

Appendix Figure S7

Spatial profiles of AIS-related molecules.

(**A**–**I**) Control and LINC-DN-expressing cortical neurons at 21 DIV were co-immunostained with antibodies against βIV-spectrin (**A**), Trim46 (**D**), or pMLC (**G**), along with HA (for LINC-DN). The AIS is indicated by the two arrowheads. The spatial intensity (**B**, **E**, **H**) and total intensity (**C**, **F**, **I**) of βIV-spectrin, Trim46, and pMLC in the AIS were quantified. The data represent the mean ± SEM. n=31 (Control and LINC-DN) (**B**, **C**); n=30 (Control) and n=32 (LINC-DN) (**E**, **F**); n=24 (Control) and n=22 cells (LINC-DN) (**H**, **I**) from three independent experiments. ****P<0.0001 (unpaired two-tailed Welch's t-test). Scale bars: 10 µm.

A *Sun1* gRNA off-target

	Locus	Off-target sequence	PAM	Gene	Edit
#1	chr15:+37020587	TTTGGTCTGCTCACatATtA	GCCTCC	-	No
#2	chr9:-24913250	TTaGGTCTGCTCAaGAAaCA	TTTTCC	-	No
#3	chr10:-15715531	TTTGGTCTGCTtACcAATaA	GGAACC	-	No
#4	chr15:-85168741	TcTGGTCaGCTCACaAAcCA	TCACCC	-	No
#5	chr5:+89827178	TTTGcTCTcCTtACaAATCA	CAGTCC	-	No
#6	chr2:-90810023	TTgGGTgTGCaCAaGAATCA	GCGCCC	-	No
#7	chr4:-40227392	TTTaGTgTGCTCAgGAATgA	CGCCCC	-	No
#8	chrX:-76692469	TTTGcTCTGCcCACaAATCt	TCCTCC	-	No
#9	chr14:+51981982	TTTGGgCTGCcCACcAATCc	TTAGCC	-	No
#10	chr13:-70009086	TTTGGTCTtCTgAtGAATgA	CAAGCC	-	No

B *Sun2* gRNA off-target

	Locus	Off-target sequence	PAM	Gene	Edit
#1	chr15:-96166450	TCcCAGGATGATtgCGATGG	GGCACC	-	No
#2	chr2:+84620039	gCTCAGGATGAaAACGATGc	AGGTCC	*Ctnnd1*	No
#3	chr17:+88121560	TCTgAGGATGcTAAgGATGG	GAAGCC	-	No
#4	chrX:+70355498	TCTCAGGcaGATAAtGATGG	AGACCC	-	No
#5	chr10:-62432856	TCTCAGGATGATgAgGcTGG	AGGCCC	-	No
#6	chr13:+6623438	cCTCAGGATGAaAACtATGG	ACAACC	*Pfkc*	No
#7	chr7:+51154675	TCTgAaGATGATgAtGATGG	TTCCCC	-	No
#8	chr4:-56501559	TCTCAtGAaGAgAACGAaGG	GTGACC	-	No
#9	chr6:-83505080	TCTCAGGgTGtTAtCcATGG	GAGGCC	-	No
#10	chr18:+58292666	TCTCAGGATGAaAAgGcTGa	GAACCC	-	No

C *Ank3* gRNA off-target

	Locus	Off-target sequence	PAM	Gene	Edit
#1	chr3:+129548229	TGCGATCCCtGGgaaGTTTGCaGTGCAGCC	GCAGCC	-	No
#2	chr7:+101388915	TGgGAcCCaGGGAaCGTcTGCaGTGCCACC	GCCACC	-	No
#3	chr9:+21343297	TGCtAcCCCGGGACCGcTTtCaGaAAAGCC	AAAGCC	-	No
#4	chr13:+108301970	TGCtATtCaGGGACCaTTTGtGaTGAAGCC	GAAGCC	-	No
#5	chr3:+27190094	aGCGtTCCCaGGACaGTcTGaGGTGTGTCC	GTGTCC	-	No
#6	chr4:+81307387	TGatATCCCtGGACaGTTTGCaGaAACACC	AACACC	-	No
#7	chr14:-101616787	TGaGATCCCaGGAtCcTTTGCccTGATTCC	GATTCC	-	No
#8	chr12:+103989379	TctGATgCtGGGACaGTTTGCtGTCTGTCC	CTGTCC	-	No
#9	chr17:+5979009	TGCtAgCCaaGGAtCGTTTcCGGTTCCCCC	TCCCCC	-	No
#10	chr11:+43493331	TGaGAagCtGGGcCtGTTTGCGGTCTTCCC	CTTCCC	-	No

Appendix Table S1

Off-target analysis in gene-editing.

(**A**–**C**) List of off-target loci and sequences, proto-spacer adjacent motif (PAMs), and editing results by Nme2Cas9 for the deletion of Sun1 (**A**), Sun2 (**B**), and Ankyrin-G (**C**). The '+' or '–' in the Locus column denotes the sense and antisense DNA strands, respectively. Lowercase letters in the Off-target sequence column indicate mismatched bases relative to the gRNA sequence. In the Gene column, a notation of '–' indicates that the target sequence is located in a non-coding region of the genome. A notation of 'No' in the Edit column indicates no genome editing at the target sequence.

Gene	Forward (5′ – 3′)	Reverse (5′ – 3′)	Amplicon (bp)
Sun1	AGGCAGCATGGTCTGAGACGGT	GTGTTCTCCGGCACACACTGGG	82
Sun2	AGAGTCCTACATCGGCAGCCCC	CCGCTCCAGTAGGGCTCACTGT	90
Syne1	CCAGAGCATCTTCCCGGTCCCA	CACCATGGGAGGCTTCCGCTTG	137
Syne2	TGCTGCCCACGGAAGATGGAGA	GGAGGTGTGTGCTTTGCCAGCT	136
Scn1a	GGTCATGGTGATTGGGAACCTTG	CATCCTGTCCACAGCAATCTGC	136
Scn2a	GGTCATGGTGATTGGGAACCTTG	CACGGCTATCTGGAGGTTGTTC	127
Scn8a	AGTCCCAGTGGAGCAGCCTGAG	GCAGGATCCACCACGACTTGCC	128
Kcna2	GTGACGGGGACTGAGCTGCCTA	AGCCCCAGAGCCCTTTGTGAGT	130
Kcnq3	CTGCGGAACTCATCCAGGCTGC	TTTTGGCTGGCTGCTGCTTCCA	145
Nfasc	ACGAGGGCATCAGCAGTACCGT	AGCACCAGAAGGGCAATGGCAC	124
Cntnap2	TGGCATCGGGTCACTGCAGAGA	GGCCTCCAGCACCACCAACAAA	145
Dlg2	GGTGGCAAGCCAGAAGGGTCAC	CTTTCCACCCTCCGCTTGCTGG	79
Gapdh	GAATACGGCTACAGCAACAG	GCAGCGAACTTTATTGATGGTA	234

Appendix Table S2

List of qPCR primer sequences.

List of gene names, their corresponding primer sequences for cDNA amplification, and the expected amplicon sizes.
