# Supplementary figures and images for: Age-related decline in nuclear envelope LINC complex drives neuronal aging via axon initial segment dysfunction (part 1 of 9)
Source: EMBO Rep. 2026 May 22;27(13):3788–825. doi: 10.1038/s44319-026-00786-5 (PMC13354796; doi:10.1038/s44319-026-00786-5)

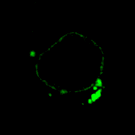

Supplement: Supplementary file 3 — Source data Fig. 1 [file 44319_2026_786_MOESM3_ESM.zip › Figure 1 Source Data/1I/Sun1_20M.tif]

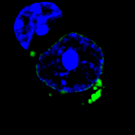

Supplement: Supplementary file 3 — Source data Fig. 1 [file 44319_2026_786_MOESM3_ESM.zip › Figure 1 Source Data/1I/Merge_20M.tif]

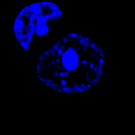

Supplement: Supplementary file 3 — Source data Fig. 1 [file 44319_2026_786_MOESM3_ESM.zip › Figure 1 Source Data/1I/Hoechst_20M.tif]

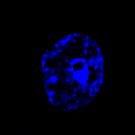

Supplement: Supplementary file 3 — Source data Fig. 1 [file 44319_2026_786_MOESM3_ESM.zip › Figure 1 Source Data/1I/Hoechst_12M.tif]

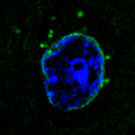

Supplement: Supplementary file 3 — Source data Fig. 1 [file 44319_2026_786_MOESM3_ESM.zip › Figure 1 Source Data/1I/Merge_12M.tif]

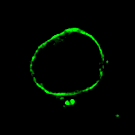

Supplement: Supplementary file 3 — Source data Fig. 1 [file 44319_2026_786_MOESM3_ESM.zip › Figure 1 Source Data/1I/Sun1_3M.tif]

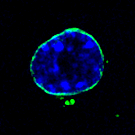

Supplement: Supplementary file 3 — Source data Fig. 1 [file 44319_2026_786_MOESM3_ESM.zip › Figure 1 Source Data/1I/Merge_3M.tif]

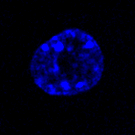

Supplement: Supplementary file 3 — Source data Fig. 1 [file 44319_2026_786_MOESM3_ESM.zip › Figure 1 Source Data/1I/Hoechst_3M.tif]

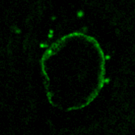

Supplement: Supplementary file 3 — Source data Fig. 1 [file 44319_2026_786_MOESM3_ESM.zip › Figure 1 Source Data/1I/Sun1_12M.tif]

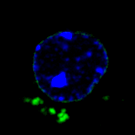

Supplement: Supplementary file 3 — Source data Fig. 1 [file 44319_2026_786_MOESM3_ESM.zip › Figure 1 Source Data/1A/Merge_20M.tif]

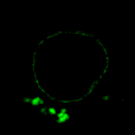

Supplement: Supplementary file 3 — Source data Fig. 1 [file 44319_2026_786_MOESM3_ESM.zip › Figure 1 Source Data/1A/Sun1_20M.tif]

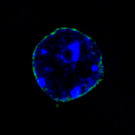

Supplement: Supplementary file 3 — Source data Fig. 1 [file 44319_2026_786_MOESM3_ESM.zip › Figure 1 Source Data/1A/Merge_12M.tif]

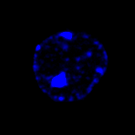

Supplement: Supplementary file 3 — Source data Fig. 1 [file 44319_2026_786_MOESM3_ESM.zip › Figure 1 Source Data/1A/Hoechst_20M.tif]

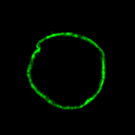

Supplement: Supplementary file 3 — Source data Fig. 1 [file 44319_2026_786_MOESM3_ESM.zip › Figure 1 Source Data/1A/Sun1_3M.tif]

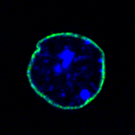

Supplement: Supplementary file 3 — Source data Fig. 1 [file 44319_2026_786_MOESM3_ESM.zip › Figure 1 Source Data/1A/Merge_3M.tif]

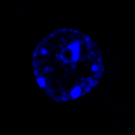

Supplement: Supplementary file 3 — Source data Fig. 1 [file 44319_2026_786_MOESM3_ESM.zip › Figure 1 Source Data/1A/Hoechst_12M.tif]

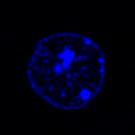

Supplement: Supplementary file 3 — Source data Fig. 1 [file 44319_2026_786_MOESM3_ESM.zip › Figure 1 Source Data/1A/Hoechst_3M.tif]

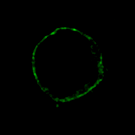

Supplement: Supplementary file 3 — Source data Fig. 1 [file 44319_2026_786_MOESM3_ESM.zip › Figure 1 Source Data/1A/Sun1_12M.tif]

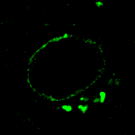

Supplement: Supplementary file 3 — Source data Fig. 1 [file 44319_2026_786_MOESM3_ESM.zip › Figure 1 Source Data/1M/Nesprin-1_20M.tif]

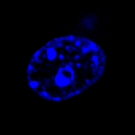

Supplement: Supplementary file 3 — Source data Fig. 1 [file 44319_2026_786_MOESM3_ESM.zip › Figure 1 Source Data/1M/Hoechst_20M.tif]

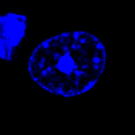

Supplement: Supplementary file 3 — Source data Fig. 1 [file 44319_2026_786_MOESM3_ESM.zip › Figure 1 Source Data/1M/Hoechst_12M.tif]

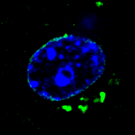

Supplement: Supplementary file 3 — Source data Fig. 1 [file 44319_2026_786_MOESM3_ESM.zip › Figure 1 Source Data/1M/Merge_20M.tif]

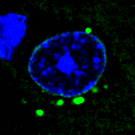

Supplement: Supplementary file 3 — Source data Fig. 1 [file 44319_2026_786_MOESM3_ESM.zip › Figure 1 Source Data/1M/Merge_12M.tif]

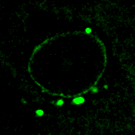

Supplement: Supplementary file 3 — Source data Fig. 1 [file 44319_2026_786_MOESM3_ESM.zip › Figure 1 Source Data/1M/Nesprin-1_12M.tif]

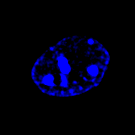

Supplement: Supplementary file 3 — Source data Fig. 1 [file 44319_2026_786_MOESM3_ESM.zip › Figure 1 Source Data/1M/Hoechst_3M.tif]

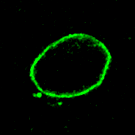

Supplement: Supplementary file 3 — Source data Fig. 1 [file 44319_2026_786_MOESM3_ESM.zip › Figure 1 Source Data/1M/Nesprin-1_3M.tif]

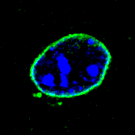

Supplement: Supplementary file 3 — Source data Fig. 1 [file 44319_2026_786_MOESM3_ESM.zip › Figure 1 Source Data/1M/Merge_3M.tif]

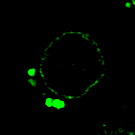

Supplement: Supplementary file 3 — Source data Fig. 1 [file 44319_2026_786_MOESM3_ESM.zip › Figure 1 Source Data/1G/Nesprin-2_12M.tif]

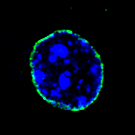

Supplement: Supplementary file 3 — Source data Fig. 1 [file 44319_2026_786_MOESM3_ESM.zip › Figure 1 Source Data/1G/Merge_3M.tif]

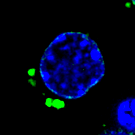

Supplement: Supplementary file 3 — Source data Fig. 1 [file 44319_2026_786_MOESM3_ESM.zip › Figure 1 Source Data/1G/Merge_12M.tif]

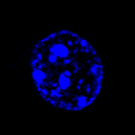

Supplement: Supplementary file 3 — Source data Fig. 1 [file 44319_2026_786_MOESM3_ESM.zip › Figure 1 Source Data/1G/Hoechst_3M.tif]

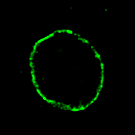

Supplement: Supplementary file 3 — Source data Fig. 1 [file 44319_2026_786_MOESM3_ESM.zip › Figure 1 Source Data/1G/Nesprin-2_3M.tif]

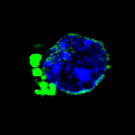

Supplement: Supplementary file 3 — Source data Fig. 1 [file 44319_2026_786_MOESM3_ESM.zip › Figure 1 Source Data/1G/Merge_20M.tif]

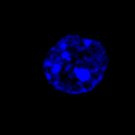

Supplement: Supplementary file 3 — Source data Fig. 1 [file 44319_2026_786_MOESM3_ESM.zip › Figure 1 Source Data/1G/Hoechst_20M.tif]

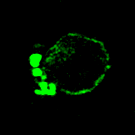

Supplement: Supplementary file 3 — Source data Fig. 1 [file 44319_2026_786_MOESM3_ESM.zip › Figure 1 Source Data/1G/Nesprin-2_20M.tif]

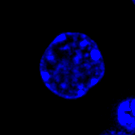

Supplement: Supplementary file 3 — Source data Fig. 1 [file 44319_2026_786_MOESM3_ESM.zip › Figure 1 Source Data/1G/Hoechst_12M.tif]

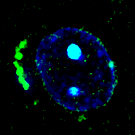

Supplement: Supplementary file 3 — Source data Fig. 1 [file 44319_2026_786_MOESM3_ESM.zip › Figure 1 Source Data/1C/Merge_12M.tif]

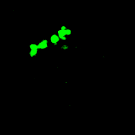

Supplement: Supplementary file 3 — Source data Fig. 1 [file 44319_2026_786_MOESM3_ESM.zip › Figure 1 Source Data/1C/Sun2_20M.tif]

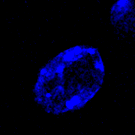

Supplement: Supplementary file 3 — Source data Fig. 1 [file 44319_2026_786_MOESM3_ESM.zip › Figure 1 Source Data/1C/Hoechst_20M.tif]

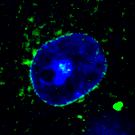

Supplement: Supplementary file 3 — Source data Fig. 1 [file 44319_2026_786_MOESM3_ESM.zip › Figure 1 Source Data/1C/Merge_3M.tif]

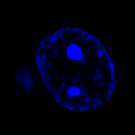

Supplement: Supplementary file 3 — Source data Fig. 1 [file 44319_2026_786_MOESM3_ESM.zip › Figure 1 Source Data/1C/Hoechst_12M.tif]

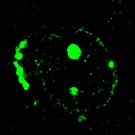

Supplement: Supplementary file 3 — Source data Fig. 1 [file 44319_2026_786_MOESM3_ESM.zip › Figure 1 Source Data/1C/Sun2_12M.tif]

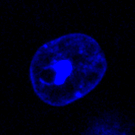

Supplement: Supplementary file 3 — Source data Fig. 1 [file 44319_2026_786_MOESM3_ESM.zip › Figure 1 Source Data/1C/Hoechst_3M.tif]

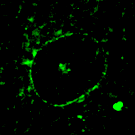

Supplement: Supplementary file 3 — Source data Fig. 1 [file 44319_2026_786_MOESM3_ESM.zip › Figure 1 Source Data/1C/Sun2_3M.tif]

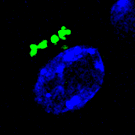

Supplement: Supplementary file 3 — Source data Fig. 1 [file 44319_2026_786_MOESM3_ESM.zip › Figure 1 Source Data/1C/Merge_20M.tif]

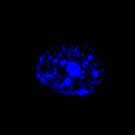

Supplement: Supplementary file 3 — Source data Fig. 1 [file 44319_2026_786_MOESM3_ESM.zip › Figure 1 Source Data/1O/Hoechst_20M.tif]

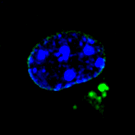

Supplement: Supplementary file 3 — Source data Fig. 1 [file 44319_2026_786_MOESM3_ESM.zip › Figure 1 Source Data/1O/Merge_12M.tif]

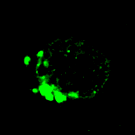

Supplement: Supplementary file 3 — Source data Fig. 1 [file 44319_2026_786_MOESM3_ESM.zip › Figure 1 Source Data/1O/Nesprin-2_20M.tif]

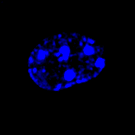

Supplement: Supplementary file 3 — Source data Fig. 1 [file 44319_2026_786_MOESM3_ESM.zip › Figure 1 Source Data/1O/Hoechst_12M.tif]

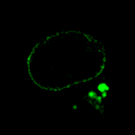

Supplement: Supplementary file 3 — Source data Fig. 1 [file 44319_2026_786_MOESM3_ESM.zip › Figure 1 Source Data/1O/Nesprin-2_12M.tif]

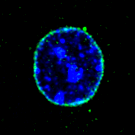

Supplement: Supplementary file 3 — Source data Fig. 1 [file 44319_2026_786_MOESM3_ESM.zip › Figure 1 Source Data/1O/Merge_3M.tif]

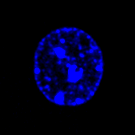

Supplement: Supplementary file 3 — Source data Fig. 1 [file 44319_2026_786_MOESM3_ESM.zip › Figure 1 Source Data/1O/Hoechst_3M.tif]

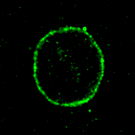

Supplement: Supplementary file 3 — Source data Fig. 1 [file 44319_2026_786_MOESM3_ESM.zip › Figure 1 Source Data/1O/Nesprin-2_3M.tif]

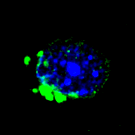

Supplement: Supplementary file 3 — Source data Fig. 1 [file 44319_2026_786_MOESM3_ESM.zip › Figure 1 Source Data/1O/Merge_20M.tif]

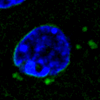

Supplement: Supplementary file 3 — Source data Fig. 1 [file 44319_2026_786_MOESM3_ESM.zip › Figure 1 Source Data/1K/Merge_20M.tif]

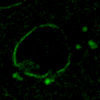

Supplement: Supplementary file 3 — Source data Fig. 1 [file 44319_2026_786_MOESM3_ESM.zip › Figure 1 Source Data/1K/Sun2_20M.tif]

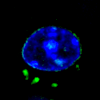

Supplement: Supplementary file 3 — Source data Fig. 1 [file 44319_2026_786_MOESM3_ESM.zip › Figure 1 Source Data/1K/Merge_12M.tif]

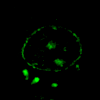

Supplement: Supplementary file 3 — Source data Fig. 1 [file 44319_2026_786_MOESM3_ESM.zip › Figure 1 Source Data/1K/Sun2_12M.tif]

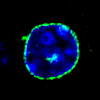

Supplement: Supplementary file 3 — Source data Fig. 1 [file 44319_2026_786_MOESM3_ESM.zip › Figure 1 Source Data/1K/Merge_3M.tif]

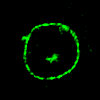

Supplement: Supplementary file 3 — Source data Fig. 1 [file 44319_2026_786_MOESM3_ESM.zip › Figure 1 Source Data/1K/Sun2_3M.tif]

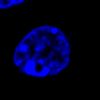

Supplement: Supplementary file 3 — Source data Fig. 1 [file 44319_2026_786_MOESM3_ESM.zip › Figure 1 Source Data/1K/Hoechst_20M.tif]

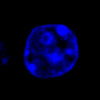

Supplement: Supplementary file 3 — Source data Fig. 1 [file 44319_2026_786_MOESM3_ESM.zip › Figure 1 Source Data/1K/Hoechst_3M.tif]

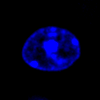

Supplement: Supplementary file 3 — Source data Fig. 1 [file 44319_2026_786_MOESM3_ESM.zip › Figure 1 Source Data/1K/Hoechst_12M.tif]

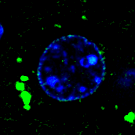

Supplement: Supplementary file 3 — Source data Fig. 1 [file 44319_2026_786_MOESM3_ESM.zip › Figure 1 Source Data/1E/Merge_20M.tif]

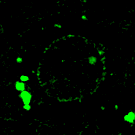

Supplement: Supplementary file 3 — Source data Fig. 1 [file 44319_2026_786_MOESM3_ESM.zip › Figure 1 Source Data/1E/Nesprin-1_20M.tif]

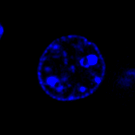

Supplement: Supplementary file 3 — Source data Fig. 1 [file 44319_2026_786_MOESM3_ESM.zip › Figure 1 Source Data/1E/Hoechst_20M.tif]

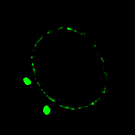

Supplement: Supplementary file 3 — Source data Fig. 1 [file 44319_2026_786_MOESM3_ESM.zip › Figure 1 Source Data/1E/Nesprin-1_12M.tif]

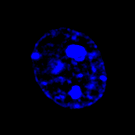

Supplement: Supplementary file 3 — Source data Fig. 1 [file 44319_2026_786_MOESM3_ESM.zip › Figure 1 Source Data/1E/Hoechst_12M.tif]

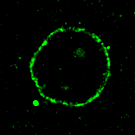

Supplement: Supplementary file 3 — Source data Fig. 1 [file 44319_2026_786_MOESM3_ESM.zip › Figure 1 Source Data/1E/Nesprin-1_3M.tif]

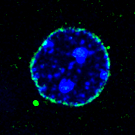

Supplement: Supplementary file 3 — Source data Fig. 1 [file 44319_2026_786_MOESM3_ESM.zip › Figure 1 Source Data/1E/Merge_3M.tif]

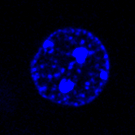

Supplement: Supplementary file 3 — Source data Fig. 1 [file 44319_2026_786_MOESM3_ESM.zip › Figure 1 Source Data/1E/Hoechst_3M.tif]

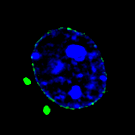

Supplement: Supplementary file 3 — Source data Fig. 1 [file 44319_2026_786_MOESM3_ESM.zip › Figure 1 Source Data/1E/Merge_12M.tif]

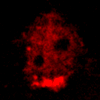

Supplement: Supplementary file 4 — Source data Fig. 2 [file 44319_2026_786_MOESM4_ESM.zip › Figure 2 Source Data/2I/NeuN_20M.tif]

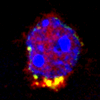

Supplement: Supplementary file 4 — Source data Fig. 2 [file 44319_2026_786_MOESM4_ESM.zip › Figure 2 Source Data/2I/Merge_20M.tif]

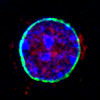

Supplement: Supplementary file 4 — Source data Fig. 2 [file 44319_2026_786_MOESM4_ESM.zip › Figure 2 Source Data/2I/Merge_3M.tif]

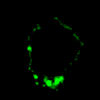

Supplement: Supplementary file 4 — Source data Fig. 2 [file 44319_2026_786_MOESM4_ESM.zip › Figure 2 Source Data/2I/Lamin B1_20M.tif]

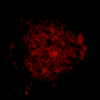

Supplement: Supplementary file 4 — Source data Fig. 2 [file 44319_2026_786_MOESM4_ESM.zip › Figure 2 Source Data/2I/NeuN_3M.tif]

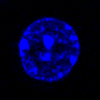

Supplement: Supplementary file 4 — Source data Fig. 2 [file 44319_2026_786_MOESM4_ESM.zip › Figure 2 Source Data/2I/Hoechst_3M.tif]

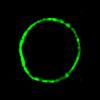

Supplement: Supplementary file 4 — Source data Fig. 2 [file 44319_2026_786_MOESM4_ESM.zip › Figure 2 Source Data/2I/Lamin B1_3M.tif]

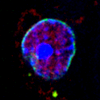

Supplement: Supplementary file 4 — Source data Fig. 2 [file 44319_2026_786_MOESM4_ESM.zip › Figure 2 Source Data/2I/Merge_12M.tif]

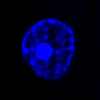

Supplement: Supplementary file 4 — Source data Fig. 2 [file 44319_2026_786_MOESM4_ESM.zip › Figure 2 Source Data/2I/Hoechst_12M.tif]

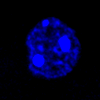

Supplement: Supplementary file 4 — Source data Fig. 2 [file 44319_2026_786_MOESM4_ESM.zip › Figure 2 Source Data/2I/Hoechst_20M.tif]

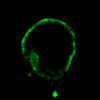

Supplement: Supplementary file 4 — Source data Fig. 2 [file 44319_2026_786_MOESM4_ESM.zip › Figure 2 Source Data/2I/Lamin B1_12M.tif]

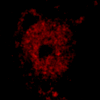

Supplement: Supplementary file 4 — Source data Fig. 2 [file 44319_2026_786_MOESM4_ESM.zip › Figure 2 Source Data/2I/NeuN_12M.tif]

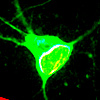

Supplement: Supplementary file 4 — Source data Fig. 2 [file 44319_2026_786_MOESM4_ESM.zip › Figure 2 Source Data/2D/Merge_3+DN.tif]

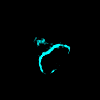

Supplement: Supplementary file 4 — Source data Fig. 2 [file 44319_2026_786_MOESM4_ESM.zip › Figure 2 Source Data/2D/HA_3+DN.tif]

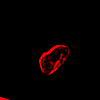

Supplement: Supplementary file 4 — Source data Fig. 2 [file 44319_2026_786_MOESM4_ESM.zip › Figure 2 Source Data/2D/Lamin B1_3+DN.tif]

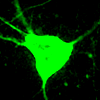

Supplement: Supplementary file 4 — Source data Fig. 2 [file 44319_2026_786_MOESM4_ESM.zip › Figure 2 Source Data/2D/Venus_3+DN.tif]

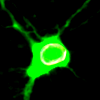

Supplement: Supplementary file 4 — Source data Fig. 2 [file 44319_2026_786_MOESM4_ESM.zip › Figure 2 Source Data/2D/Merge_2+DN.tif]

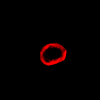

Supplement: Supplementary file 4 — Source data Fig. 2 [file 44319_2026_786_MOESM4_ESM.zip › Figure 2 Source Data/2D/Lamin B1_2+DN.tif]

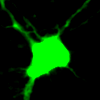

Supplement: Supplementary file 4 — Source data Fig. 2 [file 44319_2026_786_MOESM4_ESM.zip › Figure 2 Source Data/2D/Venus_2+DN.tif]

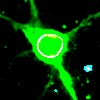

Supplement: Supplementary file 4 — Source data Fig. 2 [file 44319_2026_786_MOESM4_ESM.zip › Figure 2 Source Data/2D/Merge_1+DN.tif]

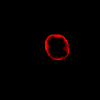

Supplement: Supplementary file 4 — Source data Fig. 2 [file 44319_2026_786_MOESM4_ESM.zip › Figure 2 Source Data/2D/Lamin B1_Control.tif]

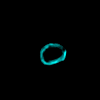

Supplement: Supplementary file 4 — Source data Fig. 2 [file 44319_2026_786_MOESM4_ESM.zip › Figure 2 Source Data/2D/HA_2+DN.tif]

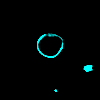

Supplement: Supplementary file 4 — Source data Fig. 2 [file 44319_2026_786_MOESM4_ESM.zip › Figure 2 Source Data/2D/HA_1+DN.tif]

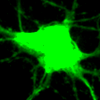

Supplement: Supplementary file 4 — Source data Fig. 2 [file 44319_2026_786_MOESM4_ESM.zip › Figure 2 Source Data/2D/Venus_Control.tif]

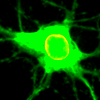

Supplement: Supplementary file 4 — Source data Fig. 2 [file 44319_2026_786_MOESM4_ESM.zip › Figure 2 Source Data/2D/Merge_Control.tif]

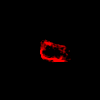

Supplement: Supplementary file 4 — Source data Fig. 2 [file 44319_2026_786_MOESM4_ESM.zip › Figure 2 Source Data/2D/Lamin B1_DN only.tif]

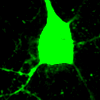

Supplement: Supplementary file 4 — Source data Fig. 2 [file 44319_2026_786_MOESM4_ESM.zip › Figure 2 Source Data/2D/Venus_DN only.tif]

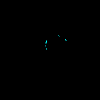

Supplement: Supplementary file 4 — Source data Fig. 2 [file 44319_2026_786_MOESM4_ESM.zip › Figure 2 Source Data/2D/HA_Control.tif]
