# Supplementary figures and images for: Age-related decline in nuclear envelope LINC complex drives neuronal aging via axon initial segment dysfunction (part 2 of 9)
Source: EMBO Rep. 2026 May 22;27(13):3788–825. doi: 10.1038/s44319-026-00786-5 (PMC13354796; doi:10.1038/s44319-026-00786-5)

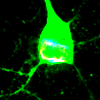

Supplement: Supplementary file 4 — Source data Fig. 2 [file 44319_2026_786_MOESM4_ESM.zip › Figure 2 Source Data/2D/Merge_DN only.tif]

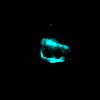

Supplement: Supplementary file 4 — Source data Fig. 2 [file 44319_2026_786_MOESM4_ESM.zip › Figure 2 Source Data/2D/HA_DN only.tif]

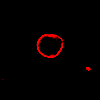

Supplement: Supplementary file 4 — Source data Fig. 2 [file 44319_2026_786_MOESM4_ESM.zip › Figure 2 Source Data/2D/Lamin B1_1+DN.tif]

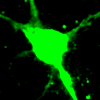

Supplement: Supplementary file 4 — Source data Fig. 2 [file 44319_2026_786_MOESM4_ESM.zip › Figure 2 Source Data/2D/Venus_1+DN.tif]

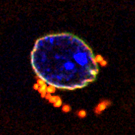

Supplement: Supplementary file 4 — Source data Fig. 2 [file 44319_2026_786_MOESM4_ESM.zip › Figure 2 Source Data/2L/Merge_20M + Sun1.tif]

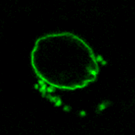

Supplement: Supplementary file 4 — Source data Fig. 2 [file 44319_2026_786_MOESM4_ESM.zip › Figure 2 Source Data/2L/HA_20M + Sun1.tif]

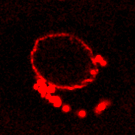

Supplement: Supplementary file 4 — Source data Fig. 2 [file 44319_2026_786_MOESM4_ESM.zip › Figure 2 Source Data/2L/Lamin B1_20M + Sun1.tif]

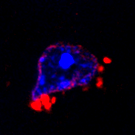

Supplement: Supplementary file 4 — Source data Fig. 2 [file 44319_2026_786_MOESM4_ESM.zip › Figure 2 Source Data/2L/Merge_20M NV.tif]

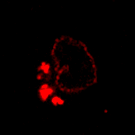

Supplement: Supplementary file 4 — Source data Fig. 2 [file 44319_2026_786_MOESM4_ESM.zip › Figure 2 Source Data/2L/Lamin B1_20M Control.tif]

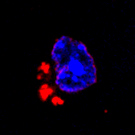

Supplement: Supplementary file 4 — Source data Fig. 2 [file 44319_2026_786_MOESM4_ESM.zip › Figure 2 Source Data/2L/Merge_20M Control.tif]

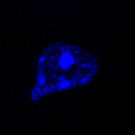

Supplement: Supplementary file 4 — Source data Fig. 2 [file 44319_2026_786_MOESM4_ESM.zip › Figure 2 Source Data/2L/Hoechst_20M NV.tif]

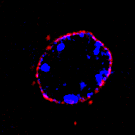

Supplement: Supplementary file 4 — Source data Fig. 2 [file 44319_2026_786_MOESM4_ESM.zip › Figure 2 Source Data/2L/Merge_3M NV.tif]

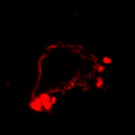

Supplement: Supplementary file 4 — Source data Fig. 2 [file 44319_2026_786_MOESM4_ESM.zip › Figure 2 Source Data/2L/Lamin B1_20M NV.tif]

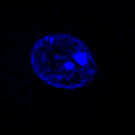

Supplement: Supplementary file 4 — Source data Fig. 2 [file 44319_2026_786_MOESM4_ESM.zip › Figure 2 Source Data/2L/Hoechst_20M + Sun1.tif]

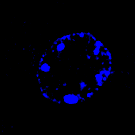

Supplement: Supplementary file 4 — Source data Fig. 2 [file 44319_2026_786_MOESM4_ESM.zip › Figure 2 Source Data/2L/Hoechst_3M NV.tif]

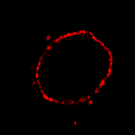

Supplement: Supplementary file 4 — Source data Fig. 2 [file 44319_2026_786_MOESM4_ESM.zip › Figure 2 Source Data/2L/Lamin B1_3M NV.tif]

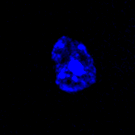

Supplement: Supplementary file 4 — Source data Fig. 2 [file 44319_2026_786_MOESM4_ESM.zip › Figure 2 Source Data/2L/Hoechst_20M Control.tif]

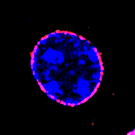

Supplement: Supplementary file 4 — Source data Fig. 2 [file 44319_2026_786_MOESM4_ESM.zip › Figure 2 Source Data/2A/Merge_3M Control.tif]

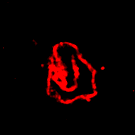

Supplement: Supplementary file 4 — Source data Fig. 2 [file 44319_2026_786_MOESM4_ESM.zip › Figure 2 Source Data/2A/Lamin B1_3M LINC-DN.tif]

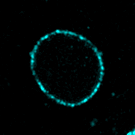

Supplement: Supplementary file 4 — Source data Fig. 2 [file 44319_2026_786_MOESM4_ESM.zip › Figure 2 Source Data/2A/HA_3M Control.tif]

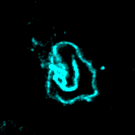

Supplement: Supplementary file 4 — Source data Fig. 2 [file 44319_2026_786_MOESM4_ESM.zip › Figure 2 Source Data/2A/HA_3M LINC-DN.tif]

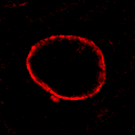

Supplement: Supplementary file 4 — Source data Fig. 2 [file 44319_2026_786_MOESM4_ESM.zip › Figure 2 Source Data/2A/Lamin B1_3M NV.tif]

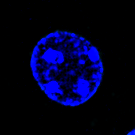

Supplement: Supplementary file 4 — Source data Fig. 2 [file 44319_2026_786_MOESM4_ESM.zip › Figure 2 Source Data/2A/Hoechst_3M Control.tif]

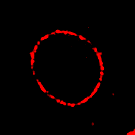

Supplement: Supplementary file 4 — Source data Fig. 2 [file 44319_2026_786_MOESM4_ESM.zip › Figure 2 Source Data/2A/Lamin B1_3M Control.tif]

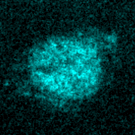

Supplement: Supplementary file 4 — Source data Fig. 2 [file 44319_2026_786_MOESM4_ESM.zip › Figure 2 Source Data/2A/HA_3M NV.tif]

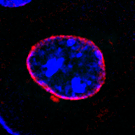

Supplement: Supplementary file 4 — Source data Fig. 2 [file 44319_2026_786_MOESM4_ESM.zip › Figure 2 Source Data/2A/Merge_3M NV.tif]

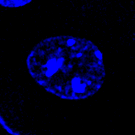

Supplement: Supplementary file 4 — Source data Fig. 2 [file 44319_2026_786_MOESM4_ESM.zip › Figure 2 Source Data/2A/Hoechst_3M NV.tif]

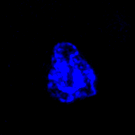

Supplement: Supplementary file 4 — Source data Fig. 2 [file 44319_2026_786_MOESM4_ESM.zip › Figure 2 Source Data/2A/Hoechst_3M LINC-DN.tif]

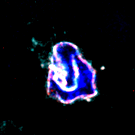

Supplement: Supplementary file 4 — Source data Fig. 2 [file 44319_2026_786_MOESM4_ESM.zip › Figure 2 Source Data/2A/Merge_3M LINC-DN.tif]

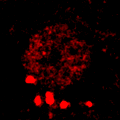

Supplement: Supplementary file 4 — Source data Fig. 2 [file 44319_2026_786_MOESM4_ESM.zip › Figure 2 Source Data/2F/NeuN_20M.tif]

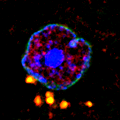

Supplement: Supplementary file 4 — Source data Fig. 2 [file 44319_2026_786_MOESM4_ESM.zip › Figure 2 Source Data/2F/Merge_20M.tif]

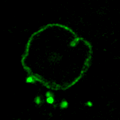

Supplement: Supplementary file 4 — Source data Fig. 2 [file 44319_2026_786_MOESM4_ESM.zip › Figure 2 Source Data/2F/Lamin B1_20M.tif]

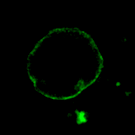

Supplement: Supplementary file 4 — Source data Fig. 2 [file 44319_2026_786_MOESM4_ESM.zip › Figure 2 Source Data/2F/Lamin B1_12M.tif]

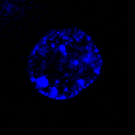

Supplement: Supplementary file 4 — Source data Fig. 2 [file 44319_2026_786_MOESM4_ESM.zip › Figure 2 Source Data/2F/Hoechst_12M.tif]

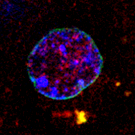

Supplement: Supplementary file 4 — Source data Fig. 2 [file 44319_2026_786_MOESM4_ESM.zip › Figure 2 Source Data/2F/Merge_12M.tif]

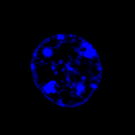

Supplement: Supplementary file 4 — Source data Fig. 2 [file 44319_2026_786_MOESM4_ESM.zip › Figure 2 Source Data/2F/Hoechst_3M.tif]

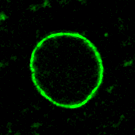

Supplement: Supplementary file 4 — Source data Fig. 2 [file 44319_2026_786_MOESM4_ESM.zip › Figure 2 Source Data/2F/Lamin B1_3M.tif]

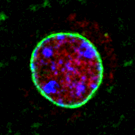

Supplement: Supplementary file 4 — Source data Fig. 2 [file 44319_2026_786_MOESM4_ESM.zip › Figure 2 Source Data/2F/Merge_3M.tif]

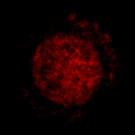

Supplement: Supplementary file 4 — Source data Fig. 2 [file 44319_2026_786_MOESM4_ESM.zip › Figure 2 Source Data/2F/NeuN_3M.tif]

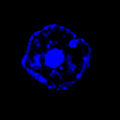

Supplement: Supplementary file 4 — Source data Fig. 2 [file 44319_2026_786_MOESM4_ESM.zip › Figure 2 Source Data/2F/Hoechst_20M.tif]

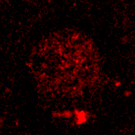

Supplement: Supplementary file 4 — Source data Fig. 2 [file 44319_2026_786_MOESM4_ESM.zip › Figure 2 Source Data/2F/NeuN_12M.tif]

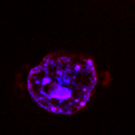

Supplement: Supplementary file 5 — Source data Fig. 3 [file 44319_2026_786_MOESM5_ESM.zip › Figure 3 Source Data/3A/Merge_20M Control.tif]

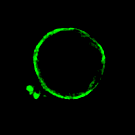

Supplement: Supplementary file 5 — Source data Fig. 3 [file 44319_2026_786_MOESM5_ESM.zip › Figure 3 Source Data/3A/HA_20M + Sun1.tif]

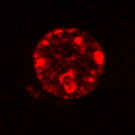

Supplement: Supplementary file 5 — Source data Fig. 3 [file 44319_2026_786_MOESM5_ESM.zip › Figure 3 Source Data/3A/H3K9me3_20M + Sun1.tif]

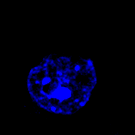

Supplement: Supplementary file 5 — Source data Fig. 3 [file 44319_2026_786_MOESM5_ESM.zip › Figure 3 Source Data/3A/Hoechst_20M Control.tif]

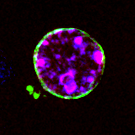

Supplement: Supplementary file 5 — Source data Fig. 3 [file 44319_2026_786_MOESM5_ESM.zip › Figure 3 Source Data/3A/Merge_20M + Sun1.tif]

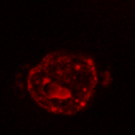

Supplement: Supplementary file 5 — Source data Fig. 3 [file 44319_2026_786_MOESM5_ESM.zip › Figure 3 Source Data/3A/H3K9me3_20M Control.tif]

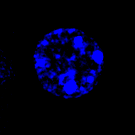

Supplement: Supplementary file 5 — Source data Fig. 3 [file 44319_2026_786_MOESM5_ESM.zip › Figure 3 Source Data/3A/Hoechst_20M + Sun1.tif]

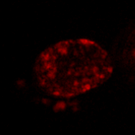

Supplement: Supplementary file 5 — Source data Fig. 3 [file 44319_2026_786_MOESM5_ESM.zip › Figure 3 Source Data/3A/H3K9me3_20M NV.tif]

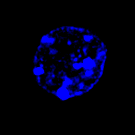

Supplement: Supplementary file 5 — Source data Fig. 3 [file 44319_2026_786_MOESM5_ESM.zip › Figure 3 Source Data/3A/Hoechst_3M NV.tif]

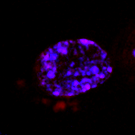

Supplement: Supplementary file 5 — Source data Fig. 3 [file 44319_2026_786_MOESM5_ESM.zip › Figure 3 Source Data/3A/Merge_20M NV.tif]

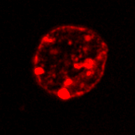

Supplement: Supplementary file 5 — Source data Fig. 3 [file 44319_2026_786_MOESM5_ESM.zip › Figure 3 Source Data/3A/H3K9me3_3M NV.tif]

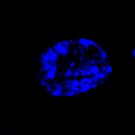

Supplement: Supplementary file 5 — Source data Fig. 3 [file 44319_2026_786_MOESM5_ESM.zip › Figure 3 Source Data/3A/Hoechst_20M NV.tif]

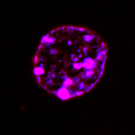

Supplement: Supplementary file 5 — Source data Fig. 3 [file 44319_2026_786_MOESM5_ESM.zip › Figure 3 Source Data/3A/Merge_3M NV.tif]

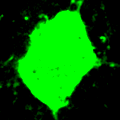

Supplement: Supplementary file 5 — Source data Fig. 3 [file 44319_2026_786_MOESM5_ESM.zip › Figure 3 Source Data/3C/Venus_LINC-DN.tif]

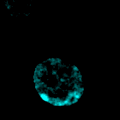

Supplement: Supplementary file 5 — Source data Fig. 3 [file 44319_2026_786_MOESM5_ESM.zip › Figure 3 Source Data/3C/HA_Control.tif]

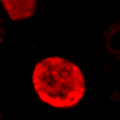

Supplement: Supplementary file 5 — Source data Fig. 3 [file 44319_2026_786_MOESM5_ESM.zip › Figure 3 Source Data/3C/H3K9me3_Control.tif]

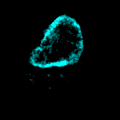

Supplement: Supplementary file 5 — Source data Fig. 3 [file 44319_2026_786_MOESM5_ESM.zip › Figure 3 Source Data/3C/HA_LINC-DN.tif]

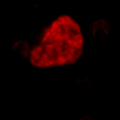

Supplement: Supplementary file 5 — Source data Fig. 3 [file 44319_2026_786_MOESM5_ESM.zip › Figure 3 Source Data/3C/H3K9me3_LINC-DN.tif]

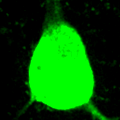

Supplement: Supplementary file 5 — Source data Fig. 3 [file 44319_2026_786_MOESM5_ESM.zip › Figure 3 Source Data/3C/Venus_Control.tif]

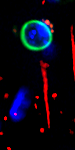

Supplement: Supplementary file 6 — Source data Fig. 4 [file 44319_2026_786_MOESM6_ESM.zip › Figure 4 Source Data/4C/Merge_20M + Sun1.tif]

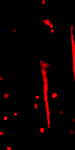

Supplement: Supplementary file 6 — Source data Fig. 4 [file 44319_2026_786_MOESM6_ESM.zip › Figure 4 Source Data/4C/Nav1.6_20M + Sun1.tif]

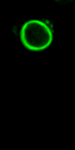

Supplement: Supplementary file 6 — Source data Fig. 4 [file 44319_2026_786_MOESM6_ESM.zip › Figure 4 Source Data/4C/HA_20M + Sun1.tif]

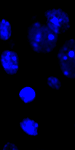

Supplement: Supplementary file 6 — Source data Fig. 4 [file 44319_2026_786_MOESM6_ESM.zip › Figure 4 Source Data/4C/Hoechst_20M Control.tif]

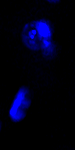

Supplement: Supplementary file 6 — Source data Fig. 4 [file 44319_2026_786_MOESM6_ESM.zip › Figure 4 Source Data/4C/Hoechst_20M + Sun1.tif]

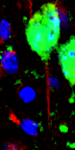

Supplement: Supplementary file 6 — Source data Fig. 4 [file 44319_2026_786_MOESM6_ESM.zip › Figure 4 Source Data/4C/Merge_20M Control.tif]

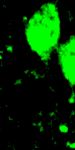

Supplement: Supplementary file 6 — Source data Fig. 4 [file 44319_2026_786_MOESM6_ESM.zip › Figure 4 Source Data/4C/Venus_20M Control.tif]

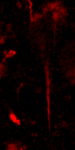

Supplement: Supplementary file 6 — Source data Fig. 4 [file 44319_2026_786_MOESM6_ESM.zip › Figure 4 Source Data/4C/Nav1.6_20M Control.tif]

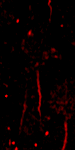

Supplement: Supplementary file 6 — Source data Fig. 4 [file 44319_2026_786_MOESM6_ESM.zip › Figure 4 Source Data/4C/Nav1.6_20M NV.tif]

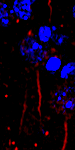

Supplement: Supplementary file 6 — Source data Fig. 4 [file 44319_2026_786_MOESM6_ESM.zip › Figure 4 Source Data/4C/Merge_20M NV.tif]

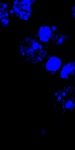

Supplement: Supplementary file 6 — Source data Fig. 4 [file 44319_2026_786_MOESM6_ESM.zip › Figure 4 Source Data/4C/Hoechst_20M NV.tif]

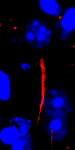

Supplement: Supplementary file 6 — Source data Fig. 4 [file 44319_2026_786_MOESM6_ESM.zip › Figure 4 Source Data/4C/Merge_3M NV.tif]

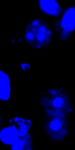

Supplement: Supplementary file 6 — Source data Fig. 4 [file 44319_2026_786_MOESM6_ESM.zip › Figure 4 Source Data/4C/Hoechst_3M NV.tif]

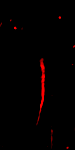

Supplement: Supplementary file 6 — Source data Fig. 4 [file 44319_2026_786_MOESM6_ESM.zip › Figure 4 Source Data/4C/Nav1.6_3M NV.tif]

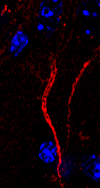

Supplement: Supplementary file 6 — Source data Fig. 4 [file 44319_2026_786_MOESM6_ESM.zip › Figure 4 Source Data/4G/Merge_3M HP.tif]

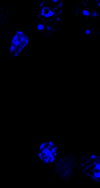

Supplement: Supplementary file 6 — Source data Fig. 4 [file 44319_2026_786_MOESM6_ESM.zip › Figure 4 Source Data/4G/Hoechst_3M HP.tif]

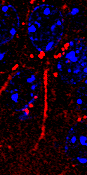

Supplement: Supplementary file 6 — Source data Fig. 4 [file 44319_2026_786_MOESM6_ESM.zip › Figure 4 Source Data/4G/Merge_20M HP.tif]

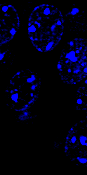

Supplement: Supplementary file 6 — Source data Fig. 4 [file 44319_2026_786_MOESM6_ESM.zip › Figure 4 Source Data/4G/Hoechst_20M HP.tif]

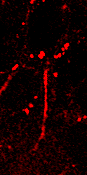

Supplement: Supplementary file 6 — Source data Fig. 4 [file 44319_2026_786_MOESM6_ESM.zip › Figure 4 Source Data/4G/Ankyrin-G_20M HP.tif]

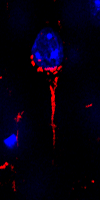

Supplement: Supplementary file 6 — Source data Fig. 4 [file 44319_2026_786_MOESM6_ESM.zip › Figure 4 Source Data/4G/Merge_20M MC.tif]

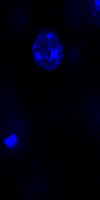

Supplement: Supplementary file 6 — Source data Fig. 4 [file 44319_2026_786_MOESM6_ESM.zip › Figure 4 Source Data/4G/Hoechst_20M MC.tif]

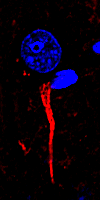

Supplement: Supplementary file 6 — Source data Fig. 4 [file 44319_2026_786_MOESM6_ESM.zip › Figure 4 Source Data/4G/Merge_3M MC.tif]

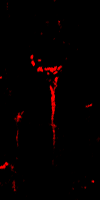

Supplement: Supplementary file 6 — Source data Fig. 4 [file 44319_2026_786_MOESM6_ESM.zip › Figure 4 Source Data/4G/Ankyrin-G_20M MC.tif]

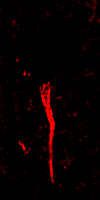

Supplement: Supplementary file 6 — Source data Fig. 4 [file 44319_2026_786_MOESM6_ESM.zip › Figure 4 Source Data/4G/Ankyrin-G_3M MC.tif]

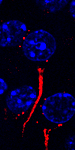

Supplement: Supplementary file 6 — Source data Fig. 4 [file 44319_2026_786_MOESM6_ESM.zip › Figure 4 Source Data/4G/Merge_3M SSC.tif]

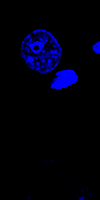

Supplement: Supplementary file 6 — Source data Fig. 4 [file 44319_2026_786_MOESM6_ESM.zip › Figure 4 Source Data/4G/Hoechst_3M MC.tif]

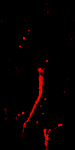

Supplement: Supplementary file 6 — Source data Fig. 4 [file 44319_2026_786_MOESM6_ESM.zip › Figure 4 Source Data/4G/Ankyrin-G_3M SSC.tif]

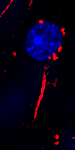

Supplement: Supplementary file 6 — Source data Fig. 4 [file 44319_2026_786_MOESM6_ESM.zip › Figure 4 Source Data/4G/Merge_20M SSC.tif]

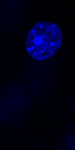

Supplement: Supplementary file 6 — Source data Fig. 4 [file 44319_2026_786_MOESM6_ESM.zip › Figure 4 Source Data/4G/Hoechst_20M SSC.tif]

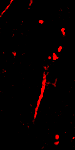

Supplement: Supplementary file 6 — Source data Fig. 4 [file 44319_2026_786_MOESM6_ESM.zip › Figure 4 Source Data/4G/Ankyrin-G_20M SSC.tif]

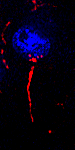

Supplement: Supplementary file 6 — Source data Fig. 4 [file 44319_2026_786_MOESM6_ESM.zip › Figure 4 Source Data/4G/Merge_20M PFC.tif]

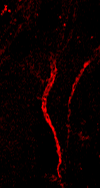

Supplement: Supplementary file 6 — Source data Fig. 4 [file 44319_2026_786_MOESM6_ESM.zip › Figure 4 Source Data/4G/Ankyrin-G_3M HP.tif]

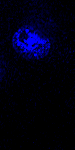

Supplement: Supplementary file 6 — Source data Fig. 4 [file 44319_2026_786_MOESM6_ESM.zip › Figure 4 Source Data/4G/Hoechst_20M PFC.tif]

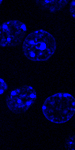

Supplement: Supplementary file 6 — Source data Fig. 4 [file 44319_2026_786_MOESM6_ESM.zip › Figure 4 Source Data/4G/Hoechst_3M SSC.tif]

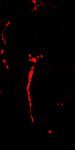

Supplement: Supplementary file 6 — Source data Fig. 4 [file 44319_2026_786_MOESM6_ESM.zip › Figure 4 Source Data/4G/Ankyrin-G_20M PFC.tif]

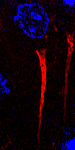

Supplement: Supplementary file 6 — Source data Fig. 4 [file 44319_2026_786_MOESM6_ESM.zip › Figure 4 Source Data/4G/Merge_3M PFC.tif]

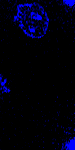

Supplement: Supplementary file 6 — Source data Fig. 4 [file 44319_2026_786_MOESM6_ESM.zip › Figure 4 Source Data/4G/Hoechst_3M PFC.tif]

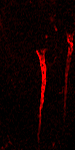

Supplement: Supplementary file 6 — Source data Fig. 4 [file 44319_2026_786_MOESM6_ESM.zip › Figure 4 Source Data/4G/Ankyrin-G_3M PFC.tif]

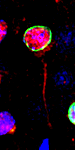

Supplement: Supplementary file 6 — Source data Fig. 4 [file 44319_2026_786_MOESM6_ESM.zip › Figure 4 Source Data/4E/Merge_20M + Sun1.tif]

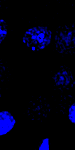

Supplement: Supplementary file 6 — Source data Fig. 4 [file 44319_2026_786_MOESM6_ESM.zip › Figure 4 Source Data/4E/Hoechst_20M + Sun1.tif]
