# Supplementary figures and images for: Age-related decline in nuclear envelope LINC complex drives neuronal aging via axon initial segment dysfunction (part 3 of 9)
Source: EMBO Rep. 2026 May 22;27(13):3788–825. doi: 10.1038/s44319-026-00786-5 (PMC13354796; doi:10.1038/s44319-026-00786-5)

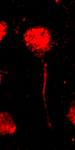

Supplement: Supplementary file 6 — Source data Fig. 4 [file 44319_2026_786_MOESM6_ESM.zip › Figure 4 Source Data/4E/Kv7.3_20M + Sun1.tif]

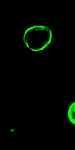

Supplement: Supplementary file 6 — Source data Fig. 4 [file 44319_2026_786_MOESM6_ESM.zip › Figure 4 Source Data/4E/HA_20M + Sun1.tif]

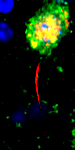

Supplement: Supplementary file 6 — Source data Fig. 4 [file 44319_2026_786_MOESM6_ESM.zip › Figure 4 Source Data/4E/Merge_20M Control.tif]

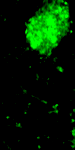

Supplement: Supplementary file 6 — Source data Fig. 4 [file 44319_2026_786_MOESM6_ESM.zip › Figure 4 Source Data/4E/Venus_20M Control.tif]

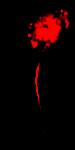

Supplement: Supplementary file 6 — Source data Fig. 4 [file 44319_2026_786_MOESM6_ESM.zip › Figure 4 Source Data/4E/Kv7.3_20M Control.tif]

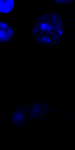

Supplement: Supplementary file 6 — Source data Fig. 4 [file 44319_2026_786_MOESM6_ESM.zip › Figure 4 Source Data/4E/Hoechst_20M Control.tif]

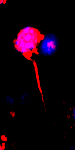

Supplement: Supplementary file 6 — Source data Fig. 4 [file 44319_2026_786_MOESM6_ESM.zip › Figure 4 Source Data/4E/Merge_20M NV.tif]

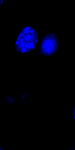

Supplement: Supplementary file 6 — Source data Fig. 4 [file 44319_2026_786_MOESM6_ESM.zip › Figure 4 Source Data/4E/Hoechst_20M NV.tif]

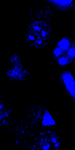

Supplement: Supplementary file 6 — Source data Fig. 4 [file 44319_2026_786_MOESM6_ESM.zip › Figure 4 Source Data/4E/Hoechst_3M NV.tif]

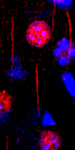

Supplement: Supplementary file 6 — Source data Fig. 4 [file 44319_2026_786_MOESM6_ESM.zip › Figure 4 Source Data/4E/Merge_3M NV.tif]

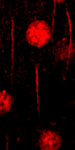

Supplement: Supplementary file 6 — Source data Fig. 4 [file 44319_2026_786_MOESM6_ESM.zip › Figure 4 Source Data/4E/Kv7.3_3M NV.tif]

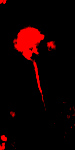

Supplement: Supplementary file 6 — Source data Fig. 4 [file 44319_2026_786_MOESM6_ESM.zip › Figure 4 Source Data/4E/Kv7.3_20M NV.tif]

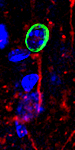

Supplement: Supplementary file 6 — Source data Fig. 4 [file 44319_2026_786_MOESM6_ESM.zip › Figure 4 Source Data/4A/Merge_20M + Sun1.tif]

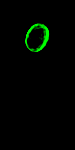

Supplement: Supplementary file 6 — Source data Fig. 4 [file 44319_2026_786_MOESM6_ESM.zip › Figure 4 Source Data/4A/HA_20M + Sun1.tif]

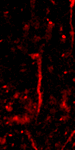

Supplement: Supplementary file 6 — Source data Fig. 4 [file 44319_2026_786_MOESM6_ESM.zip › Figure 4 Source Data/4A/Nav1.2_20M + Sun1.tif]

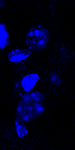

Supplement: Supplementary file 6 — Source data Fig. 4 [file 44319_2026_786_MOESM6_ESM.zip › Figure 4 Source Data/4A/Hoechst_20M + Sun1.tif]

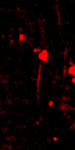

Supplement: Supplementary file 6 — Source data Fig. 4 [file 44319_2026_786_MOESM6_ESM.zip › Figure 4 Source Data/4A/Nav1.2_20M Control.tif]

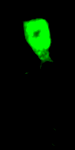

Supplement: Supplementary file 6 — Source data Fig. 4 [file 44319_2026_786_MOESM6_ESM.zip › Figure 4 Source Data/4A/Venus_20M Control.tif]

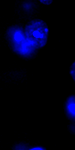

Supplement: Supplementary file 6 — Source data Fig. 4 [file 44319_2026_786_MOESM6_ESM.zip › Figure 4 Source Data/4A/Hoechst_20M Control.tif]

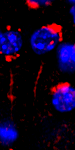

Supplement: Supplementary file 6 — Source data Fig. 4 [file 44319_2026_786_MOESM6_ESM.zip › Figure 4 Source Data/4A/Merge_20M NV.tif]

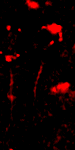

Supplement: Supplementary file 6 — Source data Fig. 4 [file 44319_2026_786_MOESM6_ESM.zip › Figure 4 Source Data/4A/Nav1.2_20M NV.tif]

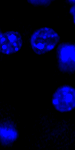

Supplement: Supplementary file 6 — Source data Fig. 4 [file 44319_2026_786_MOESM6_ESM.zip › Figure 4 Source Data/4A/Hoechst_20M NV.tif]

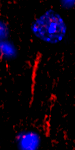

Supplement: Supplementary file 6 — Source data Fig. 4 [file 44319_2026_786_MOESM6_ESM.zip › Figure 4 Source Data/4A/Merge_3M NV.tif]

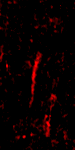

Supplement: Supplementary file 6 — Source data Fig. 4 [file 44319_2026_786_MOESM6_ESM.zip › Figure 4 Source Data/4A/Nav1.2_3M NV.tif]

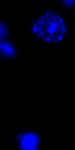

Supplement: Supplementary file 6 — Source data Fig. 4 [file 44319_2026_786_MOESM6_ESM.zip › Figure 4 Source Data/4A/Hoechst_3M NV.tif]

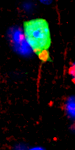

Supplement: Supplementary file 6 — Source data Fig. 4 [file 44319_2026_786_MOESM6_ESM.zip › Figure 4 Source Data/4A/Merge_20M Control.tif]

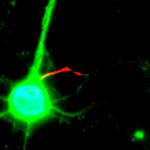

Supplement: Supplementary file 7 — Source data Fig. 5 [file 44319_2026_786_MOESM7_ESM.zip › Figure 5 Source Data/5G/Merge_LINC-DN.tif]

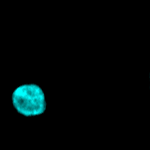

Supplement: Supplementary file 7 — Source data Fig. 5 [file 44319_2026_786_MOESM7_ESM.zip › Figure 5 Source Data/5G/HA_LINC-DN.tif]

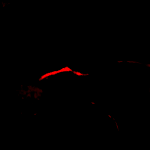

Supplement: Supplementary file 7 — Source data Fig. 5 [file 44319_2026_786_MOESM7_ESM.zip › Figure 5 Source Data/5G/Ankyrin-G_LINC-DN.tif]

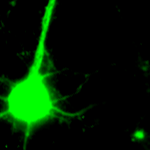

Supplement: Supplementary file 7 — Source data Fig. 5 [file 44319_2026_786_MOESM7_ESM.zip › Figure 5 Source Data/5G/Venus_LINC-DN.tif]

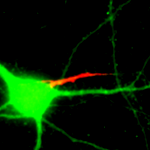

Supplement: Supplementary file 7 — Source data Fig. 5 [file 44319_2026_786_MOESM7_ESM.zip › Figure 5 Source Data/5G/Merge_Control.tif]

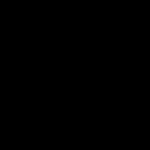

Supplement: Supplementary file 7 — Source data Fig. 5 [file 44319_2026_786_MOESM7_ESM.zip › Figure 5 Source Data/5G/HA_Control.tif]

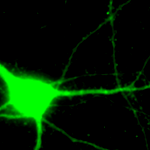

Supplement: Supplementary file 7 — Source data Fig. 5 [file 44319_2026_786_MOESM7_ESM.zip › Figure 5 Source Data/5G/Venus_Control.tif]

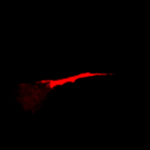

Supplement: Supplementary file 7 — Source data Fig. 5 [file 44319_2026_786_MOESM7_ESM.zip › Figure 5 Source Data/5G/Ankyrin-G_Control.tif]

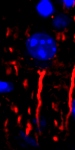

Supplement: Supplementary file 7 — Source data Fig. 5 [file 44319_2026_786_MOESM7_ESM.zip › Figure 5 Source Data/5P/Merge_3M NV.tif]

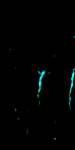

Supplement: Supplementary file 7 — Source data Fig. 5 [file 44319_2026_786_MOESM7_ESM.zip › Figure 5 Source Data/5P/HA_3M NV.tif]

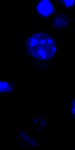

Supplement: Supplementary file 7 — Source data Fig. 5 [file 44319_2026_786_MOESM7_ESM.zip › Figure 5 Source Data/5P/Hoechst_3M NV.tif]

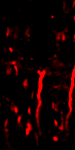

Supplement: Supplementary file 7 — Source data Fig. 5 [file 44319_2026_786_MOESM7_ESM.zip › Figure 5 Source Data/5P/Ankyrin-G_3M NV.tif]

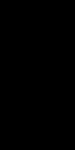

Supplement: Supplementary file 7 — Source data Fig. 5 [file 44319_2026_786_MOESM7_ESM.zip › Figure 5 Source Data/5P/Venus_3M NV.tif]

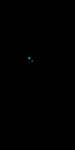

Supplement: Supplementary file 7 — Source data Fig. 5 [file 44319_2026_786_MOESM7_ESM.zip › Figure 5 Source Data/5P/HA_3M Control.tif]

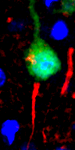

Supplement: Supplementary file 7 — Source data Fig. 5 [file 44319_2026_786_MOESM7_ESM.zip › Figure 5 Source Data/5P/Merge_3M Control.tif]

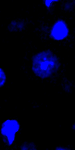

Supplement: Supplementary file 7 — Source data Fig. 5 [file 44319_2026_786_MOESM7_ESM.zip › Figure 5 Source Data/5P/Hoechst_3M Control.tif]

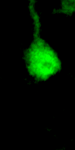

Supplement: Supplementary file 7 — Source data Fig. 5 [file 44319_2026_786_MOESM7_ESM.zip › Figure 5 Source Data/5P/Venus_3M Control.tif]

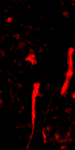

Supplement: Supplementary file 7 — Source data Fig. 5 [file 44319_2026_786_MOESM7_ESM.zip › Figure 5 Source Data/5P/Ankyrin-G_3M Control.tif]

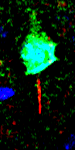

Supplement: Supplementary file 7 — Source data Fig. 5 [file 44319_2026_786_MOESM7_ESM.zip › Figure 5 Source Data/5P/Merge_3M LINC-DN.tif]

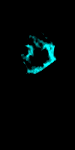

Supplement: Supplementary file 7 — Source data Fig. 5 [file 44319_2026_786_MOESM7_ESM.zip › Figure 5 Source Data/5P/HA_3M LINC-DN.tif]

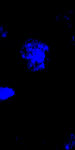

Supplement: Supplementary file 7 — Source data Fig. 5 [file 44319_2026_786_MOESM7_ESM.zip › Figure 5 Source Data/5P/Hoechst_3M LINC-DN.tif]

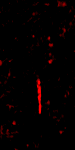

Supplement: Supplementary file 7 — Source data Fig. 5 [file 44319_2026_786_MOESM7_ESM.zip › Figure 5 Source Data/5P/Ankyrin-G_3M LINC-DN.tif]

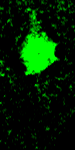

Supplement: Supplementary file 7 — Source data Fig. 5 [file 44319_2026_786_MOESM7_ESM.zip › Figure 5 Source Data/5P/Venus_3M LINC-DN.tif]

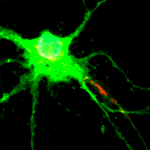

Supplement: Supplementary file 7 — Source data Fig. 5 [file 44319_2026_786_MOESM7_ESM.zip › Figure 5 Source Data/5J/Merge_LINC-DN.tif]

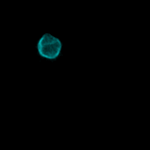

Supplement: Supplementary file 7 — Source data Fig. 5 [file 44319_2026_786_MOESM7_ESM.zip › Figure 5 Source Data/5J/HA_LINC-DN.tif]

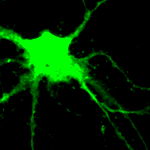

Supplement: Supplementary file 7 — Source data Fig. 5 [file 44319_2026_786_MOESM7_ESM.zip › Figure 5 Source Data/5J/Venus_LINC-DN.tif]

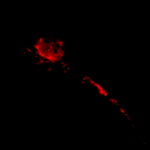

Supplement: Supplementary file 7 — Source data Fig. 5 [file 44319_2026_786_MOESM7_ESM.zip › Figure 5 Source Data/5J/pan-Nav_LINC-DN.tif]

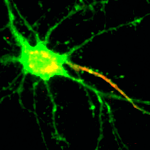

Supplement: Supplementary file 7 — Source data Fig. 5 [file 44319_2026_786_MOESM7_ESM.zip › Figure 5 Source Data/5J/Merge_Control.tif]

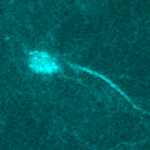

Supplement: Supplementary file 7 — Source data Fig. 5 [file 44319_2026_786_MOESM7_ESM.zip › Figure 5 Source Data/5J/HA_Control.tif]

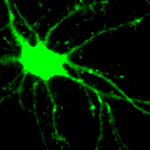

Supplement: Supplementary file 7 — Source data Fig. 5 [file 44319_2026_786_MOESM7_ESM.zip › Figure 5 Source Data/5J/Venus_Control.tif]

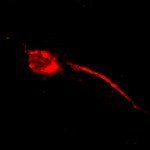

Supplement: Supplementary file 7 — Source data Fig. 5 [file 44319_2026_786_MOESM7_ESM.zip › Figure 5 Source Data/5J/pan-Nav_Control.tif]

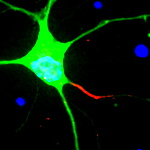

Supplement: Supplementary file 7 — Source data Fig. 5 [file 44319_2026_786_MOESM7_ESM.zip › Figure 5 Source Data/5A/Merge_LINC-DN.tif]

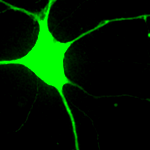

Supplement: Supplementary file 7 — Source data Fig. 5 [file 44319_2026_786_MOESM7_ESM.zip › Figure 5 Source Data/5A/Venus_LINC-DN.tif]

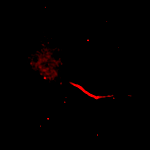

Supplement: Supplementary file 7 — Source data Fig. 5 [file 44319_2026_786_MOESM7_ESM.zip › Figure 5 Source Data/5A/Ankyrin-G_LINC-DN.tif]

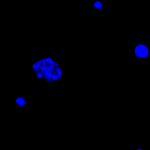

Supplement: Supplementary file 7 — Source data Fig. 5 [file 44319_2026_786_MOESM7_ESM.zip › Figure 5 Source Data/5A/Hoechst_LINC-DN.tif]

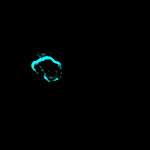

Supplement: Supplementary file 7 — Source data Fig. 5 [file 44319_2026_786_MOESM7_ESM.zip › Figure 5 Source Data/5A/HA_LINC-DN.tif]

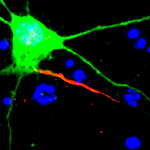

Supplement: Supplementary file 7 — Source data Fig. 5 [file 44319_2026_786_MOESM7_ESM.zip › Figure 5 Source Data/5A/Merge_Control.tif]

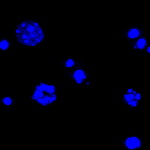

Supplement: Supplementary file 7 — Source data Fig. 5 [file 44319_2026_786_MOESM7_ESM.zip › Figure 5 Source Data/5A/Hoechst_Control.tif]

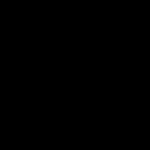

Supplement: Supplementary file 7 — Source data Fig. 5 [file 44319_2026_786_MOESM7_ESM.zip › Figure 5 Source Data/5A/HA_Control.tif]

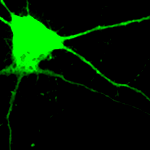

Supplement: Supplementary file 7 — Source data Fig. 5 [file 44319_2026_786_MOESM7_ESM.zip › Figure 5 Source Data/5A/Venus_Control.tif]

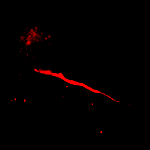

Supplement: Supplementary file 7 — Source data Fig. 5 [file 44319_2026_786_MOESM7_ESM.zip › Figure 5 Source Data/5A/Ankyrin-G_Control.tif]

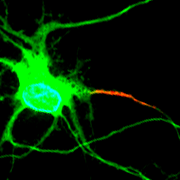

Supplement: Supplementary file 7 — Source data Fig. 5 [file 44319_2026_786_MOESM7_ESM.zip › Figure 5 Source Data/5D/Merge_LINC-DN KCl.tif]

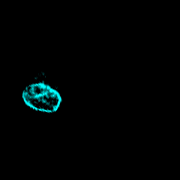

Supplement: Supplementary file 7 — Source data Fig. 5 [file 44319_2026_786_MOESM7_ESM.zip › Figure 5 Source Data/5D/HA_LINC-DN KCl.tif]

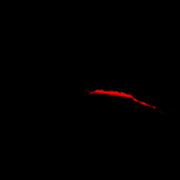

Supplement: Supplementary file 7 — Source data Fig. 5 [file 44319_2026_786_MOESM7_ESM.zip › Figure 5 Source Data/5D/Ankyrin-G_LINC-DN KCl.tif]

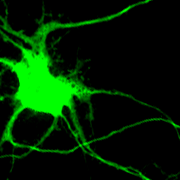

Supplement: Supplementary file 7 — Source data Fig. 5 [file 44319_2026_786_MOESM7_ESM.zip › Figure 5 Source Data/5D/Venus_LINC-DN KCl.tif]

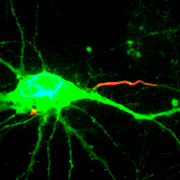

Supplement: Supplementary file 7 — Source data Fig. 5 [file 44319_2026_786_MOESM7_ESM.zip › Figure 5 Source Data/5D/Merge_LINC-DN NaCl.tif]

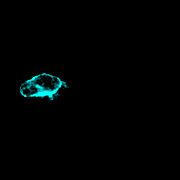

Supplement: Supplementary file 7 — Source data Fig. 5 [file 44319_2026_786_MOESM7_ESM.zip › Figure 5 Source Data/5D/HA_LINC-DN NaCl.tif]

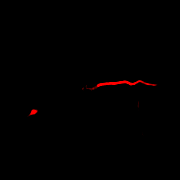

Supplement: Supplementary file 7 — Source data Fig. 5 [file 44319_2026_786_MOESM7_ESM.zip › Figure 5 Source Data/5D/Ankyrin-G_LINC-DN NaCl.tif]

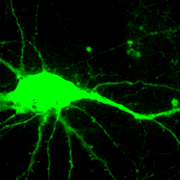

Supplement: Supplementary file 7 — Source data Fig. 5 [file 44319_2026_786_MOESM7_ESM.zip › Figure 5 Source Data/5D/Venus_LINC-DN NaCl.tif]

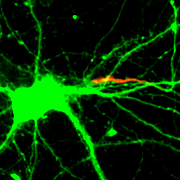

Supplement: Supplementary file 7 — Source data Fig. 5 [file 44319_2026_786_MOESM7_ESM.zip › Figure 5 Source Data/5D/Merge_Control KCl.tif]

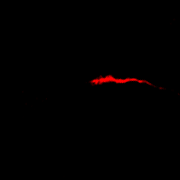

Supplement: Supplementary file 7 — Source data Fig. 5 [file 44319_2026_786_MOESM7_ESM.zip › Figure 5 Source Data/5D/Ankyrin-G_Control KCl.tif]

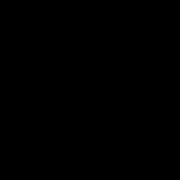

Supplement: Supplementary file 7 — Source data Fig. 5 [file 44319_2026_786_MOESM7_ESM.zip › Figure 5 Source Data/5D/HA_Control KCl.tif]

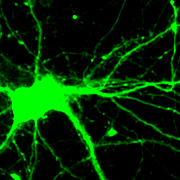

Supplement: Supplementary file 7 — Source data Fig. 5 [file 44319_2026_786_MOESM7_ESM.zip › Figure 5 Source Data/5D/Venus_Control KCl.tif]

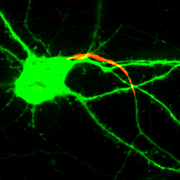

Supplement: Supplementary file 7 — Source data Fig. 5 [file 44319_2026_786_MOESM7_ESM.zip › Figure 5 Source Data/5D/Merge_Control NaCl.tif]

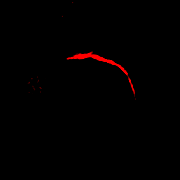

Supplement: Supplementary file 7 — Source data Fig. 5 [file 44319_2026_786_MOESM7_ESM.zip › Figure 5 Source Data/5D/Ankyrin-G_Control NaCl.tif]

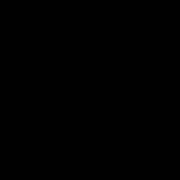

Supplement: Supplementary file 7 — Source data Fig. 5 [file 44319_2026_786_MOESM7_ESM.zip › Figure 5 Source Data/5D/HA_Control NaCl.tif]

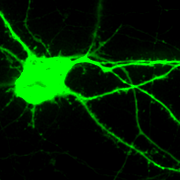

Supplement: Supplementary file 7 — Source data Fig. 5 [file 44319_2026_786_MOESM7_ESM.zip › Figure 5 Source Data/5D/Venus_Control NaCl.tif]

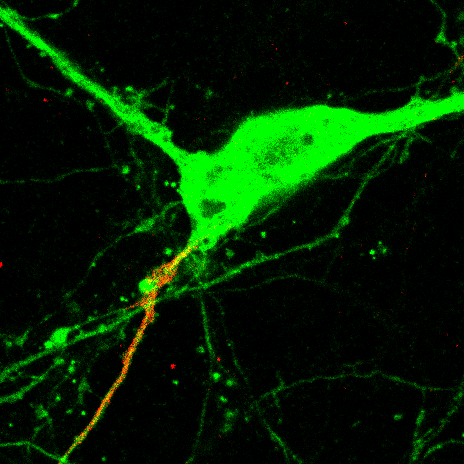

Supplement: Supplementary file 7 — Source data Fig. 5 [file 44319_2026_786_MOESM7_ESM.zip › Figure 5 Source Data/5M/Merge_Control.tif]

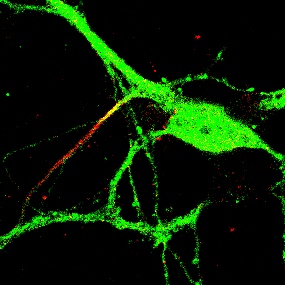

Supplement: Supplementary file 7 — Source data Fig. 5 [file 44319_2026_786_MOESM7_ESM.zip › Figure 5 Source Data/5M/Merge_LINC-DN.tif]

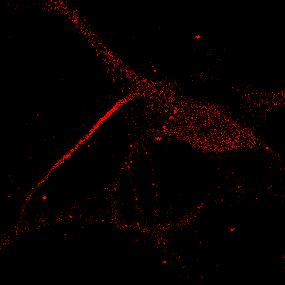

Supplement: Supplementary file 7 — Source data Fig. 5 [file 44319_2026_786_MOESM7_ESM.zip › Figure 5 Source Data/5M/Ankyrin-G_LINC-DN.tif]

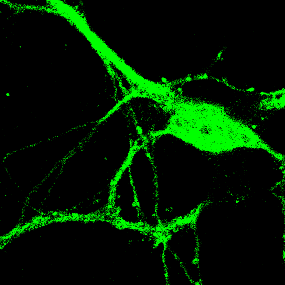

Supplement: Supplementary file 7 — Source data Fig. 5 [file 44319_2026_786_MOESM7_ESM.zip › Figure 5 Source Data/5M/Venus_LINC-DN.tif]

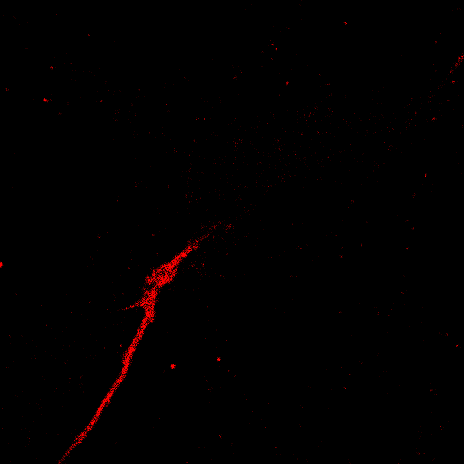

Supplement: Supplementary file 7 — Source data Fig. 5 [file 44319_2026_786_MOESM7_ESM.zip › Figure 5 Source Data/5M/Ankyrin-G _Control.tif]

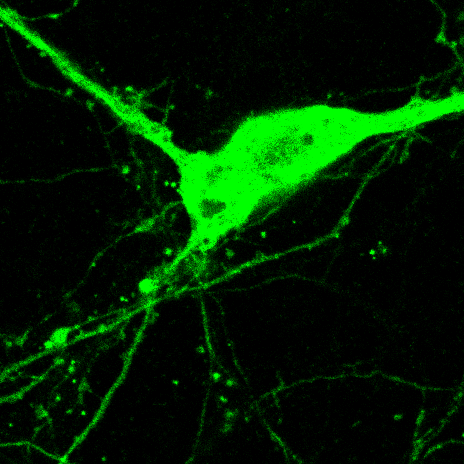

Supplement: Supplementary file 7 — Source data Fig. 5 [file 44319_2026_786_MOESM7_ESM.zip › Figure 5 Source Data/5M/Venus_Control.tif]

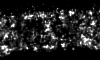

Supplement: Supplementary file 7 — Source data Fig. 5 [file 44319_2026_786_MOESM7_ESM.zip › Figure 5 Source Data/5M/Actin_LINC-DN right.tif]

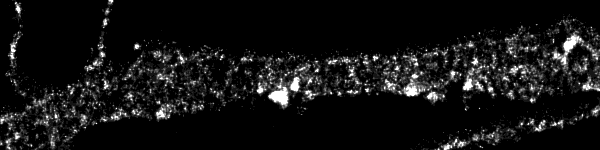

Supplement: Supplementary file 7 — Source data Fig. 5 [file 44319_2026_786_MOESM7_ESM.zip › Figure 5 Source Data/5M/Actin_LINC-DN Middle.tif]

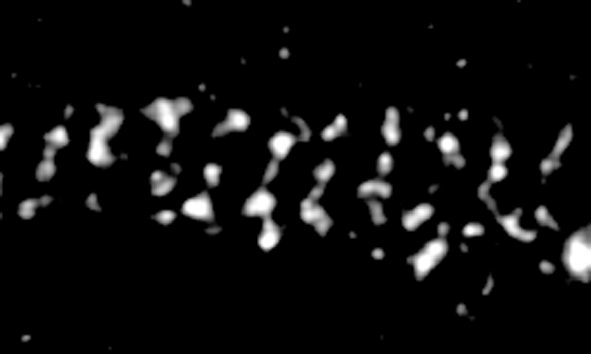

Supplement: Supplementary file 7 — Source data Fig. 5 [file 44319_2026_786_MOESM7_ESM.zip › Figure 5 Source Data/5M/Actin_Control right.tif]

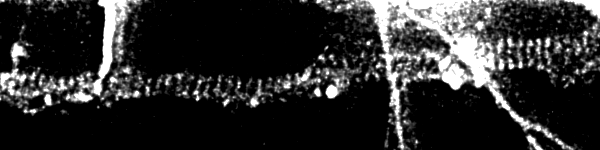

Supplement: Supplementary file 7 — Source data Fig. 5 [file 44319_2026_786_MOESM7_ESM.zip › Figure 5 Source Data/5M/Actin_Control Middle.tif]

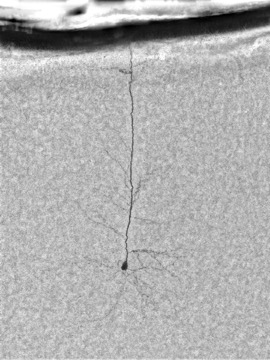

Supplement: Supplementary file 8 — Source data Fig. 6 [file 44319_2026_786_MOESM8_ESM.zip › Figure 6 Source Data/6A/Pyramidal neuron.tif]

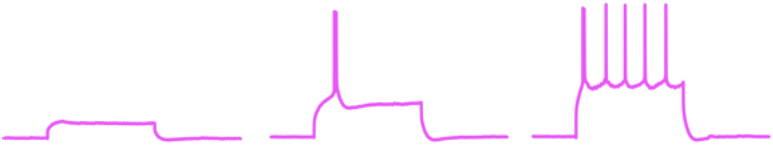

Supplement: Supplementary file 8 — Source data Fig. 6 [file 44319_2026_786_MOESM8_ESM.zip › Figure 6 Source Data/6D/3M LINC-DN.tif]

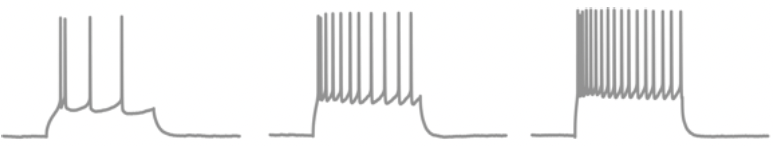

Supplement: Supplementary file 8 — Source data Fig. 6 [file 44319_2026_786_MOESM8_ESM.zip › Figure 6 Source Data/6D/3M NV.tif]

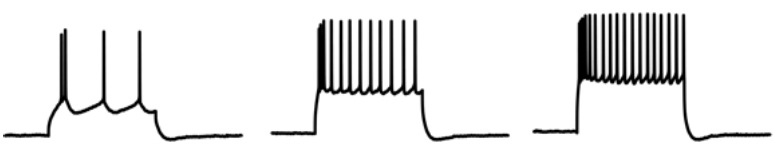

Supplement: Supplementary file 8 — Source data Fig. 6 [file 44319_2026_786_MOESM8_ESM.zip › Figure 6 Source Data/6D/3M Control.tif]

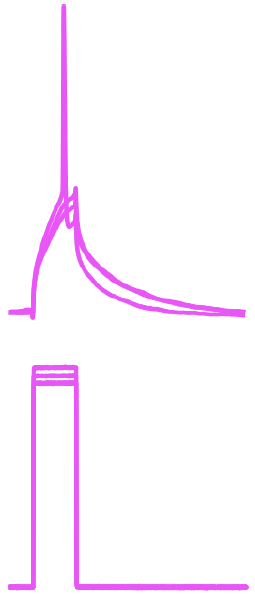

Supplement: Supplementary file 8 — Source data Fig. 6 [file 44319_2026_786_MOESM8_ESM.zip › Figure 6 Source Data/6B/3M LINC-DN.tif]

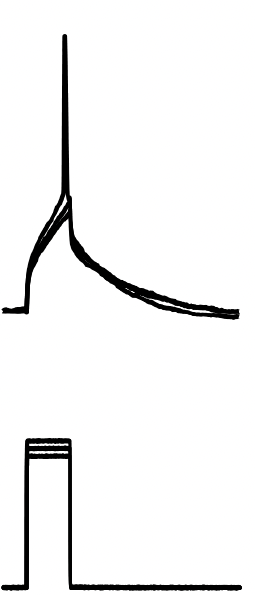

Supplement: Supplementary file 8 — Source data Fig. 6 [file 44319_2026_786_MOESM8_ESM.zip › Figure 6 Source Data/6B/3M Control.tif]

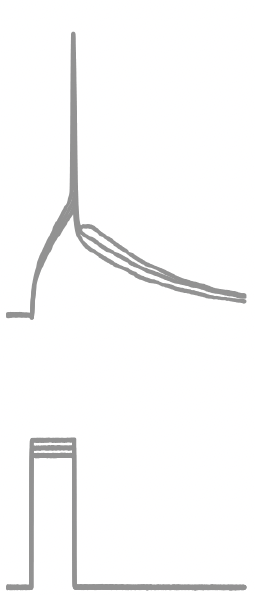

Supplement: Supplementary file 8 — Source data Fig. 6 [file 44319_2026_786_MOESM8_ESM.zip › Figure 6 Source Data/6B/3M NV.tif]
