# Supplementary figures and images for: Age-related decline in nuclear envelope LINC complex drives neuronal aging via axon initial segment dysfunction (part 4 of 9)
Source: EMBO Rep. 2026 May 22;27(13):3788–825. doi: 10.1038/s44319-026-00786-5 (PMC13354796; doi:10.1038/s44319-026-00786-5)

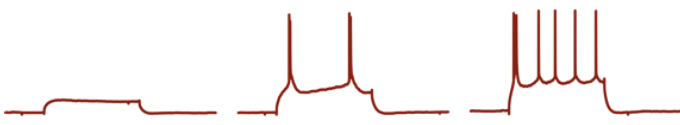

Supplement: Supplementary file 9 — Source data Fig. 7 [file 44319_2026_786_MOESM9_ESM.zip › Figure 7 Source Data/7E/20M NV.tif]

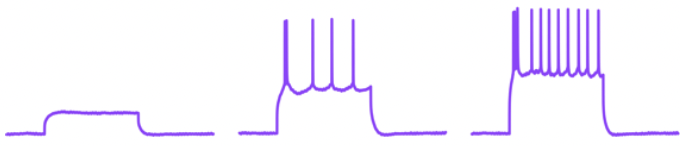

Supplement: Supplementary file 9 — Source data Fig. 7 [file 44319_2026_786_MOESM9_ESM.zip › Figure 7 Source Data/7E/20M + Sun1 + AnkG KO.tif]

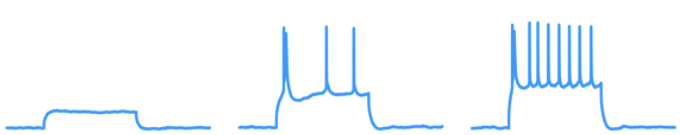

Supplement: Supplementary file 9 — Source data Fig. 7 [file 44319_2026_786_MOESM9_ESM.zip › Figure 7 Source Data/7E/20M + AnkG KO.tif]

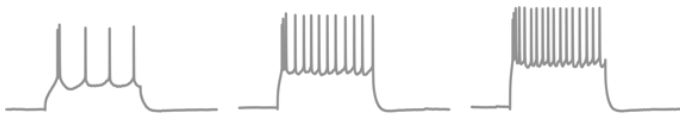

Supplement: Supplementary file 9 — Source data Fig. 7 [file 44319_2026_786_MOESM9_ESM.zip › Figure 7 Source Data/7E/3M NV.tif]

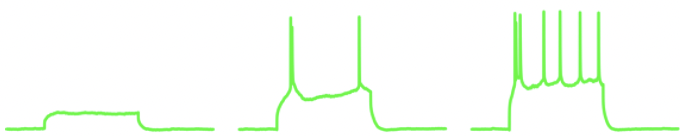

Supplement: Supplementary file 9 — Source data Fig. 7 [file 44319_2026_786_MOESM9_ESM.zip › Figure 7 Source Data/7E/20M Control.tif]

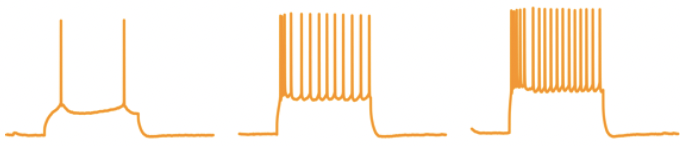

Supplement: Supplementary file 9 — Source data Fig. 7 [file 44319_2026_786_MOESM9_ESM.zip › Figure 7 Source Data/7E/20M + Sun1.tif]

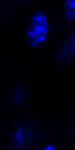

Supplement: Supplementary file 9 — Source data Fig. 7 [file 44319_2026_786_MOESM9_ESM.zip › Figure 7 Source Data/7A/Hoechst_3M NV.tif]

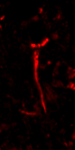

Supplement: Supplementary file 9 — Source data Fig. 7 [file 44319_2026_786_MOESM9_ESM.zip › Figure 7 Source Data/7A/Ankyrin-G_20M + Sun1.tif]

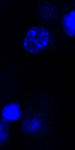

Supplement: Supplementary file 9 — Source data Fig. 7 [file 44319_2026_786_MOESM9_ESM.zip › Figure 7 Source Data/7A/Hoechst_20M NV.tif]

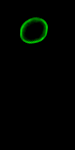

Supplement: Supplementary file 9 — Source data Fig. 7 [file 44319_2026_786_MOESM9_ESM.zip › Figure 7 Source Data/7A/HA_20M + Sun1.tif]

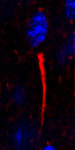

Supplement: Supplementary file 9 — Source data Fig. 7 [file 44319_2026_786_MOESM9_ESM.zip › Figure 7 Source Data/7A/Merge_3M NV.tif]

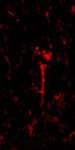

Supplement: Supplementary file 9 — Source data Fig. 7 [file 44319_2026_786_MOESM9_ESM.zip › Figure 7 Source Data/7A/Ankyrin-G_20M Control.tif]

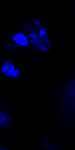

Supplement: Supplementary file 9 — Source data Fig. 7 [file 44319_2026_786_MOESM9_ESM.zip › Figure 7 Source Data/7A/Hoechst_20M Control.tif]

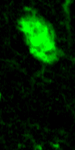

Supplement: Supplementary file 9 — Source data Fig. 7 [file 44319_2026_786_MOESM9_ESM.zip › Figure 7 Source Data/7A/Venus_20M Control.tif]

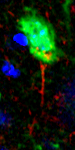

Supplement: Supplementary file 9 — Source data Fig. 7 [file 44319_2026_786_MOESM9_ESM.zip › Figure 7 Source Data/7A/Merge_20M Control.tif]

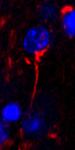

Supplement: Supplementary file 9 — Source data Fig. 7 [file 44319_2026_786_MOESM9_ESM.zip › Figure 7 Source Data/7A/Merge_20M NV.tif]

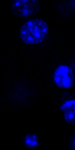

Supplement: Supplementary file 9 — Source data Fig. 7 [file 44319_2026_786_MOESM9_ESM.zip › Figure 7 Source Data/7A/Hoechst_20M + Sun1.tif]

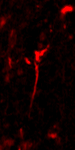

Supplement: Supplementary file 9 — Source data Fig. 7 [file 44319_2026_786_MOESM9_ESM.zip › Figure 7 Source Data/7A/Ankyrin-G_20M NV.tif]

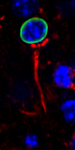

Supplement: Supplementary file 9 — Source data Fig. 7 [file 44319_2026_786_MOESM9_ESM.zip › Figure 7 Source Data/7A/Merge_20M + Sun1.tif]

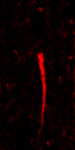

Supplement: Supplementary file 9 — Source data Fig. 7 [file 44319_2026_786_MOESM9_ESM.zip › Figure 7 Source Data/7A/Ankyrin-G_3M NV.tif]

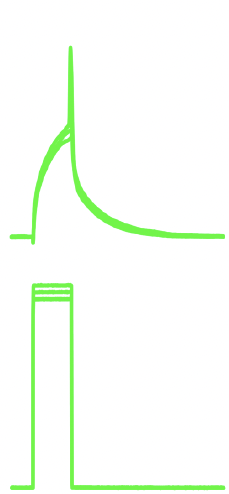

Supplement: Supplementary file 9 — Source data Fig. 7 [file 44319_2026_786_MOESM9_ESM.zip › Figure 7 Source Data/7C/20M Control.tif]

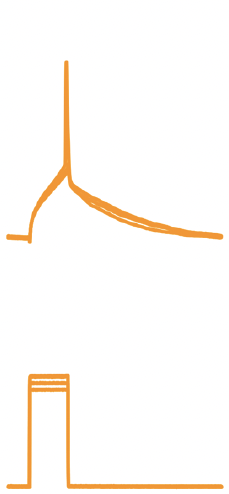

Supplement: Supplementary file 9 — Source data Fig. 7 [file 44319_2026_786_MOESM9_ESM.zip › Figure 7 Source Data/7C/20M + Sun1.tif]

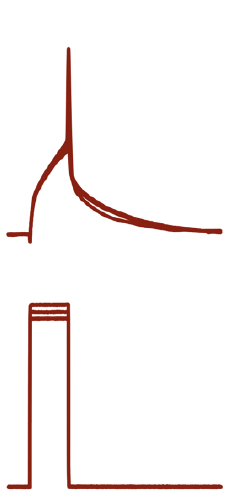

Supplement: Supplementary file 9 — Source data Fig. 7 [file 44319_2026_786_MOESM9_ESM.zip › Figure 7 Source Data/7C/20M NV.tif]

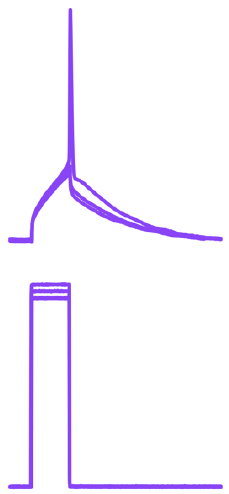

Supplement: Supplementary file 9 — Source data Fig. 7 [file 44319_2026_786_MOESM9_ESM.zip › Figure 7 Source Data/7C/20M + Sun1 + AnkG KO.tif]

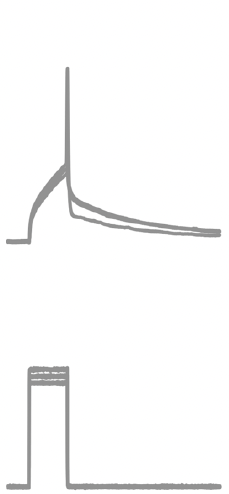

Supplement: Supplementary file 9 — Source data Fig. 7 [file 44319_2026_786_MOESM9_ESM.zip › Figure 7 Source Data/7C/3M NV.tif]

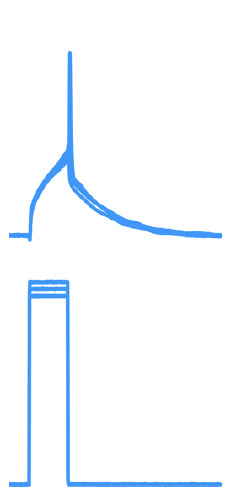

Supplement: Supplementary file 9 — Source data Fig. 7 [file 44319_2026_786_MOESM9_ESM.zip › Figure 7 Source Data/7C/20M + AnkG KO.tif]

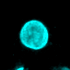

Supplement: Supplementary file 10 — Figure EV1 Source Data [file 44319_2026_786_MOESM10_ESM.zip › Figure EV1 Source Data/EV1D/HA_Control.tif]

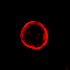

Supplement: Supplementary file 10 — Figure EV1 Source Data [file 44319_2026_786_MOESM10_ESM.zip › Figure EV1 Source Data/EV1D/Nesprin-1_Control.tif]

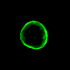

Supplement: Supplementary file 10 — Figure EV1 Source Data [file 44319_2026_786_MOESM10_ESM.zip › Figure EV1 Source Data/EV1D/Sun2_Control.tif]

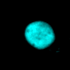

Supplement: Supplementary file 10 — Figure EV1 Source Data [file 44319_2026_786_MOESM10_ESM.zip › Figure EV1 Source Data/EV1D/HA_Sun2 KO.tif]

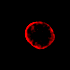

Supplement: Supplementary file 10 — Figure EV1 Source Data [file 44319_2026_786_MOESM10_ESM.zip › Figure EV1 Source Data/EV1D/Nesprin-1_Sun2 KO.tif]

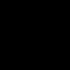

Supplement: Supplementary file 10 — Figure EV1 Source Data [file 44319_2026_786_MOESM10_ESM.zip › Figure EV1 Source Data/EV1D/Sun2_Sun2 KO.tif]

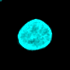

Supplement: Supplementary file 10 — Figure EV1 Source Data [file 44319_2026_786_MOESM10_ESM.zip › Figure EV1 Source Data/EV1E/HA_Control.tif]

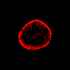

Supplement: Supplementary file 10 — Figure EV1 Source Data [file 44319_2026_786_MOESM10_ESM.zip › Figure EV1 Source Data/EV1E/Nesprin-2_Control.tif]

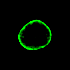

Supplement: Supplementary file 10 — Figure EV1 Source Data [file 44319_2026_786_MOESM10_ESM.zip › Figure EV1 Source Data/EV1E/Sun2_Control.tif]

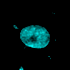

Supplement: Supplementary file 10 — Figure EV1 Source Data [file 44319_2026_786_MOESM10_ESM.zip › Figure EV1 Source Data/EV1E/HA_Sun2 KO.tif]

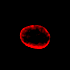

Supplement: Supplementary file 10 — Figure EV1 Source Data [file 44319_2026_786_MOESM10_ESM.zip › Figure EV1 Source Data/EV1E/Nesprin-2_Sun2 KO.tif]

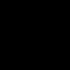

Supplement: Supplementary file 10 — Figure EV1 Source Data [file 44319_2026_786_MOESM10_ESM.zip › Figure EV1 Source Data/EV1E/Sun2_Sun2 KO.tif]

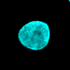

Supplement: Supplementary file 10 — Figure EV1 Source Data [file 44319_2026_786_MOESM10_ESM.zip › Figure EV1 Source Data/EV1A/HA_Control.tif]

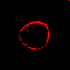

Supplement: Supplementary file 10 — Figure EV1 Source Data [file 44319_2026_786_MOESM10_ESM.zip › Figure EV1 Source Data/EV1A/Sun2_Control.tif]

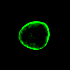

Supplement: Supplementary file 10 — Figure EV1 Source Data [file 44319_2026_786_MOESM10_ESM.zip › Figure EV1 Source Data/EV1A/Sun1_Control.tif]

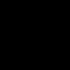

Supplement: Supplementary file 10 — Figure EV1 Source Data [file 44319_2026_786_MOESM10_ESM.zip › Figure EV1 Source Data/EV1A/Sun1_Sun1 KO.tif]

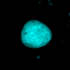

Supplement: Supplementary file 10 — Figure EV1 Source Data [file 44319_2026_786_MOESM10_ESM.zip › Figure EV1 Source Data/EV1A/HA_Sun1 KO.tif]

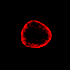

Supplement: Supplementary file 10 — Figure EV1 Source Data [file 44319_2026_786_MOESM10_ESM.zip › Figure EV1 Source Data/EV1A/Sun2_Sun1 KO.tif]

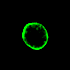

Supplement: Supplementary file 10 — Figure EV1 Source Data [file 44319_2026_786_MOESM10_ESM.zip › Figure EV1 Source Data/EV1A/Sun1_Sun2 KO.tif]

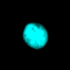

Supplement: Supplementary file 10 — Figure EV1 Source Data [file 44319_2026_786_MOESM10_ESM.zip › Figure EV1 Source Data/EV1A/HA_Sun2 KO.tif]

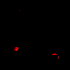

Supplement: Supplementary file 10 — Figure EV1 Source Data [file 44319_2026_786_MOESM10_ESM.zip › Figure EV1 Source Data/EV1A/Sun2_Sun2 KO.tif]

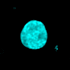

Supplement: Supplementary file 10 — Figure EV1 Source Data [file 44319_2026_786_MOESM10_ESM.zip › Figure EV1 Source Data/EV1B/HA_Control.tif]

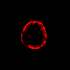

Supplement: Supplementary file 10 — Figure EV1 Source Data [file 44319_2026_786_MOESM10_ESM.zip › Figure EV1 Source Data/EV1B/Nesprin-1_Control.tif]

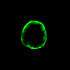

Supplement: Supplementary file 10 — Figure EV1 Source Data [file 44319_2026_786_MOESM10_ESM.zip › Figure EV1 Source Data/EV1B/Sun1_Control.tif]

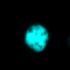

Supplement: Supplementary file 10 — Figure EV1 Source Data [file 44319_2026_786_MOESM10_ESM.zip › Figure EV1 Source Data/EV1B/HA_Sun1 KO.tif]

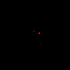

Supplement: Supplementary file 10 — Figure EV1 Source Data [file 44319_2026_786_MOESM10_ESM.zip › Figure EV1 Source Data/EV1B/Nesprin-1_Sun1 KO.tif]

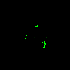

Supplement: Supplementary file 10 — Figure EV1 Source Data [file 44319_2026_786_MOESM10_ESM.zip › Figure EV1 Source Data/EV1B/Sun1_Sun1 KO.tif]

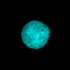

Supplement: Supplementary file 10 — Figure EV1 Source Data [file 44319_2026_786_MOESM10_ESM.zip › Figure EV1 Source Data/EV1C/HA_Control.tif]

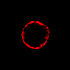

Supplement: Supplementary file 10 — Figure EV1 Source Data [file 44319_2026_786_MOESM10_ESM.zip › Figure EV1 Source Data/EV1C/Nesprin-2_Control.tif]

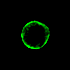

Supplement: Supplementary file 10 — Figure EV1 Source Data [file 44319_2026_786_MOESM10_ESM.zip › Figure EV1 Source Data/EV1C/Sun1_Control.tif]

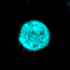

Supplement: Supplementary file 10 — Figure EV1 Source Data [file 44319_2026_786_MOESM10_ESM.zip › Figure EV1 Source Data/EV1C/HA_Sun1 KO.tif]

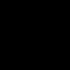

Supplement: Supplementary file 10 — Figure EV1 Source Data [file 44319_2026_786_MOESM10_ESM.zip › Figure EV1 Source Data/EV1C/Nesprin-2_Sun1 KO.tif]

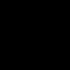

Supplement: Supplementary file 10 — Figure EV1 Source Data [file 44319_2026_786_MOESM10_ESM.zip › Figure EV1 Source Data/EV1C/Sun1_Sun1 KO.tif]

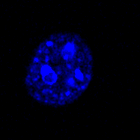

Supplement: Supplementary file 11 — Figure EV2 Source Data [file 44319_2026_786_MOESM11_ESM.zip › Figure EV2 Source Data/EV2B/Hoechst_20M NV.tif]

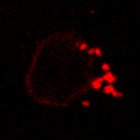

Supplement: Supplementary file 11 — Figure EV2 Source Data [file 44319_2026_786_MOESM11_ESM.zip › Figure EV2 Source Data/EV2B/Sun1_20M NV.tif]

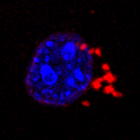

Supplement: Supplementary file 11 — Figure EV2 Source Data [file 44319_2026_786_MOESM11_ESM.zip › Figure EV2 Source Data/EV2B/Merge_20M NV.tif]

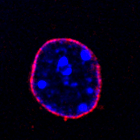

Supplement: Supplementary file 11 — Figure EV2 Source Data [file 44319_2026_786_MOESM11_ESM.zip › Figure EV2 Source Data/EV2B/Merge_3M NV.tif]

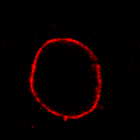

Supplement: Supplementary file 11 — Figure EV2 Source Data [file 44319_2026_786_MOESM11_ESM.zip › Figure EV2 Source Data/EV2B/Sun1_3M NV.tif]

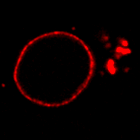

Supplement: Supplementary file 11 — Figure EV2 Source Data [file 44319_2026_786_MOESM11_ESM.zip › Figure EV2 Source Data/EV2B/Sun1_20M+Sun1.tif]

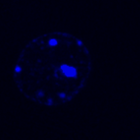

Supplement: Supplementary file 11 — Figure EV2 Source Data [file 44319_2026_786_MOESM11_ESM.zip › Figure EV2 Source Data/EV2B/Hoechst_20M+Sun1.tif]

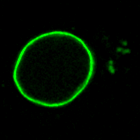

Supplement: Supplementary file 11 — Figure EV2 Source Data [file 44319_2026_786_MOESM11_ESM.zip › Figure EV2 Source Data/EV2B/HA_20M+Sun1.tif]

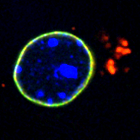

Supplement: Supplementary file 11 — Figure EV2 Source Data [file 44319_2026_786_MOESM11_ESM.zip › Figure EV2 Source Data/EV2B/Merge_20M+Sun1.tif]

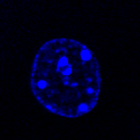

Supplement: Supplementary file 11 — Figure EV2 Source Data [file 44319_2026_786_MOESM11_ESM.zip › Figure EV2 Source Data/EV2B/Hoechst_3M NV.tif]

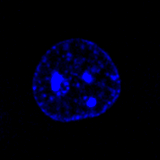

Supplement: Supplementary file 11 — Figure EV2 Source Data [file 44319_2026_786_MOESM11_ESM.zip › Figure EV2 Source Data/EV2D/Hoechst_3M NV.tif]

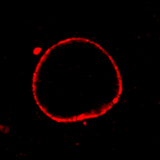

Supplement: Supplementary file 11 — Figure EV2 Source Data [file 44319_2026_786_MOESM11_ESM.zip › Figure EV2 Source Data/EV2D/Nesprin-1_3M NV.tif]

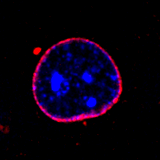

Supplement: Supplementary file 11 — Figure EV2 Source Data [file 44319_2026_786_MOESM11_ESM.zip › Figure EV2 Source Data/EV2D/Merge_3M NV.tif]

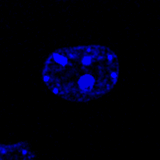

Supplement: Supplementary file 11 — Figure EV2 Source Data [file 44319_2026_786_MOESM11_ESM.zip › Figure EV2 Source Data/EV2D/Hoechst_20M NV.tif]

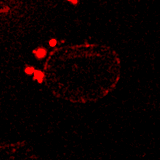

Supplement: Supplementary file 11 — Figure EV2 Source Data [file 44319_2026_786_MOESM11_ESM.zip › Figure EV2 Source Data/EV2D/Nesprin-1_20M NV.tif]

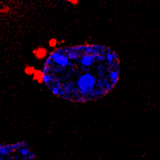

Supplement: Supplementary file 11 — Figure EV2 Source Data [file 44319_2026_786_MOESM11_ESM.zip › Figure EV2 Source Data/EV2D/Merge_20M NV.tif]

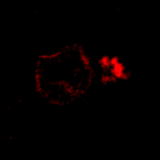

Supplement: Supplementary file 11 — Figure EV2 Source Data [file 44319_2026_786_MOESM11_ESM.zip › Figure EV2 Source Data/EV2D/Nesprin-1_20M Control.tif]

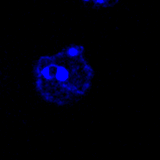

Supplement: Supplementary file 11 — Figure EV2 Source Data [file 44319_2026_786_MOESM11_ESM.zip › Figure EV2 Source Data/EV2D/Hoechst_20M Control.tif]

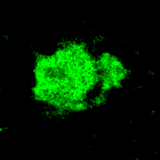

Supplement: Supplementary file 11 — Figure EV2 Source Data [file 44319_2026_786_MOESM11_ESM.zip › Figure EV2 Source Data/EV2D/Venus_20M Control.tif]

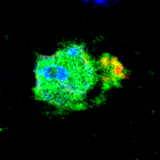

Supplement: Supplementary file 11 — Figure EV2 Source Data [file 44319_2026_786_MOESM11_ESM.zip › Figure EV2 Source Data/EV2D/Merge_20M Control.tif]

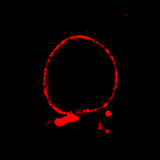

Supplement: Supplementary file 11 — Figure EV2 Source Data [file 44319_2026_786_MOESM11_ESM.zip › Figure EV2 Source Data/EV2D/Nesprin-1_20M+Sun1.tif]

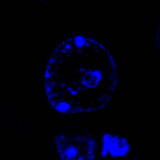

Supplement: Supplementary file 11 — Figure EV2 Source Data [file 44319_2026_786_MOESM11_ESM.zip › Figure EV2 Source Data/EV2D/Hoechst_20M+Sun1.tif]

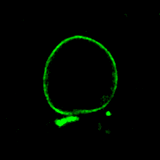

Supplement: Supplementary file 11 — Figure EV2 Source Data [file 44319_2026_786_MOESM11_ESM.zip › Figure EV2 Source Data/EV2D/HA_20M+Sun1.tif]

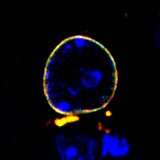

Supplement: Supplementary file 11 — Figure EV2 Source Data [file 44319_2026_786_MOESM11_ESM.zip › Figure EV2 Source Data/EV2D/Merge_20M+Sun1.tif]

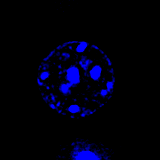

Supplement: Supplementary file 11 — Figure EV2 Source Data [file 44319_2026_786_MOESM11_ESM.zip › Figure EV2 Source Data/EV2F/Hoechst_3M NV.tif]

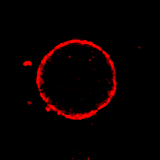

Supplement: Supplementary file 11 — Figure EV2 Source Data [file 44319_2026_786_MOESM11_ESM.zip › Figure EV2 Source Data/EV2F/Nesprin-2_3M NV.tif]

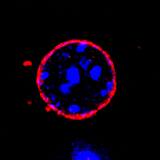

Supplement: Supplementary file 11 — Figure EV2 Source Data [file 44319_2026_786_MOESM11_ESM.zip › Figure EV2 Source Data/EV2F/Merge_3M NV.tif]

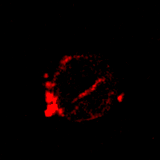

Supplement: Supplementary file 11 — Figure EV2 Source Data [file 44319_2026_786_MOESM11_ESM.zip › Figure EV2 Source Data/EV2F/Nesprin-2_20M NV.tif]

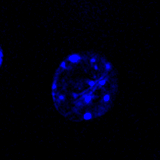

Supplement: Supplementary file 11 — Figure EV2 Source Data [file 44319_2026_786_MOESM11_ESM.zip › Figure EV2 Source Data/EV2F/Hoechst_20M NV.tif]

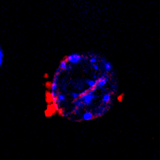

Supplement: Supplementary file 11 — Figure EV2 Source Data [file 44319_2026_786_MOESM11_ESM.zip › Figure EV2 Source Data/EV2F/Merge_20M NV.tif]

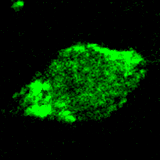

Supplement: Supplementary file 11 — Figure EV2 Source Data [file 44319_2026_786_MOESM11_ESM.zip › Figure EV2 Source Data/EV2F/Venus_20M Control.tif]

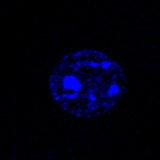

Supplement: Supplementary file 11 — Figure EV2 Source Data [file 44319_2026_786_MOESM11_ESM.zip › Figure EV2 Source Data/EV2F/Hoechst_20M Control.tif]

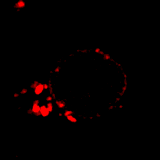

Supplement: Supplementary file 11 — Figure EV2 Source Data [file 44319_2026_786_MOESM11_ESM.zip › Figure EV2 Source Data/EV2F/Nesprin-2_20M Control.tif]

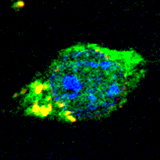

Supplement: Supplementary file 11 — Figure EV2 Source Data [file 44319_2026_786_MOESM11_ESM.zip › Figure EV2 Source Data/EV2F/Merge_20M Control.tif]

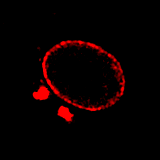

Supplement: Supplementary file 11 — Figure EV2 Source Data [file 44319_2026_786_MOESM11_ESM.zip › Figure EV2 Source Data/EV2F/Nesprin-2_20M+Sun1.tif]

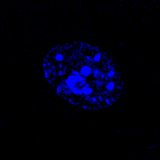

Supplement: Supplementary file 11 — Figure EV2 Source Data [file 44319_2026_786_MOESM11_ESM.zip › Figure EV2 Source Data/EV2F/Hoechst_20M+Sun1.tif]

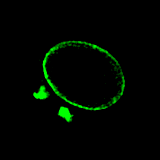

Supplement: Supplementary file 11 — Figure EV2 Source Data [file 44319_2026_786_MOESM11_ESM.zip › Figure EV2 Source Data/EV2F/HA_20M+Sun1.tif]

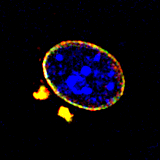

Supplement: Supplementary file 11 — Figure EV2 Source Data [file 44319_2026_786_MOESM11_ESM.zip › Figure EV2 Source Data/EV2F/Merge_20M+Sun1.tif]

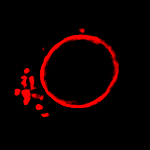

Supplement: Supplementary file 11 — Figure EV2 Source Data [file 44319_2026_786_MOESM11_ESM.zip › Figure EV2 Source Data/EV2H/HA_20M+Sun1.tif]

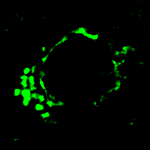

Supplement: Supplementary file 11 — Figure EV2 Source Data [file 44319_2026_786_MOESM11_ESM.zip › Figure EV2 Source Data/EV2H/GM130_20M+Sun1.tif]

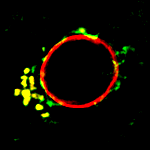

Supplement: Supplementary file 11 — Figure EV2 Source Data [file 44319_2026_786_MOESM11_ESM.zip › Figure EV2 Source Data/EV2H/Merge_20M+Sun1.tif]
