# Supplementary figures and images for: Age-related decline in nuclear envelope LINC complex drives neuronal aging via axon initial segment dysfunction (part 5 of 9)
Source: EMBO Rep. 2026 May 22;27(13):3788–825. doi: 10.1038/s44319-026-00786-5 (PMC13354796; doi:10.1038/s44319-026-00786-5)

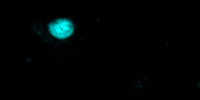

Supplement: Supplementary file 14 — Figure EV5 Source Data [file 44319_2026_786_MOESM14_ESM.zip › Figure EV5 Source Data/EV5F/HA_Control for Sun1 KO.tif]

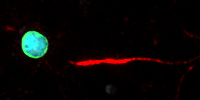

Supplement: Supplementary file 14 — Figure EV5 Source Data [file 44319_2026_786_MOESM14_ESM.zip › Figure EV5 Source Data/EV5F/Merge_Control for Sun2 KO.tif]

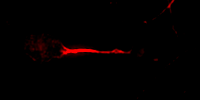

Supplement: Supplementary file 14 — Figure EV5 Source Data [file 44319_2026_786_MOESM14_ESM.zip › Figure EV5 Source Data/EV5F/Ankyrin-G_Sun1 KO.tif]

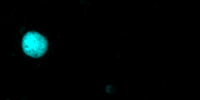

Supplement: Supplementary file 14 — Figure EV5 Source Data [file 44319_2026_786_MOESM14_ESM.zip › Figure EV5 Source Data/EV5F/HA_Control for Sun2 KO.tif]

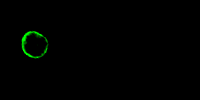

Supplement: Supplementary file 14 — Figure EV5 Source Data [file 44319_2026_786_MOESM14_ESM.zip › Figure EV5 Source Data/EV5F/Sun2_Control for Sun2 KO.tif]

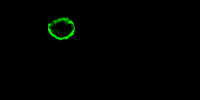

Supplement: Supplementary file 14 — Figure EV5 Source Data [file 44319_2026_786_MOESM14_ESM.zip › Figure EV5 Source Data/EV5F/Sun1_Control for Sun1 KO.tif]

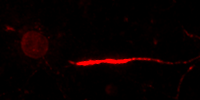

Supplement: Supplementary file 14 — Figure EV5 Source Data [file 44319_2026_786_MOESM14_ESM.zip › Figure EV5 Source Data/EV5F/Ankyrin-G_Control for Sun2 KO.tif]

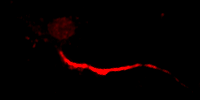

Supplement: Supplementary file 14 — Figure EV5 Source Data [file 44319_2026_786_MOESM14_ESM.zip › Figure EV5 Source Data/EV5F/Ankyrin-G_Control for Sun1 KO.tif]

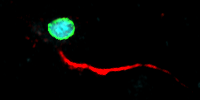

Supplement: Supplementary file 14 — Figure EV5 Source Data [file 44319_2026_786_MOESM14_ESM.zip › Figure EV5 Source Data/EV5F/Merge_Control for Sun1 KO.tif]

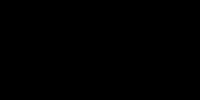

Supplement: Supplementary file 14 — Figure EV5 Source Data [file 44319_2026_786_MOESM14_ESM.zip › Figure EV5 Source Data/EV5F/Sun1_Sun1 KO.tif]

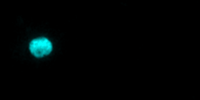

Supplement: Supplementary file 14 — Figure EV5 Source Data [file 44319_2026_786_MOESM14_ESM.zip › Figure EV5 Source Data/EV5F/HA_Sun1 KO.tif]

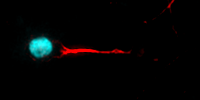

Supplement: Supplementary file 14 — Figure EV5 Source Data [file 44319_2026_786_MOESM14_ESM.zip › Figure EV5 Source Data/EV5F/Merge_Sun1 KO.tif]

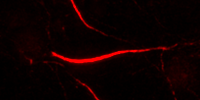

Supplement: Supplementary file 14 — Figure EV5 Source Data [file 44319_2026_786_MOESM14_ESM.zip › Figure EV5 Source Data/EV5F/Ankyrin-G_Sun2 KO.tif]

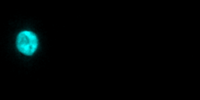

Supplement: Supplementary file 14 — Figure EV5 Source Data [file 44319_2026_786_MOESM14_ESM.zip › Figure EV5 Source Data/EV5F/HA_Sun2 KO.tif]

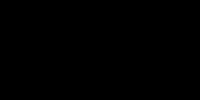

Supplement: Supplementary file 14 — Figure EV5 Source Data [file 44319_2026_786_MOESM14_ESM.zip › Figure EV5 Source Data/EV5F/Sun2_Sun2 KO.tif]

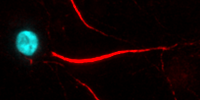

Supplement: Supplementary file 14 — Figure EV5 Source Data [file 44319_2026_786_MOESM14_ESM.zip › Figure EV5 Source Data/EV5F/Merge_Sun2 KO.tif]

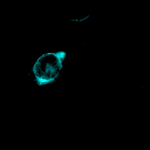

Supplement: Supplementary file 14 — Figure EV5 Source Data [file 44319_2026_786_MOESM14_ESM.zip › Figure EV5 Source Data/EV5C/HA_Nesp2-KASH.tif]

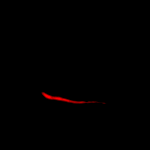

Supplement: Supplementary file 14 — Figure EV5 Source Data [file 44319_2026_786_MOESM14_ESM.zip › Figure EV5 Source Data/EV5C/Ankyrin-G_Nesp2-KASH.tif]

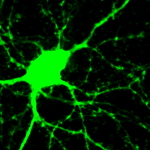

Supplement: Supplementary file 14 — Figure EV5 Source Data [file 44319_2026_786_MOESM14_ESM.zip › Figure EV5 Source Data/EV5C/Venus_Nesp2-KASH.tif]

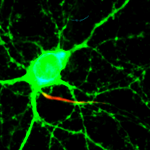

Supplement: Supplementary file 14 — Figure EV5 Source Data [file 44319_2026_786_MOESM14_ESM.zip › Figure EV5 Source Data/EV5C/Merge_Nesp2-KASH.tif]

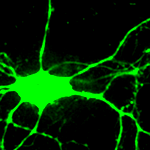

Supplement: Supplementary file 14 — Figure EV5 Source Data [file 44319_2026_786_MOESM14_ESM.zip › Figure EV5 Source Data/EV5C/Venus_Control.tif]

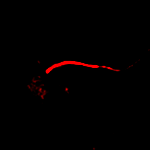

Supplement: Supplementary file 14 — Figure EV5 Source Data [file 44319_2026_786_MOESM14_ESM.zip › Figure EV5 Source Data/EV5C/Ankyrin-G_Control.tif]

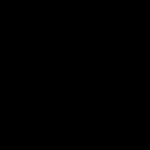

Supplement: Supplementary file 14 — Figure EV5 Source Data [file 44319_2026_786_MOESM14_ESM.zip › Figure EV5 Source Data/EV5C/HA_Control.tif]

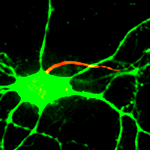

Supplement: Supplementary file 14 — Figure EV5 Source Data [file 44319_2026_786_MOESM14_ESM.zip › Figure EV5 Source Data/EV5C/Merge_Control.tif]

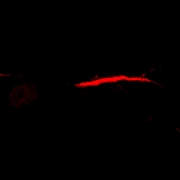

Supplement: Supplementary file 14 — Figure EV5 Source Data [file 44319_2026_786_MOESM14_ESM.zip › Figure EV5 Source Data/EV5H/Ankyrin-G_[1]+LINC-DN.tif]

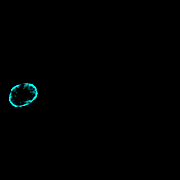

Supplement: Supplementary file 14 — Figure EV5 Source Data [file 44319_2026_786_MOESM14_ESM.zip › Figure EV5 Source Data/EV5H/HA_[1]+LINC-DN.tif]

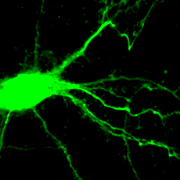

Supplement: Supplementary file 14 — Figure EV5 Source Data [file 44319_2026_786_MOESM14_ESM.zip › Figure EV5 Source Data/EV5H/Venus_[1]+LINC-DN.tif]

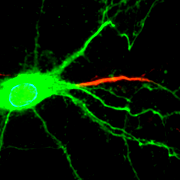

Supplement: Supplementary file 14 — Figure EV5 Source Data [file 44319_2026_786_MOESM14_ESM.zip › Figure EV5 Source Data/EV5H/Merge_[1]+LINC-DN.tif]

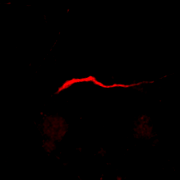

Supplement: Supplementary file 14 — Figure EV5 Source Data [file 44319_2026_786_MOESM14_ESM.zip › Figure EV5 Source Data/EV5H/Ankyrin-G_[2]+LINC-DN.tif]

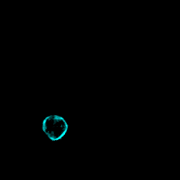

Supplement: Supplementary file 14 — Figure EV5 Source Data [file 44319_2026_786_MOESM14_ESM.zip › Figure EV5 Source Data/EV5H/HA_[2]+LINC-DN.tif]

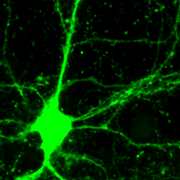

Supplement: Supplementary file 14 — Figure EV5 Source Data [file 44319_2026_786_MOESM14_ESM.zip › Figure EV5 Source Data/EV5H/Venus_[2]+LINC-DN.tif]

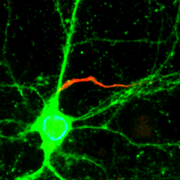

Supplement: Supplementary file 14 — Figure EV5 Source Data [file 44319_2026_786_MOESM14_ESM.zip › Figure EV5 Source Data/EV5H/Merge_[2]+LINC-DN.tif]

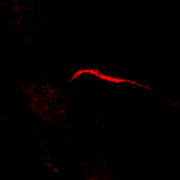

Supplement: Supplementary file 14 — Figure EV5 Source Data [file 44319_2026_786_MOESM14_ESM.zip › Figure EV5 Source Data/EV5H/Ankyrin-G_Control.tif]

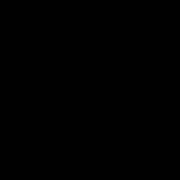

Supplement: Supplementary file 14 — Figure EV5 Source Data [file 44319_2026_786_MOESM14_ESM.zip › Figure EV5 Source Data/EV5H/HA_Control.tif]

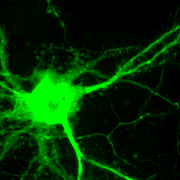

Supplement: Supplementary file 14 — Figure EV5 Source Data [file 44319_2026_786_MOESM14_ESM.zip › Figure EV5 Source Data/EV5H/Venus_Control.tif]

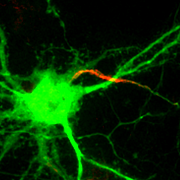

Supplement: Supplementary file 14 — Figure EV5 Source Data [file 44319_2026_786_MOESM14_ESM.zip › Figure EV5 Source Data/EV5H/Merge_Control.tif]

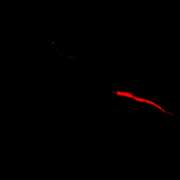

Supplement: Supplementary file 14 — Figure EV5 Source Data [file 44319_2026_786_MOESM14_ESM.zip › Figure EV5 Source Data/EV5H/Ankyrin-G_(-)+LINC-DN.tif]

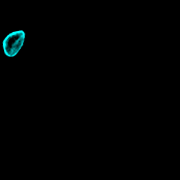

Supplement: Supplementary file 14 — Figure EV5 Source Data [file 44319_2026_786_MOESM14_ESM.zip › Figure EV5 Source Data/EV5H/HA_(-)+LINC-DN.tif]

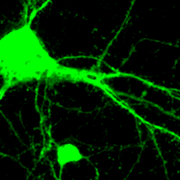

Supplement: Supplementary file 14 — Figure EV5 Source Data [file 44319_2026_786_MOESM14_ESM.zip › Figure EV5 Source Data/EV5H/Venus_(-)+LINC-DN.tif]

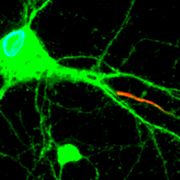

Supplement: Supplementary file 14 — Figure EV5 Source Data [file 44319_2026_786_MOESM14_ESM.zip › Figure EV5 Source Data/EV5H/Merge_(-)+LINC-DN.tif]

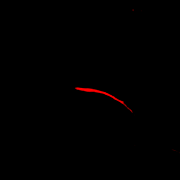

Supplement: Supplementary file 14 — Figure EV5 Source Data [file 44319_2026_786_MOESM14_ESM.zip › Figure EV5 Source Data/EV5H/Ankyrin-G_[3]+LINC-DN.tif]

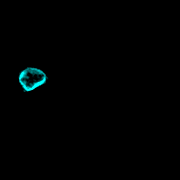

Supplement: Supplementary file 14 — Figure EV5 Source Data [file 44319_2026_786_MOESM14_ESM.zip › Figure EV5 Source Data/EV5H/HA_[3]+LINC-DN.tif]

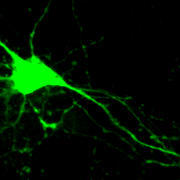

Supplement: Supplementary file 14 — Figure EV5 Source Data [file 44319_2026_786_MOESM14_ESM.zip › Figure EV5 Source Data/EV5H/Venus_[3]+LINC-DN.tif]

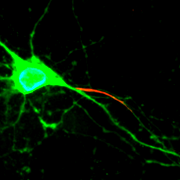

Supplement: Supplementary file 14 — Figure EV5 Source Data [file 44319_2026_786_MOESM14_ESM.zip › Figure EV5 Source Data/EV5H/Merge_[3]+LINC-DN.tif]

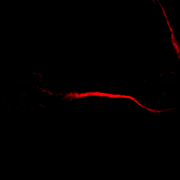

Supplement: Supplementary file 14 — Figure EV5 Source Data [file 44319_2026_786_MOESM14_ESM.zip › Figure EV5 Source Data/EV5K/Ankyrin-G_Control.tif]

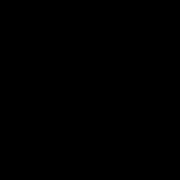

Supplement: Supplementary file 14 — Figure EV5 Source Data [file 44319_2026_786_MOESM14_ESM.zip › Figure EV5 Source Data/EV5K/HA_Control.tif]

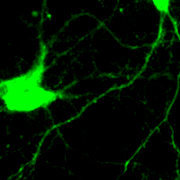

Supplement: Supplementary file 14 — Figure EV5 Source Data [file 44319_2026_786_MOESM14_ESM.zip › Figure EV5 Source Data/EV5K/Venus_Control.tif]

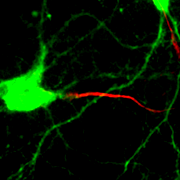

Supplement: Supplementary file 14 — Figure EV5 Source Data [file 44319_2026_786_MOESM14_ESM.zip › Figure EV5 Source Data/EV5K/Merge_Control.tif]

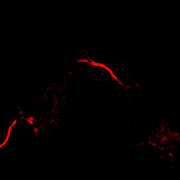

Supplement: Supplementary file 14 — Figure EV5 Source Data [file 44319_2026_786_MOESM14_ESM.zip › Figure EV5 Source Data/EV5K/Ankyrin-G_LINC-DN.tif]

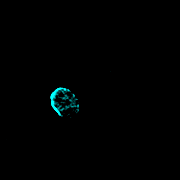

Supplement: Supplementary file 14 — Figure EV5 Source Data [file 44319_2026_786_MOESM14_ESM.zip › Figure EV5 Source Data/EV5K/HA_LINC-DN.tif]

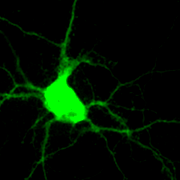

Supplement: Supplementary file 14 — Figure EV5 Source Data [file 44319_2026_786_MOESM14_ESM.zip › Figure EV5 Source Data/EV5K/Venus_LINC-DN.tif]

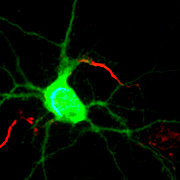

Supplement: Supplementary file 14 — Figure EV5 Source Data [file 44319_2026_786_MOESM14_ESM.zip › Figure EV5 Source Data/EV5K/Merge_LINC-DN.tif]

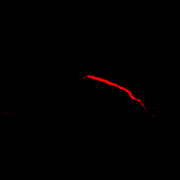

Supplement: Supplementary file 14 — Figure EV5 Source Data [file 44319_2026_786_MOESM14_ESM.zip › Figure EV5 Source Data/EV5O/Ankyrin-G_NT-NaCl.tif]

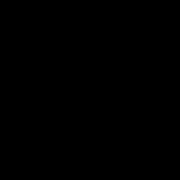

Supplement: Supplementary file 14 — Figure EV5 Source Data [file 44319_2026_786_MOESM14_ESM.zip › Figure EV5 Source Data/EV5O/HA_NT-NaCl.tif]

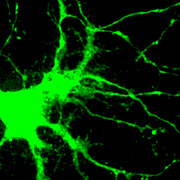

Supplement: Supplementary file 14 — Figure EV5 Source Data [file 44319_2026_786_MOESM14_ESM.zip › Figure EV5 Source Data/EV5O/Venus_NT-NaCl.tif]

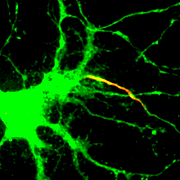

Supplement: Supplementary file 14 — Figure EV5 Source Data [file 44319_2026_786_MOESM14_ESM.zip › Figure EV5 Source Data/EV5O/Merge_NT-NaCl.tif]

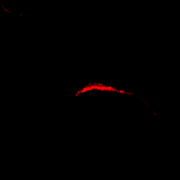

Supplement: Supplementary file 14 — Figure EV5 Source Data [file 44319_2026_786_MOESM14_ESM.zip › Figure EV5 Source Data/EV5O/Ankyrin-G_NT-KCl.tif]

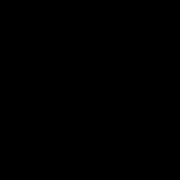

Supplement: Supplementary file 14 — Figure EV5 Source Data [file 44319_2026_786_MOESM14_ESM.zip › Figure EV5 Source Data/EV5O/HA_NT-KCl.tif]

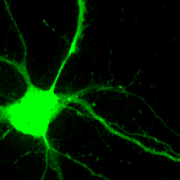

Supplement: Supplementary file 14 — Figure EV5 Source Data [file 44319_2026_786_MOESM14_ESM.zip › Figure EV5 Source Data/EV5O/Venus_NT-KCl.tif]

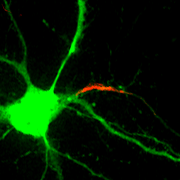

Supplement: Supplementary file 14 — Figure EV5 Source Data [file 44319_2026_786_MOESM14_ESM.zip › Figure EV5 Source Data/EV5O/Merge_NT-KCl.tif]

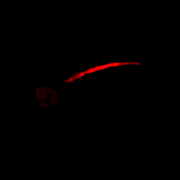

Supplement: Supplementary file 14 — Figure EV5 Source Data [file 44319_2026_786_MOESM14_ESM.zip › Figure EV5 Source Data/EV5O/Ankyrin-G_4-OHT-NaCl.tif]

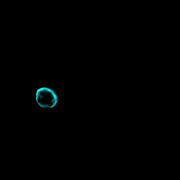

Supplement: Supplementary file 14 — Figure EV5 Source Data [file 44319_2026_786_MOESM14_ESM.zip › Figure EV5 Source Data/EV5O/HA_4-OHT-NaCl.tif]

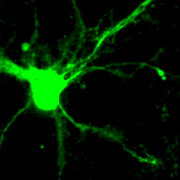

Supplement: Supplementary file 14 — Figure EV5 Source Data [file 44319_2026_786_MOESM14_ESM.zip › Figure EV5 Source Data/EV5O/Venus_4-OHT-NaCl.tif]

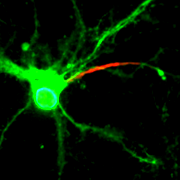

Supplement: Supplementary file 14 — Figure EV5 Source Data [file 44319_2026_786_MOESM14_ESM.zip › Figure EV5 Source Data/EV5O/Merge_4-OHT-NaCl.tif]

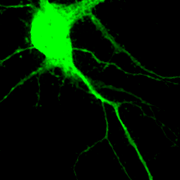

Supplement: Supplementary file 14 — Figure EV5 Source Data [file 44319_2026_786_MOESM14_ESM.zip › Figure EV5 Source Data/EV5O/Venus_4-OHT-KCl.tif]

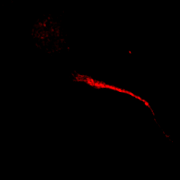

Supplement: Supplementary file 14 — Figure EV5 Source Data [file 44319_2026_786_MOESM14_ESM.zip › Figure EV5 Source Data/EV5O/Ankyrin-G_4-OHT-KCl.tif]

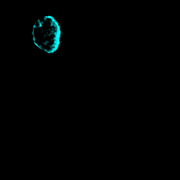

Supplement: Supplementary file 14 — Figure EV5 Source Data [file 44319_2026_786_MOESM14_ESM.zip › Figure EV5 Source Data/EV5O/HA_4-OHT-KCl.tif]

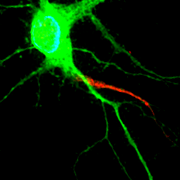

Supplement: Supplementary file 14 — Figure EV5 Source Data [file 44319_2026_786_MOESM14_ESM.zip › Figure EV5 Source Data/EV5O/Merge_4-OHT-KCl.tif]

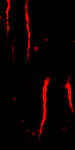

Supplement: Supplementary file 15 — Figure EV6 Source Data [file 44319_2026_786_MOESM15_ESM.zip › Figure EV6 Source Data/EV6H/Ankyrin-G_3M NV.tif]

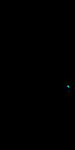

Supplement: Supplementary file 15 — Figure EV6 Source Data [file 44319_2026_786_MOESM15_ESM.zip › Figure EV6 Source Data/EV6H/HA_3M NV.tif]

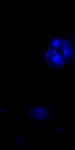

Supplement: Supplementary file 15 — Figure EV6 Source Data [file 44319_2026_786_MOESM15_ESM.zip › Figure EV6 Source Data/EV6H/Hoechst_3M NV.tif]

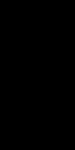

Supplement: Supplementary file 15 — Figure EV6 Source Data [file 44319_2026_786_MOESM15_ESM.zip › Figure EV6 Source Data/EV6H/Venus_3M NV.tif]

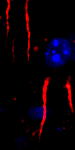

Supplement: Supplementary file 15 — Figure EV6 Source Data [file 44319_2026_786_MOESM15_ESM.zip › Figure EV6 Source Data/EV6H/Merge_3M NV.tif]

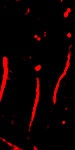

Supplement: Supplementary file 15 — Figure EV6 Source Data [file 44319_2026_786_MOESM15_ESM.zip › Figure EV6 Source Data/EV6H/Ankyrin-G_3M Control.tif]

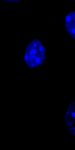

Supplement: Supplementary file 15 — Figure EV6 Source Data [file 44319_2026_786_MOESM15_ESM.zip › Figure EV6 Source Data/EV6H/Hoechst_3M Control.tif]

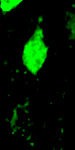

Supplement: Supplementary file 15 — Figure EV6 Source Data [file 44319_2026_786_MOESM15_ESM.zip › Figure EV6 Source Data/EV6H/Venus_3M Control.tif]

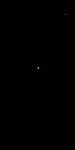

Supplement: Supplementary file 15 — Figure EV6 Source Data [file 44319_2026_786_MOESM15_ESM.zip › Figure EV6 Source Data/EV6H/HA_3M Control.tif]

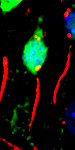

Supplement: Supplementary file 15 — Figure EV6 Source Data [file 44319_2026_786_MOESM15_ESM.zip › Figure EV6 Source Data/EV6H/Merge_3M Control.tif]

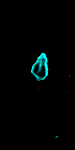

Supplement: Supplementary file 15 — Figure EV6 Source Data [file 44319_2026_786_MOESM15_ESM.zip › Figure EV6 Source Data/EV6H/HA_3M LINC-DN.tif]

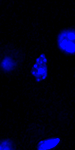

Supplement: Supplementary file 15 — Figure EV6 Source Data [file 44319_2026_786_MOESM15_ESM.zip › Figure EV6 Source Data/EV6H/Hoechst_3M LINC-DN.tif]

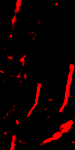

Supplement: Supplementary file 15 — Figure EV6 Source Data [file 44319_2026_786_MOESM15_ESM.zip › Figure EV6 Source Data/EV6H/Ankyrin-G_3M LINC-DN.tif]

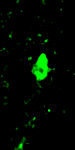

Supplement: Supplementary file 15 — Figure EV6 Source Data [file 44319_2026_786_MOESM15_ESM.zip › Figure EV6 Source Data/EV6H/Venus_3M LINC-DN.tif]

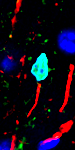

Supplement: Supplementary file 15 — Figure EV6 Source Data [file 44319_2026_786_MOESM15_ESM.zip › Figure EV6 Source Data/EV6H/Merge_3M LINC-DN.tif]

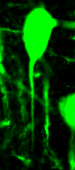

Supplement: Supplementary file 15 — Figure EV6 Source Data [file 44319_2026_786_MOESM15_ESM.zip › Figure EV6 Source Data/EV6B/Venus_Control.tif]

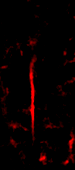

Supplement: Supplementary file 15 — Figure EV6 Source Data [file 44319_2026_786_MOESM15_ESM.zip › Figure EV6 Source Data/EV6B/Ankyrin-G_Control.tif]

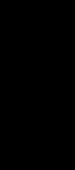

Supplement: Supplementary file 15 — Figure EV6 Source Data [file 44319_2026_786_MOESM15_ESM.zip › Figure EV6 Source Data/EV6B/HA_Control.tif]

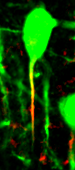

Supplement: Supplementary file 15 — Figure EV6 Source Data [file 44319_2026_786_MOESM15_ESM.zip › Figure EV6 Source Data/EV6B/Merge_Control.tif]

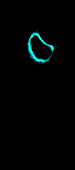

Supplement: Supplementary file 15 — Figure EV6 Source Data [file 44319_2026_786_MOESM15_ESM.zip › Figure EV6 Source Data/EV6B/HA_LINC-DN.tif]

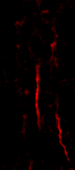

Supplement: Supplementary file 15 — Figure EV6 Source Data [file 44319_2026_786_MOESM15_ESM.zip › Figure EV6 Source Data/EV6B/Ankyrin-G_LINC-DN.tif]

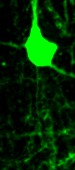

Supplement: Supplementary file 15 — Figure EV6 Source Data [file 44319_2026_786_MOESM15_ESM.zip › Figure EV6 Source Data/EV6B/Venus_LINC-DN.tif]

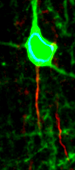

Supplement: Supplementary file 15 — Figure EV6 Source Data [file 44319_2026_786_MOESM15_ESM.zip › Figure EV6 Source Data/EV6B/Merge_LINC-DN.tif]

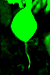

Supplement: Supplementary file 15 — Figure EV6 Source Data [file 44319_2026_786_MOESM15_ESM.zip › Figure EV6 Source Data/EV6E/Venus_Control.tif]

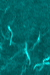

Supplement: Supplementary file 15 — Figure EV6 Source Data [file 44319_2026_786_MOESM15_ESM.zip › Figure EV6 Source Data/EV6E/HA_Control.tif]

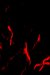

Supplement: Supplementary file 15 — Figure EV6 Source Data [file 44319_2026_786_MOESM15_ESM.zip › Figure EV6 Source Data/EV6E/Ankyrin-G_Control.tif]

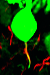

Supplement: Supplementary file 15 — Figure EV6 Source Data [file 44319_2026_786_MOESM15_ESM.zip › Figure EV6 Source Data/EV6E/Merge_Control.tif]

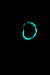

Supplement: Supplementary file 15 — Figure EV6 Source Data [file 44319_2026_786_MOESM15_ESM.zip › Figure EV6 Source Data/EV6E/HA_LINC-DN.tif]

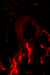

Supplement: Supplementary file 15 — Figure EV6 Source Data [file 44319_2026_786_MOESM15_ESM.zip › Figure EV6 Source Data/EV6E/Ankyrin-G_LINC-DN.tif]

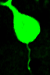

Supplement: Supplementary file 15 — Figure EV6 Source Data [file 44319_2026_786_MOESM15_ESM.zip › Figure EV6 Source Data/EV6E/Venus_LINC-DN.tif]

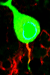

Supplement: Supplementary file 15 — Figure EV6 Source Data [file 44319_2026_786_MOESM15_ESM.zip › Figure EV6 Source Data/EV6E/Merge_LINC-DN.tif]

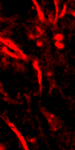

Supplement: Supplementary file 15 — Figure EV6 Source Data [file 44319_2026_786_MOESM15_ESM.zip › Figure EV6 Source Data/EV6L/Ankyrin-G_3M LINC-DN.tif]
