# Supplementary figures and images for: Age-related decline in nuclear envelope LINC complex drives neuronal aging via axon initial segment dysfunction (part 6 of 9)
Source: EMBO Rep. 2026 May 22;27(13):3788–825. doi: 10.1038/s44319-026-00786-5 (PMC13354796; doi:10.1038/s44319-026-00786-5)

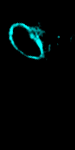

Supplement: Supplementary file 15 — Figure EV6 Source Data [file 44319_2026_786_MOESM15_ESM.zip › Figure EV6 Source Data/EV6L/HA_3M LINC-DN.tif]

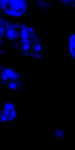

Supplement: Supplementary file 15 — Figure EV6 Source Data [file 44319_2026_786_MOESM15_ESM.zip › Figure EV6 Source Data/EV6L/Hoechst_3M LINC-DN.tif]

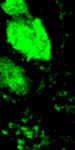

Supplement: Supplementary file 15 — Figure EV6 Source Data [file 44319_2026_786_MOESM15_ESM.zip › Figure EV6 Source Data/EV6L/Venus_3M LINC-DN.tif]

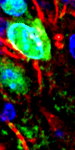

Supplement: Supplementary file 15 — Figure EV6 Source Data [file 44319_2026_786_MOESM15_ESM.zip › Figure EV6 Source Data/EV6L/Merge_3M LINC-DN.tif]

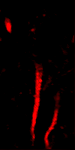

Supplement: Supplementary file 15 — Figure EV6 Source Data [file 44319_2026_786_MOESM15_ESM.zip › Figure EV6 Source Data/EV6L/Ankyrin-G_3M Control.tif]

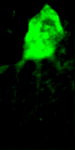

Supplement: Supplementary file 15 — Figure EV6 Source Data [file 44319_2026_786_MOESM15_ESM.zip › Figure EV6 Source Data/EV6L/Venus_3M Control.tif]

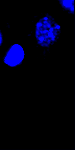

Supplement: Supplementary file 15 — Figure EV6 Source Data [file 44319_2026_786_MOESM15_ESM.zip › Figure EV6 Source Data/EV6L/Hoechst_3M Control.tif]

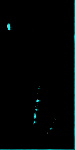

Supplement: Supplementary file 15 — Figure EV6 Source Data [file 44319_2026_786_MOESM15_ESM.zip › Figure EV6 Source Data/EV6L/HA_3M Control.tif]

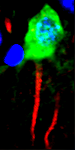

Supplement: Supplementary file 15 — Figure EV6 Source Data [file 44319_2026_786_MOESM15_ESM.zip › Figure EV6 Source Data/EV6L/Merge_3M Control.tif]

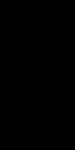

Supplement: Supplementary file 15 — Figure EV6 Source Data [file 44319_2026_786_MOESM15_ESM.zip › Figure EV6 Source Data/EV6L/HA_3M NV.tif]

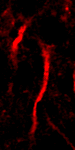

Supplement: Supplementary file 15 — Figure EV6 Source Data [file 44319_2026_786_MOESM15_ESM.zip › Figure EV6 Source Data/EV6L/Ankyrin-G_3M NV.tif]

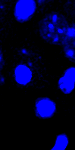

Supplement: Supplementary file 15 — Figure EV6 Source Data [file 44319_2026_786_MOESM15_ESM.zip › Figure EV6 Source Data/EV6L/Hoechst_3M NV.tif]

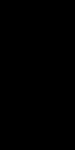

Supplement: Supplementary file 15 — Figure EV6 Source Data [file 44319_2026_786_MOESM15_ESM.zip › Figure EV6 Source Data/EV6L/Venus_3M NV.tif]

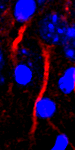

Supplement: Supplementary file 15 — Figure EV6 Source Data [file 44319_2026_786_MOESM15_ESM.zip › Figure EV6 Source Data/EV6L/Merge_3M NV.tif]

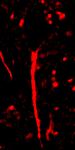

Supplement: Supplementary file 15 — Figure EV6 Source Data [file 44319_2026_786_MOESM15_ESM.zip › Figure EV6 Source Data/EV6J/Ankyrin-G_3M NV.tif]

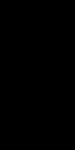

Supplement: Supplementary file 15 — Figure EV6 Source Data [file 44319_2026_786_MOESM15_ESM.zip › Figure EV6 Source Data/EV6J/HA_3M NV.tif]

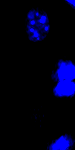

Supplement: Supplementary file 15 — Figure EV6 Source Data [file 44319_2026_786_MOESM15_ESM.zip › Figure EV6 Source Data/EV6J/Hoechst_3M NV.tif]

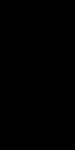

Supplement: Supplementary file 15 — Figure EV6 Source Data [file 44319_2026_786_MOESM15_ESM.zip › Figure EV6 Source Data/EV6J/Venus_3M NV.tif]

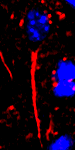

Supplement: Supplementary file 15 — Figure EV6 Source Data [file 44319_2026_786_MOESM15_ESM.zip › Figure EV6 Source Data/EV6J/Merge_3M NV.tif]

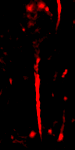

Supplement: Supplementary file 15 — Figure EV6 Source Data [file 44319_2026_786_MOESM15_ESM.zip › Figure EV6 Source Data/EV6J/Ankyrin-G_3M Control.tif]

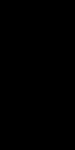

Supplement: Supplementary file 15 — Figure EV6 Source Data [file 44319_2026_786_MOESM15_ESM.zip › Figure EV6 Source Data/EV6J/HA_3M Control.tif]

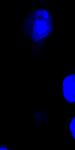

Supplement: Supplementary file 15 — Figure EV6 Source Data [file 44319_2026_786_MOESM15_ESM.zip › Figure EV6 Source Data/EV6J/Hoechst_3M Control.tif]

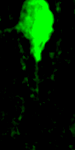

Supplement: Supplementary file 15 — Figure EV6 Source Data [file 44319_2026_786_MOESM15_ESM.zip › Figure EV6 Source Data/EV6J/Venus_3M Control.tif]

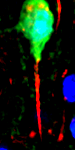

Supplement: Supplementary file 15 — Figure EV6 Source Data [file 44319_2026_786_MOESM15_ESM.zip › Figure EV6 Source Data/EV6J/Merge_3M Control.tif]

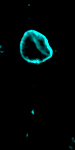

Supplement: Supplementary file 15 — Figure EV6 Source Data [file 44319_2026_786_MOESM15_ESM.zip › Figure EV6 Source Data/EV6J/HA_3M LINC-DN.tif]

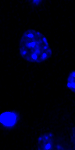

Supplement: Supplementary file 15 — Figure EV6 Source Data [file 44319_2026_786_MOESM15_ESM.zip › Figure EV6 Source Data/EV6J/Hoechst_3M LINC-DN.tif]

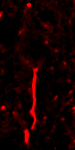

Supplement: Supplementary file 15 — Figure EV6 Source Data [file 44319_2026_786_MOESM15_ESM.zip › Figure EV6 Source Data/EV6J/Ankyrin-G_3M LINC-DN.tif]

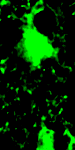

Supplement: Supplementary file 15 — Figure EV6 Source Data [file 44319_2026_786_MOESM15_ESM.zip › Figure EV6 Source Data/EV6J/Venus_3M LINC-DN.tif]

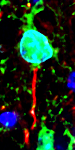

Supplement: Supplementary file 15 — Figure EV6 Source Data [file 44319_2026_786_MOESM15_ESM.zip › Figure EV6 Source Data/EV6J/Merge_3M LINC-DN.tif]

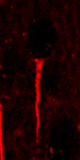

Supplement: Supplementary file 16 — Figure EV7 Source Data [file 44319_2026_786_MOESM16_ESM.zip › Figure EV7 Source Data/EV7G/Ankyrin-G_3M NV.tif]

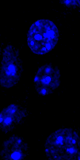

Supplement: Supplementary file 16 — Figure EV7 Source Data [file 44319_2026_786_MOESM16_ESM.zip › Figure EV7 Source Data/EV7G/Hoechst_3M NV.tif]

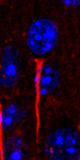

Supplement: Supplementary file 16 — Figure EV7 Source Data [file 44319_2026_786_MOESM16_ESM.zip › Figure EV7 Source Data/EV7G/Merge_3M NV.tif]

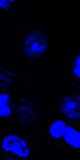

Supplement: Supplementary file 16 — Figure EV7 Source Data [file 44319_2026_786_MOESM16_ESM.zip › Figure EV7 Source Data/EV7G/Hoechst_23M NV.tif]

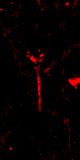

Supplement: Supplementary file 16 — Figure EV7 Source Data [file 44319_2026_786_MOESM16_ESM.zip › Figure EV7 Source Data/EV7G/Ankyrin-G_23M NV.tif]

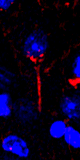

Supplement: Supplementary file 16 — Figure EV7 Source Data [file 44319_2026_786_MOESM16_ESM.zip › Figure EV7 Source Data/EV7G/Merge_23M NV.tif]

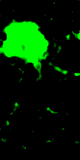

Supplement: Supplementary file 16 — Figure EV7 Source Data [file 44319_2026_786_MOESM16_ESM.zip › Figure EV7 Source Data/EV7G/Venus_23M Control.tif]

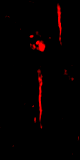

Supplement: Supplementary file 16 — Figure EV7 Source Data [file 44319_2026_786_MOESM16_ESM.zip › Figure EV7 Source Data/EV7G/Ankyrin-G_23M Control.tif]

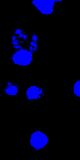

Supplement: Supplementary file 16 — Figure EV7 Source Data [file 44319_2026_786_MOESM16_ESM.zip › Figure EV7 Source Data/EV7G/Hoechst_23M Control.tif]

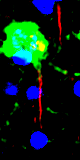

Supplement: Supplementary file 16 — Figure EV7 Source Data [file 44319_2026_786_MOESM16_ESM.zip › Figure EV7 Source Data/EV7G/Merge_23M Control.tif]

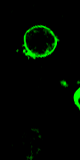

Supplement: Supplementary file 16 — Figure EV7 Source Data [file 44319_2026_786_MOESM16_ESM.zip › Figure EV7 Source Data/EV7G/HA_23M + Sun1.tif]

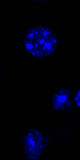

Supplement: Supplementary file 16 — Figure EV7 Source Data [file 44319_2026_786_MOESM16_ESM.zip › Figure EV7 Source Data/EV7G/Hoechst_23M + Sun1.tif]

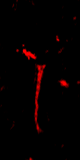

Supplement: Supplementary file 16 — Figure EV7 Source Data [file 44319_2026_786_MOESM16_ESM.zip › Figure EV7 Source Data/EV7G/Ankyrin-G_23M + Sun1.tif]

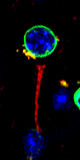

Supplement: Supplementary file 16 — Figure EV7 Source Data [file 44319_2026_786_MOESM16_ESM.zip › Figure EV7 Source Data/EV7G/Merge_23M + Sun1.tif]

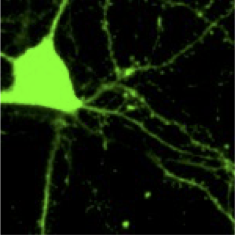

Supplement: Supplementary file 16 — Figure EV7 Source Data [file 44319_2026_786_MOESM16_ESM.zip › Figure EV7 Source Data/EV7A/Venus_Control.tif]

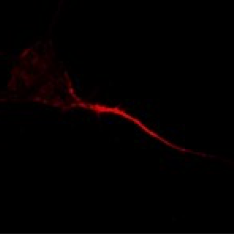

Supplement: Supplementary file 16 — Figure EV7 Source Data [file 44319_2026_786_MOESM16_ESM.zip › Figure EV7 Source Data/EV7A/Ankyrin-G_Control.tif]

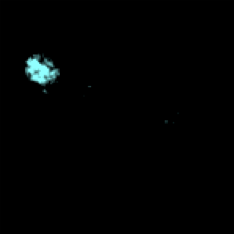

Supplement: Supplementary file 16 — Figure EV7 Source Data [file 44319_2026_786_MOESM16_ESM.zip › Figure EV7 Source Data/EV7A/HA_Control.tif]

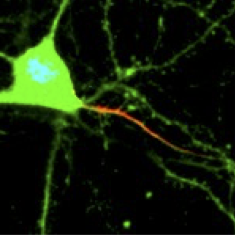

Supplement: Supplementary file 16 — Figure EV7 Source Data [file 44319_2026_786_MOESM16_ESM.zip › Figure EV7 Source Data/EV7A/Merge_Control.tif]

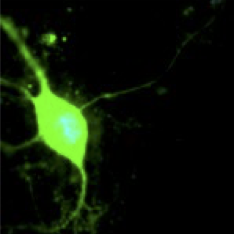

Supplement: Supplementary file 16 — Figure EV7 Source Data [file 44319_2026_786_MOESM16_ESM.zip › Figure EV7 Source Data/EV7A/Venus_AnkG KO.tif]

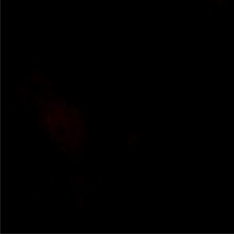

Supplement: Supplementary file 16 — Figure EV7 Source Data [file 44319_2026_786_MOESM16_ESM.zip › Figure EV7 Source Data/EV7A/Ankyrin-G_AnkG KO.tif]

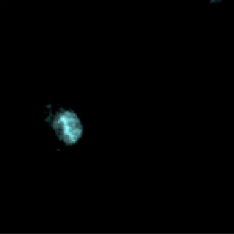

Supplement: Supplementary file 16 — Figure EV7 Source Data [file 44319_2026_786_MOESM16_ESM.zip › Figure EV7 Source Data/EV7A/HA_AnkG KO.tif]

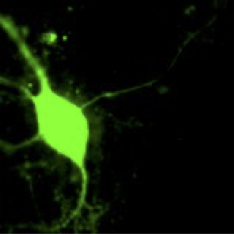

Supplement: Supplementary file 16 — Figure EV7 Source Data [file 44319_2026_786_MOESM16_ESM.zip › Figure EV7 Source Data/EV7A/Merge_AnkG KO.tif]

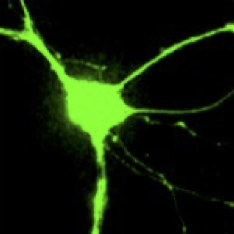

Supplement: Supplementary file 16 — Figure EV7 Source Data [file 44319_2026_786_MOESM16_ESM.zip › Figure EV7 Source Data/EV7C/Venus_Control.tif]

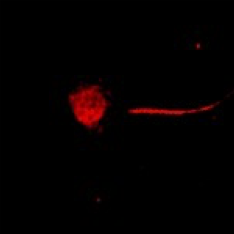

Supplement: Supplementary file 16 — Figure EV7 Source Data [file 44319_2026_786_MOESM16_ESM.zip › Figure EV7 Source Data/EV7C/pan-Nav_Control.tif]

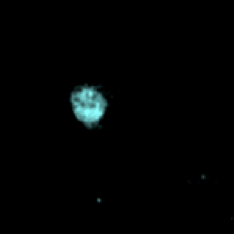

Supplement: Supplementary file 16 — Figure EV7 Source Data [file 44319_2026_786_MOESM16_ESM.zip › Figure EV7 Source Data/EV7C/HA_Control.tif]

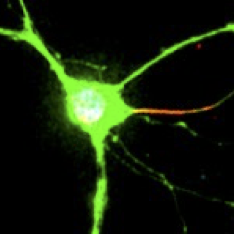

Supplement: Supplementary file 16 — Figure EV7 Source Data [file 44319_2026_786_MOESM16_ESM.zip › Figure EV7 Source Data/EV7C/Merge_Control.tif]

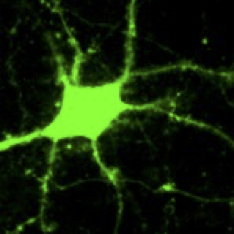

Supplement: Supplementary file 16 — Figure EV7 Source Data [file 44319_2026_786_MOESM16_ESM.zip › Figure EV7 Source Data/EV7C/Venus_AnkG KO.tif]

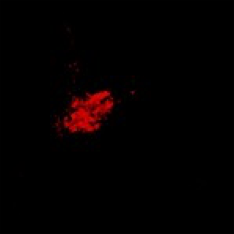

Supplement: Supplementary file 16 — Figure EV7 Source Data [file 44319_2026_786_MOESM16_ESM.zip › Figure EV7 Source Data/EV7C/pan-Nav_AnkG KO.tif]

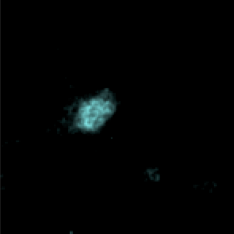

Supplement: Supplementary file 16 — Figure EV7 Source Data [file 44319_2026_786_MOESM16_ESM.zip › Figure EV7 Source Data/EV7C/HA_AnkG KO.tif]

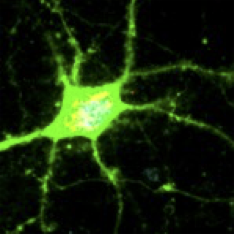

Supplement: Supplementary file 16 — Figure EV7 Source Data [file 44319_2026_786_MOESM16_ESM.zip › Figure EV7 Source Data/EV7C/Merge_AnkG KO.tif]

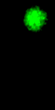

Supplement: Supplementary file 16 — Figure EV7 Source Data [file 44319_2026_786_MOESM16_ESM.zip › Figure EV7 Source Data/EV7E/V5_20M + AnkG KO.tif]

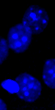

Supplement: Supplementary file 16 — Figure EV7 Source Data [file 44319_2026_786_MOESM16_ESM.zip › Figure EV7 Source Data/EV7E/Hoechst_20M + AnkG KO.tif]

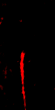

Supplement: Supplementary file 16 — Figure EV7 Source Data [file 44319_2026_786_MOESM16_ESM.zip › Figure EV7 Source Data/EV7E/Ankyrin-G_20M + AnkG KO.tif]

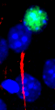

Supplement: Supplementary file 16 — Figure EV7 Source Data [file 44319_2026_786_MOESM16_ESM.zip › Figure EV7 Source Data/EV7E/Merge_20M + AnkG KO.tif]

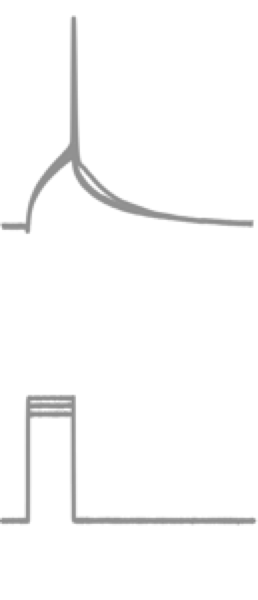

Supplement: Supplementary file 16 — Figure EV7 Source Data [file 44319_2026_786_MOESM16_ESM.zip › Figure EV7 Source Data/EV7I/3M NV.tiff]

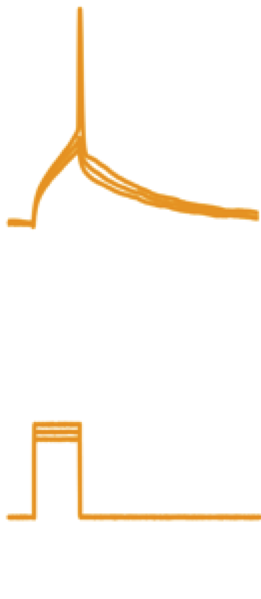

Supplement: Supplementary file 16 — Figure EV7 Source Data [file 44319_2026_786_MOESM16_ESM.zip › Figure EV7 Source Data/EV7I/23M + Sun1.tiff]

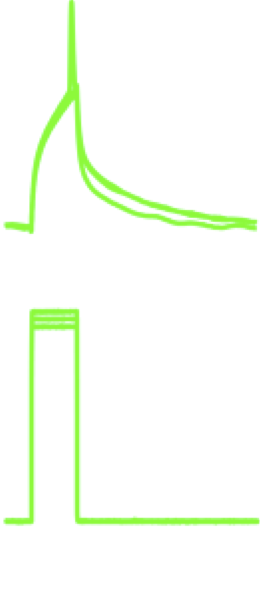

Supplement: Supplementary file 16 — Figure EV7 Source Data [file 44319_2026_786_MOESM16_ESM.zip › Figure EV7 Source Data/EV7I/23M Control.tiff]

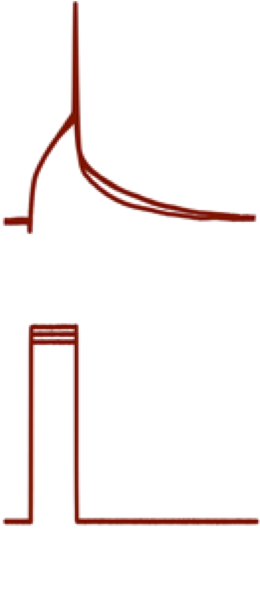

Supplement: Supplementary file 16 — Figure EV7 Source Data [file 44319_2026_786_MOESM16_ESM.zip › Figure EV7 Source Data/EV7I/23M NV.tiff]

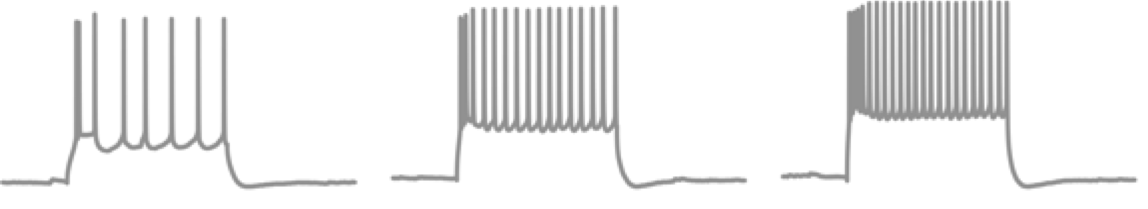

Supplement: Supplementary file 16 — Figure EV7 Source Data [file 44319_2026_786_MOESM16_ESM.zip › Figure EV7 Source Data/EV7K/3M NV.tiff]

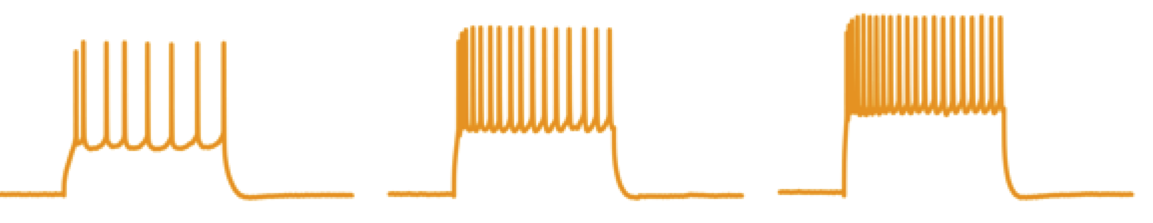

Supplement: Supplementary file 16 — Figure EV7 Source Data [file 44319_2026_786_MOESM16_ESM.zip › Figure EV7 Source Data/EV7K/23M + Sun1.tiff]

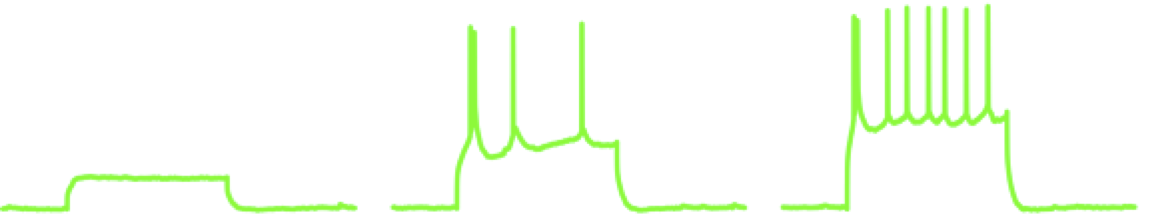

Supplement: Supplementary file 16 — Figure EV7 Source Data [file 44319_2026_786_MOESM16_ESM.zip › Figure EV7 Source Data/EV7K/23M Control.tiff]

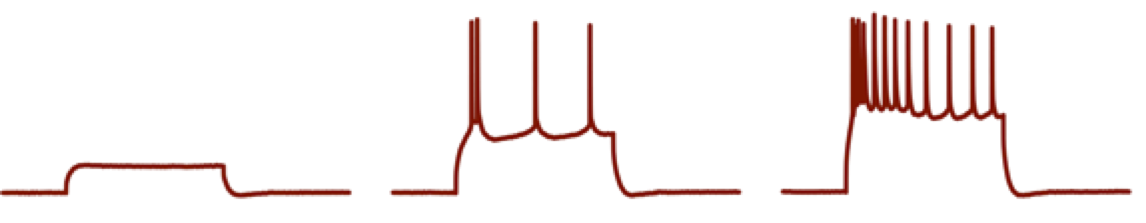

Supplement: Supplementary file 16 — Figure EV7 Source Data [file 44319_2026_786_MOESM16_ESM.zip › Figure EV7 Source Data/EV7K/23M NV.tiff]

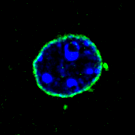

Supplement: Supplementary file 17 — Appendix Figure S1 Source Data [file 44319_2026_786_MOESM17_ESM.zip › Appendix Figure S1 Source Data/S1F/Merge_3M.tif]

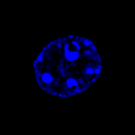

Supplement: Supplementary file 17 — Appendix Figure S1 Source Data [file 44319_2026_786_MOESM17_ESM.zip › Appendix Figure S1 Source Data/S1F/Hoechst_3M.tif]

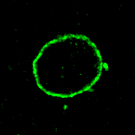

Supplement: Supplementary file 17 — Appendix Figure S1 Source Data [file 44319_2026_786_MOESM17_ESM.zip › Appendix Figure S1 Source Data/S1F/Nesprin-1_3M.tif]

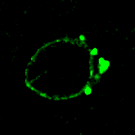

Supplement: Supplementary file 17 — Appendix Figure S1 Source Data [file 44319_2026_786_MOESM17_ESM.zip › Appendix Figure S1 Source Data/S1F/Nesprin-1_12M.tif]

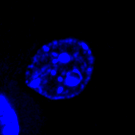

Supplement: Supplementary file 17 — Appendix Figure S1 Source Data [file 44319_2026_786_MOESM17_ESM.zip › Appendix Figure S1 Source Data/S1F/Hoechst_12M.tif]

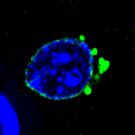

Supplement: Supplementary file 17 — Appendix Figure S1 Source Data [file 44319_2026_786_MOESM17_ESM.zip › Appendix Figure S1 Source Data/S1F/Merge_12M.tif]

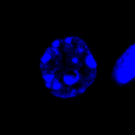

Supplement: Supplementary file 17 — Appendix Figure S1 Source Data [file 44319_2026_786_MOESM17_ESM.zip › Appendix Figure S1 Source Data/S1F/Hoechst_20M.tif]

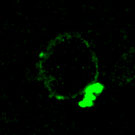

Supplement: Supplementary file 17 — Appendix Figure S1 Source Data [file 44319_2026_786_MOESM17_ESM.zip › Appendix Figure S1 Source Data/S1F/Nesprin-1_20M.tif]

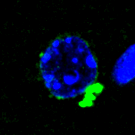

Supplement: Supplementary file 17 — Appendix Figure S1 Source Data [file 44319_2026_786_MOESM17_ESM.zip › Appendix Figure S1 Source Data/S1F/Merge_20M.tif]

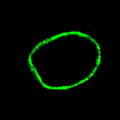

Supplement: Supplementary file 17 — Appendix Figure S1 Source Data [file 44319_2026_786_MOESM17_ESM.zip › Appendix Figure S1 Source Data/S1A/Sun1_Sun1.tif]

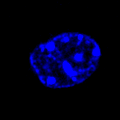

Supplement: Supplementary file 17 — Appendix Figure S1 Source Data [file 44319_2026_786_MOESM17_ESM.zip › Appendix Figure S1 Source Data/S1A/Hoechst_Sun1.tif]

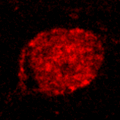

Supplement: Supplementary file 17 — Appendix Figure S1 Source Data [file 44319_2026_786_MOESM17_ESM.zip › Appendix Figure S1 Source Data/S1A/NeuN_Sun1.tif]

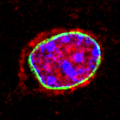

Supplement: Supplementary file 17 — Appendix Figure S1 Source Data [file 44319_2026_786_MOESM17_ESM.zip › Appendix Figure S1 Source Data/S1A/Merge_Sun1.tif]

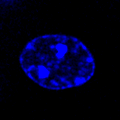

Supplement: Supplementary file 17 — Appendix Figure S1 Source Data [file 44319_2026_786_MOESM17_ESM.zip › Appendix Figure S1 Source Data/S1A/Hoechst_Sun2.tif]

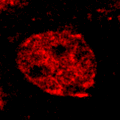

Supplement: Supplementary file 17 — Appendix Figure S1 Source Data [file 44319_2026_786_MOESM17_ESM.zip › Appendix Figure S1 Source Data/S1A/NeuN_Sun2.tif]

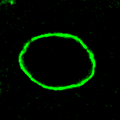

Supplement: Supplementary file 17 — Appendix Figure S1 Source Data [file 44319_2026_786_MOESM17_ESM.zip › Appendix Figure S1 Source Data/S1A/Sun2_Sun2.tif]

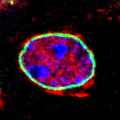

Supplement: Supplementary file 17 — Appendix Figure S1 Source Data [file 44319_2026_786_MOESM17_ESM.zip › Appendix Figure S1 Source Data/S1A/Merge_Sun2.tif]

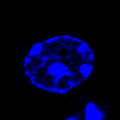

Supplement: Supplementary file 17 — Appendix Figure S1 Source Data [file 44319_2026_786_MOESM17_ESM.zip › Appendix Figure S1 Source Data/S1A/Hoechst_Nesprin-1.tif]

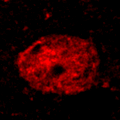

Supplement: Supplementary file 17 — Appendix Figure S1 Source Data [file 44319_2026_786_MOESM17_ESM.zip › Appendix Figure S1 Source Data/S1A/NeuN_Nesprin-1.tif]

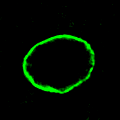

Supplement: Supplementary file 17 — Appendix Figure S1 Source Data [file 44319_2026_786_MOESM17_ESM.zip › Appendix Figure S1 Source Data/S1A/Nesprin-1_Nesprin-1.tif]

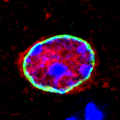

Supplement: Supplementary file 17 — Appendix Figure S1 Source Data [file 44319_2026_786_MOESM17_ESM.zip › Appendix Figure S1 Source Data/S1A/Merge_Nesprin-1.tif]

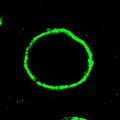

Supplement: Supplementary file 17 — Appendix Figure S1 Source Data [file 44319_2026_786_MOESM17_ESM.zip › Appendix Figure S1 Source Data/S1A/Nesprin-2_Nesprin-2.tif]

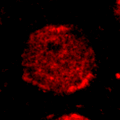

Supplement: Supplementary file 17 — Appendix Figure S1 Source Data [file 44319_2026_786_MOESM17_ESM.zip › Appendix Figure S1 Source Data/S1A/NeuN_Nesprin-2.tif]

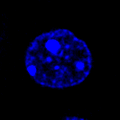

Supplement: Supplementary file 17 — Appendix Figure S1 Source Data [file 44319_2026_786_MOESM17_ESM.zip › Appendix Figure S1 Source Data/S1A/Hoechst_Nesprin-2.tif]

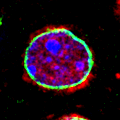

Supplement: Supplementary file 17 — Appendix Figure S1 Source Data [file 44319_2026_786_MOESM17_ESM.zip › Appendix Figure S1 Source Data/S1A/Merge_Nesprin-2.tif]

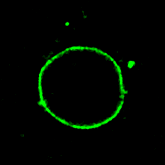

Supplement: Supplementary file 17 — Appendix Figure S1 Source Data [file 44319_2026_786_MOESM17_ESM.zip › Appendix Figure S1 Source Data/S1B/Sun1_3M.tif]

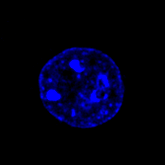

Supplement: Supplementary file 17 — Appendix Figure S1 Source Data [file 44319_2026_786_MOESM17_ESM.zip › Appendix Figure S1 Source Data/S1B/Hoechst_3M.tif]

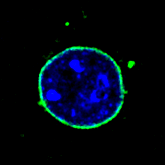

Supplement: Supplementary file 17 — Appendix Figure S1 Source Data [file 44319_2026_786_MOESM17_ESM.zip › Appendix Figure S1 Source Data/S1B/Merge_3M.tif]

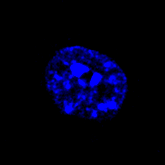

Supplement: Supplementary file 17 — Appendix Figure S1 Source Data [file 44319_2026_786_MOESM17_ESM.zip › Appendix Figure S1 Source Data/S1B/Hoechst_12M.tif]
