# Supplementary figures and images for: Age-related decline in nuclear envelope LINC complex drives neuronal aging via axon initial segment dysfunction (part 7 of 9)
Source: EMBO Rep. 2026 May 22;27(13):3788–825. doi: 10.1038/s44319-026-00786-5 (PMC13354796; doi:10.1038/s44319-026-00786-5)

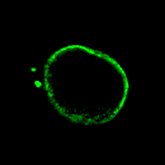

Supplement: Supplementary file 17 — Appendix Figure S1 Source Data [file 44319_2026_786_MOESM17_ESM.zip › Appendix Figure S1 Source Data/S1B/Sun1_12M.tif]

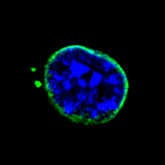

Supplement: Supplementary file 17 — Appendix Figure S1 Source Data [file 44319_2026_786_MOESM17_ESM.zip › Appendix Figure S1 Source Data/S1B/Merge_12M.tif]

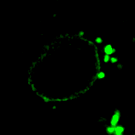

Supplement: Supplementary file 17 — Appendix Figure S1 Source Data [file 44319_2026_786_MOESM17_ESM.zip › Appendix Figure S1 Source Data/S1B/Sun1_20M.tif]

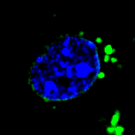

Supplement: Supplementary file 17 — Appendix Figure S1 Source Data [file 44319_2026_786_MOESM17_ESM.zip › Appendix Figure S1 Source Data/S1B/Merge_20M.tif]

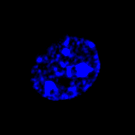

Supplement: Supplementary file 17 — Appendix Figure S1 Source Data [file 44319_2026_786_MOESM17_ESM.zip › Appendix Figure S1 Source Data/S1B/Hoechst_20M.tif]

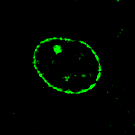

Supplement: Supplementary file 17 — Appendix Figure S1 Source Data [file 44319_2026_786_MOESM17_ESM.zip › Appendix Figure S1 Source Data/S1D/Sun2_3M.tif]

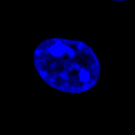

Supplement: Supplementary file 17 — Appendix Figure S1 Source Data [file 44319_2026_786_MOESM17_ESM.zip › Appendix Figure S1 Source Data/S1D/Hoechst_3M.tif]

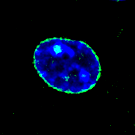

Supplement: Supplementary file 17 — Appendix Figure S1 Source Data [file 44319_2026_786_MOESM17_ESM.zip › Appendix Figure S1 Source Data/S1D/Merge_3M.tif]

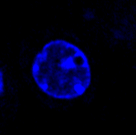

Supplement: Supplementary file 17 — Appendix Figure S1 Source Data [file 44319_2026_786_MOESM17_ESM.zip › Appendix Figure S1 Source Data/S1D/Hoechst_12M.tif]

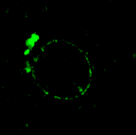

Supplement: Supplementary file 17 — Appendix Figure S1 Source Data [file 44319_2026_786_MOESM17_ESM.zip › Appendix Figure S1 Source Data/S1D/Sun2_12M.tif]

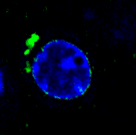

Supplement: Supplementary file 17 — Appendix Figure S1 Source Data [file 44319_2026_786_MOESM17_ESM.zip › Appendix Figure S1 Source Data/S1D/Merge_12M.tif]

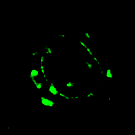

Supplement: Supplementary file 17 — Appendix Figure S1 Source Data [file 44319_2026_786_MOESM17_ESM.zip › Appendix Figure S1 Source Data/S1D/Sun2_20M.tif]

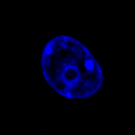

Supplement: Supplementary file 17 — Appendix Figure S1 Source Data [file 44319_2026_786_MOESM17_ESM.zip › Appendix Figure S1 Source Data/S1D/Hoechst_20M.tif]

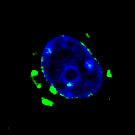

Supplement: Supplementary file 17 — Appendix Figure S1 Source Data [file 44319_2026_786_MOESM17_ESM.zip › Appendix Figure S1 Source Data/S1D/Merge_20M.tif]

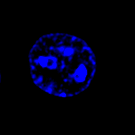

Supplement: Supplementary file 17 — Appendix Figure S1 Source Data [file 44319_2026_786_MOESM17_ESM.zip › Appendix Figure S1 Source Data/S1H/Hoechst_3M.tif]

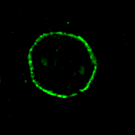

Supplement: Supplementary file 17 — Appendix Figure S1 Source Data [file 44319_2026_786_MOESM17_ESM.zip › Appendix Figure S1 Source Data/S1H/Nesprin-2_3M.tif]

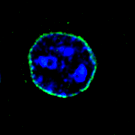

Supplement: Supplementary file 17 — Appendix Figure S1 Source Data [file 44319_2026_786_MOESM17_ESM.zip › Appendix Figure S1 Source Data/S1H/Merge_3M.tif]

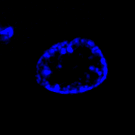

Supplement: Supplementary file 17 — Appendix Figure S1 Source Data [file 44319_2026_786_MOESM17_ESM.zip › Appendix Figure S1 Source Data/S1H/Hoechst_12M.tif]

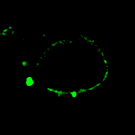

Supplement: Supplementary file 17 — Appendix Figure S1 Source Data [file 44319_2026_786_MOESM17_ESM.zip › Appendix Figure S1 Source Data/S1H/Nesprin-2_12M.tif]

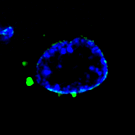

Supplement: Supplementary file 17 — Appendix Figure S1 Source Data [file 44319_2026_786_MOESM17_ESM.zip › Appendix Figure S1 Source Data/S1H/Merge_12M.tif]

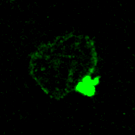

Supplement: Supplementary file 17 — Appendix Figure S1 Source Data [file 44319_2026_786_MOESM17_ESM.zip › Appendix Figure S1 Source Data/S1H/Nesprin-2_20M.tif]

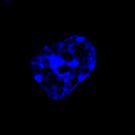

Supplement: Supplementary file 17 — Appendix Figure S1 Source Data [file 44319_2026_786_MOESM17_ESM.zip › Appendix Figure S1 Source Data/S1H/Hoechst_20M.tif]

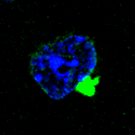

Supplement: Supplementary file 17 — Appendix Figure S1 Source Data [file 44319_2026_786_MOESM17_ESM.zip › Appendix Figure S1 Source Data/S1H/Merge_20M.tif]

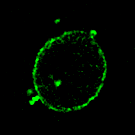

Supplement: Supplementary file 17 — Appendix Figure S1 Source Data [file 44319_2026_786_MOESM17_ESM.zip › Appendix Figure S1 Source Data/S1J/Sun1_3M.tif]

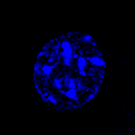

Supplement: Supplementary file 17 — Appendix Figure S1 Source Data [file 44319_2026_786_MOESM17_ESM.zip › Appendix Figure S1 Source Data/S1J/Hoechst_3M.tif]

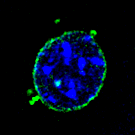

Supplement: Supplementary file 17 — Appendix Figure S1 Source Data [file 44319_2026_786_MOESM17_ESM.zip › Appendix Figure S1 Source Data/S1J/Merge_3M.tif]

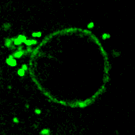

Supplement: Supplementary file 17 — Appendix Figure S1 Source Data [file 44319_2026_786_MOESM17_ESM.zip › Appendix Figure S1 Source Data/S1J/Sun1_12M.tif]

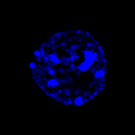

Supplement: Supplementary file 17 — Appendix Figure S1 Source Data [file 44319_2026_786_MOESM17_ESM.zip › Appendix Figure S1 Source Data/S1J/Hoechst_12M.tif]

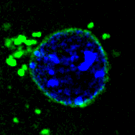

Supplement: Supplementary file 17 — Appendix Figure S1 Source Data [file 44319_2026_786_MOESM17_ESM.zip › Appendix Figure S1 Source Data/S1J/Merge_12M.tif]

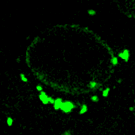

Supplement: Supplementary file 17 — Appendix Figure S1 Source Data [file 44319_2026_786_MOESM17_ESM.zip › Appendix Figure S1 Source Data/S1J/Sun1_20M.tif]

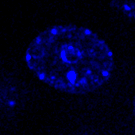

Supplement: Supplementary file 17 — Appendix Figure S1 Source Data [file 44319_2026_786_MOESM17_ESM.zip › Appendix Figure S1 Source Data/S1J/Hoechst_20M.tif]

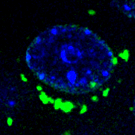

Supplement: Supplementary file 17 — Appendix Figure S1 Source Data [file 44319_2026_786_MOESM17_ESM.zip › Appendix Figure S1 Source Data/S1J/Merge_20M.tif]

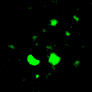

Supplement: Supplementary file 17 — Appendix Figure S1 Source Data [file 44319_2026_786_MOESM17_ESM.zip › Appendix Figure S1 Source Data/S1L/Sun2_12M.tif]

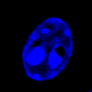

Supplement: Supplementary file 17 — Appendix Figure S1 Source Data [file 44319_2026_786_MOESM17_ESM.zip › Appendix Figure S1 Source Data/S1L/Hoechst_12M.tif]

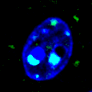

Supplement: Supplementary file 17 — Appendix Figure S1 Source Data [file 44319_2026_786_MOESM17_ESM.zip › Appendix Figure S1 Source Data/S1L/Merge_12M.tif]

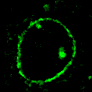

Supplement: Supplementary file 17 — Appendix Figure S1 Source Data [file 44319_2026_786_MOESM17_ESM.zip › Appendix Figure S1 Source Data/S1L/Sun2_3M.tif]

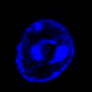

Supplement: Supplementary file 17 — Appendix Figure S1 Source Data [file 44319_2026_786_MOESM17_ESM.zip › Appendix Figure S1 Source Data/S1L/Hoechst_3M.tif]

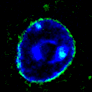

Supplement: Supplementary file 17 — Appendix Figure S1 Source Data [file 44319_2026_786_MOESM17_ESM.zip › Appendix Figure S1 Source Data/S1L/Merge_3M.tif]

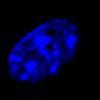

Supplement: Supplementary file 17 — Appendix Figure S1 Source Data [file 44319_2026_786_MOESM17_ESM.zip › Appendix Figure S1 Source Data/S1L/Hoechst_20M.tif]

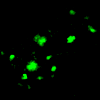

Supplement: Supplementary file 17 — Appendix Figure S1 Source Data [file 44319_2026_786_MOESM17_ESM.zip › Appendix Figure S1 Source Data/S1L/Sun2_20M.tif]

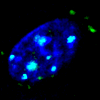

Supplement: Supplementary file 17 — Appendix Figure S1 Source Data [file 44319_2026_786_MOESM17_ESM.zip › Appendix Figure S1 Source Data/S1L/Merge_20M.tif]

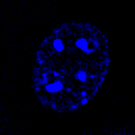

Supplement: Supplementary file 17 — Appendix Figure S1 Source Data [file 44319_2026_786_MOESM17_ESM.zip › Appendix Figure S1 Source Data/S1N/Hoechst_3M.tif]

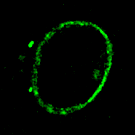

Supplement: Supplementary file 17 — Appendix Figure S1 Source Data [file 44319_2026_786_MOESM17_ESM.zip › Appendix Figure S1 Source Data/S1N/Nesprin-1_3M.tif]

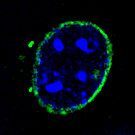

Supplement: Supplementary file 17 — Appendix Figure S1 Source Data [file 44319_2026_786_MOESM17_ESM.zip › Appendix Figure S1 Source Data/S1N/Merge_3M.tif]

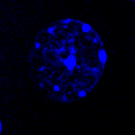

Supplement: Supplementary file 17 — Appendix Figure S1 Source Data [file 44319_2026_786_MOESM17_ESM.zip › Appendix Figure S1 Source Data/S1N/Hoechst_12M.tif]

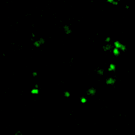

Supplement: Supplementary file 17 — Appendix Figure S1 Source Data [file 44319_2026_786_MOESM17_ESM.zip › Appendix Figure S1 Source Data/S1N/Nesprin-1_12M.tif]

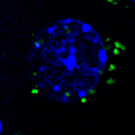

Supplement: Supplementary file 17 — Appendix Figure S1 Source Data [file 44319_2026_786_MOESM17_ESM.zip › Appendix Figure S1 Source Data/S1N/Merge_12M.tif]

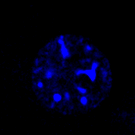

Supplement: Supplementary file 17 — Appendix Figure S1 Source Data [file 44319_2026_786_MOESM17_ESM.zip › Appendix Figure S1 Source Data/S1N/Hoechst_20M.tif]

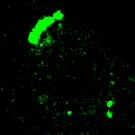

Supplement: Supplementary file 17 — Appendix Figure S1 Source Data [file 44319_2026_786_MOESM17_ESM.zip › Appendix Figure S1 Source Data/S1N/Nesprin-1_20M.tif]

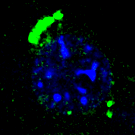

Supplement: Supplementary file 17 — Appendix Figure S1 Source Data [file 44319_2026_786_MOESM17_ESM.zip › Appendix Figure S1 Source Data/S1N/Merge_20M.tif]

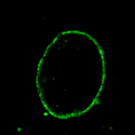

Supplement: Supplementary file 17 — Appendix Figure S1 Source Data [file 44319_2026_786_MOESM17_ESM.zip › Appendix Figure S1 Source Data/S1P/Nesprin-2_3M.tif]

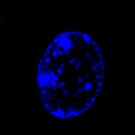

Supplement: Supplementary file 17 — Appendix Figure S1 Source Data [file 44319_2026_786_MOESM17_ESM.zip › Appendix Figure S1 Source Data/S1P/Hoechst_3M.tif]

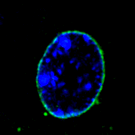

Supplement: Supplementary file 17 — Appendix Figure S1 Source Data [file 44319_2026_786_MOESM17_ESM.zip › Appendix Figure S1 Source Data/S1P/Merge_3M.tif]

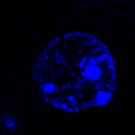

Supplement: Supplementary file 17 — Appendix Figure S1 Source Data [file 44319_2026_786_MOESM17_ESM.zip › Appendix Figure S1 Source Data/S1P/Hoechst_12M.tif]

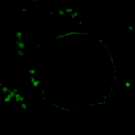

Supplement: Supplementary file 17 — Appendix Figure S1 Source Data [file 44319_2026_786_MOESM17_ESM.zip › Appendix Figure S1 Source Data/S1P/Nesprin-2_12M.tif]

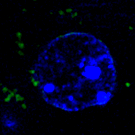

Supplement: Supplementary file 17 — Appendix Figure S1 Source Data [file 44319_2026_786_MOESM17_ESM.zip › Appendix Figure S1 Source Data/S1P/Merge_12M.tif]

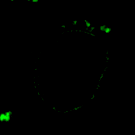

Supplement: Supplementary file 17 — Appendix Figure S1 Source Data [file 44319_2026_786_MOESM17_ESM.zip › Appendix Figure S1 Source Data/S1P/Nesprin-2_20M.tif]

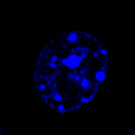

Supplement: Supplementary file 17 — Appendix Figure S1 Source Data [file 44319_2026_786_MOESM17_ESM.zip › Appendix Figure S1 Source Data/S1P/Hoechst_20M.tif]

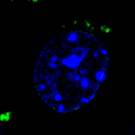

Supplement: Supplementary file 17 — Appendix Figure S1 Source Data [file 44319_2026_786_MOESM17_ESM.zip › Appendix Figure S1 Source Data/S1P/Merge_20M.tif]

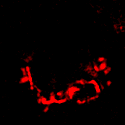

Supplement: Supplementary file 18 — Appendix Figure S2 Source Data [file 44319_2026_786_MOESM18_ESM.zip › Appendix Figure S2 Source Data/S2A/GM130_Sun1.tif]

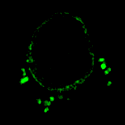

Supplement: Supplementary file 18 — Appendix Figure S2 Source Data [file 44319_2026_786_MOESM18_ESM.zip › Appendix Figure S2 Source Data/S2A/Sun1_Sun1.tif]

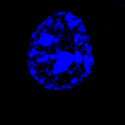

Supplement: Supplementary file 18 — Appendix Figure S2 Source Data [file 44319_2026_786_MOESM18_ESM.zip › Appendix Figure S2 Source Data/S2A/Hoechst_Sun1.tif]

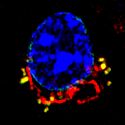

Supplement: Supplementary file 18 — Appendix Figure S2 Source Data [file 44319_2026_786_MOESM18_ESM.zip › Appendix Figure S2 Source Data/S2A/Merge_Sun1.tif]

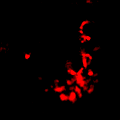

Supplement: Supplementary file 18 — Appendix Figure S2 Source Data [file 44319_2026_786_MOESM18_ESM.zip › Appendix Figure S2 Source Data/S2A/GM130_Sun2.tif]

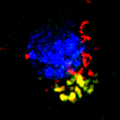

Supplement: Supplementary file 18 — Appendix Figure S2 Source Data [file 44319_2026_786_MOESM18_ESM.zip › Appendix Figure S2 Source Data/S2A/Merge_Sun2.tif]

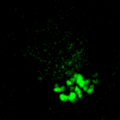

Supplement: Supplementary file 18 — Appendix Figure S2 Source Data [file 44319_2026_786_MOESM18_ESM.zip › Appendix Figure S2 Source Data/S2A/Sun2_Sun2.tif]

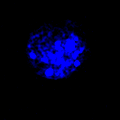

Supplement: Supplementary file 18 — Appendix Figure S2 Source Data [file 44319_2026_786_MOESM18_ESM.zip › Appendix Figure S2 Source Data/S2A/Hoechst_Sun2.tif]

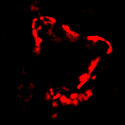

Supplement: Supplementary file 18 — Appendix Figure S2 Source Data [file 44319_2026_786_MOESM18_ESM.zip › Appendix Figure S2 Source Data/S2A/GM130_Nesprin-1.tif]

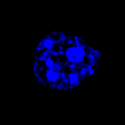

Supplement: Supplementary file 18 — Appendix Figure S2 Source Data [file 44319_2026_786_MOESM18_ESM.zip › Appendix Figure S2 Source Data/S2A/Hoechst_Nesprin-1.tif]

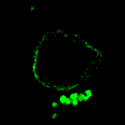

Supplement: Supplementary file 18 — Appendix Figure S2 Source Data [file 44319_2026_786_MOESM18_ESM.zip › Appendix Figure S2 Source Data/S2A/Nesprin-1_Nesprin-1.tif]

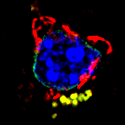

Supplement: Supplementary file 18 — Appendix Figure S2 Source Data [file 44319_2026_786_MOESM18_ESM.zip › Appendix Figure S2 Source Data/S2A/Merge_Nesprin-1.tif]

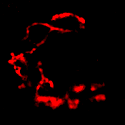

Supplement: Supplementary file 18 — Appendix Figure S2 Source Data [file 44319_2026_786_MOESM18_ESM.zip › Appendix Figure S2 Source Data/S2A/GM130_Nesprin-2.tif]

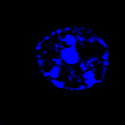

Supplement: Supplementary file 18 — Appendix Figure S2 Source Data [file 44319_2026_786_MOESM18_ESM.zip › Appendix Figure S2 Source Data/S2A/Hoechst_Nesprin-2.tif]

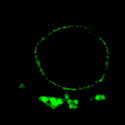

Supplement: Supplementary file 18 — Appendix Figure S2 Source Data [file 44319_2026_786_MOESM18_ESM.zip › Appendix Figure S2 Source Data/S2A/Nesprin-2_Nesprin-2.tif]

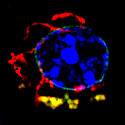

Supplement: Supplementary file 18 — Appendix Figure S2 Source Data [file 44319_2026_786_MOESM18_ESM.zip › Appendix Figure S2 Source Data/S2A/Merge_Nesprin-2.tif]

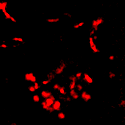

Supplement: Supplementary file 18 — Appendix Figure S2 Source Data [file 44319_2026_786_MOESM18_ESM.zip › Appendix Figure S2 Source Data/S2B/GM130_Lamin B1.tif]

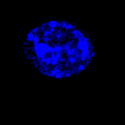

Supplement: Supplementary file 18 — Appendix Figure S2 Source Data [file 44319_2026_786_MOESM18_ESM.zip › Appendix Figure S2 Source Data/S2B/Hoechst_Lamin B1.tif]

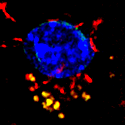

Supplement: Supplementary file 18 — Appendix Figure S2 Source Data [file 44319_2026_786_MOESM18_ESM.zip › Appendix Figure S2 Source Data/S2B/Merge_Lamin B1.tif]

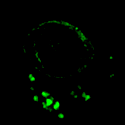

Supplement: Supplementary file 18 — Appendix Figure S2 Source Data [file 44319_2026_786_MOESM18_ESM.zip › Appendix Figure S2 Source Data/S2B/Lamin B1_Lamin B1.tif]

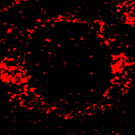

Supplement: Supplementary file 19 — Appendix Figure S3 Source Data [file 44319_2026_786_MOESM19_ESM.zip › Appendix Figure S3 Source Data/S3D/CaMKIIalpha_3M.tif]

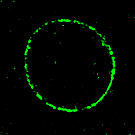

Supplement: Supplementary file 19 — Appendix Figure S3 Source Data [file 44319_2026_786_MOESM19_ESM.zip › Appendix Figure S3 Source Data/S3D/Nesprin-2_3M.tif]

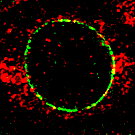

Supplement: Supplementary file 19 — Appendix Figure S3 Source Data [file 44319_2026_786_MOESM19_ESM.zip › Appendix Figure S3 Source Data/S3D/Merge_3M.tif]

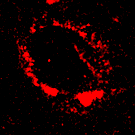

Supplement: Supplementary file 19 — Appendix Figure S3 Source Data [file 44319_2026_786_MOESM19_ESM.zip › Appendix Figure S3 Source Data/S3D/CaMKIIalpha_20M.tif]

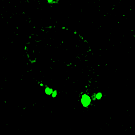

Supplement: Supplementary file 19 — Appendix Figure S3 Source Data [file 44319_2026_786_MOESM19_ESM.zip › Appendix Figure S3 Source Data/S3D/Nesprin-2_20M.tif]

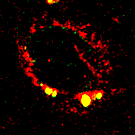

Supplement: Supplementary file 19 — Appendix Figure S3 Source Data [file 44319_2026_786_MOESM19_ESM.zip › Appendix Figure S3 Source Data/S3D/Merge_20M.tif]

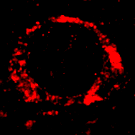

Supplement: Supplementary file 19 — Appendix Figure S3 Source Data [file 44319_2026_786_MOESM19_ESM.zip › Appendix Figure S3 Source Data/S3E/GAD65_3M.tif]

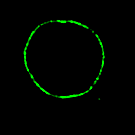

Supplement: Supplementary file 19 — Appendix Figure S3 Source Data [file 44319_2026_786_MOESM19_ESM.zip › Appendix Figure S3 Source Data/S3E/Sun1_3M.tif]

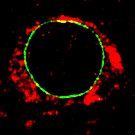

Supplement: Supplementary file 19 — Appendix Figure S3 Source Data [file 44319_2026_786_MOESM19_ESM.zip › Appendix Figure S3 Source Data/S3E/Merge_3M.tif]

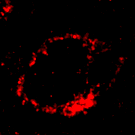

Supplement: Supplementary file 19 — Appendix Figure S3 Source Data [file 44319_2026_786_MOESM19_ESM.zip › Appendix Figure S3 Source Data/S3E/GAD65_20M.tif]

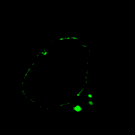

Supplement: Supplementary file 19 — Appendix Figure S3 Source Data [file 44319_2026_786_MOESM19_ESM.zip › Appendix Figure S3 Source Data/S3E/Sun1_20M.tif]

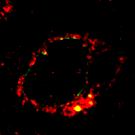

Supplement: Supplementary file 19 — Appendix Figure S3 Source Data [file 44319_2026_786_MOESM19_ESM.zip › Appendix Figure S3 Source Data/S3E/Merge_20M.tif]

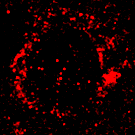

Supplement: Supplementary file 19 — Appendix Figure S3 Source Data [file 44319_2026_786_MOESM19_ESM.zip › Appendix Figure S3 Source Data/S3A/CaMKIIalpha_3M.tif]

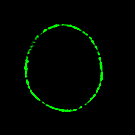

Supplement: Supplementary file 19 — Appendix Figure S3 Source Data [file 44319_2026_786_MOESM19_ESM.zip › Appendix Figure S3 Source Data/S3A/Sun1_3M.tif]

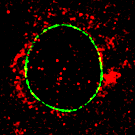

Supplement: Supplementary file 19 — Appendix Figure S3 Source Data [file 44319_2026_786_MOESM19_ESM.zip › Appendix Figure S3 Source Data/S3A/Merge_3M.tif]

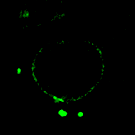

Supplement: Supplementary file 19 — Appendix Figure S3 Source Data [file 44319_2026_786_MOESM19_ESM.zip › Appendix Figure S3 Source Data/S3A/Sun1_20M.tif]

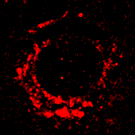

Supplement: Supplementary file 19 — Appendix Figure S3 Source Data [file 44319_2026_786_MOESM19_ESM.zip › Appendix Figure S3 Source Data/S3A/CaMKIIalpha_20M.tif]

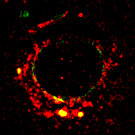

Supplement: Supplementary file 19 — Appendix Figure S3 Source Data [file 44319_2026_786_MOESM19_ESM.zip › Appendix Figure S3 Source Data/S3A/Merge_20M.tif]

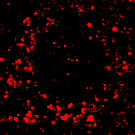

Supplement: Supplementary file 19 — Appendix Figure S3 Source Data [file 44319_2026_786_MOESM19_ESM.zip › Appendix Figure S3 Source Data/S3B/CaMKIIalpha_3M.tif]

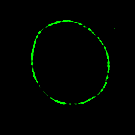

Supplement: Supplementary file 19 — Appendix Figure S3 Source Data [file 44319_2026_786_MOESM19_ESM.zip › Appendix Figure S3 Source Data/S3B/Sun2_3M.tif]

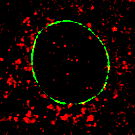

Supplement: Supplementary file 19 — Appendix Figure S3 Source Data [file 44319_2026_786_MOESM19_ESM.zip › Appendix Figure S3 Source Data/S3B/Merge_3M.tif]
