# Supplementary figures and images for: Age-related decline in nuclear envelope LINC complex drives neuronal aging via axon initial segment dysfunction (part 8 of 9)
Source: EMBO Rep. 2026 May 22;27(13):3788–825. doi: 10.1038/s44319-026-00786-5 (PMC13354796; doi:10.1038/s44319-026-00786-5)

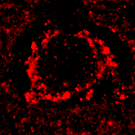

Supplement: Supplementary file 19 — Appendix Figure S3 Source Data [file 44319_2026_786_MOESM19_ESM.zip › Appendix Figure S3 Source Data/S3B/CaMKIIalpha_20M.tif]

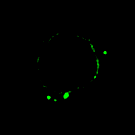

Supplement: Supplementary file 19 — Appendix Figure S3 Source Data [file 44319_2026_786_MOESM19_ESM.zip › Appendix Figure S3 Source Data/S3B/Sun2_20M.tif]

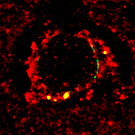

Supplement: Supplementary file 19 — Appendix Figure S3 Source Data [file 44319_2026_786_MOESM19_ESM.zip › Appendix Figure S3 Source Data/S3B/Merge_20M.tif]

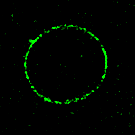

Supplement: Supplementary file 19 — Appendix Figure S3 Source Data [file 44319_2026_786_MOESM19_ESM.zip › Appendix Figure S3 Source Data/S3C/Nesprin-1_3M.tif]

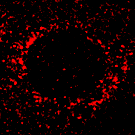

Supplement: Supplementary file 19 — Appendix Figure S3 Source Data [file 44319_2026_786_MOESM19_ESM.zip › Appendix Figure S3 Source Data/S3C/CaMKIIalpha_3M.tif]

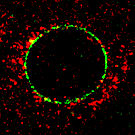

Supplement: Supplementary file 19 — Appendix Figure S3 Source Data [file 44319_2026_786_MOESM19_ESM.zip › Appendix Figure S3 Source Data/S3C/Merge_3M.tif]

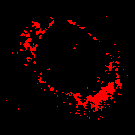

Supplement: Supplementary file 19 — Appendix Figure S3 Source Data [file 44319_2026_786_MOESM19_ESM.zip › Appendix Figure S3 Source Data/S3C/CaMKIIalpha_20M.tif]

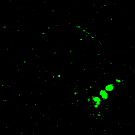

Supplement: Supplementary file 19 — Appendix Figure S3 Source Data [file 44319_2026_786_MOESM19_ESM.zip › Appendix Figure S3 Source Data/S3C/Nesprin-1_20M.tif]

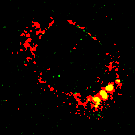

Supplement: Supplementary file 19 — Appendix Figure S3 Source Data [file 44319_2026_786_MOESM19_ESM.zip › Appendix Figure S3 Source Data/S3C/Merge_20M.tif]

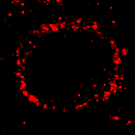

Supplement: Supplementary file 19 — Appendix Figure S3 Source Data [file 44319_2026_786_MOESM19_ESM.zip › Appendix Figure S3 Source Data/S3F/GAD65_3M.tif]

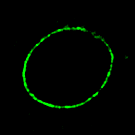

Supplement: Supplementary file 19 — Appendix Figure S3 Source Data [file 44319_2026_786_MOESM19_ESM.zip › Appendix Figure S3 Source Data/S3F/Sun2_3M.tif]

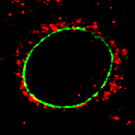

Supplement: Supplementary file 19 — Appendix Figure S3 Source Data [file 44319_2026_786_MOESM19_ESM.zip › Appendix Figure S3 Source Data/S3F/Merge_3M.tif]

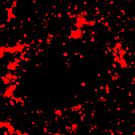

Supplement: Supplementary file 19 — Appendix Figure S3 Source Data [file 44319_2026_786_MOESM19_ESM.zip › Appendix Figure S3 Source Data/S3F/GAD65_20M.tif]

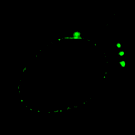

Supplement: Supplementary file 19 — Appendix Figure S3 Source Data [file 44319_2026_786_MOESM19_ESM.zip › Appendix Figure S3 Source Data/S3F/Sun2_20M.tif]

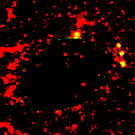

Supplement: Supplementary file 19 — Appendix Figure S3 Source Data [file 44319_2026_786_MOESM19_ESM.zip › Appendix Figure S3 Source Data/S3F/Merge_20M.tif]

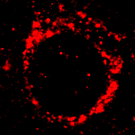

Supplement: Supplementary file 19 — Appendix Figure S3 Source Data [file 44319_2026_786_MOESM19_ESM.zip › Appendix Figure S3 Source Data/S3G/GAD65_3M.tif]

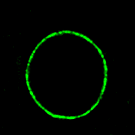

Supplement: Supplementary file 19 — Appendix Figure S3 Source Data [file 44319_2026_786_MOESM19_ESM.zip › Appendix Figure S3 Source Data/S3G/Nesprin-1_3M.tif]

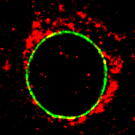

Supplement: Supplementary file 19 — Appendix Figure S3 Source Data [file 44319_2026_786_MOESM19_ESM.zip › Appendix Figure S3 Source Data/S3G/Merge_3M.tif]

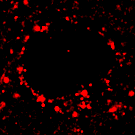

Supplement: Supplementary file 19 — Appendix Figure S3 Source Data [file 44319_2026_786_MOESM19_ESM.zip › Appendix Figure S3 Source Data/S3G/GAD65_20M.tif]

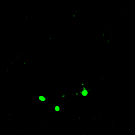

Supplement: Supplementary file 19 — Appendix Figure S3 Source Data [file 44319_2026_786_MOESM19_ESM.zip › Appendix Figure S3 Source Data/S3G/Nesprin-1_20M.tif]

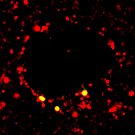

Supplement: Supplementary file 19 — Appendix Figure S3 Source Data [file 44319_2026_786_MOESM19_ESM.zip › Appendix Figure S3 Source Data/S3G/Merge_20M.tif]

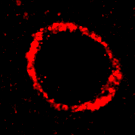

Supplement: Supplementary file 19 — Appendix Figure S3 Source Data [file 44319_2026_786_MOESM19_ESM.zip › Appendix Figure S3 Source Data/S3H/GAD65_3M.tif]

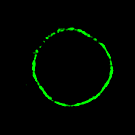

Supplement: Supplementary file 19 — Appendix Figure S3 Source Data [file 44319_2026_786_MOESM19_ESM.zip › Appendix Figure S3 Source Data/S3H/Nesprin-2_3M.tif]

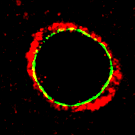

Supplement: Supplementary file 19 — Appendix Figure S3 Source Data [file 44319_2026_786_MOESM19_ESM.zip › Appendix Figure S3 Source Data/S3H/Merge_3M.tif]

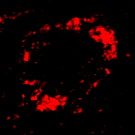

Supplement: Supplementary file 19 — Appendix Figure S3 Source Data [file 44319_2026_786_MOESM19_ESM.zip › Appendix Figure S3 Source Data/S3H/GAD65_20M.tif]

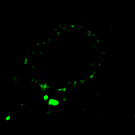

Supplement: Supplementary file 19 — Appendix Figure S3 Source Data [file 44319_2026_786_MOESM19_ESM.zip › Appendix Figure S3 Source Data/S3H/Nesprin-2_20M.tif]

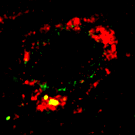

Supplement: Supplementary file 19 — Appendix Figure S3 Source Data [file 44319_2026_786_MOESM19_ESM.zip › Appendix Figure S3 Source Data/S3H/Merge_20M.tif]

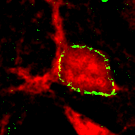

Supplement: Supplementary file 19 — Appendix Figure S3 Source Data [file 44319_2026_786_MOESM19_ESM.zip › Appendix Figure S3 Source Data/S3J/Merge_3M.tif]

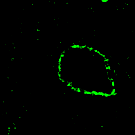

Supplement: Supplementary file 19 — Appendix Figure S3 Source Data [file 44319_2026_786_MOESM19_ESM.zip › Appendix Figure S3 Source Data/S3J/Sun2_3M.tif]

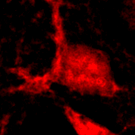

Supplement: Supplementary file 19 — Appendix Figure S3 Source Data [file 44319_2026_786_MOESM19_ESM.zip › Appendix Figure S3 Source Data/S3J/S100beta_3M.tif]

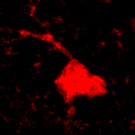

Supplement: Supplementary file 19 — Appendix Figure S3 Source Data [file 44319_2026_786_MOESM19_ESM.zip › Appendix Figure S3 Source Data/S3J/S100beta_20M.tif]

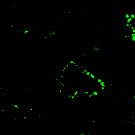

Supplement: Supplementary file 19 — Appendix Figure S3 Source Data [file 44319_2026_786_MOESM19_ESM.zip › Appendix Figure S3 Source Data/S3J/Sun2_20M.tif]

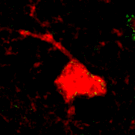

Supplement: Supplementary file 19 — Appendix Figure S3 Source Data [file 44319_2026_786_MOESM19_ESM.zip › Appendix Figure S3 Source Data/S3J/Merge_20M.tif]

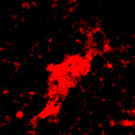

Supplement: Supplementary file 19 — Appendix Figure S3 Source Data [file 44319_2026_786_MOESM19_ESM.zip › Appendix Figure S3 Source Data/S3K/S100beta_3M.tif]

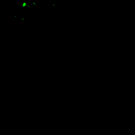

Supplement: Supplementary file 19 — Appendix Figure S3 Source Data [file 44319_2026_786_MOESM19_ESM.zip › Appendix Figure S3 Source Data/S3K/Nesprin-1_3M.tif]

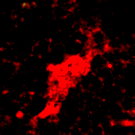

Supplement: Supplementary file 19 — Appendix Figure S3 Source Data [file 44319_2026_786_MOESM19_ESM.zip › Appendix Figure S3 Source Data/S3K/Merge_3M.tif]

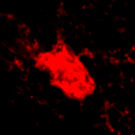

Supplement: Supplementary file 19 — Appendix Figure S3 Source Data [file 44319_2026_786_MOESM19_ESM.zip › Appendix Figure S3 Source Data/S3K/S100beta_20M.tif]

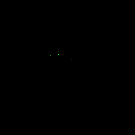

Supplement: Supplementary file 19 — Appendix Figure S3 Source Data [file 44319_2026_786_MOESM19_ESM.zip › Appendix Figure S3 Source Data/S3K/Nesprin-1_20M.tif]

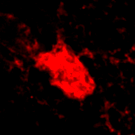

Supplement: Supplementary file 19 — Appendix Figure S3 Source Data [file 44319_2026_786_MOESM19_ESM.zip › Appendix Figure S3 Source Data/S3K/Merge_20M.tif]

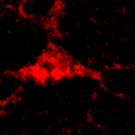

Supplement: Supplementary file 19 — Appendix Figure S3 Source Data [file 44319_2026_786_MOESM19_ESM.zip › Appendix Figure S3 Source Data/S3I/S100beta_3M.tif]

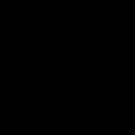

Supplement: Supplementary file 19 — Appendix Figure S3 Source Data [file 44319_2026_786_MOESM19_ESM.zip › Appendix Figure S3 Source Data/S3I/Sun1_3M.tif]

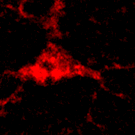

Supplement: Supplementary file 19 — Appendix Figure S3 Source Data [file 44319_2026_786_MOESM19_ESM.zip › Appendix Figure S3 Source Data/S3I/Merge_3M.tif]

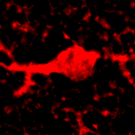

Supplement: Supplementary file 19 — Appendix Figure S3 Source Data [file 44319_2026_786_MOESM19_ESM.zip › Appendix Figure S3 Source Data/S3I/S100beta_20M.tif]

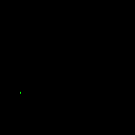

Supplement: Supplementary file 19 — Appendix Figure S3 Source Data [file 44319_2026_786_MOESM19_ESM.zip › Appendix Figure S3 Source Data/S3I/Sun1_20M.tif]

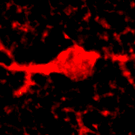

Supplement: Supplementary file 19 — Appendix Figure S3 Source Data [file 44319_2026_786_MOESM19_ESM.zip › Appendix Figure S3 Source Data/S3I/Merge_20M.tif]

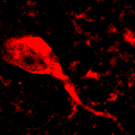

Supplement: Supplementary file 19 — Appendix Figure S3 Source Data [file 44319_2026_786_MOESM19_ESM.zip › Appendix Figure S3 Source Data/S3L/S100beta_3M.tif]

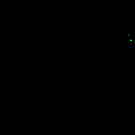

Supplement: Supplementary file 19 — Appendix Figure S3 Source Data [file 44319_2026_786_MOESM19_ESM.zip › Appendix Figure S3 Source Data/S3L/Nesprin-2_3M.tif]

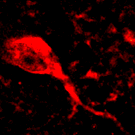

Supplement: Supplementary file 19 — Appendix Figure S3 Source Data [file 44319_2026_786_MOESM19_ESM.zip › Appendix Figure S3 Source Data/S3L/Merge_3M.tif]

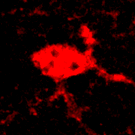

Supplement: Supplementary file 19 — Appendix Figure S3 Source Data [file 44319_2026_786_MOESM19_ESM.zip › Appendix Figure S3 Source Data/S3L/S100beta_20M.tif]

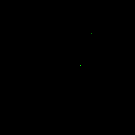

Supplement: Supplementary file 19 — Appendix Figure S3 Source Data [file 44319_2026_786_MOESM19_ESM.zip › Appendix Figure S3 Source Data/S3L/Nesprin-2_20M.tif]

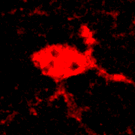

Supplement: Supplementary file 19 — Appendix Figure S3 Source Data [file 44319_2026_786_MOESM19_ESM.zip › Appendix Figure S3 Source Data/S3L/Merge_20M.tif]

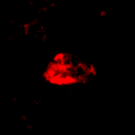

Supplement: Supplementary file 19 — Appendix Figure S3 Source Data [file 44319_2026_786_MOESM19_ESM.zip › Appendix Figure S3 Source Data/S3M/APC_3M.tif]

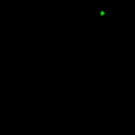

Supplement: Supplementary file 19 — Appendix Figure S3 Source Data [file 44319_2026_786_MOESM19_ESM.zip › Appendix Figure S3 Source Data/S3M/Sun1_3M.tif]

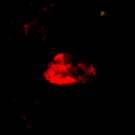

Supplement: Supplementary file 19 — Appendix Figure S3 Source Data [file 44319_2026_786_MOESM19_ESM.zip › Appendix Figure S3 Source Data/S3M/Merge_3M.tif]

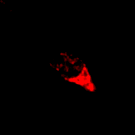

Supplement: Supplementary file 19 — Appendix Figure S3 Source Data [file 44319_2026_786_MOESM19_ESM.zip › Appendix Figure S3 Source Data/S3M/APC_20M.tif]

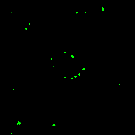

Supplement: Supplementary file 19 — Appendix Figure S3 Source Data [file 44319_2026_786_MOESM19_ESM.zip › Appendix Figure S3 Source Data/S3M/Sun1_20M.tif]

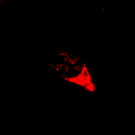

Supplement: Supplementary file 19 — Appendix Figure S3 Source Data [file 44319_2026_786_MOESM19_ESM.zip › Appendix Figure S3 Source Data/S3M/Merge_20M.tif]

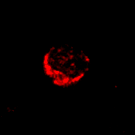

Supplement: Supplementary file 19 — Appendix Figure S3 Source Data [file 44319_2026_786_MOESM19_ESM.zip › Appendix Figure S3 Source Data/S3N/APC_3M.tif]

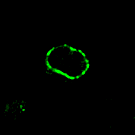

Supplement: Supplementary file 19 — Appendix Figure S3 Source Data [file 44319_2026_786_MOESM19_ESM.zip › Appendix Figure S3 Source Data/S3N/Sun2_3M.tif]

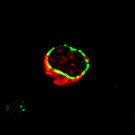

Supplement: Supplementary file 19 — Appendix Figure S3 Source Data [file 44319_2026_786_MOESM19_ESM.zip › Appendix Figure S3 Source Data/S3N/Merge_3M.tif]

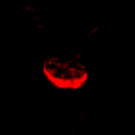

Supplement: Supplementary file 19 — Appendix Figure S3 Source Data [file 44319_2026_786_MOESM19_ESM.zip › Appendix Figure S3 Source Data/S3N/APC_20M.tif]

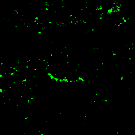

Supplement: Supplementary file 19 — Appendix Figure S3 Source Data [file 44319_2026_786_MOESM19_ESM.zip › Appendix Figure S3 Source Data/S3N/Sun2_20M.tif]

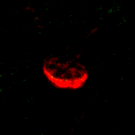

Supplement: Supplementary file 19 — Appendix Figure S3 Source Data [file 44319_2026_786_MOESM19_ESM.zip › Appendix Figure S3 Source Data/S3N/Merge_20M.tif]

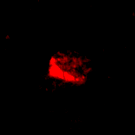

Supplement: Supplementary file 19 — Appendix Figure S3 Source Data [file 44319_2026_786_MOESM19_ESM.zip › Appendix Figure S3 Source Data/S3O/APC_3M.tif]

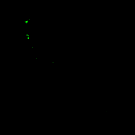

Supplement: Supplementary file 19 — Appendix Figure S3 Source Data [file 44319_2026_786_MOESM19_ESM.zip › Appendix Figure S3 Source Data/S3O/Nesprin-1_3M.tif]

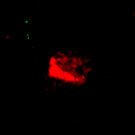

Supplement: Supplementary file 19 — Appendix Figure S3 Source Data [file 44319_2026_786_MOESM19_ESM.zip › Appendix Figure S3 Source Data/S3O/Merge_3M.tif]

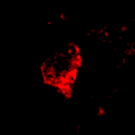

Supplement: Supplementary file 19 — Appendix Figure S3 Source Data [file 44319_2026_786_MOESM19_ESM.zip › Appendix Figure S3 Source Data/S3O/APC_20M.tif]

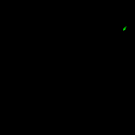

Supplement: Supplementary file 19 — Appendix Figure S3 Source Data [file 44319_2026_786_MOESM19_ESM.zip › Appendix Figure S3 Source Data/S3O/Nesprin-1_20M.tif]

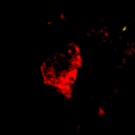

Supplement: Supplementary file 19 — Appendix Figure S3 Source Data [file 44319_2026_786_MOESM19_ESM.zip › Appendix Figure S3 Source Data/S3O/Merge_20M.tif]

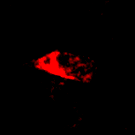

Supplement: Supplementary file 19 — Appendix Figure S3 Source Data [file 44319_2026_786_MOESM19_ESM.zip › Appendix Figure S3 Source Data/S3P/APC_3M.tif]

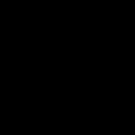

Supplement: Supplementary file 19 — Appendix Figure S3 Source Data [file 44319_2026_786_MOESM19_ESM.zip › Appendix Figure S3 Source Data/S3P/Nesprin-2_3M.tif]

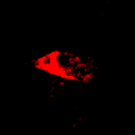

Supplement: Supplementary file 19 — Appendix Figure S3 Source Data [file 44319_2026_786_MOESM19_ESM.zip › Appendix Figure S3 Source Data/S3P/Merge_3M.tif]

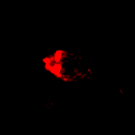

Supplement: Supplementary file 19 — Appendix Figure S3 Source Data [file 44319_2026_786_MOESM19_ESM.zip › Appendix Figure S3 Source Data/S3P/APC_20M.tif]

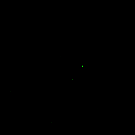

Supplement: Supplementary file 19 — Appendix Figure S3 Source Data [file 44319_2026_786_MOESM19_ESM.zip › Appendix Figure S3 Source Data/S3P/Nesprin-2_20M.tif]

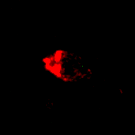

Supplement: Supplementary file 19 — Appendix Figure S3 Source Data [file 44319_2026_786_MOESM19_ESM.zip › Appendix Figure S3 Source Data/S3P/Merge_20M.tif]

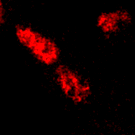

Supplement: Supplementary file 19 — Appendix Figure S3 Source Data [file 44319_2026_786_MOESM19_ESM.zip › Appendix Figure S3 Source Data/S3R/Sox2_3M.tif]

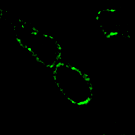

Supplement: Supplementary file 19 — Appendix Figure S3 Source Data [file 44319_2026_786_MOESM19_ESM.zip › Appendix Figure S3 Source Data/S3R/Sun2_3M.tif]

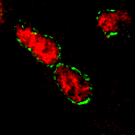

Supplement: Supplementary file 19 — Appendix Figure S3 Source Data [file 44319_2026_786_MOESM19_ESM.zip › Appendix Figure S3 Source Data/S3R/Merge_3M.tif]

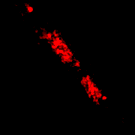

Supplement: Supplementary file 19 — Appendix Figure S3 Source Data [file 44319_2026_786_MOESM19_ESM.zip › Appendix Figure S3 Source Data/S3R/Sox2_20M.tif]

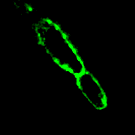

Supplement: Supplementary file 19 — Appendix Figure S3 Source Data [file 44319_2026_786_MOESM19_ESM.zip › Appendix Figure S3 Source Data/S3R/Sun2_20M.tif]

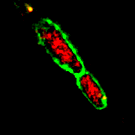

Supplement: Supplementary file 19 — Appendix Figure S3 Source Data [file 44319_2026_786_MOESM19_ESM.zip › Appendix Figure S3 Source Data/S3R/Merge_20M.tif]

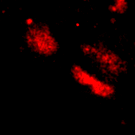

Supplement: Supplementary file 19 — Appendix Figure S3 Source Data [file 44319_2026_786_MOESM19_ESM.zip › Appendix Figure S3 Source Data/S3S/Sox2_3M.tif]

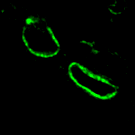

Supplement: Supplementary file 19 — Appendix Figure S3 Source Data [file 44319_2026_786_MOESM19_ESM.zip › Appendix Figure S3 Source Data/S3S/Nesprin-1_3M.tif]

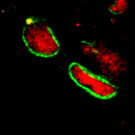

Supplement: Supplementary file 19 — Appendix Figure S3 Source Data [file 44319_2026_786_MOESM19_ESM.zip › Appendix Figure S3 Source Data/S3S/Merge_3M.tif]

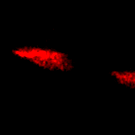

Supplement: Supplementary file 19 — Appendix Figure S3 Source Data [file 44319_2026_786_MOESM19_ESM.zip › Appendix Figure S3 Source Data/S3S/Sox2_20M.tif]

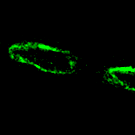

Supplement: Supplementary file 19 — Appendix Figure S3 Source Data [file 44319_2026_786_MOESM19_ESM.zip › Appendix Figure S3 Source Data/S3S/Nesprin-1_20M.tif]

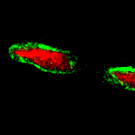

Supplement: Supplementary file 19 — Appendix Figure S3 Source Data [file 44319_2026_786_MOESM19_ESM.zip › Appendix Figure S3 Source Data/S3S/Merge_20M.tif]

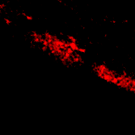

Supplement: Supplementary file 19 — Appendix Figure S3 Source Data [file 44319_2026_786_MOESM19_ESM.zip › Appendix Figure S3 Source Data/S3Q/Sox2_3M.tif]

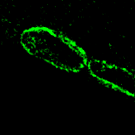

Supplement: Supplementary file 19 — Appendix Figure S3 Source Data [file 44319_2026_786_MOESM19_ESM.zip › Appendix Figure S3 Source Data/S3Q/Sun1_3M.tif]

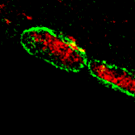

Supplement: Supplementary file 19 — Appendix Figure S3 Source Data [file 44319_2026_786_MOESM19_ESM.zip › Appendix Figure S3 Source Data/S3Q/Merge_3M.tif]

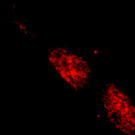

Supplement: Supplementary file 19 — Appendix Figure S3 Source Data [file 44319_2026_786_MOESM19_ESM.zip › Appendix Figure S3 Source Data/S3Q/Sox2_20M.tif]

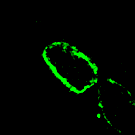

Supplement: Supplementary file 19 — Appendix Figure S3 Source Data [file 44319_2026_786_MOESM19_ESM.zip › Appendix Figure S3 Source Data/S3Q/Sun1_20M.tif]

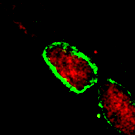

Supplement: Supplementary file 19 — Appendix Figure S3 Source Data [file 44319_2026_786_MOESM19_ESM.zip › Appendix Figure S3 Source Data/S3Q/Merge_20M.tif]

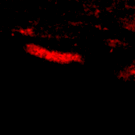

Supplement: Supplementary file 19 — Appendix Figure S3 Source Data [file 44319_2026_786_MOESM19_ESM.zip › Appendix Figure S3 Source Data/S3T/Sox2_3M.tif]

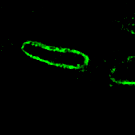

Supplement: Supplementary file 19 — Appendix Figure S3 Source Data [file 44319_2026_786_MOESM19_ESM.zip › Appendix Figure S3 Source Data/S3T/Nesprin-2_3M.tif]

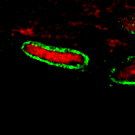

Supplement: Supplementary file 19 — Appendix Figure S3 Source Data [file 44319_2026_786_MOESM19_ESM.zip › Appendix Figure S3 Source Data/S3T/Merge_3M.tif]

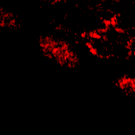

Supplement: Supplementary file 19 — Appendix Figure S3 Source Data [file 44319_2026_786_MOESM19_ESM.zip › Appendix Figure S3 Source Data/S3T/Sox2_20M.tif]

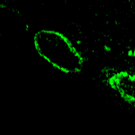

Supplement: Supplementary file 19 — Appendix Figure S3 Source Data [file 44319_2026_786_MOESM19_ESM.zip › Appendix Figure S3 Source Data/S3T/Nesprin-2_20M.tif]

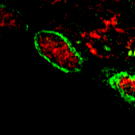

Supplement: Supplementary file 19 — Appendix Figure S3 Source Data [file 44319_2026_786_MOESM19_ESM.zip › Appendix Figure S3 Source Data/S3T/Merge_20M.tif]

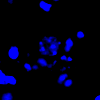

Supplement: Supplementary file 20 — Appendix Figure S4 Source Data [file 44319_2026_786_MOESM20_ESM.zip › Appendix Figure S4 Source Data/S4E/Hoechst_Control.tif]
