# Supplementary figures and images for: Age-related decline in nuclear envelope LINC complex drives neuronal aging via axon initial segment dysfunction (part 9 of 9)
Source: EMBO Rep. 2026 May 22;27(13):3788–825. doi: 10.1038/s44319-026-00786-5 (PMC13354796; doi:10.1038/s44319-026-00786-5)

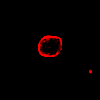

Supplement: Supplementary file 20 — Appendix Figure S4 Source Data [file 44319_2026_786_MOESM20_ESM.zip › Appendix Figure S4 Source Data/S4E/Lamin B1_Control.tif]

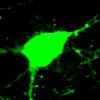

Supplement: Supplementary file 20 — Appendix Figure S4 Source Data [file 44319_2026_786_MOESM20_ESM.zip › Appendix Figure S4 Source Data/S4E/Venus_Control.tif]

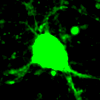

Supplement: Supplementary file 20 — Appendix Figure S4 Source Data [file 44319_2026_786_MOESM20_ESM.zip › Appendix Figure S4 Source Data/S4E/Venus_LINC-DN.tif]

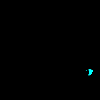

Supplement: Supplementary file 20 — Appendix Figure S4 Source Data [file 44319_2026_786_MOESM20_ESM.zip › Appendix Figure S4 Source Data/S4E/HA_Control.tif]

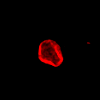

Supplement: Supplementary file 20 — Appendix Figure S4 Source Data [file 44319_2026_786_MOESM20_ESM.zip › Appendix Figure S4 Source Data/S4E/Lamin B1_LINC-DN.tif]

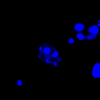

Supplement: Supplementary file 20 — Appendix Figure S4 Source Data [file 44319_2026_786_MOESM20_ESM.zip › Appendix Figure S4 Source Data/S4E/Hoechst_LINC-DN.tif]

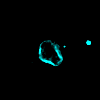

Supplement: Supplementary file 20 — Appendix Figure S4 Source Data [file 44319_2026_786_MOESM20_ESM.zip › Appendix Figure S4 Source Data/S4E/HA_LINC-DN.tif]

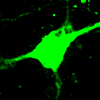

Supplement: Supplementary file 20 — Appendix Figure S4 Source Data [file 44319_2026_786_MOESM20_ESM.zip › Appendix Figure S4 Source Data/S4A/Venus_Control.tif]

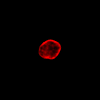

Supplement: Supplementary file 20 — Appendix Figure S4 Source Data [file 44319_2026_786_MOESM20_ESM.zip › Appendix Figure S4 Source Data/S4A/Nesprin-1_Control.tif]

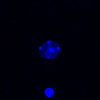

Supplement: Supplementary file 20 — Appendix Figure S4 Source Data [file 44319_2026_786_MOESM20_ESM.zip › Appendix Figure S4 Source Data/S4A/Hoechst_Control.tif]

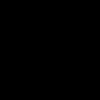

Supplement: Supplementary file 20 — Appendix Figure S4 Source Data [file 44319_2026_786_MOESM20_ESM.zip › Appendix Figure S4 Source Data/S4A/HA_Control.tif]

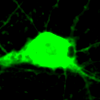

Supplement: Supplementary file 20 — Appendix Figure S4 Source Data [file 44319_2026_786_MOESM20_ESM.zip › Appendix Figure S4 Source Data/S4A/Venus_LINC-DN.tif]

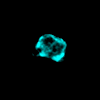

Supplement: Supplementary file 20 — Appendix Figure S4 Source Data [file 44319_2026_786_MOESM20_ESM.zip › Appendix Figure S4 Source Data/S4A/HA_LINC-DN.tif]

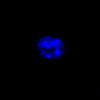

Supplement: Supplementary file 20 — Appendix Figure S4 Source Data [file 44319_2026_786_MOESM20_ESM.zip › Appendix Figure S4 Source Data/S4A/Hoechst_LINC-DN.tif]

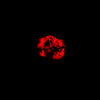

Supplement: Supplementary file 20 — Appendix Figure S4 Source Data [file 44319_2026_786_MOESM20_ESM.zip › Appendix Figure S4 Source Data/S4A/Nesprin-1_LINC-DN.tif]

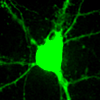

Supplement: Supplementary file 20 — Appendix Figure S4 Source Data [file 44319_2026_786_MOESM20_ESM.zip › Appendix Figure S4 Source Data/S4B/Venus_Control.tif]

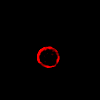

Supplement: Supplementary file 20 — Appendix Figure S4 Source Data [file 44319_2026_786_MOESM20_ESM.zip › Appendix Figure S4 Source Data/S4B/Nesprin-2_Control.tif]

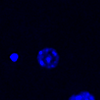

Supplement: Supplementary file 20 — Appendix Figure S4 Source Data [file 44319_2026_786_MOESM20_ESM.zip › Appendix Figure S4 Source Data/S4B/Hoechst_Control.tif]

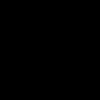

Supplement: Supplementary file 20 — Appendix Figure S4 Source Data [file 44319_2026_786_MOESM20_ESM.zip › Appendix Figure S4 Source Data/S4B/HA_Control.tif]

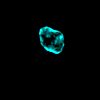

Supplement: Supplementary file 20 — Appendix Figure S4 Source Data [file 44319_2026_786_MOESM20_ESM.zip › Appendix Figure S4 Source Data/S4B/HA_LINC-DN.tif]

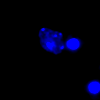

Supplement: Supplementary file 20 — Appendix Figure S4 Source Data [file 44319_2026_786_MOESM20_ESM.zip › Appendix Figure S4 Source Data/S4B/Hoechst_LINC-DN.tif]

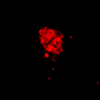

Supplement: Supplementary file 20 — Appendix Figure S4 Source Data [file 44319_2026_786_MOESM20_ESM.zip › Appendix Figure S4 Source Data/S4B/Nesprin-2_LINC-DN.tif]

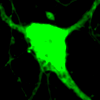

Supplement: Supplementary file 20 — Appendix Figure S4 Source Data [file 44319_2026_786_MOESM20_ESM.zip › Appendix Figure S4 Source Data/S4B/Venus_LINC-DN.tif]

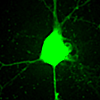

Supplement: Supplementary file 20 — Appendix Figure S4 Source Data [file 44319_2026_786_MOESM20_ESM.zip › Appendix Figure S4 Source Data/S4C/Venus_Control.tif]

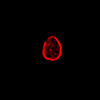

Supplement: Supplementary file 20 — Appendix Figure S4 Source Data [file 44319_2026_786_MOESM20_ESM.zip › Appendix Figure S4 Source Data/S4C/Sun1_Control.tif]

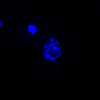

Supplement: Supplementary file 20 — Appendix Figure S4 Source Data [file 44319_2026_786_MOESM20_ESM.zip › Appendix Figure S4 Source Data/S4C/Hoechst_Control.tif]

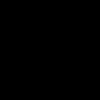

Supplement: Supplementary file 20 — Appendix Figure S4 Source Data [file 44319_2026_786_MOESM20_ESM.zip › Appendix Figure S4 Source Data/S4C/HA_Control.tif]

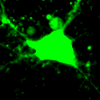

Supplement: Supplementary file 20 — Appendix Figure S4 Source Data [file 44319_2026_786_MOESM20_ESM.zip › Appendix Figure S4 Source Data/S4C/Venus_LINC-DN.tif]

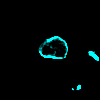

Supplement: Supplementary file 20 — Appendix Figure S4 Source Data [file 44319_2026_786_MOESM20_ESM.zip › Appendix Figure S4 Source Data/S4C/HA_LINC-DN.tif]

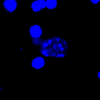

Supplement: Supplementary file 20 — Appendix Figure S4 Source Data [file 44319_2026_786_MOESM20_ESM.zip › Appendix Figure S4 Source Data/S4C/Hoechst_LINC-DN.tif]

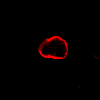

Supplement: Supplementary file 20 — Appendix Figure S4 Source Data [file 44319_2026_786_MOESM20_ESM.zip › Appendix Figure S4 Source Data/S4C/Sun1_LINC-DN.tif]

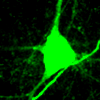

Supplement: Supplementary file 20 — Appendix Figure S4 Source Data [file 44319_2026_786_MOESM20_ESM.zip › Appendix Figure S4 Source Data/S4D/Venus_Control.tif]

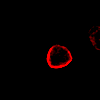

Supplement: Supplementary file 20 — Appendix Figure S4 Source Data [file 44319_2026_786_MOESM20_ESM.zip › Appendix Figure S4 Source Data/S4D/Sun2_Control.tif]

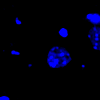

Supplement: Supplementary file 20 — Appendix Figure S4 Source Data [file 44319_2026_786_MOESM20_ESM.zip › Appendix Figure S4 Source Data/S4D/Hoechst_Control.tif]

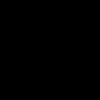

Supplement: Supplementary file 20 — Appendix Figure S4 Source Data [file 44319_2026_786_MOESM20_ESM.zip › Appendix Figure S4 Source Data/S4D/HA_Control.tif]

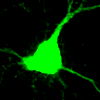

Supplement: Supplementary file 20 — Appendix Figure S4 Source Data [file 44319_2026_786_MOESM20_ESM.zip › Appendix Figure S4 Source Data/S4D/Venus_LINC-DN.tif]

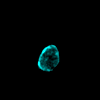

Supplement: Supplementary file 20 — Appendix Figure S4 Source Data [file 44319_2026_786_MOESM20_ESM.zip › Appendix Figure S4 Source Data/S4D/HA_LINC-DN.tif]

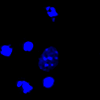

Supplement: Supplementary file 20 — Appendix Figure S4 Source Data [file 44319_2026_786_MOESM20_ESM.zip › Appendix Figure S4 Source Data/S4D/Hoechst_LINC-DN.tif]

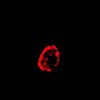

Supplement: Supplementary file 20 — Appendix Figure S4 Source Data [file 44319_2026_786_MOESM20_ESM.zip › Appendix Figure S4 Source Data/S4D/Sun2_LINC-DN.tif]

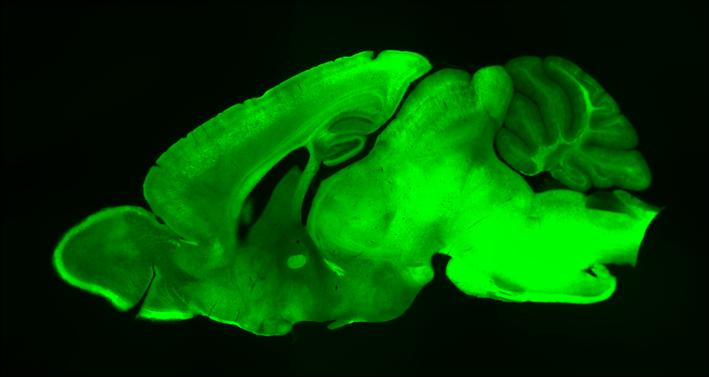

Supplement: Supplementary file 21 — Appendix Figure S5 Source Data [file 44319_2026_786_MOESM21_ESM.zip › Appendix Figure S5 Source Data/S5B/2M_whole brain.tif]

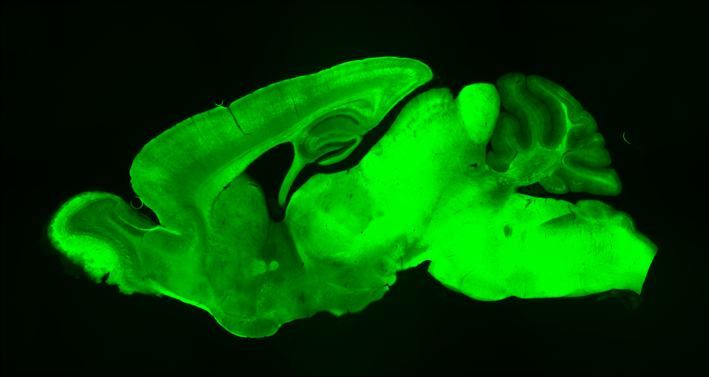

Supplement: Supplementary file 21 — Appendix Figure S5 Source Data [file 44319_2026_786_MOESM21_ESM.zip › Appendix Figure S5 Source Data/S5B/4M_whole brain.tif]

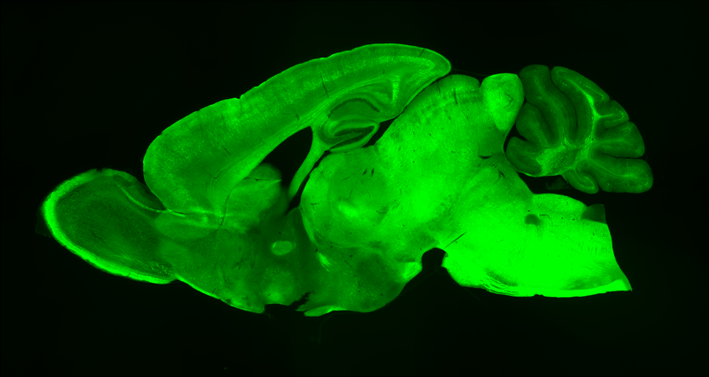

Supplement: Supplementary file 21 — Appendix Figure S5 Source Data [file 44319_2026_786_MOESM21_ESM.zip › Appendix Figure S5 Source Data/S5B/6M_whole brain.tif]

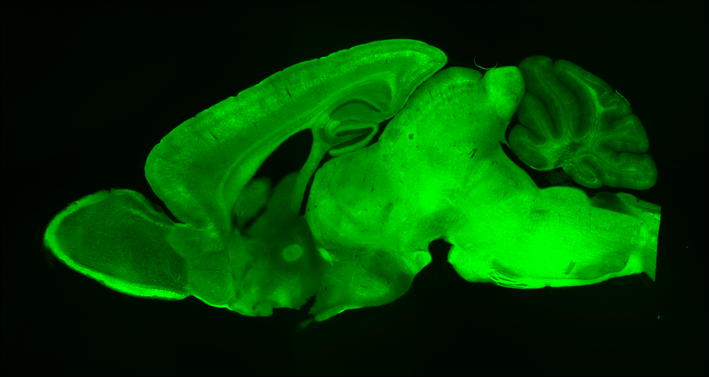

Supplement: Supplementary file 21 — Appendix Figure S5 Source Data [file 44319_2026_786_MOESM21_ESM.zip › Appendix Figure S5 Source Data/S5B/8M_whole brain.tif]

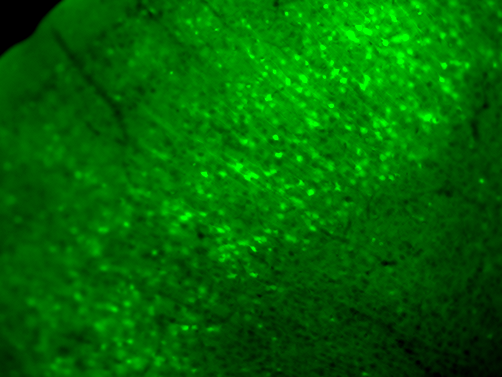

Supplement: Supplementary file 21 — Appendix Figure S5 Source Data [file 44319_2026_786_MOESM21_ESM.zip › Appendix Figure S5 Source Data/S5B/2M_PFC.tif]

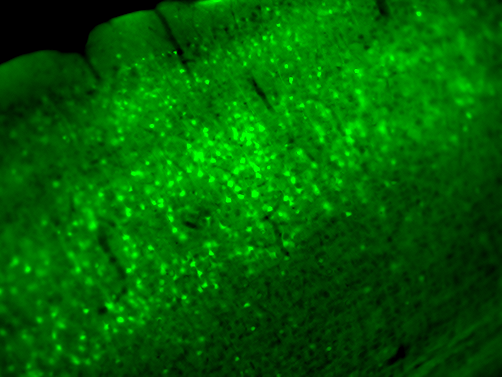

Supplement: Supplementary file 21 — Appendix Figure S5 Source Data [file 44319_2026_786_MOESM21_ESM.zip › Appendix Figure S5 Source Data/S5B/2M_MC.tif]

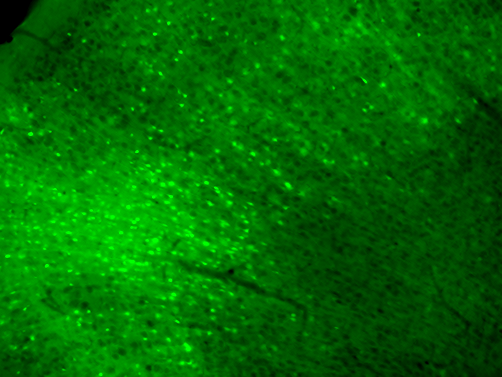

Supplement: Supplementary file 21 — Appendix Figure S5 Source Data [file 44319_2026_786_MOESM21_ESM.zip › Appendix Figure S5 Source Data/S5B/4M_PFC.tif]

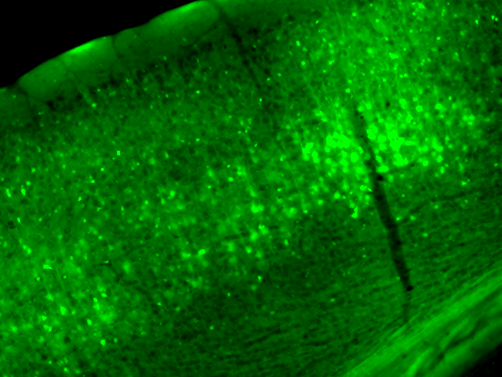

Supplement: Supplementary file 21 — Appendix Figure S5 Source Data [file 44319_2026_786_MOESM21_ESM.zip › Appendix Figure S5 Source Data/S5B/4M_MC.tif]

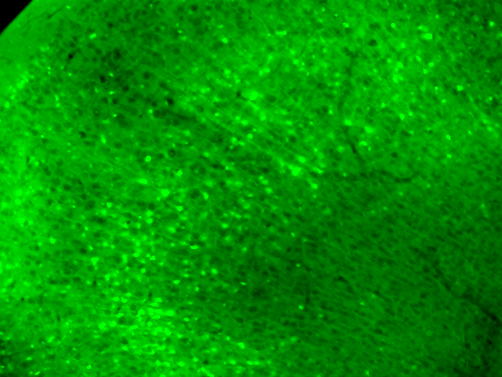

Supplement: Supplementary file 21 — Appendix Figure S5 Source Data [file 44319_2026_786_MOESM21_ESM.zip › Appendix Figure S5 Source Data/S5B/6M_PFC.tif]

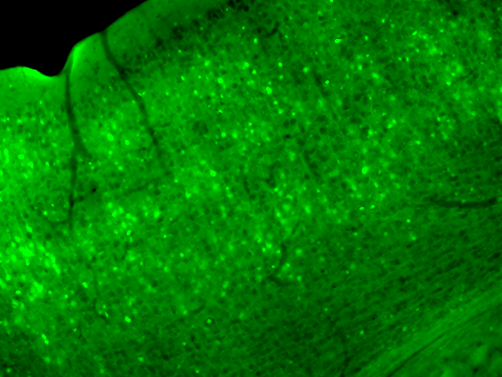

Supplement: Supplementary file 21 — Appendix Figure S5 Source Data [file 44319_2026_786_MOESM21_ESM.zip › Appendix Figure S5 Source Data/S5B/6M_MC.tif]

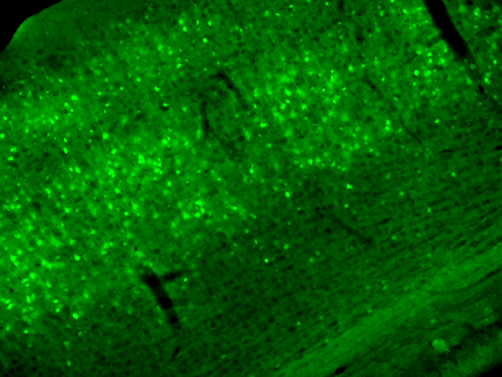

Supplement: Supplementary file 21 — Appendix Figure S5 Source Data [file 44319_2026_786_MOESM21_ESM.zip › Appendix Figure S5 Source Data/S5B/8M_MC.tif]

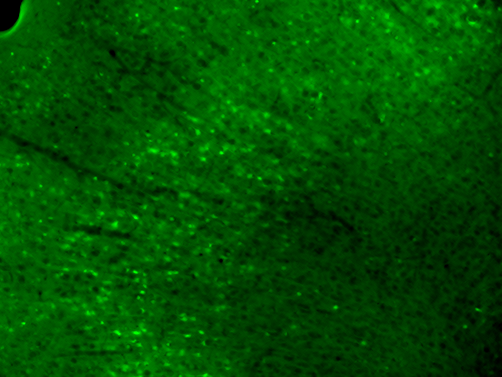

Supplement: Supplementary file 21 — Appendix Figure S5 Source Data [file 44319_2026_786_MOESM21_ESM.zip › Appendix Figure S5 Source Data/S5B/8M_PFC.tif]

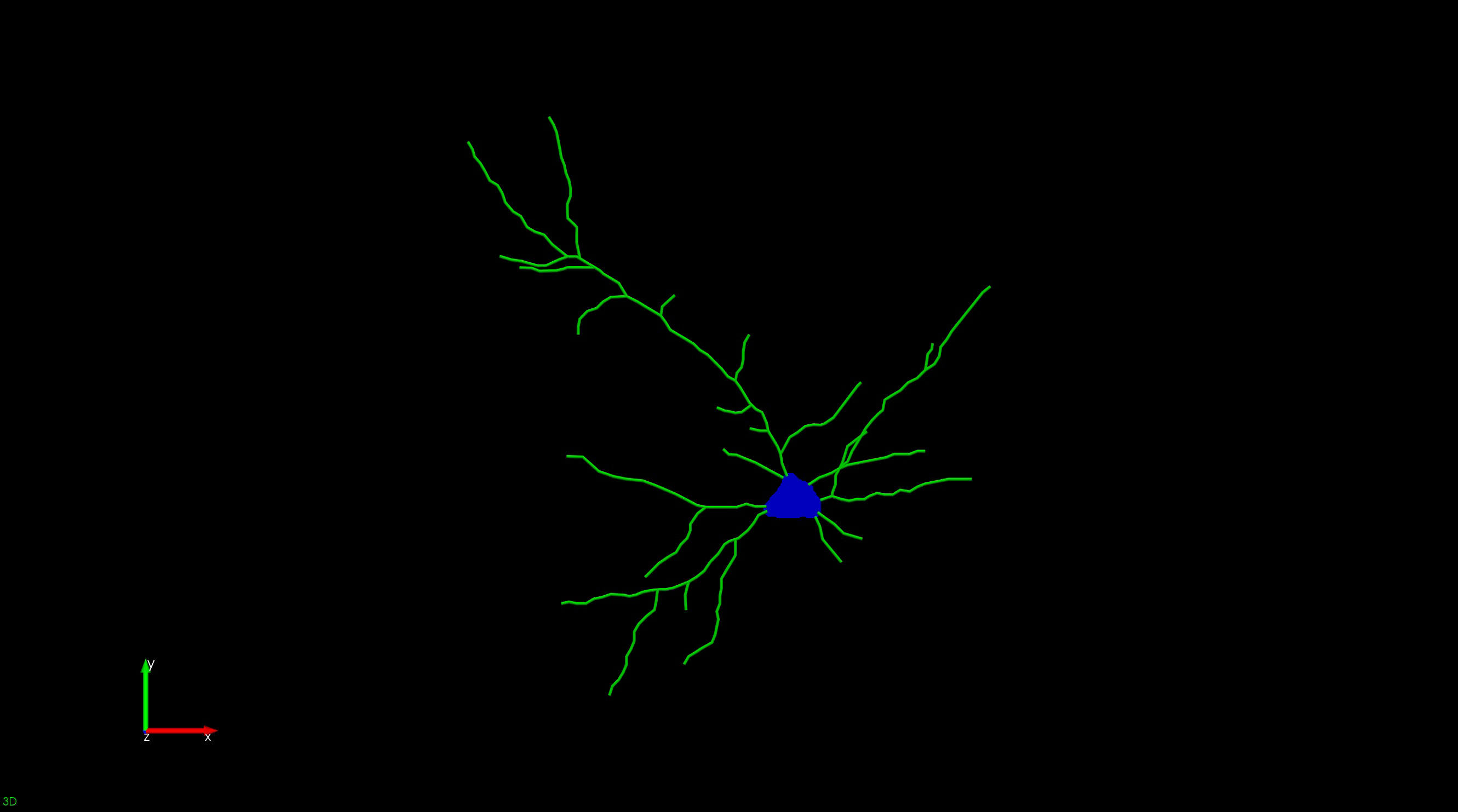

Supplement: Supplementary file 22 — Appendix Figure S6 Source Data [file 44319_2026_786_MOESM22_ESM.zip › Appendix Figure S6 Source Data/S6A/LINC-DN.tif]

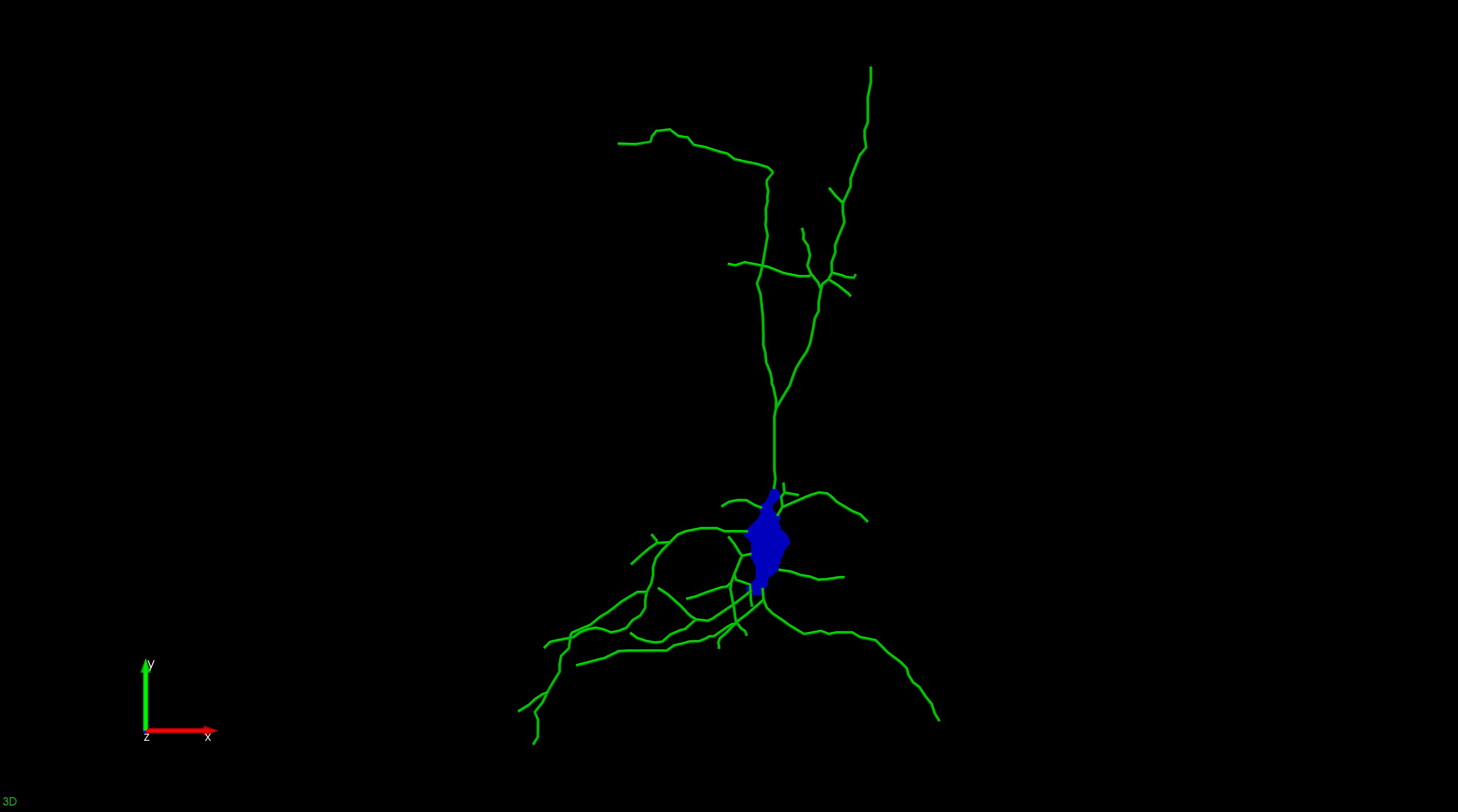

Supplement: Supplementary file 22 — Appendix Figure S6 Source Data [file 44319_2026_786_MOESM22_ESM.zip › Appendix Figure S6 Source Data/S6A/Control.tif]

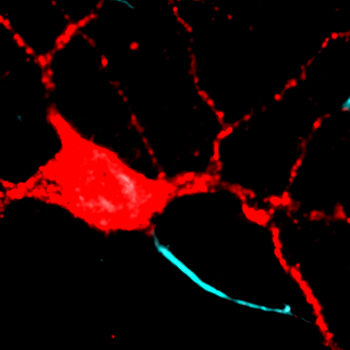

Supplement: Supplementary file 22 — Appendix Figure S6 Source Data [file 44319_2026_786_MOESM22_ESM.zip › Appendix Figure S6 Source Data/S6F/Merge_Control.tif]

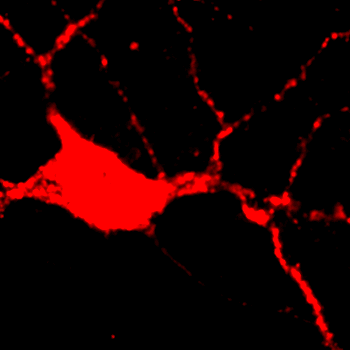

Supplement: Supplementary file 22 — Appendix Figure S6 Source Data [file 44319_2026_786_MOESM22_ESM.zip › Appendix Figure S6 Source Data/S6F/TfR_Control.tif]

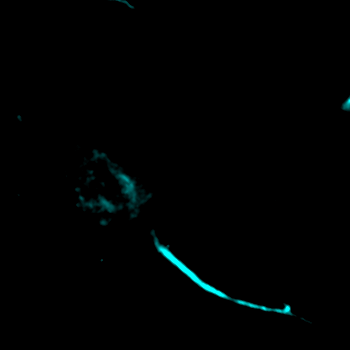

Supplement: Supplementary file 22 — Appendix Figure S6 Source Data [file 44319_2026_786_MOESM22_ESM.zip › Appendix Figure S6 Source Data/S6F/Ankyrin-G_Control.tif]

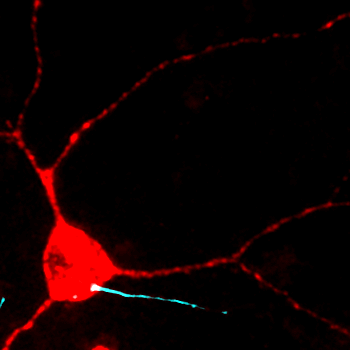

Supplement: Supplementary file 22 — Appendix Figure S6 Source Data [file 44319_2026_786_MOESM22_ESM.zip › Appendix Figure S6 Source Data/S6F/Merge_LINC-DN.tif]

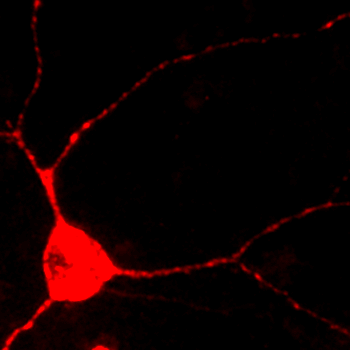

Supplement: Supplementary file 22 — Appendix Figure S6 Source Data [file 44319_2026_786_MOESM22_ESM.zip › Appendix Figure S6 Source Data/S6F/TfR_LINC-DN.tif]

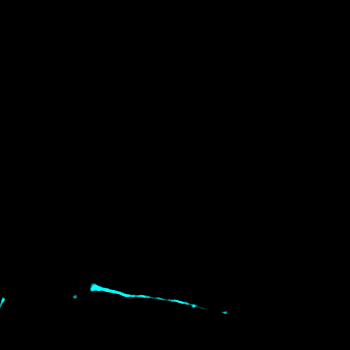

Supplement: Supplementary file 22 — Appendix Figure S6 Source Data [file 44319_2026_786_MOESM22_ESM.zip › Appendix Figure S6 Source Data/S6F/Ankyrin-G_LINC-DN.tif]

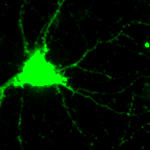

Supplement: Supplementary file 23 — Appendix Figure S7 Source Data [file 44319_2026_786_MOESM23_ESM.zip › Appendix Figure S7 Source Data/S7G/Venus_LINC-DN.tif]

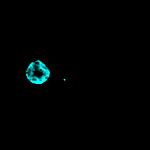

Supplement: Supplementary file 23 — Appendix Figure S7 Source Data [file 44319_2026_786_MOESM23_ESM.zip › Appendix Figure S7 Source Data/S7G/HA_LINC-DN.tif]

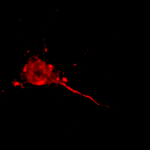

Supplement: Supplementary file 23 — Appendix Figure S7 Source Data [file 44319_2026_786_MOESM23_ESM.zip › Appendix Figure S7 Source Data/S7G/pMLC_LINC-DN.tif]

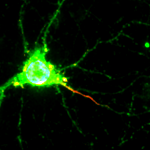

Supplement: Supplementary file 23 — Appendix Figure S7 Source Data [file 44319_2026_786_MOESM23_ESM.zip › Appendix Figure S7 Source Data/S7G/Merge_LINC-DN.tif]

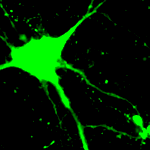

Supplement: Supplementary file 23 — Appendix Figure S7 Source Data [file 44319_2026_786_MOESM23_ESM.zip › Appendix Figure S7 Source Data/S7G/Venus_Control.tif]

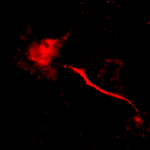

Supplement: Supplementary file 23 — Appendix Figure S7 Source Data [file 44319_2026_786_MOESM23_ESM.zip › Appendix Figure S7 Source Data/S7G/pMLC_Control.tif]

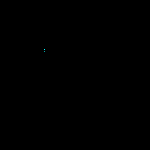

Supplement: Supplementary file 23 — Appendix Figure S7 Source Data [file 44319_2026_786_MOESM23_ESM.zip › Appendix Figure S7 Source Data/S7G/HA_Control.tif]

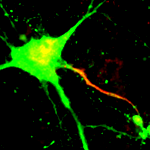

Supplement: Supplementary file 23 — Appendix Figure S7 Source Data [file 44319_2026_786_MOESM23_ESM.zip › Appendix Figure S7 Source Data/S7G/Merge_Control.tif]

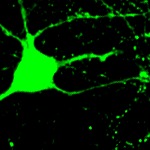

Supplement: Supplementary file 23 — Appendix Figure S7 Source Data [file 44319_2026_786_MOESM23_ESM.zip › Appendix Figure S7 Source Data/S7A/Venus_Control.tif]

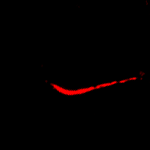

Supplement: Supplementary file 23 — Appendix Figure S7 Source Data [file 44319_2026_786_MOESM23_ESM.zip › Appendix Figure S7 Source Data/S7A/betaIV-spectrin_Control.tif]

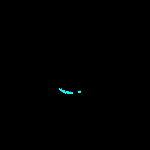

Supplement: Supplementary file 23 — Appendix Figure S7 Source Data [file 44319_2026_786_MOESM23_ESM.zip › Appendix Figure S7 Source Data/S7A/HA_Control.tif]

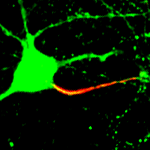

Supplement: Supplementary file 23 — Appendix Figure S7 Source Data [file 44319_2026_786_MOESM23_ESM.zip › Appendix Figure S7 Source Data/S7A/Merge_Control.tif]

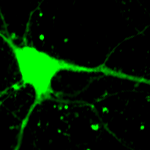

Supplement: Supplementary file 23 — Appendix Figure S7 Source Data [file 44319_2026_786_MOESM23_ESM.zip › Appendix Figure S7 Source Data/S7A/Venus_LINC-DN.tif]

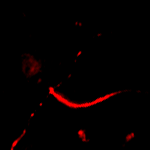

Supplement: Supplementary file 23 — Appendix Figure S7 Source Data [file 44319_2026_786_MOESM23_ESM.zip › Appendix Figure S7 Source Data/S7A/betaIV-spectrin_LINC-DN.tif]

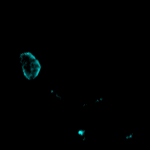

Supplement: Supplementary file 23 — Appendix Figure S7 Source Data [file 44319_2026_786_MOESM23_ESM.zip › Appendix Figure S7 Source Data/S7A/HA_LINC-DN.tif]

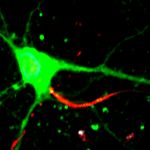

Supplement: Supplementary file 23 — Appendix Figure S7 Source Data [file 44319_2026_786_MOESM23_ESM.zip › Appendix Figure S7 Source Data/S7A/Merge_LINC-DN.tif]

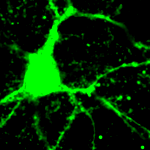

Supplement: Supplementary file 23 — Appendix Figure S7 Source Data [file 44319_2026_786_MOESM23_ESM.zip › Appendix Figure S7 Source Data/S7D/Venus_Control.tif]

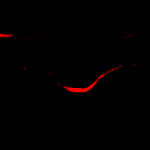

Supplement: Supplementary file 23 — Appendix Figure S7 Source Data [file 44319_2026_786_MOESM23_ESM.zip › Appendix Figure S7 Source Data/S7D/Trim46_Control.tif]

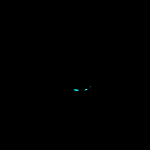

Supplement: Supplementary file 23 — Appendix Figure S7 Source Data [file 44319_2026_786_MOESM23_ESM.zip › Appendix Figure S7 Source Data/S7D/HA_Control.tif]

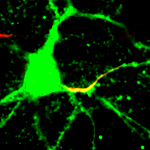

Supplement: Supplementary file 23 — Appendix Figure S7 Source Data [file 44319_2026_786_MOESM23_ESM.zip › Appendix Figure S7 Source Data/S7D/Merge_Control.tif]

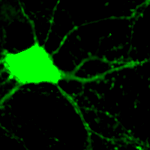

Supplement: Supplementary file 23 — Appendix Figure S7 Source Data [file 44319_2026_786_MOESM23_ESM.zip › Appendix Figure S7 Source Data/S7D/Venus_LINC-DN.tif]

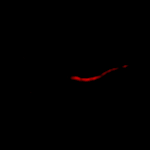

Supplement: Supplementary file 23 — Appendix Figure S7 Source Data [file 44319_2026_786_MOESM23_ESM.zip › Appendix Figure S7 Source Data/S7D/Trim46_LINC-DN.tif]

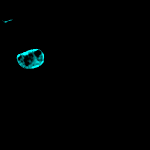

Supplement: Supplementary file 23 — Appendix Figure S7 Source Data [file 44319_2026_786_MOESM23_ESM.zip › Appendix Figure S7 Source Data/S7D/HA_LINC-DN.tif]

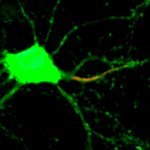

Supplement: Supplementary file 23 — Appendix Figure S7 Source Data [file 44319_2026_786_MOESM23_ESM.zip › Appendix Figure S7 Source Data/S7D/Merge_LINC-DN.tif]
