# Supplementary material for: Age-related decline in nuclear envelope LINC complex drives neuronal aging via axon initial segment dysfunction
Source: EMBO Rep. 2026 May 22;27(13):3788–825. doi: 10.1038/s44319-026-00786-5 (PMC13354796; doi:10.1038/s44319-026-00786-5)
Supplement: Supplementary file 24 — Expanded View Figures [file 44319_2026_786_MOESM24_ESM.pdf]

Expanded View Figures

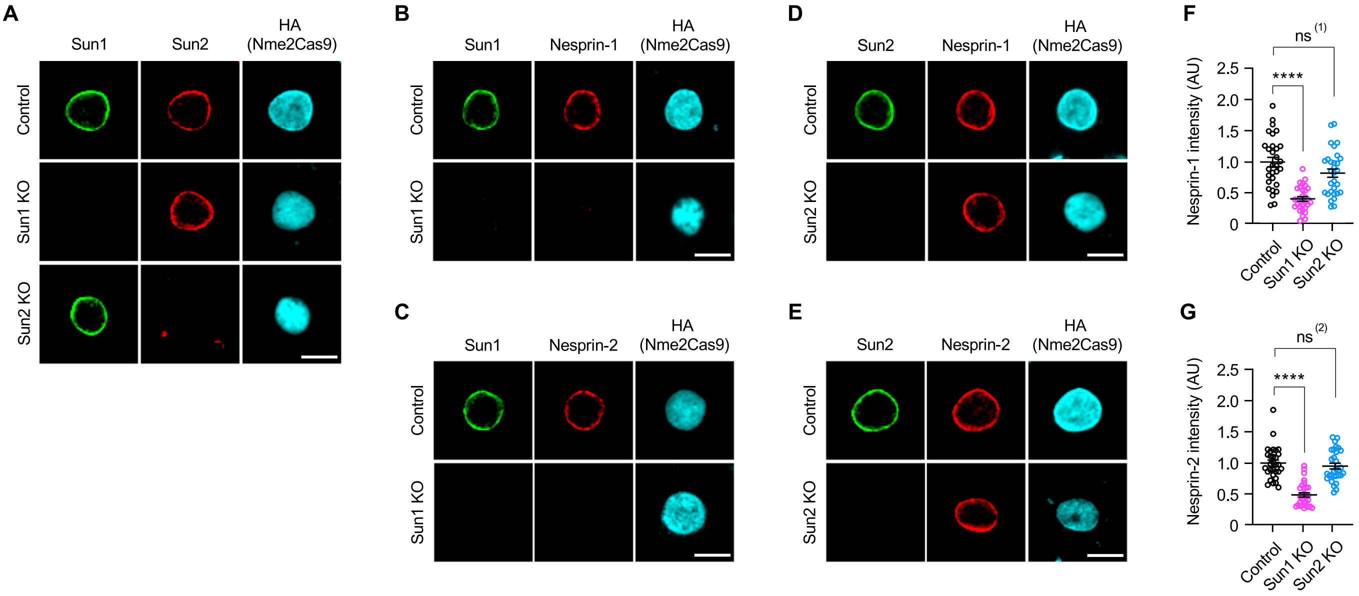

Figure EV1. Effects of Sun deletion on Nesprin expression on the NE.

(**A–E**) Control, Sun1 knockout (KO), and Sun2 KO cortical neurons at 21 DIV. (**F**, **G**) Signal intensity of Nesprin-1 (**F**) and Nesprin-2 (**G**) on the NE was quantified. The data represent the mean ± SEM. $n = 30$ cells from three independent experiments (Control, Sun1 KO, and Sun2 KO) (**F**, **G**). $^{****}P < 0.0001$; ns, not significant, $P = 0.0822$ (1), 0.6530 (2) (ordinary one-way ANOVA Dunnett's multiple comparison test). Scale bars: 10 µm. Source data are available online for this figure.

 © The Author(s)

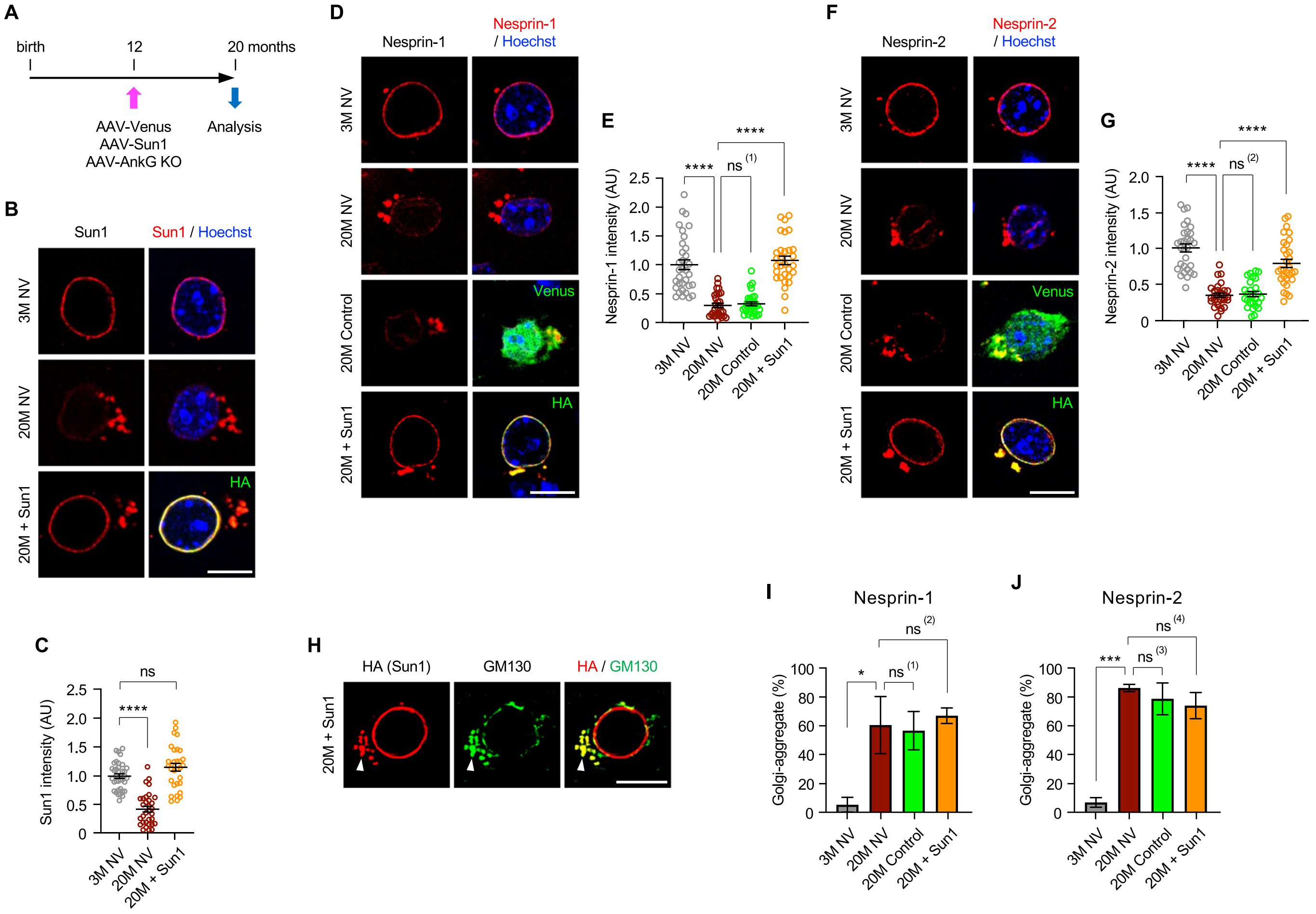

Figure EV2. Analysis of LINC complex expression in AAV-administered mice.

(**A**) Schematic of the AAV experiment to investigate the effects of Sun1 introduction and AIS disruption in aged neurons. 12-month-old mice were infected with AAV-Venus (20 M Control), AAV-Sun1 (20 M + Sun1), AAV-AnkG KO (20 M + AnkG KO), or AAV-Sun1 plus AAV-AnkG KO (20 M + Sun1 + AnkG KO), and analyzed at 20 months of age. (**B**, **C**) Analysis of Sun1 expression. Representative images are shown for layer V pyramidal neurons in the prefrontal cortex from 3- and 20-month-old mice in no virus (3 M NV or 20 M NV) and AAV-Sun1 (20 M + Sun1) groups (**B**). Signal intensity of Sun1 on the NE was quantified (**C**). The data represent the mean ± SEM. $n = 37$ (3 M NV), $n = 32$ (20 M NV), and $n = 31$ cells (20 M + Sun1) from three brains. ****$P < 0.0001$; ns, not significant, $P = 0.0708$ (ordinary one-way ANOVA Dunnett's multiple comparison test). (**D–G**) Analysis of Nesprin expression in young and aged neurons. Brain sections from 3- or 20-month-old mice in no virus (3 M NV or 20 M NV), AAV-Venus (20 M Control), and AAV-Sun1 (20 M + Sun1) groups were co-immunostained with antibodies against Nesprin-1 (**D**) or Nesprin-2 (**F**), along with HA (for Sun1). Representative images are shown for layer V pyramidal neurons in the prefrontal cortex. Signal intensity of Nesprin-1 (**E**) and Nesprin-2 (**G**) on the NE were quantified. The data represent the mean ± SEM. $n = 33$ (3 M NV), $n = 29$ (20 M NV), $n = 30$ (20 M Control), and $n = 30$ (20 M + Sun1) for Nesprin-1 (**E**); $n = 30$ (3 M NV), $n = 29$ (20 M NV), $n = 27$ (20 M Control), and $n = 31$ cells (20 M + Sun1) for Nesprin-2 (**G**) from three brains. ****$P < 0.0001$; ns, not significant, $P = 0.9797$ (1), 0.9920 (2) (ordinary one-way ANOVA Dunnett's multiple comparison test). (**H**) Localization of exogenous Sun1 in layer V pyramidal neurons in the prefrontal cortex of 20 M +Sun1 mice. Note that HA-tagged Sun1 localizes at the NE and GM130-labeled Golgi apparatus (arrowheads) in neurons. (**I**, **J**) Quantification of the percentage of cells containing five or more Golgi-localized aggregates (> 0.5 µm) of Nesprin-1 (**I**) and Nesprin-2 (**J**) in the experiments shown in (**D**, **F**). The data represent the mean ± SEM. $n = 3$ brains, 6–13 cells per brain (**I**, **J**). *$P = 0.0347$; ***$P < 0.0005$; ns, not significant, $P = 0.9919$ (1), 0.9658 (2), 0.8204 (3), 0.5478 (4) (ordinary one-way ANOVA Dunnett's multiple comparison test). Scale bars: 10 µm. Source data are available online for this figure.

© The Author(s)

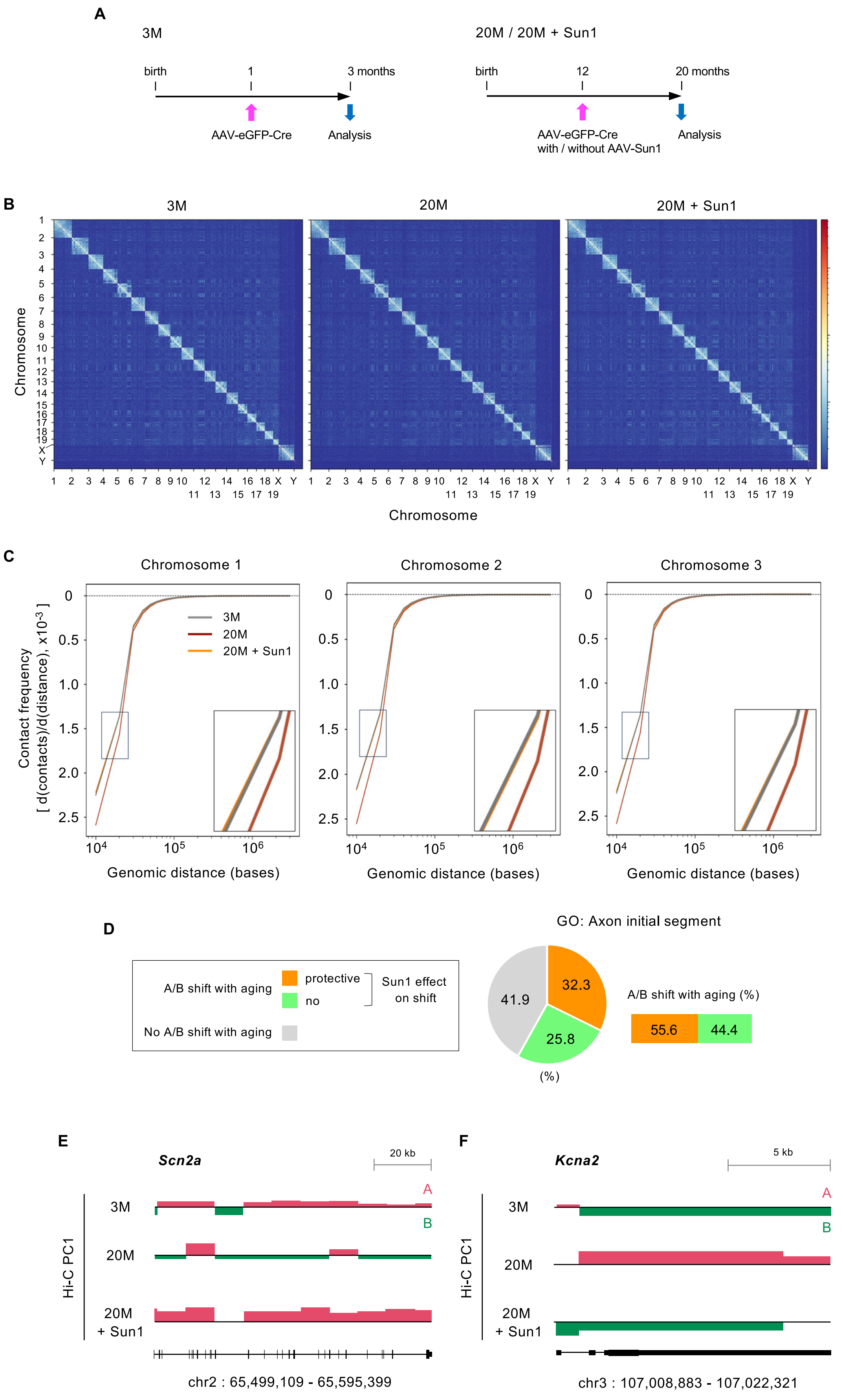
A
3M
birth
1
3 months
AAV-eGFP-Cre
Analysis
20M / 20M + Sun1
birth
12
20 months
AAV-eGFP-Cre
with / without AAV-Sun1
Analysis
B
3M
20M
20M + Sun1
Chromosome
C
Chromosome 1
Chromosome 2
Chromosome 3
3M
20M
20M + Sun1
Contact frequency
[d(contacts)/d(distance), x10-3]
Genomic distance (bases)
D
A/B shift with aging
protective
no
Sun1 effect on shift
No A/B shift with aging
GO: Axon initial segment
41.9
32.3
25.8
(%)
A/B shift with aging (%)
55.6
44.4
E
Scn2a
20 kb
F
Kcna2
5 kb
Hi-C PC1
3M
20M
20M + Sun1
A
B
chr2 : 65,499,109 - 65,595,399
chr3 : 107,008,883 - 107,022,321

 © The Author(s)

Figure EV3. Analysis of genome-wide chromatin structure in young and aged neurons.

(**A**) Schematics of the AAV experiment for genome analyses. 1- or 12-month-old mice were infected with AAV-eGFP-Cre to label the neuronal nuclei, and analyzed at 3 or 20 months of age, respectively (3 M or 20 M). In a separate group, 12-month-old mice were co-infected with AAV-Sun1 and AAV-eGFP-Cre, and analyzed at 20 months of age (20 M + Sun1). (**B**) Contact map across the whole genome in AAV-eGFP-Cre alone (3 M or 20 M) and AAV-eGFP-Cre + AAV-Sun1 (20 M + Sun1) groups.
(**C**) Distance-dependent contact frequency profiles on chromosomes 1–3. The boxed regions are magnified in the inset. d(contacts)/d(distance) represents the derivative of the contact number according to distance. (**D**) Percentage of age-related A/B compartment shift for the GO term "Axon initial segment." Note that 58.1% of the genes undergo A/B shifts with aging (indicated in orange and green in the left pie chart), whereas 55.6% of these shifts are inhibited by Sun1 (indicated in orange in the right bar graph). (**E**, **F**) A/B compartment analysis of the *Scn2a* (**E**) and *Kcna2* (**F**) loci. Source data are available online for this figure.

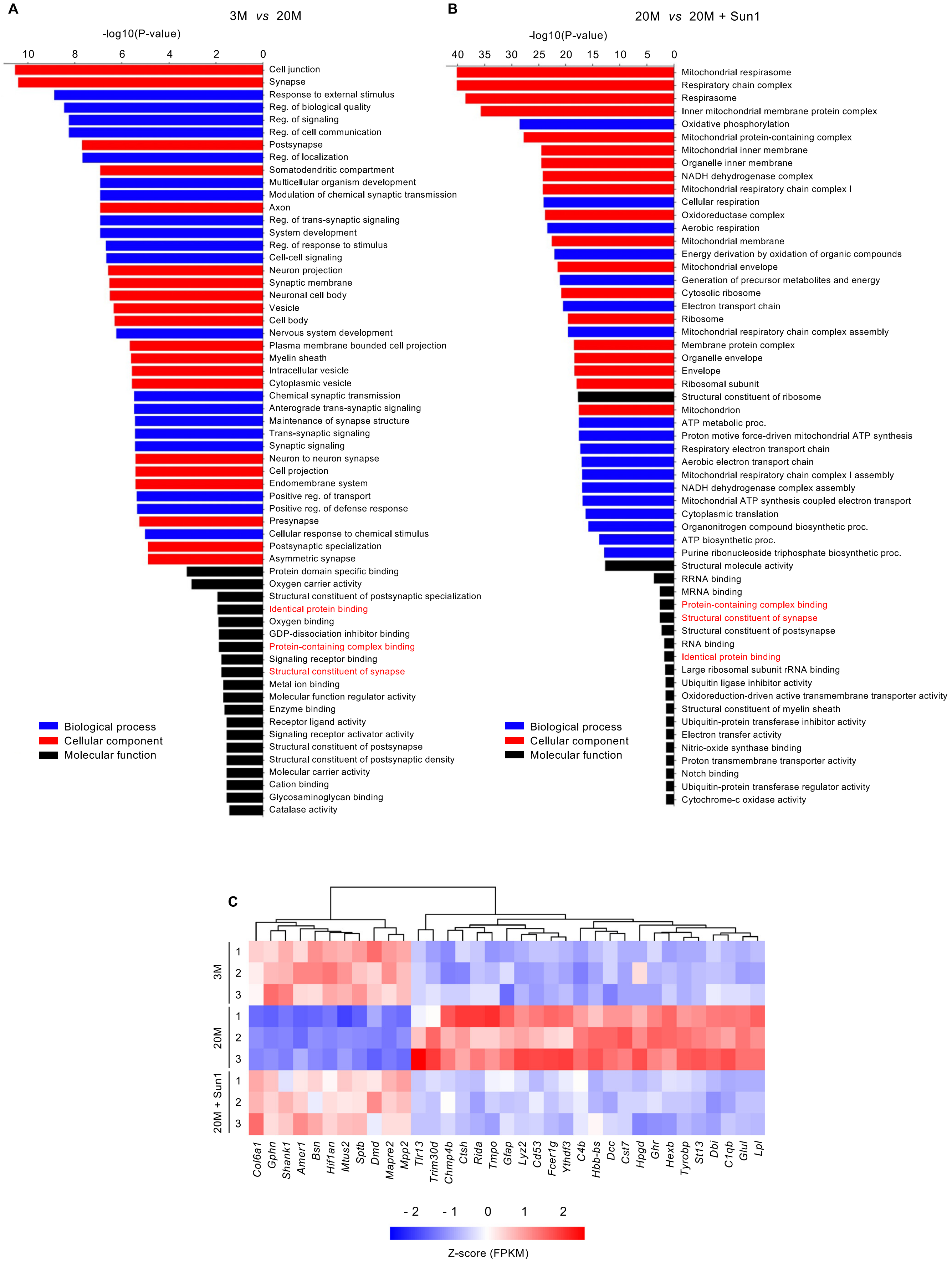
A
3M vs 20M
-log10(P-value)
10
8
6
4
2
0
Cell junction
Synapse
Response to external stimulus
Reg. of biological quality
Reg. of signaling
Reg. of cell communication
Postsynapse
Reg. of localization
Somatodendritic compartment
Multicellular organism development
Modulation of chemical synaptic transmission
Axon
Reg. of trans-synaptic signaling
System development
Reg. of response to stimulus
Cell-cell signaling
Neuron projection
Synaptic membrane
Neuronal cell body
Vesicle
Cell body
Nervous system development
Plasma membrane bounded cell projection
Myelin sheath
Intracellular vesicle
Cytoplasmic vesicle
Chemical synaptic transmission
Anterograde trans-synaptic signaling
Maintenance of synapse structure
Trans-synaptic signaling
Synaptic signaling
Neuron to neuron synapse
Cell projection
Endomembrane system
Positive reg. of transport
Positive reg. of defense response
Presynapse
Cellular response to chemical stimulus
Postsynaptic specialization
Asymmetric synapse
Protein domain specific binding
Oxygen carrier activity
Structural constituent of postsynaptic specialization
Identical protein binding
Oxygen binding
GDP-dissociation inhibitor binding
Protein-containing complex binding
Signaling receptor binding
Structural constituent of synapse
Metal ion binding
Molecular function regulator activity
Enzyme binding
Receptor ligand activity
Signaling receptor activator activity
Structural constituent of postsynapse
Structural constituent of postsynaptic density
Molecular carrier activity
Cation binding
Glycosaminoglycan binding
Catalase activity
Biological process
Cellular component
Molecular function
B
20M vs 20M + Sun1
-log10(P-value)
40
35
30
25
20
15
10
5
0
Mitochondrial respirasome
Respiratory chain complex
Respirasome
Inner mitochondrial membrane protein complex
Oxidative phosphorylation
Mitochondrial protein-containing complex
Mitochondrial inner membrane
Organelle inner membrane
NADH dehydrogenase complex
Mitochondrial respiratory chain complex I
Cellular respiration
Oxidoreductase complex
Aerobic respiration
Mitochondrial membrane
Energy derivation by oxidation of organic compounds
Mitochondrial envelope
Generation of precursor metabolites and energy
Cytosolic ribosome
Electron transport chain
Ribosome
Mitochondrial respiratory chain complex assembly
Membrane protein complex
Organelle envelope
Envelope
Ribosomal subunit
Structural constituent of ribosome
Mitochondrion
ATP metabolic proc.
Proton motive force-driven mitochondrial ATP synthesis
Respiratory electron transport chain
Aerobic electron transport chain
Mitochondrial respiratory chain complex I assembly
NADH dehydrogenase complex assembly
Mitochondrial ATP synthesis coupled electron transport
Cytoplasmic translation
Organonitrogen compound biosynthetic proc.
ATP biosynthetic proc.
Purine ribonucleoside triphosphate biosynthetic proc.
Structural molecule activity
RRNA binding
MRNA binding
Protein-containing complex binding
Structural constituent of synapse
Structural constituent of postsynapse
RNA binding
Identical protein binding
Large ribosomal subunit rRNA binding
Ubiquitin ligase inhibitor activity
Oxidoreduction-driven active transmembrane transporter activity
Structural constituent of myelin sheath
Ubiquitin-protein transferase inhibitor activity
Electron transfer activity
Nitric-oxide synthase binding
Proton transmembrane transporter activity
Notch binding
Ubiquitin-protein transferase regulator activity
Cytochrome-c oxidase activity
Biological process
Cellular component
Molecular function
C
3M
1
2
3
20M
1
2
3
20M + Sun1
1
2
3
Col6a1
Gphn
Shank1
Amer1
Bsn
Hif1an
Mtus2
Sptb
Dmd
Mapre2
Mpp2
Tlr13
Trim30d
Chmp4b
Ctsh
Rida
Tmpo
Gfap
Lyz2
Cd53
Fcer1g
Ythdf3
C4b
Hbb-bs
Dcc
Cst7
Hpgd
Ghr
Hexb
Tyrobp
St13
Dbi
C1qb
Glul
Lpl
- 2
- 1
0
1
2
Z-score (FPKM)

 © The Author(s)

Figure EV4. Analysis of gene expression in young and aged neurons.

(**A**, **B**) GO analysis for AAV-eGFP-Cre alone (3 M or 20 M) and AAV-eGFP-Cre + AAV-Sun1 (20 M + Sun1) groups. The three GO terms highlighted in red showed significant expression changes in both 3 M *vs* 20 M (**A**) and 20 M *vs* 20 M + Sun1 (**B**). *P* values were derived from hypergeometric tests and adjusted using the Benjamini–Hochberg method. (**C**) Heatmap of clustered DEGs associated with GO terms highlighted in red in (**A**, **B**). The line above the heatmap indicates the classification of expression similarity of the 3 M and 20 M + Sun1 groups relative to the 20 M group. Each group consists of three mice. DEGs were defined as those with |log2(fold_change)| >1, $P < 0.001$. FPKM values were subjected to gene-wise z-score normalization before generating the heatmap. Source data are available online for this figure.

© The Author(s)

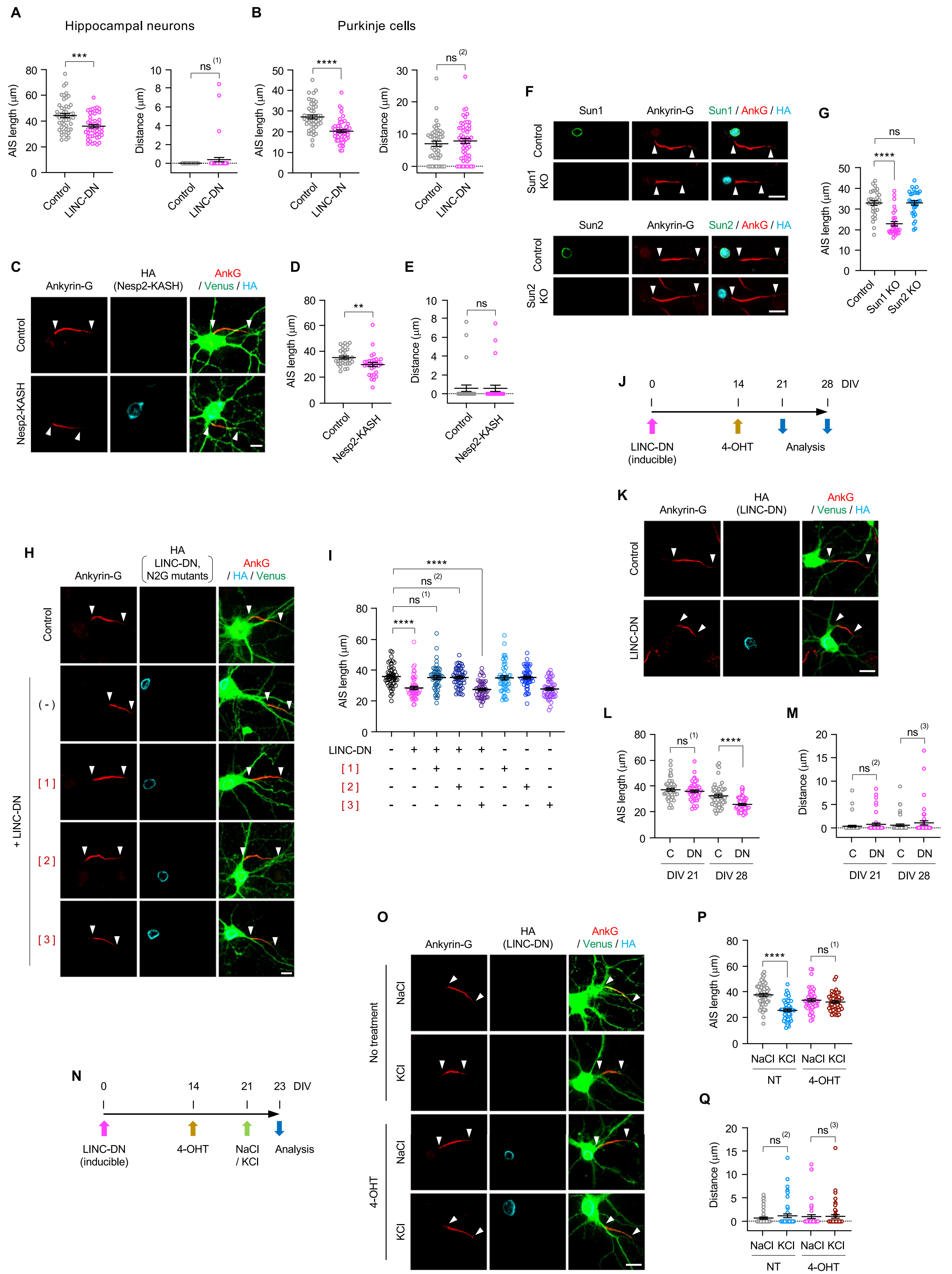
A
Hippocampal neurons
AIS length (μm)
Distance (μm)
Control
LINC-DN
B
Purkinje cells
F
Sun1
Ankyrin-G
Sun1 / AnkG / HA
Sun1 KO
Sun2
Sun2 / AnkG / HA
Sun2 KO
G
Control
Sun1 KO
Sun2 KO
C
Ankyrin-G
HA (Nesp2-KASH)
AnkG / Venus / HA
Nesp2-KASH
D
E
J
0 14 21 28 DIV
LINC-DN (inducible)
4-OHT
Analysis
K
HA (LINC-DN)
H
HA LINC-DN, N2G mutants
AnkG / HA / Venus
+ LINC-DN
I
L
M
DIV 21
DIV 28
O
No treatment
NaCl
KCl
4-OHT
P
NT
Q
N
0 14 21 23 DIV
NaCl / KCl

 © The Author(s)

Figure EV5. Effects of LINC complex inhibition on the AIS in vitro.

(**A**, **B**) Quantification of AIS length and position, measured as the distance from the soma, in control and LINC-DN-expressing hippocampal neurons at 21 DIV (**A**) and Purkinje cells at 7 DIV (**B**). The data represent the mean ± SEM. $n = 50$ (Control and LINC-DN) (**A**); $n = 47$ (Control) and $n = 55$ cells (LINC-DN) (**B**) from three independent experiments. ***$P < 0.0005$; ****$P < 0.0001$ (unpaired two-tailed Welch's t test for the left graphs in (**A**, **B**)). ns, not significant, $P = 0.2424$ (1), 0.4637 (2) (unpaired two-tailed Mann–Whitney test for the right graphs in (**A**, **B**)). (**C–E**) Effects of Nesp2-KASH expression on the AIS. Representative images of control and Nesp2-KASH-expressing cortical neurons at 21 DIV are shown (**C**). The AIS is indicated by the two arrowheads, and its length (**D**) and position (**E**) were quantified. The data represent the mean ± SEM. $n = 30$ cells from three independent experiments (Control and Nesp2-KASH) (**D**, **E**). **$P = 0.0089$ (unpaired two-tailed Welch's t test for (**D**)). ns, not significant, $P > 0.9999$ (unpaired two-tailed Mann–Whitney test for (**E**)). (**F**, **G**) Effects of Sun deletions on the AIS. Representative images of control, Sun1 knockout (KO), and Sun2 KO cortical neurons at 21 DIV are shown (**F**). Nme2Cas9 (HA-tagged) was detected by immunostaining with an anti-HA antibody. The AIS is indicated by the two arrowheads, and its length was quantified (**G**). The data represent the mean ± SEM. $n = 30$ cells from three independent experiments (Control, Sun1 KO, and Sun2 KO). ****$P < 0.0001$; ns, not significant, $P = 0.9995$ (ordinary one-way ANOVA Dunnett's multiple comparison test). (**H**, **I**) Analysis of cytoskeletal interactions essential for LINC complex-mediated regulation of the AIS. Mini N2G SR52-56, mini N2G SR55-56, or N2G SR52-56 (see schematic shown in Fig. 2C) were expressed in cortical neurons with or without LINC-DN and analyzed at 21 DIV for AIS structure (**H**). The AIS is indicated by the two arrowheads, and its length was quantified (**I**). The data represent the mean ± SEM. $n = 50$ cells from three independent experiments (all groups). ****$P < 0.0001$; ns, not significant, $P = 0.9831$ (1), 0.9973 (2) (ordinary one-way ANOVA Dunnett's multiple comparison test). (**J–M**) Effects of LINC complex inhibition on AIS structure in mature neurons. Cortical neurons were induced to express LINC-DN by the addition of 4-OHT at 14 DIV and cultured for an additional 7 or 14 days (**J**). Representative images of neurons at 28 DIV are shown (**K**). The AIS is indicated by the two arrowheads, and its length (**L**) and position (**M**) were quantified. C, Control; DN, LINC-DN. The data represent the mean ± SEM. $n = 51$ (C, 21 DIV), $n = 50$ (DN, 21 DIV), $n = 50$ (C, 28 DIV), and $n = 50$ cells (DN, 28 DIV) (**L**, **M**) from three independent experiments. ****$P < 0.0001$; ns, not significant, $P = 0.4454$ (1), 0.1778 (2), 0.3621 (3) (unpaired two-tailed Welch's t test for (**L**)). ns, not significant (unpaired two-tailed Mann–Whitney test for (**M**)). (**N–Q**) Effects of LINC complex inhibition on the structural plasticity of the AIS in mature neurons. Cortical neurons were induced to express LINC-DN by the addition of 4-OHT at 14 DIV and cultured for an additional 7 days, followed by treatment with 10 mM NaCl or 10 mM KCl for 48 h (**N**, **O**). The AIS is indicated by the two arrowheads, and its length (**P**) and position (**Q**) were quantified. NT no treatment. The data represent the mean ± SEM. $n = 51$ (NT, NaCl), $n = 52$ (NT, KCl), $n = 50$ (4-OHT, NaCl), and $n = 50$ cells (4-OHT, KCl) (**P**, **Q**) from three independent experiments. ****$P < 0.0001$; ns, not significant, $P = 0.3741$ (1), 0.8195 (2), 0.4115 (3) (unpaired two-tailed Welch's t test for (**P**)). ns, not significant (unpaired two-tailed Mann–Whitney test for (**Q**)). Scale bars: 10 µm. Source data are available online for this figure.

© The Author(s)

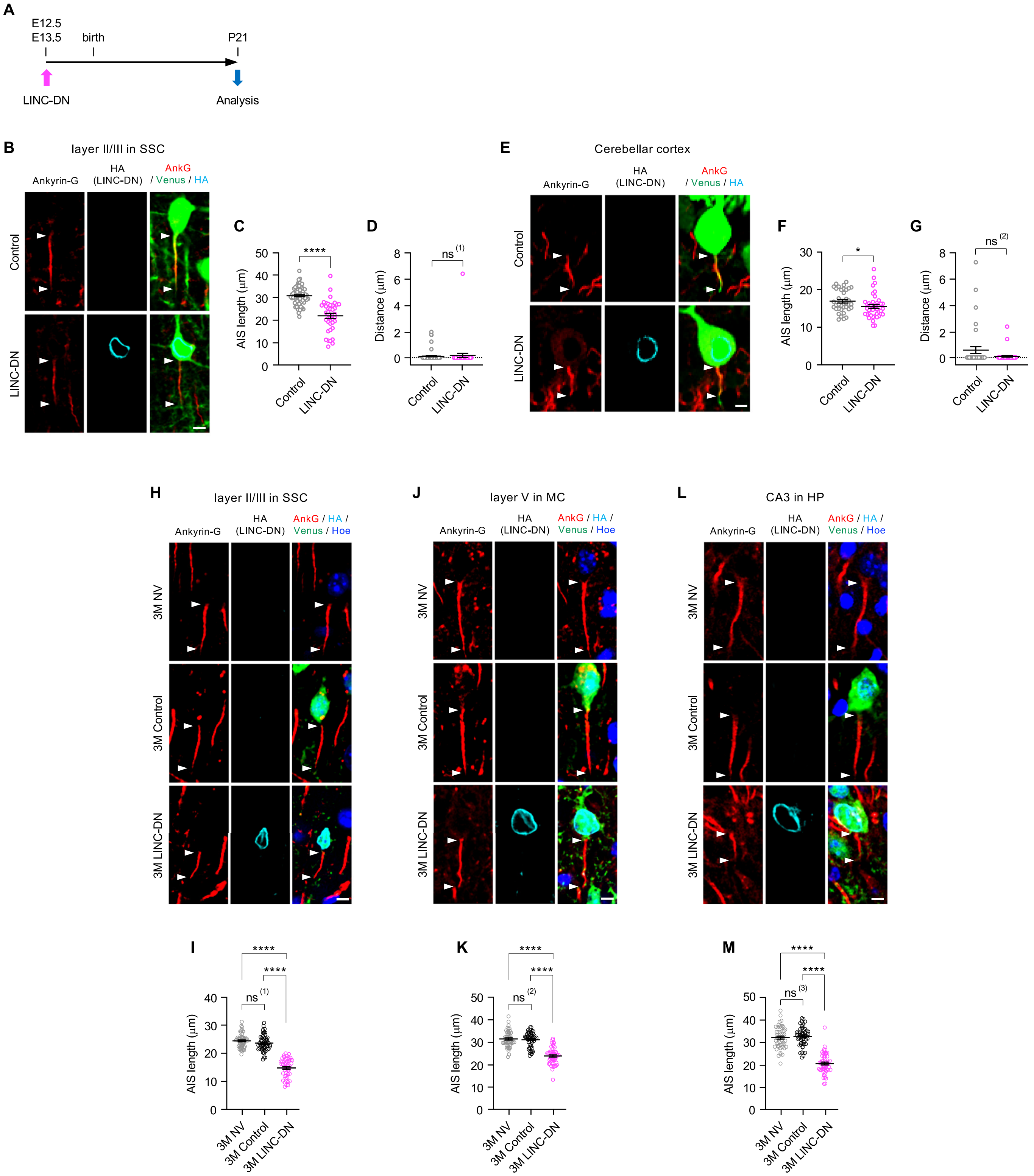
A
E12.5
E13.5
birth
P21
LINC-DN
Analysis
B
layer II/III in SSC
Ankyrin-G
HA (LINC-DN)
AnkG / Venus / HA
Control
LINC-DN
C
AIS length (µm)
****
D
Distance (µm)
ns (1)
E
Cerebellar cortex
F
*
G
ns (2)
H
layer II/III in SSC
AnkG / HA / Venus / Hoe
3M NV
3M Control
3M LINC-DN
J
layer V in MC
L
CA3 in HP
I
K
M
ns (3)

© The Author(s)

Figure EV6. Effects of LINC complex inhibition on the AIS in vivo.

(**A–G**) Effects of embryonic LINC-DN expression on the AIS. LINC-DN was expressed in neurons using in utero electroporation (**A**). Representative images are shown for layer II/III pyramidal neurons in the somatosensory cortex (**B**) and Purkinje cells in the cerebellum (**E**). The AIS is indicated by the two arrowheads. AIS length (**C**, **F**) and position (**D**, **G**), measured as the distance from the soma, were quantified. The data represent the mean ± SEM. $n = 72$ (Control) and $n = 39$ cells (LINC-DN) (**C**, **D**) from four brains; $n = 37$ (Control) and $n = 42$ cells (LINC-DN) (**F**, **G**) from six brains. *$P = 0.0472$; ****$P < 0.0001$ (unpaired two-tailed Welch's t test for (**C**, **F**)). ns, not significant, $P = 0.4226$ (1), 0.1258 (2) (unpaired two-tailed Mann–Whitney test for (**D**, **G**)). (**H–M**) Effects of postnatal LINC-DN expression on the AIS. LINC-DN was expressed in neurons of 1.5-month-old mice via AAV delivery. Representative images are shown for layer II/III pyramidal neurons in the somatosensory cortex (SSC) (**H**), layer V pyramidal neurons in the motor cortex (MC) (**J**), and CA3 neurons in the hippocampus (HP) (**L**) from 3-month-old mice in no virus (3 M NV), AAV-Venus alone (3 M Control), and AAV-Venus + AAV-LINC-DN (3 M LINC-DN) groups. The AIS is indicated by the two arrowheads, and its length was quantified (**I**, **K**, **M**). The data represent the mean ± SEM. $n = 55$ (3 M NV), $n = 53$ (3 M Control), and $n = 47$ (3 M LINC-DN) (**I**); $n = 51$ (3 M NV), $n = 50$ (3 M Control), and $n = 51$ (3 M LINC-DN) (**K**); $n = 51$ (3 M NV), $n = 50$ (3 M Control), and $n = 51$ cells (3 M LINC-DN) (**M**) from three brains. ****$P < 0.0001$; ns, not significant, $P = 0.3183$ (1), 0.8891 (2), 0.8679 (3) (ordinary one-way ANOVA Tukey's multiple comparison test). Scale bars: 10 µm. Source data are available online for this figure.

© The Author(s)

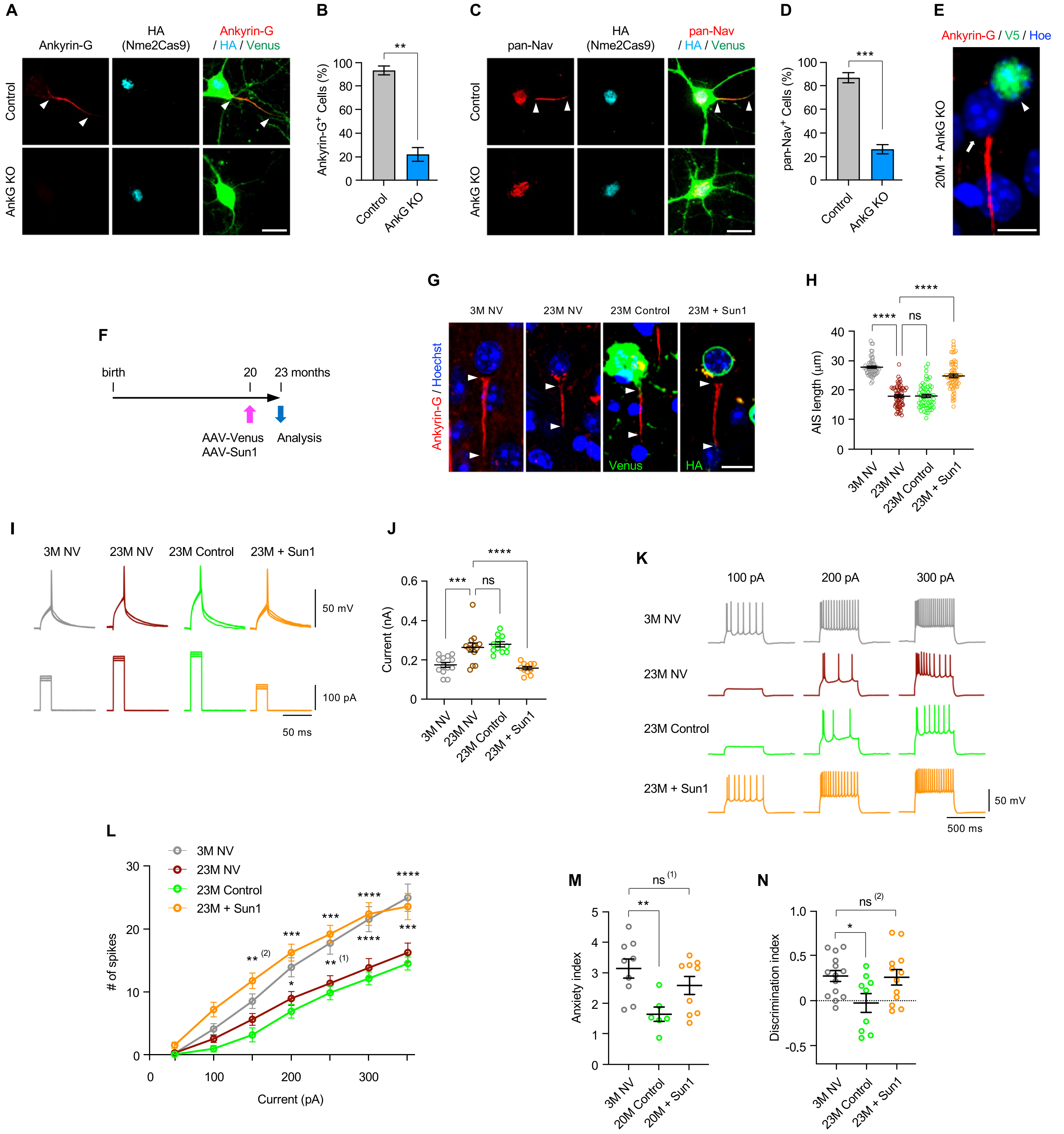
A
Ankyrin-G
HA (Nme2Cas9)
Ankyrin-G / HA / Venus
Control
AnkG KO
B
**
Ankyrin-G+ Cells (%)
Control
AnkG KO
C
pan-Nav
HA (Nme2Cas9)
pan-Nav / HA / Venus
Control
AnkG KO
D
***
pan-Nav+ Cells (%)
Control
AnkG KO
E
Ankyrin-G / V5 / Hoe
20M + AnkG KO
F
birth
20
23 months
AAV-Venus
AAV-Sun1
Analysis
G
3M NV
23M NV
23M Control
23M + Sun1
Ankyrin-G / Hoechst
Venus
HA
H
****
****
ns
AIS length (μm)
3M NV
23M NV
23M Control
23M + Sun1
I
3M NV
23M NV
23M Control
23M + Sun1
50 mV
100 pA
50 ms
J
****
***
ns
Current (nA)
3M NV
23M NV
23M Control
23M + Sun1
K
100 pA
200 pA
300 pA
3M NV
23M NV
23M Control
23M + Sun1
50 mV
500 ms
L
3M NV
23M NV
23M Control
23M + Sun1
# of spikes
Current (pA)
** (2)
*
*** (1)
M
ns (1)
**
Anxiety index
3M NV
20M Control
20M + Sun1
N
ns (2)
*
Discrimination index
3M NV
23M Control
23M + Sun1

 © The Author(s)

Figure EV7. Analysis of AIS length, neuronal excitability, and brain function in aged mice.

(**A–D**) Analysis of Ankyrin-G deletion in neurons in vitro. Control and Ankyrin-G knockout (AnkG KO) cortical neurons at 21 DIV (**A**, **C**). The AIS is indicated by the two arrowheads. Ankyrin-G-positive cells (**B**) and cells exhibiting AIS-localized pan-Nav signal (**D**) were quantified. The data represent the mean ± SEM. $n = 3$ independent experiments, eight–fifteen cells per experiment (**B**, **D**). $^{**}P = 0.0011$; $^{***}P < 0.001$ (unpaired two-tailed Welch's t test). (**E**) Analysis of Ankyrin-G deletion in neurons in vivo. Representative image of layer V pyramidal neurons in the prefrontal cortex of 20-month-old mice administered AAV-AnkG KO (20 M + AnkG KO). The arrowhead and arrow indicate Nme2Cas9-positive and -negative neurons, respectively. Note that the Ankyrin-G signal is specifically lost in Nme2Cas9-positive neurons. The efficiency of Ankyrin-G deletion is 81.6% ± 0.2 ($n = 3$) and 82.0% ± 1.1 ($n = 3$) in the prefrontal and somatosensory cortices, respectively. (**F**) Schematic of the AAV experiment to investigate the effects of Sun1 introduction in aged neurons. 20-month-old mice were infected with AAV-Venus (23 M Control) or AAV-Sun1 (23 M + Sun1), and analyzed at 23 months of age. (**G**, **H**) Analysis of AIS structure in 3- or 23-month-old mice in no virus (3 M NV or 23 M NV), AAV-Venus (23 M Control), and AAV-Sun1 (23 M + Sun1) groups. Brain sections from the indicated groups were co-immunostained with antibodies against Ankyrin-G and HA (for Sun1). Representative images are shown for layer V pyramidal neurons in the prefrontal cortex (**G**). The AIS is indicated by the two arrowheads, and its length was quantified (**H**). The data represent the mean ± SEM. $n = 60$ (3 M NV), $n = 60$ (23 M NV), $n = 58$ (23 M Control), and n = 60 cells (23 M + Sun1) from three brains. $^{****}P < 0.0001$; ns, not significant, $P = 0.9996$ (ordinary one-way ANOVA Dunnett's multiple comparison test). (**I–L**) Representative recordings in layer V pyramidal neurons in the prefrontal cortex of 3- or 23-month-old mice from the same groups as shown in (**G**, **H**): responses to a 20-ms current injection (**I**) and spiking activity during 500-ms current injections at 100, 200, and 300 pA (**K**). Current threshold (**J**) and firing frequency (**L**) were quantified. The data represent the mean ± SEM. For current threshold (**J**): $n = 13$ (3 M NV), $n = 13$ (23 M NV), $n = 11$ (23 M Control), and n = 11 cells (23 M + Sun1) from 6 to 11 brains. $^{***}P < 0.001$; $^{****}P < 0.0001$; ns, not significant, $P = 0.8224$ (ordinary one-way ANOVA Dunnett's multiple comparison test). For firing frequency (**L**): $n = 17$ (3 M NV), $n = 13$ (23 M NV), $n = 11$ (23 M Control), and $n = 11$ cells (23 M + Sun1) from 6 to 11 brains. $^{*}P = 0.0167$; $^{**}P = 0.0012$ (1), 0.0063 (2); $^{***}P < 0.001$; $^{****}P < 0.0001$ (two-way ANOVA Dunnett's multiple comparison test *vs* 23 M NV). (**M**, **N**) Behavioral analyses. The elevated plus maze test was performed on 3- or 20-month-old mice in no virus (3 M NV), AAV-Venus (20 M Control), and AAV-Sun1 (20 M + Sun1) groups (**M**). Novel object recognition test was performed on 3- or 23-month-old mice in no virus (3 M NV), AAV-Venus (23 M Control), and AAV-Sun1 (23 M + Sun1) groups (**N**). The data represent the mean ± SEM. $n = 9$ (3 M NV), $n = 6$ (20 M Control), and $n = 9$ (20 M + Sun1) (**M**); $n = 14$ (3 M NV), $n = 9$ (23 M Control), and $n = 12$ mice (23 M + Sun1) (**N**). $^{*}P = 0.0308$; $^{**}P = 0.0056$; ns, not significant, $P = 0.2995$ (1), 0.9890 (2) (ordinary one-way ANOVA Dunnett's multiple comparison test). Scale bars: 10 µm. Source data are available online for this figure.

© The Author(s)
