# Supplementary material for: Age-related decline in nuclear envelope LINC complex drives neuronal aging via axon initial segment dysfunction
Source: EMBO Rep. 2026 May 22;27(13):3788–825. doi: 10.1038/s44319-026-00786-5 (PMC13354796; doi:10.1038/s44319-026-00786-5)
Supplement: Supplementary file 2 — Peer Review File [file 44319_2026_786_MOESM2_ESM.pdf]

EMBO reports

Age-related decline in nuclear envelope LINC complex drives neuronal aging via axon initial segment dysfunction

Koichi Hasegawa, Noriyuki Hama, Mina Amemiya, Chao Zeng, Yasuyuki Ito, Sadafumi Suzuki, Keiichiro Nakamura, Junpei Kondo, Chiharu Takeda, Yuji Kurihara, Kazuho Ikeda, Yuki Fujita, Yasushi Okada, Atsushi Toyoda, Michiaki Hamada, and Ken-ichiro Kuwako

Corresponding author(s): Ken-ichiro Kuwako (kuwako@med.shimane-u.ac.jp)

Review Timeline:

Submission Date:	23rd Sep 25
Editorial Decision:	17th Nov 25
Revision Received:	4th Jan 26
Editorial Decision:	12th Mar 26
Revision Received:	31st Mar 26
Accepted:	13th Apr 26

Editor: Martina Rembold

Transaction Report:

(Note: With the exception of the correction of typographical or spelling errors that could be a source of ambiguity, letters and reports are not edited. Depending on transfer agreements, referee reports obtained elsewhere may or may not be included in this compilation. Referee reports are anonymous unless the Referee chooses to sign their reports.)

Dear Prof. Kuwako

Thank you for the submission of your research manuscript to our journal. Three referees agreed to review your manuscript. So far, we have received two referee reports that are copied below. Given that both referees are in fair agreement that you should be given a chance to revise the manuscript, I would like to ask you to begin revising your study along the lines suggested by the referees.

Please note that this is a preliminary decision made in the interest of time, and that it is subject to change should the third referee offer very strong and convincing reasons for this. As soon as we receive the final report on your manuscript, we will forward it to you as well.

Please address all referee concerns in a complete point-by-point response. Acceptance of the manuscript will depend on a positive outcome of a second round of review. It is EMBO Reports policy to allow a single round of revision only and acceptance or rejection of the manuscript will therefore depend on the completeness of your responses included in the next, final version of the manuscript.

We realize that it is difficult to revise to a specific deadline. In the interest of protecting the conceptual advance provided by the work, we recommend a revision within 3 months (February 17, 2026). Please discuss the revision progress ahead of this time with the editor if you require more time to complete the revisions.

I am also happy to discuss the revision further via e-mail or a video call, if you wish.

================

IMPORTANT NOTE:

We perform an initial quality control of all revised manuscripts before re-review. Your manuscript will FAIL this control and the handling will be delayed IN CASE the following APPLIES:

1) A data availability section providing access to data deposited in public databases is missing. If you have not deposited any data, please add a sentence to the data availability section that explains that.

2) Your manuscript contains statistics and error bars based on n=2. Please use scatter blots in these cases. No statistics should be calculated if n=2.

When submitting your revised manuscript, please carefully review the instructions that follow below. Failure to include requested items will delay the evaluation of your revision.

================

When submitting your revised manuscript, we will require:

1) a .docx formatted version of the manuscript text (including legends for main figures, EV figures and tables). Please make sure that the changes are highlighted to be clearly visible.

2) individual production quality figure files as .eps, .tif, .jpg (one file per figure).
Please download our Figure Preparation Guidelines (figure preparation pdf) from our Author Guidelines pages https://www.embopress.org/page/journal/14693178/authorguide for more info on how to prepare your figures.

3) a .docx formatted letter INCLUDING the reviewers' reports and your detailed point-by-point responses to their comments. As part of the EMBO Press transparent editorial process, the point-by-point response is part of the Review Process File (RPF), which will be published alongside your paper.

4) a complete author checklist, which you can download from our author guidelines (<https://www.embopress.org/page/journal/14693178/authorguide>). Please insert information in the checklist that is also reflected in the manuscript. The completed author checklist will also be part of the RPF.

5) Please note that all corresponding authors are required to supply an ORCID ID for their name upon submission of a revised manuscript (<https://orcid.org/>). Please find instructions on how to link your ORCID ID to your account in our manuscript tracking system in our Author guidelines

(<https://www.embopress.org/page/journal/14693178/authorguide#authorshipguidelines>)

6) We replaced Supplementary Information with Expanded View (EV) Figures and Tables that are collapsible/expandable online. A maximum of 5 EV Figures can be typeset. EV Figures should be cited as 'Figure EV1, Figure EV2" etc... in the text and their respective legends should be included in the main text after the legends of regular figures.

- For the figures that you do NOT wish to display as Expanded View figures, they should be bundled together with their legends in a single PDF file called *Appendix*, which should start with a short Table of Content. Appendix figures should be referred to in the main text as: "Appendix Figure S1, Appendix Figure S2" etc. See detailed instructions regarding expanded view here: <https://www.embopress.org/page/journal/14693178/authorguide#expandedview>

- Additional Tables/Datasets should be labeled and referred to as Table EV1, Dataset EV1, etc. Legends have to be provided in a separate tab in case of .xls files. Alternatively, the legend can be supplied as a separate text file (README) and zipped together with the Table/Dataset file.

7) Before submitting your revision, primary datasets (and computer code, where appropriate) produced in this study need to be deposited in an appropriate public database (see < https://www.embopress.org/page/journal/14693178/authorguide#dataavailability>).

Please remember to provide a reviewer password if the datasets are not yet public.

The accession numbers and database should be listed in a formal "Data Availability " section (placed after Materials & Method) that follows the model below (see also < https://www.embopress.org/page/journal/14693178/authorguide#dataavailability>). Please note that the Data Availability Section is restricted to new primary data that are part of this study.

Data availability

The datasets (and computer code) produced in this study are available in the following databases:

- RNA-Seq data: Gene Expression Omnibus GSE46843 (https://www.ncbi.nlm.nih.gov/geo/query/acc.cgi?acc=GSE46843)
- [data type]: [name of the resource] [accession number/identifier/doi] ([URL or identifiers.org/DATABASE:ACCESSION])

*** Note - All links should resolve to a page where the data can be accessed. ***

8) At EMBO Press we ask authors to provide source data for the main manuscript figures. You will receive a separate email with instructions for providing source data with your revised manuscript, including how to upload and organize the files.

Additional information on source data and instruction on how to label the files are available <https://www.embopress.org/page/journal/14693178/authorguide#sourcedata>

9) The journal requires a statement specifying whether or not authors have competing interests (defined as all potential or actual interests that could be perceived to influence the presentation or interpretation of an article). In case of competing interests, this must be specified in your disclosure statement. Further information: https://www.embopress.org/competing-interests

10) Figure legends and data quantification:
The following points must be specified in each figure legend:

- the name of the statistical test used to generate error bars and P values,
- the EXACT p-values,
- the number (n) of independent experiments (please specify technical or biological replicates) underlying each data point,
- the nature of the bars and error bars (s.d., s.e.m.)

- If the data are obtained from n {less than or equal to} 5, show the individual data points in addition to the SD or SEM.
- If the data are obtained from n {less than or equal to} 2, use scatter blots showing the individual data points.

Discussion of statistical methodology can be reported in the materials and methods section, but figure legends should contain a basic description of n, P and the test applied.

See also the guidelines for figure legend preparation:
https://www.embopress.org/page/journal/14693178/authorguide#figureformat

- Please also include scale bars in all microscopy images and define their size in the legend, not the image.

11) Our journal encourages inclusion of *data citations in the reference list* to directly cite datasets that were re-used and obtained from public databases. Data citations in the article text are distinct from normal bibliographical citations and should directly link to the database records from which the data can be accessed. In the main text, data citations are formatted as follows: "Data ref: Smith et al, 2001" or "Data ref: NCBI Sequence Read Archive PRJNA342805, 2017". In the Reference list, data citations must be labeled with "[DATASET]". A data reference must provide the database name, accession number/identifiers and a resolvable link to the landing page from which the data can be accessed at the end of the reference. Further instructions are available at <https://www.embopress.org/page/journal/14693178/authorguide#referencesformat>.

12) All Materials and Methods need to be described in the main text using our 'Structured Methods' format. According to this format, the Methods section includes a Reagents and Tools Table (listing key reagents, experimental models, software and relevant equipment and including their sources and relevant identifiers) followed by a Methods and Protocols section describing the methods, ideally using a step-by-step protocol format. The aim is to facilitate adoption of the methodologies across labs. Please download and fill our Reagents and Tools Table template (.docx), which you can find in our author guidelines: https://www.embopress.org/page/journal/14693178/authorguide#structuredmethods.
When submitting your revised manuscript, please do not include the Reagents and Tools Table in the Methods section of the manuscript but upload it as a separate file choosing the file type "Reagent Table".
An example of a Method paper with Structured Methods can be found here:
https://www.embopress.org/doi/10.15252/msb.20178071.

13) As part of the EMBO publication's Transparent Editorial Process, EMBO Reports publishes online a Review Process File to accompany accepted manuscripts. This File will be published in conjunction with your paper and will include the referee reports, your point-by-point response and all pertinent correspondence relating to the manuscript.

You are able to opt out of this by letting the editorial office know (emboreports@embo.org). If you do opt out, the Review Process File link will point to the following statement: "No Review Process File is available with this article, as the authors have chosen not to make the review process public in this case."

We would also welcome the submission of cover suggestions, or motifs to be used by our Graphics Illustrator in designing a cover.

I look forward to seeing a revised form of your manuscript when it is ready and please let me know if you have questions or comments regarding the revision.

Yours sincerely,

Martina Rembold, PhD
Senior Editor
EMBO reports

===================

Referee #2:

In this study, the authors combine microscopy of brain tissue and cultured neurons, genomics, electrophysiology, and mouse behavior to characterize the changes to LINC proteins and nuclear integrity in the brain during aging and how these link to defective AIS function. They find that LINC protein localization at the NE in the brain is decreased in normal aging, which leads to disrupted nuclear integrity and gene expression changes. Interestingly, these changes are dependent on Sun1 as re-expression of Sun1 ameliorates these changes. The authors focus on axon initial segment (AIS) genes, whose expression changes in aged mice in a Sun1-dependent manner, and demonstrate AIS shortening, disrupted segment spacing, and ion channel localization in aged mice as well as neuronal excitability differences that can be rescued with Sun1 expression. This study would be of interest to the neuronal cell biology and aging communities. The combination of different approaches is commendable and overall, most conclusions are supported by the data. However, the manuscript would benefit from multiple points of clarification regarding experimental design, analysis methods and measurements, and figure panels. Further, significant updates to the genomics analyses and interpretation are necessary, and whether Sun1 is directly responsible for the observed impact on AIS structure and function through transcriptomic changes is unclear.

General Comments

1. The authors should improve the clarity of the manuscript by clearly stating the experimental design, including the timeline of AAV expression and harvesting/analysis, for all experiments when they first introduce them.
2. The methods lack details on exactly how some of the analysis was conducted. The authors should ensure that each analysis has a clear description that includes exactly what was measured, such that readers can clearly understand how each plot was generated. As an example, in the "Analysis of AIS" section, the authors state "signal intensities were measured using ImageJ 1.53 .. " However, this does not indicate whether the mean intensities or the total intensity in the entire traced area was measured. It is also unclear from the methods whether the same analysis was conducted for the ion channel intensity measurements as for AIS intensities.
3. When the authors mention "n", they should specify whether the number they indicate refer to animals, cells, AIS, nuclei, etc. For imaging experiments looking at intensity, AIS length, etc., it is best practice to treat n as each animal and conduct statistics on the mean value from each animal (see https://doi.org/10.1083/jcb.202001064). The authors should update their plots and statistical tests as to not overpower their analyses, and clarify how many animals were used in their experiments where they report n as individual cell or AIS.
4. For gene expression analyses, the authors should use DESeq2 (or EdgeR) analysis to report adjusted p-values and log2 fold changes, since expression of each gene is not an independent observation and RNA-seq data are not normally distributed.

Specific Comments

5. On Page 6 the authors write "These results suggest that aging induces neuron-specific changes in the expression of LINC complex components." However, EV3 shows that Sun2 is down in glial cells in 20 months as well, indicating that the changes to LINC complex components are not only in neurons.
6. In Fig 3 and Fig EV 8, instead of plotting the entire p(s) curve for contact frequency, plotting the derivative of the curve would be more helpful in visualizing the fine-scale changes between conditions.
7. The definition of A / B compartments on page 10 should not include information on nuclear positioning, as it is not always true that heterochromatin or silenced genes are at the periphery while active genes are more towards the inner nucleus.
8. In Fig 3F, the distribution of genes in compartment A vs B from 20M+Sun1 mice appear more similar to that of 20M than 3M, and does not really support the authors' conclusion "in the neurons of 20M+Sun1 mice, the distribution of genes in compartments A and B shifted toward the pattern observed in young neurons" is not well supported.
9. The % of genes with A/B compartment shifts (Fig. 3G, Fig. EV8C) are not very informative. ChIP-seq studies (for example, H3K9me3 to follow up on Fig 3A, B) to show changes in histone modifications at specific genes including the AIS genes would be much more informative.
10. The authors should be cautious in directly linking Sun1 to the genomics changes they observe. They show that LaminB intensity is increased in 20M+Sun1 neurons in Fig 2L, and as LaminB is associated with genomic regions (lamina-associated domains), the increased intensity of LaminB may be more directly causing gene expression changes than Sun1. Because the mechanism of how Sun1 reexpression rescues or prevents the nuclear defects of 20M mice is unknown, the authors should adequately discuss the possibility the indirect effects due to Sun1 re-expression.
11. Fig. 3H: It is unclear whether a correlation of 0.94 is meaningfully different from correlation of 1. Volcano plots that show the log fold change in gene expression and clearly reporting how many genes are significantly differentially regulated in all pairwise comparisons would be a much more informative way to reflect the differences.
12. Do the authors have an explanation for why most of the genes in Fig. EV9B (20M vs 20M+Sun1 comparison) are mitochondrial genes, and not nuclear processes related to Sun1?
13. In the methods, the authors state having used 1000 cells total for the RNA-seq experiments. This seem extremely low, even accounting for the sorting step, and may not be sufficient to accurately reflect the gene expression profile of neurons in the entire cerebrum. Is there a reason the authors have used such a low number of input cells?
14. Fig. 3J, the colorbar that goes from -2 to 2 needs to have a legend. Is this fold change or log(FPKM)?
15. For RNA-seq data, the authors should not use statistical test that assume equal variance and/or normal distribution on normalized data. Rather, they should use standard differential gene expression analysis pipelines (such as DEseq2, which they describe using in the methods) to obtain log2 fold change for each gene. For Fig 3I, the authors can then conduct a regression analysis to assess gene length dependency of expression differences. For Figure 3K, the authors should report log2 fold changes and the adjusted p-values from DEseq2 instead of normalizing to one condition and conducting an ANOVA test.
16. The 3M vs 20M plot in Fig. 3I does not support the conclusion that long genes exhibit decreased expression in 20M mice, since the coding sequence length between up- and down-regulated genes is similar and the variability is larger in the down-regulated genes. Most long genes are in the 'other' category (which should be defined in the legends), and the actual number of genes with differential expression is small. It is also not clear what qualifies as "long genes." The authors preface this figure by citing papers that have previously looked at transcriptomic changes in long genes over aging, but since the subsequent focus of the manuscript is on AIS genes and function, it would be more interesting and cohesive to conduct more (and properly conducted) in-depth analysis of AIS genes and other neuronal activity related genes rather than looking at these "long genes."
17. Are the changes in gene expression in 20M vs 3M comparison specific to AIS genes, or also observed in other gene groups? Similarly, are AIS genes most significantly restored in 20M+Sun1 mice compared to other families of genes? The logic of specifically looking at AIS genes is difficult to follow, especially because the GO analysis shown in EV9B indicate major differences in mitochondrial-related processes rather than AIS.
18. Fig EV9C: The legend needs to be more descriptive. The current legend says this heatmap shows DEGs, but it's not clear what groups are actually being compared. Further, the lines on the top of the plot are not explained, nor are the GO terms that groups of genes belong to.

19. While Sun1 re-expression in aged neurons restores AIS length, ion channel intensities, and neuronal activity, it is not clear how directly Sun1 contributes to the observed abnormalities in AIS structure and neuronal excitability. The authors suggest the aging-related defects are due to Sun1- dependent transcriptomic changes. However, could there be other mechanisms downstream of Sun1 expression that are more directly responsible? For example, nuclear-cytoskeleton communication or mechanotransduction via the LINC complex may be diminished in older neurons but could be rescued with Sun1 re-expression, and may be more relevant to the actin rings of the AIS.
20. What is the link between the observed lack of neuronal excitability and the differences in mouse behavioral test? It is unclear why these tests were specifically chosen and whether the observed behaviors are expected from the neuronal excitability data.

Referee #3:

This is a very interesting paper by Hasegawa et al which makes the observation that aged mice lose expression of LINC complex proteins in neurons. The observation that the LINC complex proteins decrease over time (aging) is a new and novel observation that merits publication. Additionally, the authors show that overexpression of a LINC complex protein (SUN1) reverses a number of aging-related changes with regards to LINC complex formation, neuron nuclear morphology, and neuron excitability.
The paper is quite extensive, and in some aspects overwhelming given the large number of extended figures. This is just a general comment, as I am unsure which data could be reduced or consolidated.
I have a few concerns I am hoping the authors can address with some additional discussion.
1. There are some studies already implicating LINC complex changes in neurodegenerative diseases, such as ALS and Alzheimer's. Can the authors put their results in this context in the discussion?
2. Can the authors better discuss the mouse behavior tests and what specific aspects of neurological function they are assessing? There are other memory tests (e.g. Morris Water Maze) that were not used, can the authors be clearer on why the specific behavior tests were chosen?
3. Are the authors trying to claim that SUN1 is more important than SUN2? Is this correct, or could have SUN2 over expression also rescued, preventing the aging-associated changes? Did the authors try rescue with over expression of other LINC complex proteins (mini-nesprin or SUN2)? I am not expecting the authors to go back and do these experiments (if they didn't already), but it would be helpful to discuss if it is possible that over expression of other LINC complex proteins could rescue.
4. Are the authors able to speculate why is SUN1 expression enough to rescue the loss of other LINC complex proteins? Is there anything known about the role of SUN1 in the formation or stabilization of the LINC complex?
5. The authors show that LINC-DN did not lead to cell death over a 1.5 month time interval. Did the authors look at longer time points? Is there neuron loss in control aged mice (without LINC-DN)? Are there any markers of neurodegeneration that are present in aged mice or lost with SUN1 rescue?
6. Did the authors assess the overall neuron organization (number, morphology, positioning) in the brain over time? The images are mostly of only a few cells-are there any obvious histological changes in the brain in how neurons or other supporting cells are organized?
7. Can the authors comment if they think that SUN1 overexpression is changing primarily mechanics or gene expression (or perhaps these two things are too interconnected to know what is the major regulator of the LINC complex)?

Responses to Reviewers

Responses to reviewer 2

General comments

1) The authors should improve the clarity of the manuscript by clearly stating the experimental design, including the timeline of AAV expression and harvesting/analysis, for all experiments when they first introduce them.

<u>**Response**</u>

To help understanding of the AAV experiment designs, we have added schematics (EV4F, EV7A, EV8A, EV14F) detailing the timing of administration and analysis, as well as AAV types. In this study, we conducted the following four AAV experiments.

1. **Experiments to examine the effects of dysfunction of the LINC complex using 3-month-old mice infected with AAV-LINC-DN (or AAV-Venus).**

< EV4F >

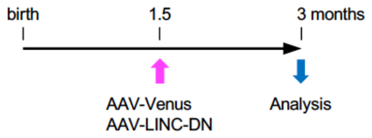

2. **Experiments to examine the effects of Sun1 introduction on age-related changes in tissue, neuronal activity, and behavior using 20-month-old mice infected with AAV-Sun1 (or AAV-Venus, or AAV-AnkG KO).**

< EV7A >

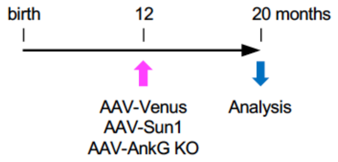

3. **Experiments for genome analyses using 3- or 20-month-old mice infected with AAV-eGFP-Cre (with/without AAV-Sun1) to enable isolation of neuronal nuclei.**

< EV8A >

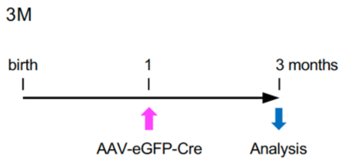

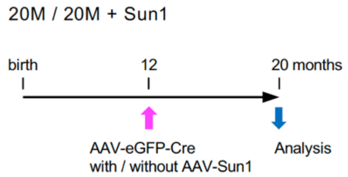

4. Experiments to examine the effects of Sun1 introduction on age-related changes in tissue, neuronal activity, and behavior using 23-month-old mice infected with AAV-Sun1 (or AAV-Venus).

< EV14F >

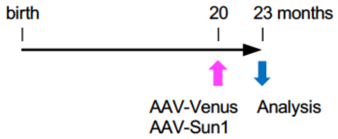

2) The methods lack details on exactly how some of the analysis was conducted. The authors should ensure that each analysis has a clear description that includes exactly what was measured, such that readers can clearly understand how each plot was generated. As an example, in the "Analysis of AIS" section, the authors state "signal intensities were measured using ImageJ 1.53. " However, this does not indicate whether the mean intensities or the total intensity in the entire traced area was measured. It is also unclear from the methods whether the same analysis was conducted for the ion channel intensity measurements as for AIS intensities.

Response

In response to the reviewer's comments, we have added detailed descriptions of the AIS and the nuclear analyses in the Methods.

"Analysis of AIS" (page 30, line 21 to page 31, line 8)

Original: … For spatial molecular profiling, immunoreactive signals of target proteins in the AIS were traced with a line that accurately followed AIS structure using PenTablet software (XP-PEN Technology), and signal intensities were measured using ImageJ 1.53 software. …[ellipsis]… The resulting intensity profiles were exported to Excel (Microsoft) and analyzed using a custom MATLAB/Octave script (Berger *et al*, 2018) (provided by Dr. J.L. Salzer).

↓

Revised: … For spatial molecular profiling, immunoreactive signals of the AIS localized proteins were manually traced with a line that accurately followed AIS structure using PenTablet software (XP-PEN Technology), and signal intensities per 0.38-μm-length segments along the traced line were measured using ImageJ 1.53 software. Background intensity from regions lacking specific staining was subtracted from the values obtained in signal-positive regions. The resulting intensity profiles were exported to Excel (Microsoft), analyzed using a custom MATLAB/Octave script (Berger *et al*, 2018) (provided by Dr. J.L. Salzer) (Figs. 5H,K and EV12B,E,H). The total intensity of the AIS localized proteins was calculated by integrating the signal intensity of each segment along the traced line (Figs. 4B,D,F, 5I,L and EV12C,F,I). …

"Nuclear analysis" (page 32, line 21 to page 33, line 12)

Original: … Quantification of signal intensity on the NE was performed by

tracing a line that accurately followed the contour of the NE using PenTablet software, and the signal intensity along this line was measured using ImageJ 1.53 software. ….[ellipsis]…. For quantification of nuclear H3K9me3 signal, the NE was traced using PenTablet software to define the nuclear region, and signal intensity within this area was measured using ImageJ 1.53 software.

↓

Revised: … Quantification of signal intensity on the NE was performed by manual tracing a line that accurately followed the contour of the NE using PenTablet software, and the total signal intensity along this line was measured using ImageJ 1.53 software. ….[ellipsis]…. For quantification of nuclear H3K9me3 signal, the NE was manually traced using PenTablet software to define the nuclear region, and total signal intensity within this area was measured using ImageJ 1.53 software. ….[ellipsis]…. For the analysis of nuclear structure, nuclei in layer V pyramidal neurons of the prefrontal cortex, layer II/III pyramidal neurons of the somatosensory cortex, and primary cultured cortical neurons were visualized by Lamin B1 staining, manually inspected, and those with two or more distortions were classified as structurally abnormal.

3) When the authors mention "n", they should specify whether the number they indicate refer to animals, cells, AIS, nuclei, etc. For imaging experiments looking at intensity, AIS length, etc., it is best practice to treat n as each animal and conduct statistics on the mean value from each animal (see https://doi.org/10.1083/jcb.202001064). The authors should update their plots and statistical tests as to not overpower their analyses, and clarify how many animals were used in their experiments where they report n as individual cell or AIS.

Response

Throughout this study, we have carefully confirmed that, when measuring the intensity or length of all target molecules and structures, the patterns across animals were consistent within each group. Furthermore, we confirmed that the conclusions of each experiment were not affected by whether the data were analyzed at the cellular or animal level. Below, we present similarities in the results of each representative experiments concerning the expression levels of LINC complex and channel molecules, as well as AIS length. Based on these observations, we consider that statistical analyses at the cellular level are appropriate for this study; therefore we have adopted cellular-level rather than animal-based statistical analyses. Instead, to facilitate readers' understanding, we have clarified in the relevant figure legends what "n" represents, as well as the total number of mice or brains used. These clarifications have been incorporated into the legends for all figures (highlighted in yellow), except EV5, EV8, and EV9.

< Figure 1F: intensities of Nesprin-1 on the nuclear envelope >

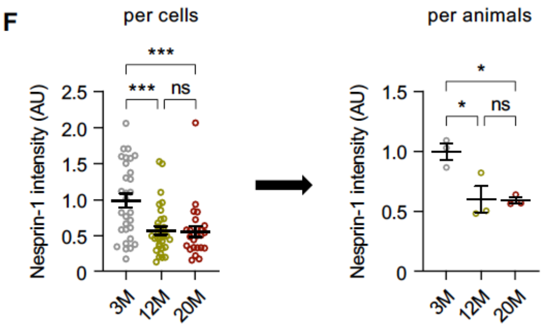

< Figure 4B: intensity of Nav 1.2 at the AIS >

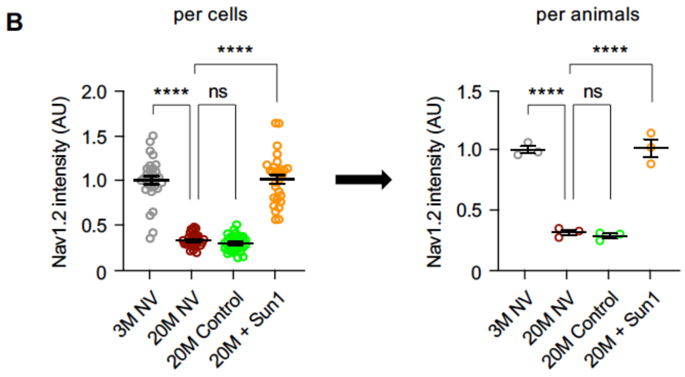

< Figure 4H: AIS length >

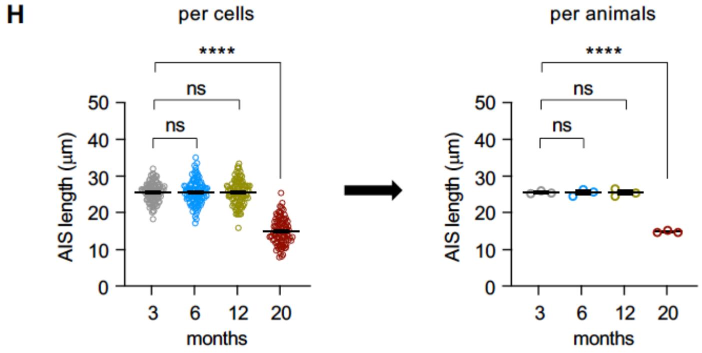

4) For gene expression analyses, the authors should use DESeq2 (or EdgeR) analysis to report adjusted p-values and log2 fold changes, since expression of each gene is not an independent observation and RNA-seq data are not normally distributed.

Response

We appreciate the reviewer's valuable comment. We would like to clarify that differential expression analysis in this study was already performed using DESeq2. In the original manuscript, adjusted p-values (padj) were reported together with p-values from the DESeq2 analysis (original: Source Data 5, revised: Figure EV9 Source Data EV9A,B).

Importantly, gene selection was based on log2 fold change and p-values obtained from the DESeq2 analysis, without applying a strict padj-based cutoff, because the primary objective of this analysis is not to compile a definitive catalog of differentially expressed genes, but rather to examine expression trends and functional patterns relevant to the biological process under investigation. In this context, applying a conventional padj < 0.05 threshold would substantially reduce the number of genes available for downstream analyses and potentially obscure biologically meaningful expression changes. Consistent with this rationale, key genes selected based on log2 fold_change and p-values were independently validated by qPCR. As shown in new Fig. 3J, these genes exhibited statistically significant expression differences, providing experimental support for the biological relevance of our gene selection strategy.

Specific comments

5) On Page 6 the authors write "These results suggest that aging induces neuron-specific changes in the expression of LINC complex components." However, EV3 shows that Sun2 is down in glial cells in 20 months as well, indicating that the changes to LINC complex components are not only in neurons.

Response

According to the reviewer's comment, we have revised the relevant sentence as follows. (page 6, line 8 to line10)

Original: These results suggest that aging induces neuron-specific changes in the expression of LINC complex components.

↓

Revised: These results suggest that aging induces cell type-specific changes in the expression of LINC complex components in neuronal lineage cells, characterized by a coordinated reduction of all major components in neurons.

6) In Fig 3 and Fig EV 8, instead of plotting the entire p(s) curve for contact frequency, plotting the derivative of the curve would be more helpful in visualizing the fine-scale changes between conditions.

Response

In response to the reviewer's constructive advice, we have revised the derivative plots of the curves in Figs. 3E and EV8C, as follows.

< Figure 3E >

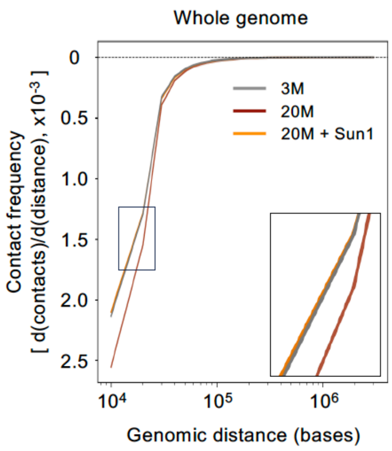

< Figure EV8C >

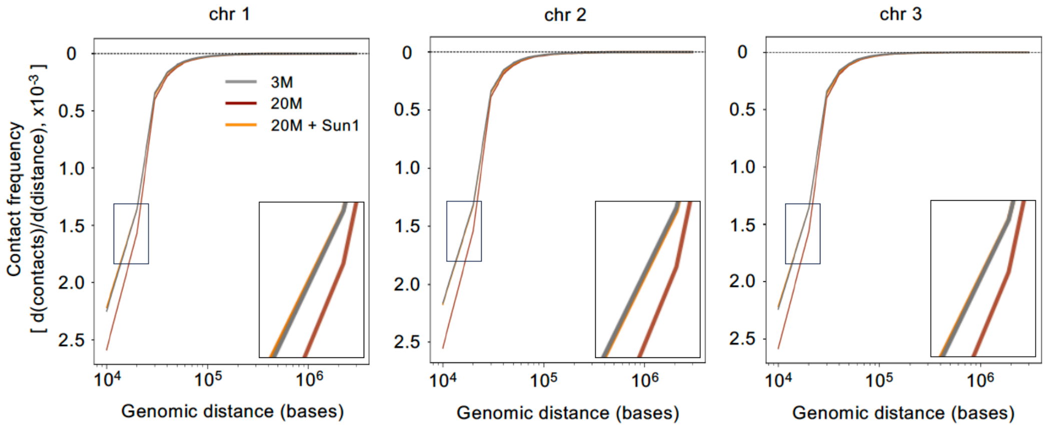

7) The definition of A / B compartments on page 10 should not include information on nuclear positioning, as it is not always true that heterochromatin or silenced genes are at the periphery while active genes are more towards the inner nucleus.

Response

Based on the reviewer's suggestion, we have deleted the information

regarding the nuclear positioning of the A/B compartments, as follows. (page 9, line 21 to page 10, line 3)

Original: … into compartment A, which are genomic regions with active histone modifications at the inner nuclear space, and exhibited an increase in the number of genes classified into compartment B, which are genomic regions with repressive histone modifications at the NE periphery, compared to…

↓

Revised: … into compartment A, which are genomic regions with active histone modifications, and exhibited an increase in the number of genes classified into compartment B, which are genomic regions with repressive histone modifications, compared to…

8) In Fig 3F, the distribution of genes in compartment A vs B from 20M+Sun1 mice appear more similar to that of 20M than 3M, and does not really support the authors' conclusion "in the neurons of 20M+Sun1 mice, the distribution of genes in compartments A and B shifted toward the pattern observed in young neurons" is not well supported.

Response

In accordance with the reviewer's comment, we revised the explanation of the compartment A/B shift to a more modest description that reflects the actual changes, as follows. (page 10, line 3 to line 5)

Original: However, in the neurons of 20M+Sun1 mice, the distribution of genes in compartments A and B shifted toward the pattern observed in young neurons (Fig. 3F).

↓

Revised: However, in the neurons of 20M+Sun1 mice, the distribution of genes in compartments A and B showed a partial shift toward the pattern observed in young neurons (Fig. 3F).

9) The % of genes with A/B compartment shifts (Fig. 3G, Fig. EV8C) are not very informative. ChIP-seq studies (for example, H3K9me3 to follow up on Fig 3A, B) to show changes in histone modifications at specific genes including the AIS genes would be much more informative.

Response

We agree with the reviewer's point and plan to conduct comprehensive genomic analyses using ChIP-seq and ATAC-seq to investigate histone modifications and transcriptional regulation. Specifically, we will focus on age-related changes in the expression regulation of AIS-related genes, including those encoding Nav and Kv identified in this study, and the effects of Sun1 introduction on these changes.

Considering the balance with the principal claims established through diverse analyses, such as cell biology, histology, electrophysiology, and

behavioral analyses in this study, we intend to present further genomic analyses mentioned above as a separate paper particularly focusing on gene expression regulation.

10) The authors should be cautious in directly linking Sun1 to the genomics changes they observe. They show that LaminB intensity is increased in 20M+Sun1 neurons in Fig 2L, and as LaminB is associated with genomic regions (lamina-associated domains), the increased intensity of LaminB may be more directly causing gene expression changes than Sun1. Because the mechanism of how Sun1 reexpression rescues or prevents the nuclear defects of 20M mice is unknown, the authors should adequately discuss the possibility the indirect effects due to Sun1 re-expression.

Response

We do not claim that Sun1 directly induces changes in the genome. As the reviewer pointed out, the Sun1 reexpression-induced alterations in chromatin dynamics and nuclear structure in aged neurons may directly involve Lamin B1. We have now addressed this possibility in the Discussion, as follows. (page 18, line 13 to line 16)

New description: It is noteworthy that the pronounced restoration of Lamin B1, which directly associates with the genome, following Sun1 introduction in aged neurons suggests that the resulting change in Lamin B1 level may impact chromatin dynamics.

11) Fig. 3H: It is unclear whether a correlation of 0.94 is meaningfully different from correlation of 1. Volcano plots that show the log fold change in gene expression and clearly reporting how many genes are significantly differentially regulated in all pairwise comparisons would be a much more informative way to reflect the differences.

Response

We appreciate the reviewer's important comments. Correlation coefficients close to 1 should be interpreted cautiously, and a correlation of 0.94 is not meant to be statistically different from 1. In this study, correlation analysis was used to describe overall gene expression similarity rather than as a statistical test. Biological replicates under the same experimental condition showed very high correlations ($r > 0.99$), whereas correlations between 3M and 20M samples were lower ($r \approx 0.94$), reflecting widespread expression differences. Notably, the correlation between 20M + Sun1 and 3M increased to ~0.98, indicating substantial restoration toward a young-like state. We interpret these differences in correlation coefficients as reflecting biologically meaningful changes in overall gene expression similarity, not precise deviations from perfect correlation. In addition, we consider that a heatmap, rather than volcano plots, is more appropriate for visually showing overall gene expression similarity across three groups.

12) Do the authors have an explanation for why most of the genes in Fig. EV9B (20M vs 20M+Sun1 comparison) are mitochondrial genes, and not nuclear processes related to Sun1?

<u>Response</u>

It is difficult to provide a definitive answer to the reviewer's question. The high frequency of changes in mitochondria-related genes may in part reflect their relative susceptibility to chromatin structural alterations induced by Sun1 introduction, or alternatively, they may represent secondary effects arising from alterations in various cellular functions via distinct mechanisms. In addition, since there is no evidence that Sun1 introduction necessarily affects the transcriptional regulation of genes related to nuclear processes, we can appreciate why these genes were not identified in the GO analysis.

13) In the methods, the authors state having used 1000 cells total for the RNA-seq experiments. This seem extremely low, even accounting for the sorting step, and may not be sufficient to accurately reflect the gene expression profile of neurons in the entire cerebrum. Is there a reason the authors have used such a low number of input cells?

<u>Response</u>

Because isolating intact nuclei of sufficient quality for expression analysis from fragile *in vivo* neurons is technically challenging, we employed a kit that has been validated in previous studies (ref. 1, 2). The protocol for this kit (SMART-seq kit, Takara Bio) recommends using 1,000 cells for cDNA synthesis, and we adhered to this guideline.

Our perspective regarding the accuracy of the neuronal profile within the cerebral cortex in this study is as follows. The cerebral cortex is composed of approximately ten principal neuronal cell types, which transcriptomic analyses further refine into slightly over one hundred distinct subtypes (ref. 3). Considering the proportion of neuronal subtypes with low abundance (ref. 3), the use of 1,000 cells in this study is deemed sufficient to represent most neuronal subtypes present in the cerebral cortex. Indeed, prior studies have conducted bulk expression analyses of mouse cortical neurons using as few as 100 to 200 cells (ref. 1), or an equivalent amount of cellular material (ref. 2). Hence, we assert that this dataset provides an accurate and representative characterization of the neuronal composition of the cerebral cortex.

<u>References for the reviewer</u>

1. **Apelblat D et al., Meso-seq for in-depth transcriptomics in ultra-low amounts of FACS-purified neuronal nuclei.** ***Cell Rep Methods*** **2: 100259 (2022).**
2. **Amamoto R et al., FIN-Seq: transcriptional profiling of specific cell types from frozen archived tissue of the human central nervous system.** ***Nucleic Acids Res*** **48: e4 (2020).**
3. **Mao X and Staiger JF, Multimodel cortical neuronal cell type classification.**

Pflugers Arch **476: 721-733 (2024).**

14) Fig. 3J, the colorbar that goes from -2 to 2 needs to have a legend. Is this fold change or log(FPKM)?

Response

In Fig. 3J (new Fig. 3I), the colorbar represents the Z-score (FPKM). According to the reviewer's comment, we have added a more detailed explanation, as follows. (page 47, line 24 to page 48, line 3)

Original: (**J**) Heatmap of selected genes encoding AIS-related molecules including Nav and Kv channels. The color scale represents fragments per kilobase of exon per million mapped reads (FPKM) across groups, scaled row-wise.

↓

Revised: (**I**) Heatmap of selected genes encoding AIS-related molecules including Nav and Kv channels. DEGs were defined as those with |log2(fold_change)| >1, *P*<0.001. Fragments per kilobase of exon per million mapped reads (FPKM) values were subjected to gene-wise z-score normalization before generating the heatmap.

15) For RNA-seq data, the authors should not use statistical test that assume equal variance and/or normal distribution on normalized data. Rather, they should use standard differential gene expression analysis pipelines (such as DEseq2, which they describe using in the methods) to obtain log2 fold change for each gene. For Fig 3I, the authors can then conduct a regression analysis to assess gene length dependency of expression differences. For Figure 3K, the authors should report log2 fold changes and the adjusted p-values from DEseq2 instead of normalizing to one condition and conducting an ANOVA test.

Response

In accordance with the reviewer's comment 16, we have withdrawn original Fig. 3I and therefore did not perform a regression analysis to assess gene length–dependent expression differences. With regard to Fig. 3K (new Fig. 3J), these analyses were performed using qPCR targeting specific genes and were not derived from RNA-seq data. Performing ANOVA on mRNA levels normalized to a specific condition is a standard statistical procedure in qPCR-based gene expression analysis.

16) The 3M vs 20M plot in Fig. 3I does not support the conclusion that long genes exhibit decreased expression in 20M mice, since the coding sequence length between up- and down-regulated genes is similar and the variability is larger in the down-regulated genes. Most long genes are in the 'other' category (which should be defined in the legends), and the actual number of genes with differential expression is small. It is also not clear what qualifies as "long genes." The authors preface this figure by citing papers that have

previously looked at transcriptomic changes in long genes over aging, but since the subsequent focus of the manuscript is on AIS genes and function, it would be more interesting and cohesive to conduct more (and properly conducted) in-depth analysis of AIS genes and other neuronal activity related genes rather than looking at these "long genes."

Response

In response to the reviewer's comments, we have withdrawn Fig. 3I. As shown in new Fig. 3J, based on RNA-seq data, we further validated AIS-related gene expression, including ion channel genes, by qPCR. As noted in our response to Reviewer 2 comment 9, our future work will involve in-depth analyses, such as ChIP-seq and ATAC-seq, to investigate epigenetic modifications (e.g., DNA methylation and histone modifications) and other transcriptional regulatory mechanisms of these genes.

17) Are the changes in gene expression in 20M vs 3M comparison specific to AIS genes, or also observed in other gene groups? Similarly, are AIS genes most significantly restored in 20M+Sun1 mice compared to other families of genes? The logic of specifically looking at AIS genes is difficult to follow, especially because the GO analysis shown in EV9B indicate major differences in mitochondrial-related processes rather than AIS.

Response

As shown in EV9A, GO analysis comparing 3M and 20M revealed changes across multiple gene groups. Genes in the GO terms "Identical protein binding", "Protein-containing complex binding", and "Structural constituent of synapse" were altered in both the 3M vs 20M and 20M vs 20M+Sun1 comparisons (EV9A,B), with some subsets shifting markedly toward young-neuron expression patterns upon Sun1 introduction (EV9C). However, these datasets offered limited insight into the mechanisms of neuronal aging. Mitochondrial genes, well-known for their role in cellular senescence across various cell types, were scarcely detected in the 3M vs 20M comparison.

Then, independently of the GO analysis, we examined the expression of individual gene groups and identified a subset of key AIS-related genes that exhibited highly intriguing expression changes associated with aging and Sun1 introduction. Although age-related changes in neuronal excitability have been reported, their significance in brain aging remained unclear, which prompted us to focus on the AIS to elucidate the molecular mechanisms underlying neuronal aging.

18) Fig EV9C: The legend needs to be more descriptive. The current legend says this heatmap shows DEGs, but it's not clear what groups are actually being compared. Further, the lines on the top of the plot are not explained, nor are the GO terms that groups of genes belong to.

Response

According to the reviewer's comments, we have added a more detailed explanation to the legend of EV9. (page 58, line 12 to line 19)

Original: (**A**, **B**) GO analysis for AAV-eGFP-Cre alone (3M or 20M) and AAV-eGFP-Cre + AAV-Sun1 (20M + Sun1) groups. (**C**), Heatmap of selected and clustered DEGs associated with common GO terms shown in (**A**, **B**, highlighted in red). Each group consists of three mice. DEGs were defined as those with |log2(fold_change)| >1, *P*<0.001. The color scale represents FPKM across groups, scaled row-wise.

↓

Revised: (**A**, **B**) GO analysis for AAV-eGFP-Cre alone (3M or 20M) and AAV-eGFP-Cre + AAV-Sun1 (20M + Sun1) groups. The three GO terms highlighted in red showed significant expression changes in both 3M *vs* 20M (**A**) and 20M *vs* 20M + Sun1 (**B**). (**C**), Heatmap of clustered DEGs associated with GO terms highlighted in red in **A** and **B**. The line above the heatmap indicates the classification of expression similarity of the 3M and 20M + Sun1 groups relative to the 20M group. Each group consists of three mice. DEGs were defined as those with |log2(fold_change)| >1, *P*<0.001. FPKM values were subjected to gene-wise z-score normalization before generating the heatmap.

19) While Sun1 re-expression in aged neurons restores AIS length, ion channel intensities, and neuronal activity, it is not clear how directly Sun1 contributes to the observed abnormalities in AIS structure and neuronal excitability. The authors suggest the aging-related defects are due to Sun1- dependent transcriptomic changes. However, could there be other mechanisms downstream of Sun1 expression that are more directly responsible? For example, nuclear-cytoskeleton communication or mechanotransduction via the LINC complex may be diminished in older neurons but could be rescued with Sun1 re-expression, and may be more relevant to the actin rings of the AIS.

Response

As described in the Introduction, Nav and Kv expression determines action potential generation at the AIS, which exhibits activity-dependent structural plasticity. Specifically, previous studies have shown that reduced Nav expression and increased Kv expression impair action potential generation at the AIS, thereby decreasing neuronal excitability. It is also well established that prolonged reduction in neuronal activity leads to AIS shortening. Therefore, if the loss of LINC complex function in aged neurons alters the expression of AIS-associated voltage-gated channels, substantial effects on both neuronal excitability and AIS structure would be expected.

On the other hand, as the reviewer noted, age-related LINC complex dysfunction may affect not only the nucleus but also the cytoskeleton. However, how such abnormalities might directly influence the distant AIS remains unclear. Investigating cytoplasmic changes in aged neurons—particularly in the actin/microtubule networks and AIS-related intracellular transport—and assessing how Sun1 introduction modulates these processes could provide valuable mechanistic insights.

20) What is the link between the observed lack of neuronal excitability and the differences in mouse behavioral test? It is unclear why these tests were specifically chosen and whether the observed behaviors are expected from the neuronal excitability data.

<u>Response</u>

Understanding how brain functions such as emotion and memory—reflected in behavioral outputs of coordinated neural circuits—relate to the excitability of individual neurons remains a major challenge. We believe that this study is of great significance, as it provides, at least in part, the first evidence of a causal link between age-related decline in neuronal excitability and alterations in brain function through Sun1 introduction into aged neurons.

In this study, as described in the original manuscript (page 16, line 15 to line 17), we first decided to analyze spontaneous locomotor activity, anxiety, and memory as the age-related changes in brain function with the most consensus based on previous studies. Next, we investigated which specific experimental methods had been used to support these age-associated changes through a review of over 20 papers. Consequently, we employed the open-field test, which was the leading method for evaluating spontaneous locomotor activity and anxiety. For memory assessment, we selected the novel object recognition test, identified as the most frequently applied in the reviewed studies. In addition, the elevated plus maze test, the second most commonly used for anxiety assessment, was also included in our study. We applied the same tests for behavioral analyses in young mice.

Responses to reviewer 3

1) There are some studies already implicating LINC complex changes in neurodegenerative diseases, such as ALS and Alzheimer's. Can the authors put their results in this context in the discussion?

<u>Response</u>

Although several mutations in Nesprin-1 have been identified in patients with ALS (ref. 1, 2), the impact of these mutations on pathogenesis remains unclear. In neurons harboring the ALS-associated C9ORF72 mutation, mislocalization of LINC complex components has been observed, but it is still uncertain whether this change contributes to neuronal death (ref. 3). Furthermore, Sun1 may enhance chromatin modification protein 7-mediated toxicity in neurons differentiated from ALS patient-derived iPS cells; however, it does not play a definitive role in triggering neuronal death (ref. 4). On the other hand, to the best of our knowledge, no studies have yet demonstrated the involvement of the LINC complex in Alzheimer's disease.

Given this situation, we consider that there is still insufficient evidence to clearly demonstrate the contribution of the LINC complex in neurodegenerative

diseases, which is necessary to facilitate a deep comparative discussion of its role in normal neuronal aging and neurodegenerative diseases.

References for the reviewer

1. **Ozoguz et al., The distinct genetic pattern of ALS in Turkey and novel mutations. *Neurobiol. Aging* 36, 1764.e9–1764.e18 (2015).**
2. **Naruse H et al., Juvenile amyotrophic lateral sclerosis with complex phenotypes associated with novel SYNE1 mutations. *Amyotroph. Lateral Scler. Front. Degener.* 22, 576–578 (2021).**
3. **Sirtori R et al., LINC complex alterations are a key feature of sporadic and familial ALS/FTD. *Acta Neuropathol. Commun.* 12, 69 (2024).**
4. **Baskerville V et al., SUN1 facilitates CHMP7 nuclear influx and injury cascades in sporadic amyotrophic lateral sclerosis. *Brain* 147, 109–121 (2024).**

2) Can the authors better discuss the mouse behavior tests and what specific aspects of neurological function they are assessing? There are other memory tests (e.g. Morris Water Maze) that were not used, can the authors be clearer on why the specific behavior tests were chosen?

Response

In accordance with the reviewer's comments, we have added brief discussions and additional information on the specific behavioral measures assessed in each analysis, as follows. (page 14, line 23 to page 15, line 10)

Original: In the open-field test, LINC-DN-expressing mice exhibited altered anxiety-like behaviors associated with open spaces and increased locomotor activity compared to the control groups (Figs. 6F,G). These anxiety-like phenotypes were confirmed using the elevated plus maze test (Fig. 6H). In contrast, the novel object recognition test revealed that memory function remained intact in these mice (Fig. 6I). These results suggest that the LINC complex is essential for maintaining specific brain functions, likely through AIS-dependent regulation of neural activity.

↓

Revised: In the open-field test, LINC-DN-expressing mice exhibited altered anxiety-like behaviors <u>associated with open spaces</u> and increased <u>spontaneous</u> locomotor activity compared to the control groups (Figs. 6F,G and EV4F). <u>Anxiety-like phenotype was confirmed using the elevated plus maze test (Fig. 6H), which assesses acrophobia-related anxiety</u>. In contrast, the novel object recognition test revealed that <u>cognitive</u> memory function remained intact in these mice (Fig. 6I). These results suggest that the LINC complex is essential for maintaining specific brain functions, likely through AIS-dependent regulation of neural activity. <u>Notably, anxiety-related emotions were significantly affected by reduced neural activity regardless of their underlying causes, whereas cognitive memory was unaffected, highlighting the heterogeneity in the mechanisms generating distinct brain functions.</u>

(page 16, line 17 to line 20)

New description:

In young and aged mice, memory function exhibits differential responses to LINC complex integrity and the neural activity it governs (Figs. 6I and EV14N), suggesting the involvement of additional factors whose effects vary with age in the regulation of this process.

The following explains our rationale for selecting the specific behavioral tests. As described in the original manuscript (page 16, line 15 to line 17), we first decided to analyze spontaneous locomotor activity, anxiety, and memory as the age-related changes in brain function with the most consensus based on previous studies. Next, we investigated which specific experimental methods had been used to support these age-associated changes. As a result, spontaneous locomotor activity was overwhelmingly assessed by the open-field test, while anxiety was most commonly evaluated by the open-field test and the elevated plus maze test, which were the top two methods in 17 and 11 out of 20 papers we investigated, respectively. In contrast, various methods exist for memory assessment, with the novel object recognition test being the most frequently used in 19 out of 32 papers we investigated. The Morris water maze test had fewer reports demonstrating age-associated changes.

For this reason, in this study, we selected the open-field test, the elevated plus maze test, and the novel object recognition test for behavioral experiments in aged mice, and applied the same tests for behavioral analyses in young mice.

3) Are the authors trying to claim that SUN1 is more important than SUN2? Is this correct, or could have SUN2 over expression also rescued, preventing the aging-associated changes? Did the authors try rescue with over expression of other LINC complex proteins (mini-nesprin or SUN2)? I am not expecting the authors to go back and do these experiments (if they didn't already), but it would be helpful to discuss if it is possible that over expression of other LINC complex proteins could rescue.

Response

As the reviewer pointed, we believe that Sun1 is the most important LINC complex component in preventing age-associated changes in the brain, since the introduction of Sun1 alone into aged cortical neurons can restore the expression levels of Nesprin-1/-2 on the nuclear envelope (NE) to those comparable to young neurons (EV7 D-G), thereby recovering the function of the LINC complex.

On the other hand, Sun2 does not appear to be a key molecule for anchoring Nesprins to the NE in cortical neurons (EV6), perhaps due to differential relative expression levels of Sun1 and Sun2 in this neuronal type, even though supra-physiological overexpression of Sun2 in aged neurons may potentially ameliorate age-associated changes in a manner similarly to Sun1. Regarding mini-Nesprin, the marked decline in endogenous Sun1 and Sun2 expression in aged neurons may compromise stable expression of mini-Nesprin on the NE,

thereby limiting its functional impact; consequently, further validation experiments were not conducted.

Based on the aforementioned reasons, LINC complex components other than Sun1 appear to have limited efficacy in preventing age-associated changes. Therefore, we consider that discussing these components does not provide significant implications for future research, and consequently, we have refrained from including such content in the Discussion section.

4) Are the authors able to speculate why is SUN1 expression enough to rescue the loss of other LINC complex proteins? Is there anything known about the role of SUN1 in the formation or stabilization of the LINC complex?

Response

Sun1 and Sun2 are well established to function complementarily in Nesprin binding and in stabilizing the LINC complex across diverse cell types. For example, in muscle cells, Nesprin-1 fails to localize to the NE upon simultaneous deletion of Sun1 and Sun2 (ref. 1). In aged neurons, where expression of both Sun1 and Sun2 is markedly reduced, the efficacy of exogenous Sun1 alone may reflect the relative Sun1-to-Sun2 expression ratio in cortical neurons, as noted in our response to the Reviewer 3, comment 3. Consistently, Sun1 deletion in cortical neurons leads to the loss of Nesprins from the NE in this neuron, whereas Sun2 deletion has no effect. To test this hypothesis, we plan to compare the expression levels of Sun1 and Sun2 in cortical neurons.

Reference for the reviewer

1. **Lei K et al., SUN1 and SUN2 play critical but partially redundant roles in anchoring nuclei in skeletal muscle cells in mice.** ***Proc Natl Acad Sci U S A.*** **106, 10207-12 (2009).**

5) The authors show that LINC-DN did not lead to cell death over a 1.5 month time interval. Did the authors look at longer time points? Is there neuron loss in control aged mice (without LINC-DN)? Are there any markers of neurodegeneration that are present in aged mice or lost with SUN1 rescue?

Response

In accordance with the reviewer's comment, we analyzed neurons with pyknotic nuclei using Hoechst staining and degenerating neurons using Fluoro-Jade C in samples with long-term expression of LINC-DN (extended to 3 months, twice the duration of the original data). Even with long-term LINC-DN expression, no induction of neuronal cell death was observed (Reviewer's Figure 1A–C).

In addition, analysis using Fluoro-Jade C showed no prominent neuronal cell death in aged mice (20M) compared to young mice (3M), and AAV-Sun1 expression in aged mice had no observable effect (Reviewer's Figure 1D). Therefore, as noted in the Introduction, neuronal cell death is unlikely to be a

major contributor to brain functional decline associated with physiological aging.

< Reviewer’s Figure 1 >

Effect of LINC-DN expression on Neuronal Cell Death

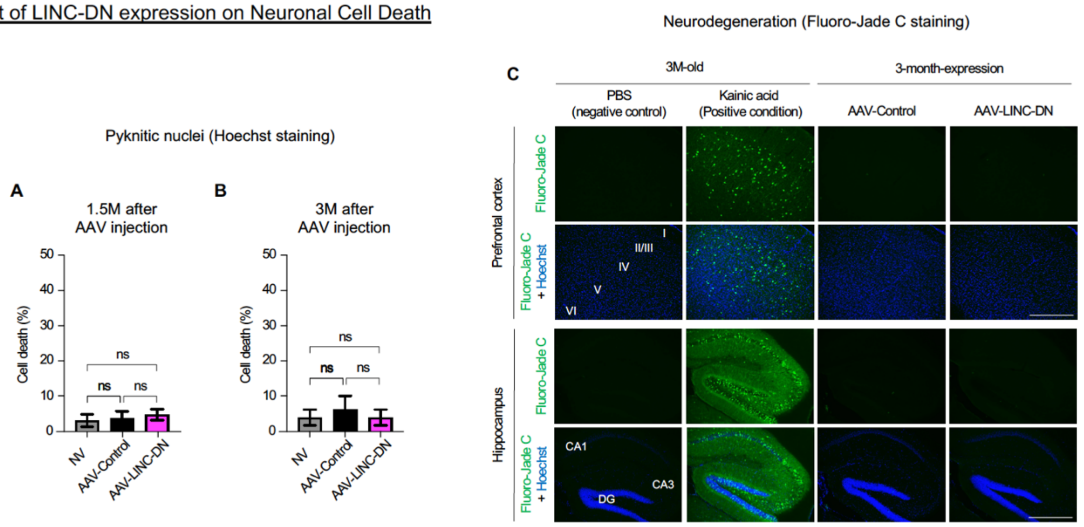

Effect of aging on Neuronal Cell Death

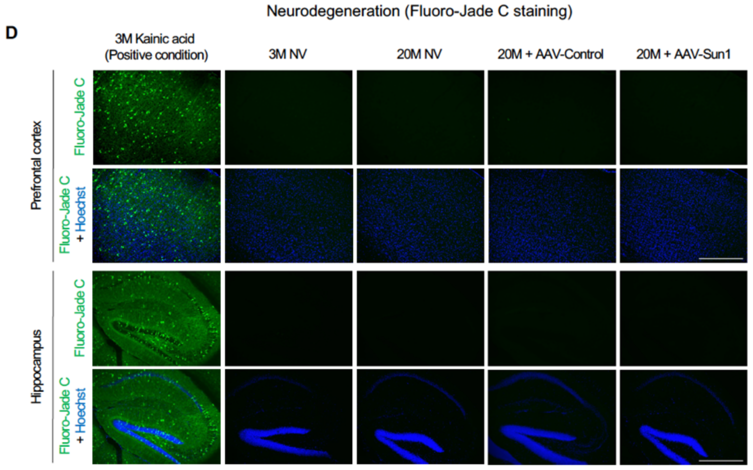

(A,B) Quantification of percentage of layer V pyramidal neurons with pyknotic nuclei in the prefrontal cortex of 3-month-old mice in no virus (NV), AAV-Venus (AAV-Control), and AAV-LINC-DN groups at 1.5 (A) or 3 (B) months after AAV injection. The data represent the mean ± SEM. n=4, 13-21 cells per brain (A), n=3, 15 cells per brain (B). ns, not significant (ordinary one-way ANOVA Tukey’s multiple comparison test). (C,D) Analysis of neuronal cell death. Brain sections from 3-month-old mice in AAV-Venus (AAV-Control) and AAV-LINC-DN groups in (C) or 3- or 20-month-old mice in no virus (3M NV or 20M NV), AAV-Venus (20M + AAV-Control), and AAV-Sun1 (20M + AAV-Sun1) groups in (D) were stained with the marker for neuronal cell death Fluoro-Jade C. Representative images of the prefrontal cortex (top two panels) and hippocampus (bottom two panels) of each group (C,D). As a positive control for neuronal cell death, 20 mg/kg (body weight) of kainic acid was administered intraperitoneally to 3-month-old C57BL/6J mice, and brains were fixed after 72 hours (second panels from the left in C and first panels from the left in D) . Phosphate-buffered saline (PBS) intraperitoneal injection was used as the negative control (first panels from the left in C). I – VI in (C) indicate the six layers of the prefrontal cortex. CA1, cornu ammonis 1; CA3, cornu ammonis 3; DG, dentate gyrus. Scale bars, 500 μm.

6) Did the authors assess the overall neuron organization (number, morphology, positioning) in the brain over time? The images are mostly of only a few cells-are there any obvious histological changes in the brain in how neurons or other supporting cells are organized?

Response

Although we did not quantitatively assess age-related changes in neuronal number, morphology, and spatial organization, nor evaluate the effects of Sun1 introduction on these parameters, we observed no overt changes in approximate number and gross structure of neurons during morphological analyses of the AIS and nucleus.

Nevertheless, given the involvement of the LINC complex in neuronal excitability and gene expression, we hypothesize that the LINC complex may also influence neuronal microstructures, such as dendritic spines, and the organization of neural circuit during brain aging. Therefore, we plan to conduct more detailed analyses of these aspects in a future publication.

7) Can the authors comment if they think that SUN1 overexpression is changing primarily mechanics or gene expression (or perhaps these two things are too interconnected to know what is the major regulator of the LINC complex)?

Response

Sun1 introduction is likely to restore LINC complex–mediated cytoskeleton–nucleus interactions, affecting both nuclear and cytoplasmic compartments. At least in the nucleus, Sun1 introduction clearly exerts substantial effects not only on nuclear structure but also on gene expression. Regarding the reduced neuronal excitability and AIS shortening in aged neurons, and their restoration following Sun1 introduction, we propose—as detailed in our response to Reviewer 2, comment 19—that alterations in Nav and Kv gene expression provide a compelling explanation.

In contrast, the effects on the cytoplasm, particularly on the actin and microtubule networks and AIS-related intracellular transport, remain entirely unknown. Future investigations into the mechanical impact on the cytoplasm are likely to provide important insights.

Taken together, we currently consider changes in gene expression, especially of AIS-associated molecules, to be the primary mechanism underlying the effects of Sun1 introduction. Nevertheless, we do not exclude other possibilities and will continue to explore the detailed mechanisms in future studies.

Dear Prof. Kuwako

Thank you for the submission of your revised manuscript to EMBO reports. We have now received the report from referee #2 who was asked to evaluate it (copied below).

As you will see, the referee is very positive about the study and requests only minor changes to clarify text and figures but also suggests removing the Hi-C dataset from the current paper. I would defer this decision to you. Please provide a point-by-point response to the remaining referee concerns.

From the editorial side, there are also a few things that we need before we can proceed with the official acceptance of your study.

- The Data Availability section needs to be placed before Acknowledgments.

- The Figure legends need to be placed after the References, at the very end of the manuscript.

- Regarding the Author Contributions, we now use CRediT to specify the contributions of each author in the journal submission system. CRediT replaces the author contribution section, which therefore needs to be removed from the manuscript text. You can use the free text box in our system if you wish to provide more detailed descriptions. See also guide to authors https://www.embopress.org/page/journal/14693178/authorguide#authorshipguidelines.

- Funding information: Shimane University Hospital Foundation for the Promotion of Advanced Medical Care might also need to be entered as a separate funder in our online manuscript tracking system. The information between the manuscript and the system must be congruent and it is the information from the system that will ultimately be transmitted to PubMed.

- I am afraid we cannot accomodate 14 EV figures. Could you please reduce the number of EV figures to 5-7 and add the remaining incl. their legends to the Appendix PDF? For the Appendix, the nomenclature is Appendix Figure S#.

- The legends and the descriptions of the Appendix tables need to be removed from the manuscript and provided in the Appendix file above each table (currently the table descriptions are missing from the Appendix).

- The "Supplementary Information" section needs to be removed from the manuscript.

- Please add callouts for Fig. 1RST.

- During our routine image integrity checks, we observed that the cell images within the figure set appear pixelated under analysis. This is often a result of converting original 16-bit TIFF files to RGB format for publication. While this is not inherently problematic, it can give the impression of image alteration to critical readers.
To address this, please upload the source data for the cell images in the Expanded View files.
This will enable us to confirm the integrity of the complete figure set and enhance transparency for readers.

Please address the following points in the figure legends
1) Please provide the exact p values in the legends of figures 1B, D, F, H; 2B, E, G, H, J, K, M, N; 3B, J; 4B, D, F, H, I, J, K; 5B, C, E, F, I, L Q; 6C, E, F, G, H; 7B, D, F, G, H; EV1 C, E, G, I, K, M, O, Q; EV2 A, B; EV3 A-D, E-H, J, N, Q, R, S, T; EV6 F, G; EV7 C, E, G, I, J; EV11 A, B, D, E, G, I, L, P; EV12 C, F, I; EV13 C, F, I, K, M; EV14 B ,D, H, J, L, M, N.
It is also possible to specify the exact p-values in the figure panel, if this is easier. The exact value is not required if $p<0.001$.
2) Please indicate the statistical test used for data analysis in the legends of figures EV9 A, B.

- Your response to the referees contains unpublished data in form of reviewer figures. We publish the referee reports and your response to these alongside the paper as Peer Review File. Please let us know in case you want to remove any of the reviewer figures. Please note that all data in the Peer Review File counts as published and that you would have to cite this document were your to re-use these in future publications.

- As a standard procedure we edit the title and abstract. Please find my suggestions with minor changes below my signature for your review.

- Finally, EMBO Reports papers are accompanied online by
A) a short (1-2 sentences) summary of the findings and their significance,
B) 2-3 bullet points highlighting key results and
C) a schematic summary figure that provides a sketch of the major findings (not a data image).
Please provide the summary figure as a separate file in PNG or JPG format at a size of 550x300-600 pixels (width x height).
Please note that the size is rather small and that text needs to be readable at the final size. Please send us this information

along with the revised manuscript.

We look forward to seeing a final version of your manuscript as soon as possible.

With kind regards,

Martina Rembold, PhD
Senior Editor
EMBO reports

==================

Referee #2:

The authors have addressed most of the reviewers' concerns from the original manuscript. This work is interesting and incorporates several different approaches to support a link between decline in LINC complex in aging neurons with AIS dysfunction. I have a few remaining comments:

• Regarding the new derivative p(s) plots in Figure 3E and Figure EV8C, these plots look strikingly different from published dP/ds plots (see PMID 41107486 Figure 1c for an example). Why are the curves so different?

• The Bioinformatics analysis in the Methods section should include more details regarding the z-score calculations, A/B compartment analysis, and contact frequency curve plotting.

• While the authors explain their rationale for reporting z-scores for differential gene analysis, it would still be informative to include a plot of log(2) fold change of all genes comparing 3M vs 20M vs 20M+Sun1 and report total number of genes upregulated vs downregulated between the conditions. Based on the rescue phenotype of 20M+Sun1, wouldn't it be expected that 3M vs 20M+Sun1 would have fewer DEGs compared to 3M vs 20M or 20M vs 20M+Sun1?

• The authors mentioned in their response to reviewers that they are working on a future manuscript containing ChIP-seq and ATAC-seq. Given this information, my recommendation is to consider incorporating the Hi-C data from this manuscript in the future manuscript instead. The contribution from the Hi-C data as it stands is that the overall genome architecture (at compartment scale) may shift towards that of younger neurons. However, as pointed out in my original review (point #8), this is not a strong result, and with more detailed and thorough analyses the authors may be able to gain more insights into the finer scale interactions that are altered. In addition, the paper is already very long with a lot of data and methods, and my view is that the main conclusions are not impacted in the absence of the 3D genomics data.

====================

The title should not exceed 100 characters incl spaces. What about:
Age-related LINC complex decline drives neuronal aging via axon initial segment dysfunction

Abstract
Brain aging is an intricate process that inevitably leads to functional deterioration. However, its molecular drivers remain unclear. Here, we show that the age-related decline in LINC complex expression on the neuronal nuclear envelope impairs axon initial segment (AIS)-mediated excitability and triggers brain aging. With aging, the expression of LINC complex components, including Sun1, decreases in various brain regions, accompanied by a reduction in AIS length. Preserving Sun1 expression rescues nuclear structural abnormalities in aged neurons, shifting chromatin dynamics and global gene expression toward those of young neurons. Particularly, it restores the expression of AIS-related molecules, including voltage-gated sodium or potassium channels essential for action potential generation. Inhibiting LINC complex in young mice impairs AIS integrity, leading to reduced neuronal excitability and brain dysfunction. Furthermore, Sun1 administration to aged neurons prevents age-related AIS shortening, excitability impairment, and brain function changes. Thus, we uncover the mechanism of normal brain aging involving AIS dysfunction, identifying the LINC complex component Sun1 as essential for preserving brain function.

Responses to Reviewer

Responses to reviewer 2

1) Regarding the new derivative p(s) plots in Figure 3E and Figure EV8C, these plots look strikingly different from published dP/ds plots (see PMID 41107486 Figure 1c for an example). Why are the curves so different?

<u>**Response**</u>

Thank you for the reference. In our analysis, we computed a simple derivative of *P*(s) using a custom script in linear space. In contrast, the published approach using Cooltools performs the derivative after log transformation, which accounts for the observed difference in curve shape. We have now re-generated the plots using Cooltools as shown below, obtaining results consistent with the style shown in PMID 41107486. Importantly, these reanalyses do not affect our conclusions.

< Reviewer's Figure 2 >
The plots using Cooltools related to Figs. 3E and EV3C (old EV8C).

<u>Effect of aging on the chromatin interaction frequency</u>

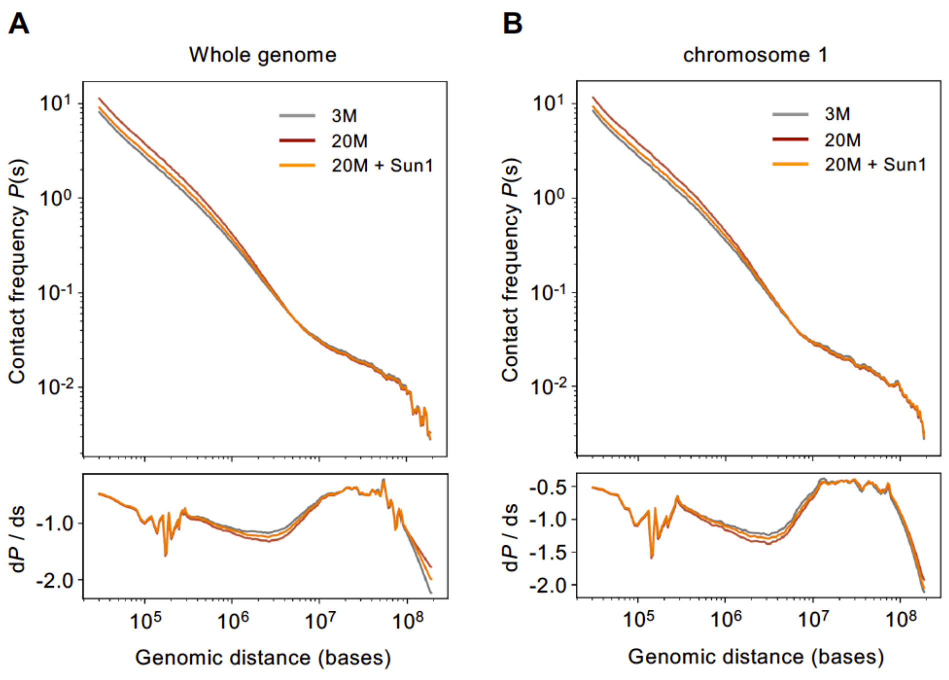

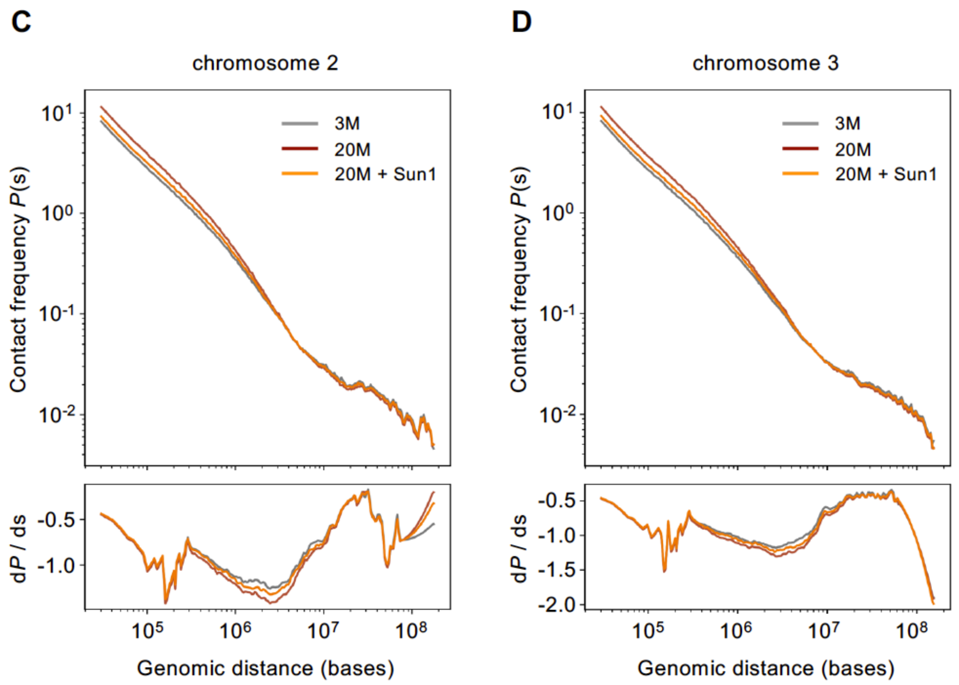

(A-D) Distance-dependent contact frequency profiles across the whole genome (A), and on chromosome 1-3 (B-D) in AAV-eGFP-Cre alone (3M or 20M) and AAV-eGFP-Cre + AAV-Sun1 (20M + Sun1) groups. The first derivatives of those *P*(s) curves are presented in the lower panel.

2) The Bioinformatics analysis in the Methods section should include more details regarding the z-score calculations, A/B compartment analysis, and contact frequency curve plotting.

Response

In response to the reviewer's comments, we have added detailed descriptions of the three analyses in the Methods.

"z-score calculations" (page 41, line 13 to line 20)

Original: … GO analysis of DEGs was performed in ShinyGO (v0.82) (Ge *et al*, 2020), and heatmaps were visualized using the online tool SRplot (Tang *et al*, 2023).

↓

Revised: … GO analysis of DEGs was performed in ShinyGO (v0.82) (Ge *et al*, 2020), in which p-values were derived from hypergeometric tests and adjusted using Benjamini-Hochberg method. For visualization of gene expression heatmaps (Figs. 3J and EV4C), FPKM values were calculated by RSEM and subsequently subjected to z-score

normalization on a per-gene basis. Specifically, for each gene, the mean FPKM across all nine samples (three biological replicates per condition) was subtracted, and the result was divided by the standard deviation across those samples, yielding a z-score for each sample. Heatmaps were generated using SRplot (Tang *et al*, 2023) with the gene-wise z-score values.

"contact frequency curve plotting" (page 42, line 4 to line 17)

Original: … Visualization included chromatin interaction heatmaps generated by hicPlotMatrix and distance-dependent contact frequency profiles plotted with hicPlotDistVsCounts.

↓

Revised: … Chromatin interaction heatmaps generated by hicPlotMatrix module of HiCExplorer. Distance-dependent contact frequency profiles were computed using the hicPlotDistVsCounts module of HiCExplorer at 10-kb resolution, retaining only intra-chromosomal contacts at genomic distances ≥ 10 kb, and log-transformed prior to plotting to allow comparison across samples. To quantify the rate of change in contact frequency with increasing genomic distance, the first derivative of contact frequency with respect to genomic distance (d(Contacts)/d(Distance)) was calculated using a custom Python script. Briefly, contact frequency profiles were first smoothed using the Savitzky–Golay filter (window size = 51 bins, polynomial order = 3) as implemented in NumPy (Harris et al, 2020) to suppress high-frequency noise while preserving the overall decay structure, and the first derivative was subsequently computed via finite differences using numpy.gradient. Derivative profiles were plotted as a function of genomic distance on a log-scaled axis to visualize differences in chromatin compaction dynamics across conditions.

"A/B compartment" (page 42, line 17 to line 23)

Original: … Compartment analysis involved calculation of the first principal component (PC1) values through hicPCA (*--format bedgraph -we 1*), with genomic regions classified as A/B compartments based on PC1 sign (+/–).

↓

Revised: … For A/B compartment analysis, contact matrices were generated at 100-kb resolution. Principal component analysis (PCA) was performed on the observed/expected contact matrices using the hicPCA module (--format bedgraph -we 1), and the first principal component (PC1) values were used to define compartment identity. The sign of PC1 was assigned such that genomic regions with higher gene density and active histone marks corresponded to compartment A (PC1 > 0), and regions with repressive marks at the nuclear periphery corresponded to compartment B (PC1 < 0).

3) While the authors explain their rationale for reporting z-scores for differential gene analysis, it would still be informative to include a plot of log(2) fold change of all genes comparing 3M vs 20M vs 20M+Sun1 and report total number of genes upregulated vs downregulated between the conditions. Based on the rescue phenotype of 20M+Sun1, wouldn't it be expected that 3M vs 20M+Sun1 would have fewer DEGs compared to 3M vs 20M or 20M vs 20M+Sun1?

Response

We thank the reviewer for this constructive suggestion. We have now prepared volcano plots for all pairwise comparisons (3M vs 20M, 3M vs 20M+Sun1, and 20M vs 20M+Sun1), along with the total numbers of upregulated and downregulated DEGs under two threshold schemes (loose: $|log_2FC| > 1$, $p < 0.01$; stringent: $|log_2FC| > 2$, $p < 0.001$) (Reviewer's Figure 3, on the next page). Regarding the reviewer's expectation that 3M vs 20M+Sun1 should yield fewer DEGs than either 3M vs 20M or 20M vs 20M+Sun1—consistent with a rescue phenotype—we find that this expectation is supported under the stringent threshold, where DEG counts are 3M vs 20M: 179, 20M vs 20M+Sun1: 76, and 3M vs 20M+Sun1: 66. Under the stringent criterion, 3M vs 20M+Sun1 indeed shows the fewest differentially expressed genes, suggesting that the transcriptome of 20M+Sun1 cells most closely resembles that of 3M cells at high-confidence loci. However, under the loose threshold, the ranking shifts slightly: 3M vs 20M: 1,063, 20M vs 20M+Sun1: 926, and 3M vs 20M+Sun1: 1,125, with 3M vs 20M+Sun1 showing marginally more DEGs than the other comparisons. This apparent inconsistency likely reflects the inclusion of many low-effect, borderline-significant features at loose thresholds, which can be dominated by stochastic noise and threshold sensitivity rather than biology. Taken together, the stringent threshold results are consistent with the rescue interpretation: 20M + Sun1 restores the transcriptional landscape toward a 3M-like state.

< Reviewer's Figure 3 >

Effect of aging on the expression correlations

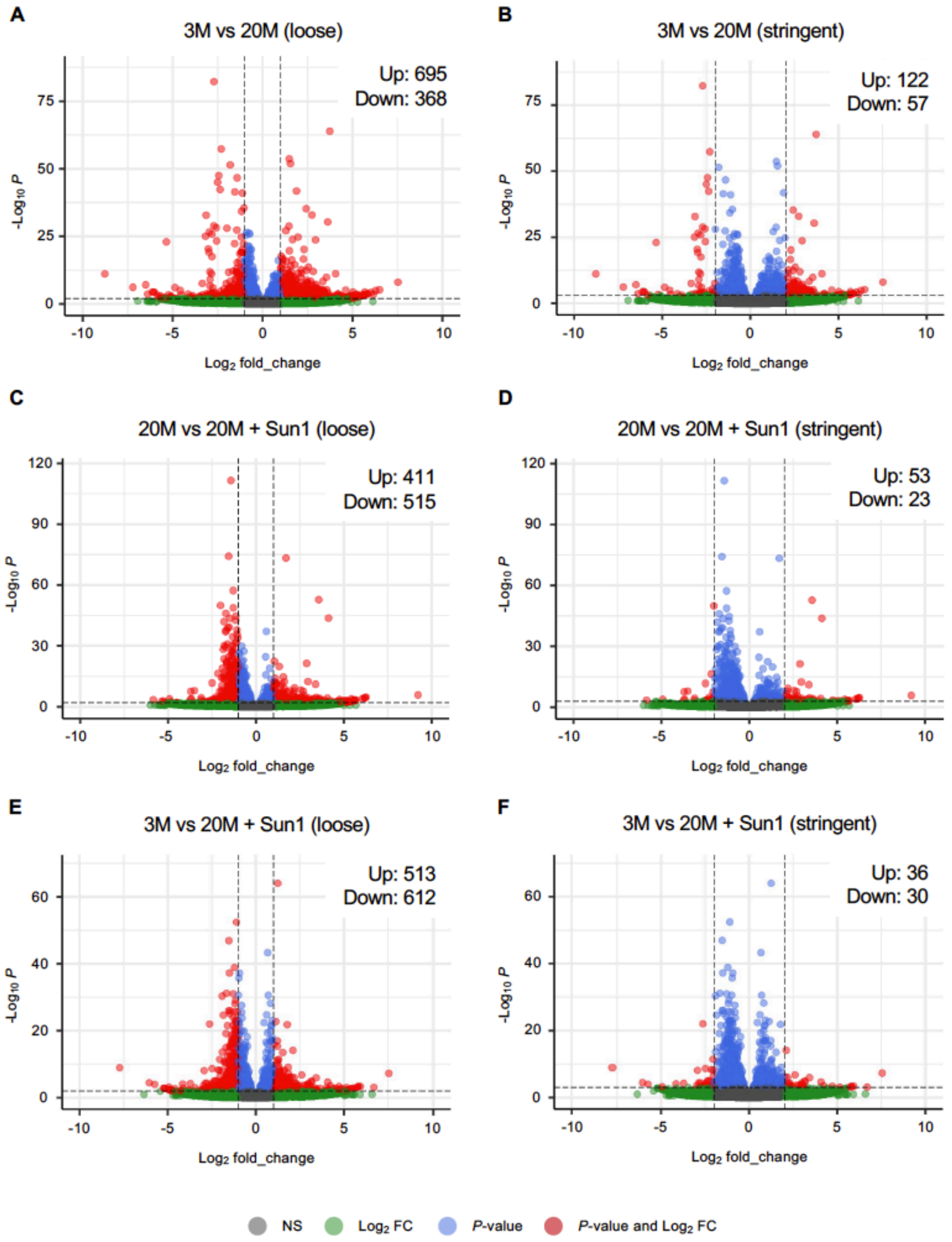

(A-F) Volcano plot of gene expression between two indicated groups [AAV-eGFP-Cre alone (3M or 20M); AAV-eGFP-Cre + AAV-Sun1 (20M + Sun1)]. DEGs were defined as those with $|\log_2 FC| > 1$, $P<0.01$ (loose, A, C, E) or $|\log_2 FC| > 2$, $P<0.001$ (stringent, B, D, F). Total variables are 78202. NS, not significant; FC, fold_change.

4) The authors mentioned in their response to reviewers that they are working on a future manuscript containing ChIP-seq and ATAC-seq. Given this information, my recommendation is to consider incorporating the Hi-C data from this manuscript in the future manuscript instead. The contribution from the Hi-C data as it stands is that the overall genome architecture (at compartment scale) may shift towards that of younger neurons. However, as pointed out in my original review (point #8), this is not a strong result, and with more detailed and thorough analyses the authors may be able to gain more insights into the finer scale interactions that are altered. In addition, the paper is already very long with a lot of data and methods, and my view is that the main conclusions are not impacted in the absence of the 3D genomics data.

Response

We appreciate the reviewer's suggestion. From the perspective of genomic research, we acknowledge that the current Hi-C dataset, by itself, does not provide detailed insights into age-related epigenomic changes. However, from the cell biological perspective of neuronal aging addressed in this study, our Hi-C dataset depicting age-related alterations in chromatin dynamics is indispensable for demonstrating the cellular mechanisms linking nuclear structural abnormalities to downstream changes in gene expression. Therefore, we have retained the Hi-C data in this manuscript.

Prof. Ken-ichiro Kuwako
Shimane University
School of Medicine
89-1 Enya-cho
Izumo-shi, Shimane 693-8501
Japan

Dear Prof. Kuwako,

I am very pleased to accept your manuscript for publication in the next available issue of EMBO reports. Thank you for your contribution to our journal.

Your manuscript will be processed for publication by EMBO Press. It will be copy edited and you will receive page proofs prior to publication. Please note that you will be contacted by Springer Nature Author Services to complete licensing and payment information.

You may qualify for financial assistance for your publication charges - either via a Springer Nature fully open access agreement or an EMBO initiative. Check your eligibility: https://link.springer.com/journal/44319/how-to-publish-with-us

Should you be planning a Press Release on your article, please get in contact with embo_production@springernature.com as early as possible in order to coordinate publication and release dates.

If you have any questions, please do not hesitate to contact the Editorial Office. Thank you for your contribution to EMBO Reports.

Yours sincerely,

Martina Rembold, PhD
Senior Editor
EMBO reports

>>> Please note that it is EMBO Reports policy for the transcript of the editorial process (containing referee reports and your response letter) to be published as an online supplement to each paper. If you do NOT want this, you will need to inform the Editorial Office via email immediately. More information is available here: https://link.springer.com/partners/embo-press/editorial-policies#Peer%20review

EMBO Press Author Checklist

Corresponding Author Name: Ken-ichiro Kuwako	
Journal Submitted to: EMBO Reports	
Manuscript Number: EMBOR-2025-62812V2	

USEFUL LINKS FOR COMPLETING THIS FORM
The EMBO Journal - Author Guidelines
EMBO Reports - Author Guidelines
Molecular Systems Biology - Author Guidelines
EMBO Molecular Medicine - Author Guidelines

Reporting Checklist for Life Science Articles (updated January
This checklist is adapted from Materials Design Analysis Reporting (MDAR) Checklist for Authors. MDAR establishes a minimum set of requirements in transparent reporting in the life sciences (see Statement of Task: 10.31222/osf.io/9sm4x). Please follow the journal's guidelines in preparing your
Please note that a copy of this checklist will be published alongside your article.

Abridged guidelines for figures
1. Data
The data shown in figures should satisfy the following conditions:
- ➞ the data were obtained and processed according to the field's best practice and are presented to reflect the results of the experiments in an accurate and unbiased manner.
- ➞ ideally, figure panels should include only measurements that are directly comparable to each other and obtained with the same assay.
- ➞ plots include clearly labeled error bars for independent experiments and sample sizes. Unless justified, error bars should not be shown for technical
- ➞ if n<5, the individual data points from each experiment should be plotted. Any statistical test employed should be justified.
- ➞ Source Data should be included to report the data underlying figures according to the guidelines set out in the authorship guidelines on Data

2. Captions
Each figure caption should contain the following information, for each panel where they are relevant:
- ➞ a specification of the experimental system investigated (eg cell line, species name).
- ➞ the assay(s) and method(s) used to carry out the reported observations and measurements.
- ➞ an explicit mention of the biological and chemical entity(ies) that are being measured.
- ➞ an explicit mention of the biological and chemical entity(ies) that are altered/varied/perturbed in a controlled manner.
- ➞ the exact sample size (n) for each experimental group/condition, given as a number, not a range;
- ➞ a description of the sample collection allowing the reader to understand whether the samples represent technical or biological replicates (including how many animals, litters, cultures, etc.).
- ➞ a statement of how many times the experiment shown was independently replicated in the laboratory.
- ➞ definitions of statistical methods and measures:
  - common tests, such as t-test (please specify whether paired vs. unpaired), simple χ2 tests, Wilcoxon and Mann-Whitney tests, can be unambiguously identified by name only, but more complex techniques should be described in the methods section;
  - are tests one-sided or two-sided?
  - are there adjustments for multiple comparisons?
  - exact statistical test results, e.g., P values = x but not P values < x;
  - definition of 'center values' as median or average;
  - definition of error bars as s.d. or s.e.m.

Please complete ALL of the questions below.
Select "Not Applicable" only when the requested information is not relevant for your study.

Materials

Newly Created Materials	Information included in the manuscript?	In which section is the information available? (Reagents and Tools Table, Materials and Methods, Figures, Data Availability Section)
New materials and reagents need to be available; do any restrictions apply?	Not Applicable	

Antibodies	Information included in the manuscript?	In which section is the information available? (Reagents and Tools Table, Materials and Methods, Figures, Data Availability Section)
For **antibodies** provide the following information:<br>- Commercial antibodies: RRID (if possible) or supplier name, catalogue number and or/clone number<br>- Non-commercial: RRID or citation	Yes	Reagents and Tools Table

DNA and RNA sequences	Information included in the manuscript?	In which section is the information available? (Reagents and Tools Table, Materials and Methods, Figures, Data Availability Section)
**Short novel DNA or RNA including primers, probes**: provide the sequences.	Yes	Reagents and Tools Table, Appendix

Cell materials	Information included in the manuscript?	In which section is the information available? (Reagents and Tools Table, Materials and Methods, Figures, Data Availability Section)
**Cell lines:** Provide species information, strain. Provide accession number in repository **OR** supplier name, catalog number, clone number, and/**OR** RRID.	Yes	Reagents and Tools Table
**Primary cultures:** Provide species, strain, sex of origin, genetic modification status.	Yes	Mehods
Report if the cell lines were recently **authenticated** (e.g., by STR profiling) and tested for mycoplasma contamination.	Not Applicable	

Experimental animals	Information included in the manuscript?	In which section is the information available? (Reagents and Tools Table, Materials and Methods, Figures, Data Availability Section)
**Laboratory animals or Model organisms:** Provide species, strain, sex, age, genetic modification status. Provide accession number in repository **OR** supplier name, catalog number, clone number, **OR** RRID.	Yes	Reagents and Tools Table
**Animal observed in or captured from the field:** Provide species, sex, and age where possible**.**	Not Applicable	
Please detail **housing and husbandry conditions**.	Yes	Mehods

Plants and microbes	Information included in the manuscript?	In which section is the information available? (Reagents and Tools Table, Materials and Methods, Figures, Data Availability Section)
**Plants:** provide species and strain, ecotype and cultivar where relevant, unique accession number if available, and source (including location for collected wild specimens).	Not Applicable	
**Microbes:** provide species and strain, unique accession number if available, and source.	Not Applicable	

Human research participants	Information included in the manuscript?	In which section is the information available? (Reagents and Tools Table, Materials and Methods, Figures, Data Availability Section)
If collected and within the bounds of privacy constraints report on age, sex and gender or ethnicity for all study participants.	Not Applicable	

Core facilities	Information included in the manuscript?	In which section is the information available? (Reagents and Tools Table, Materials and Methods, Figures, Data Availability Section)
If your work benefited from core facilities, was their service mentioned in the acknowledgments section?	Yes	Acknowledgements

Design

Study protocol	Information included in the manuscript?	In which section is the information available? (Reagents and Tools Table, Materials and Methods, Figures, Data Availability Section)
If study protocol has been **pre-registered, provide DOI in the manuscript**. For clinical trials, provide the trial registration number **OR** cite DOI.	Not Applicable	
Report the **clinical trial registration number** (at ClinicalTrials.gov or equivalent), where applicable.	Not Applicable	

Laboratory protocol	Information included in the manuscript?	In which section is the information available? (Reagents and Tools Table, Materials and Methods, Figures, Data Availability Section)
Provide DOI OR other citation details if **external detailed step-by-step protocols** are available.	Not Applicable	

Experimental study design and statistics	Information included in the manuscript?	In which section is the information available? (Reagents and Tools Table, Materials and Methods, Figures, Data Availability Section)
Include a statement about **sample size** estimate even if no statistical methods were used.	Yes	Mehods
Were any steps taken to minimize the effects of subjective bias when allocating animals/samples to treatment (e.g. **randomization procedure**)? If yes, have they been described?	Not Applicable	
Include a statement about **blinding** even if no blinding was done.	Yes	Methods
Describe **inclusion/exclusion criteria** if samples or animals were excluded from the analysis. Were the criteria pre-established?<br><br>If sample or data points were omitted from analysis, report if this was due to attrition or intentional exclusion and provide justification.	Yes	Methods
For every figure, are **statistical tests** justified as appropriate? Do the data meet the assumptions of the tests (e.g., normal distribution)? Describe any methods used to assess it. Is there an estimate of variation within each group of data? Is the variance similar between the groups that are being statistically compared?	Yes	Figures

Sample definition and in-laboratory replication	Information included in the manuscript?	In which section is the information available? (Reagents and Tools Table, Materials and Methods, Figures, Data Availability Section)
In the figure legends: state number of times the experiment was **replicated** in laboratory.	Yes	Figures
In the figure legends: define whether data describe **technical or biological replicates**.	Yes	Figures

Ethics

Ethics	Information included in the manuscript?	In which section is the information available? (Reagents and Tools Table, Materials and Methods, Figures, Data Availability Section)
Studies involving **human participants**: State details of **authority granting ethics approval** (IRB or equivalent committee(s), provide reference number for approval.	Not Applicable	
Studies involving **human participants**: Include a statement confirming that **informed consent** was obtained from all subjects and that the experiments conformed to the principles set out in the WMA Declaration of Helsinki and the Department of Health and Human Services Belmont Report.	Not Applicable	
Studies involving **human participants:** For publication of **patient photos**, include a statement confirming that consent to publish was obtained.	Not Applicable	
Studies involving experimental **animals**: State details of **authority granting ethics approval** (IRB or equivalent committee(s), provide reference number for approval. Include a statement of compliance with ethical regulations.	Yes	Methods
Studies involving **specimen and field samples:** State if relevant **permits** obtained, provide details of authority approving study; if none were required, explain why.	Not Applicable	

Dual Use Research of Concern (DURC)	Information included in the manuscript?	In which section is the information available? (Reagents and Tools Table, Materials and Methods, Figures, Data Availability Section)
Could your study fall under dual use research restrictions? Please check biosecurity documents and list of **select agents and toxins** (CDC): https://www.selectagents.gov/sat/list.htm	Not Applicable	
If you used a select agent, is the security level of the lab appropriate and reported in the manuscript?	Not Applicable	
If a study is subject to dual use research of concern regulations, is the name of the **authority granting approval and reference number** for the regulatory approval provided in the manuscript?	Not Applicable	

Reporting

The MDAR framework recommends adoption of discipline-specific guidelines, established and endorsed through community initiatives. Journals have their own policy about requiring specific guidelines and recommendations to complement MDAR.

Adherence to community standards	Information included in the manuscript?	In which section is the information available? (Reagents and Tools Table, Materials and Methods, Figures, Data Availability Section)
State if relevant guidelines or checklists (e.g., **ICMJE, MIBBI, ARRIVE, PRISMA**) have been followed or provided.	Not Applicable	
For **tumor marker prognostic studies,** we recommend that you follow the **REMARK** reporting guidelines (see link list at top right). See author guidelines, under 'Reporting Guidelines'. Please confirm you have followed these guidelines.	Not Applicable	
For **phase II and III randomized controlled trials**, please refer to the **CONSORT** flow diagram (see link list at top right) and submit the CONSORT checklist (see link list at top right) with your submission. See author guidelines, under 'Reporting Guidelines'. Please confirm you have submitted this list.	Not Applicable	

Data Availability

Data availability	Information included in the manuscript?	In which section is the information available? (Reagents and Tools Table, Materials and Methods, Figures, Data Availability Section)
Have **primary datasets** been deposited according to the journal's guidelines (see 'Data Deposition' section) and the respective accession numbers provided in the Data Availability Section?	Yes	Data Availability Section
Were **human clinical and genomic datasets** deposited in a public access-controlled repository in accordance to ethical obligations to the patients and to the applicable consent agreement?	Not Applicable	
Are **computational models** that are central and integral to a study available without restrictions in a machine-readable form? Were the relevant accession numbers or links provided?	Not Applicable	
If publicly available data were reused, provide the respective **data citations in the reference list**.	Not Applicable	
